# Supplementary material for: Dysregulated mesenchymal PDGFR‐β drives kidney fibrosis
Source: EMBO Mol Med. 2020 Jan 14;12(3):e11021. doi: 10.15252/emmm.201911021 (PMC7059015; doi:10.15252/emmm.201911021)
Supplement: Supplementary file 1 — Appendix [file EMMM-12-e11021-s001.pdf]

Appendix

Table of contents

Appendix Table S1: Genotype distribution of litters in the Foxd1*Cre::Pdgfrb*$^{+/J}$ line

Appendix Table S2: Genotype distribution of litters in the Foxd1*Cre::Pdgfrb*$^{fl/fl}$ line

Appendix Table S3: Genotype distribution of litters in the Pax8*Cre::Pdgfrb*$^{+/J}$ line

Appendix Table S4: p-values

Appendix Figure S1: FoxD1 expression and PDGFR-β staining co-localize in the kidney

Appendix Figure S2: *Pdgfrb* hemizygous mice do not show a gene-dose effect

Appendix Figure S3: Activation of PDGFR-β in mesenchymal cells in *Foxd1Cre::Pdgfrb*$^{+/J}$ mice results in progressive proliferative glomerulonephritis and mesangial glomerulosclerosis

Appendix Figure S4: Transmission electron microscopy of wt and *Foxd1Cre::Pdgfrb* mice

Appendix Figure S5: Interstitial changes in time course of *Foxd1Cre::Pdgfrb*$^{+/J}$ mice

Appendix Figure S6: Interstitial changes in the medulla in time course of *Foxd1Cre::Pdgfrb*$^{+/J}$ mice

Appendix Figure S7: Transmission electron microscopy of the interstitium of wt and *Foxd1Cre::Pdgfrb*$^{+/J}$ mice

Appendix Figure S8: The renal capsule undergoes fibrous thickening in *Foxd1Cre::Pdgfrb*$^{+/J}$ mice

Appendix Figure S9: Quantitative real-time PCR confirm array data

Appendix Figure S10: Tubular cell-specific activation of PDGFR-β signaling had no effects

Appendix Table S1: Genotype distribution of litters in the Foxd1*Cre::Pdgfrb*$^{+/J}$ line

In total 608 born mice were analyzed for numbers and percentage (%) of possible genotypes resulting from the respective breeding scheme. They were born in the expected Mendelian ratio (expected %), albeit slight deviations were observed with reduced numbers of *Foxd1*$^{+/cre}$ *Pdgfrb*$^{+/J}$ (-4%) and slightly higher numbers of *Foxd1*$^{+/+}$ *Pdgfrb*$^{+/+}$ mice (+6%). The main genotype analyzed in the study expressing the mutated PDGFR-β is highlighted in grey.

Breeding scheme: *Foxd1*$^{+/cre}$ *Pdgfrb*$^{+/+}$ x *Foxd1*$^{+/+}$ *Pdgfrb*$^{+/J}$

Genotypes	Number of born mice	%	expected (%)
Foxd1$^{+/+}$ *Pdgfrb*$^{+/+}$	189	**31.1**	25
Foxd1$^{+/+}$ *Pdgfrb*$^{+/J}$	154	**25.3**	25
Foxd1$^{+/cre}$ *Pdgfrb*$^{+/+}$	138	**22.7**	25
Foxd1$^{+/cre}$ *Pdgfrb*$^{+/J}$	127	**20.9**	25
Total	608	100	100

Appendix Table S2: Genotype distribution of litters in the Foxd1*Cre::Pdgfrb*$^{fl/fl}$ line

In total 288 born mice were analyzed for numbers and percentage (%) of possible genotypes resulting from the respective breeding scheme. They were born in the expected Mendelian ratio (expected %), with slight variations of up to 5%. The main genotype analyzed in the study with *Foxd1*-dependent PDGFR-β knockout is highlighted in grey.

Breeding scheme: *Foxd1*$^{+/cre}$ *Pdgfrb*$^{+/fl}$ x *Foxd1*$^{+/+}$ *Pdgfrb*$^{+/fl}$

Genotypes	Number of born mice	%	expected (%)
Foxd1$^{+/+}$ *Pdgfrb*$^{+/+}$	29	**10.1**	12.5
Foxd1$^{+/+}$ *Pdgfrb*$^{+/fl}$	62	**21.5**	25
Foxd1$^{+/+}$ *Pdgfrb*$^{fl/fl}$	53	**18.4**	12.5
Foxd1$^{+/cre}$ *Pdgfrb*$^{+/+}$	43	**14.9**	12.5
Foxd1$^{+/cre}$ *Pdgfrb*$^{+/fl}$	57	**19.8**	25
Foxd1$^{+/cre}$ *Pdgfrb*$^{fl/fl}$	44	**15.3**	12.5
total	288	100	100

Appendix Table S3: Genotype distribution of litters in the Pax8*Cre::Pdgfrb*$^{+/J}$ line

In total 345 born mice were analyzed for numbers and percentage (%) of possible genotypes resulting from the respective breeding scheme. They were born in the expected Mendelian ratio (expected %). The main genotype analyzed in the study expressing the mutated PDGFR-β is highlighted in grey.

Breeding scheme: *Pax8*$^{+/cre}$ *Pdgfrb*$^{+/+}$ x *Pax8*$^{+/+}$ *Pdgfrb*$^{+/J}$			
Genotypes	Number of born mice	%	expected (%)
Pax8$^{+/+}$ *Pdgfrb*$^{+/+}$	93	**27.0**	25
Pax8$^{+/+}$ *Pdgfrb*$^{+/J}$	83	**24.1**	25
Pax8$^{+/cre}$ *Pdgfrb*$^{+/+}$	79	**22.9**	25
Pax8$^{+/cre}$ *Pdgfrb*$^{+/J}$	90	**26.1**	25
total	345	100	100

Appendix Table S4: p-values

		p-values
Figure 1A	PDGFR-β	0.0006
	p-PDGFR-β	0.0202
	α-SMA	0.0037
Figure 2C	week 6 wt vs. week 6 Foxd1Cre::Pdgfrb+/J	0.2169
	week 14 wt vs. week 14 Foxd1Cre::Pdgfrb+/J	0.0027
	week 25 wt vs. week 25 Foxd1Cre::Pdgfrb+/J	0.0621
	week 35 wt vs. week 35 Foxd1Cre::Pdgfrb+/J	0.0758
Figure 2D	week 6 wt vs. week 6 Foxd1Cre::Pdgfrb+/J	0.1460
	week 14 wt vs. week 14 Foxd1Cre::Pdgfrb+/J	0.0438
	week 25 wt vs. week 25 Foxd1Cre::Pdgfrb+/J	0.0870
	week 35 wt vs. week 35 Foxd1Cre::Pdgfrb+/J	0.0664
Figure 2E	week 6 wt vs. week 6 Foxd1Cre::Pdgfrb+/J	0.3517
	week 14 wt vs. week 14 Foxd1Cre::Pdgfrb+/J	0.3419
	week 25 wt vs. week 25 Foxd1Cre::Pdgfrb+/J	0.5634
	week 35 wt vs. week 35 Foxd1Cre::Pdgfrb+/J	0.2492
Figure 2G	wt vs. Foxd1Cre::Pdgfrb+/J	0.0446
Figure 2F	Fibroblasts wt vs. Fibroblasts Foxd1Cre::Pdgfrb+/J	< 0.0001
	Mesangial cells wt vs. Mesangial cells Foxd1Cre::Pdgfrb+/J	< 0.0001
Figure 3B	week 6 wt vs. week 6 Foxd1Cre::Pdgfrb+/J	0.0004
	week 14 wt vs. week 14 Foxd1Cre::Pdgfrb+/J	0.0207
	week 25 wt vs. week 25 Foxd1Cre::Pdgfrb+/J	0.1312
	week 35 wt vs. week 35 Foxd1Cre::Pdgfrb+/J	0.0355
Figure 3D	week 6 wt vs. week 6 Foxd1Cre::Pdgfrb+/J	0.0035
	week 14 wt vs. week 14 Foxd1Cre::Pdgfrb+/J	0.0468
	week 25 wt vs. week 25 Foxd1Cre::Pdgfrb+/J	0.0239
	week 35 wt vs. week 35 Foxd1Cre::Pdgfrb+/J	0.0145
Figure 3F	week 6 wt vs. week 6 Foxd1Cre::Pdgfrb+/J	0.1841
	week 14 wt vs. week 14 Foxd1Cre::Pdgfrb+/J	0.0054
	week 25 wt vs. week 25 Foxd1Cre::Pdgfrb+/J	0.0150
	week 35 wt vs. week 35 Foxd1Cre::Pdgfrb+/J	0.0196

		p-values
Figure 4B	week 6 wt vs. week 6 Foxd1Cre::Pdgfrb+/J	0.0386
	week 14 wt vs. week 14 Foxd1Cre::Pdgfrb+/J	0.2327
	week 25 wt vs. week 25 Foxd1Cre::Pdgfrb+/J	0.0082
	week 35 wt vs. week 35 Foxd1Cre::Pdgfrb+/J	0.0581
Figure 4D	week 6 wt vs. week 6 Foxd1Cre::Pdgfrb+/J	0.0123
	week 14 wt vs. week 14 Foxd1Cre::Pdgfrb+/J	0.0009
	week 25 wt vs. week 25 Foxd1Cre::Pdgfrb+/J	< 0.0001
	week 35 wt vs. week 35 Foxd1Cre::Pdgfrb+/J	0.0061
Figure 4F	week 6 wt vs. week 6 Foxd1Cre::Pdgfrb+/J	0.1444
	week 14 wt vs. week 14 Foxd1Cre::Pdgfrb+/J	0.0437
	week 25 wt vs. week 25 Foxd1Cre::Pdgfrb+/J	0.0947
	week 35 wt vs. week 35 Foxd1Cre::Pdgfrb+/J	0.0808
Figure 4H	week 6 wt vs. week 6 Foxd1Cre::Pdgfrb+/J	0.3144
	week 14 wt vs. week 14 Foxd1Cre::Pdgfrb+/J	0.0277
	week 25 wt vs. week 25 Foxd1Cre::Pdgfrb+/J	0.0907
	week 35 wt vs. week 35 Foxd1Cre::Pdgfrb+/J	0.0052
Figure 5B	week 10 wt vs. week 10 Foxd1Cre::Pdgfrb+/J	0.0022
	week 14 wt vs. week 14 Foxd1Cre::Pdgfrb+/J	0.0317
	week 25 wt vs. week 25 Foxd1Cre::Pdgfrb+/J	0.0787
	week 35 wt vd. week 35 Foxd1Cre::Pdgfrb+/J	0.0572
Figure 6A	week 6 wt vs. week 6 Foxd1Cre::Pdgfrb+/J	> 0.9999
	week 14 wt vs. week 14 Foxd1Cre::Pdgfrb+/J	0.0097
	week 25 wt vs. week 25 Foxd1Cre::Pdgfrb+/J	0.0005
	week 35 wt vs. week 35 Foxd1Cre::Pdgfrb+/J	0.0003
Figure 6B	week 6 wt vs. week 6 Foxd1Cre::Pdgfrb+/J	0.8300
	week 14 wt vs. week 14 Foxd1Cre::Pdgfrb+/J	0.0063
	week 25 wt vs. week 25 Foxd1Cre::Pdgfrb+/J	0.0003
	week 35 wt vs. week 35 Foxd1Cre::Pdgfrb+/J	0.0006
Figure 6C	week 6 wt vs. week 6 Foxd1Cre::Pdgfrb+/J	0.6937
	week 14 wt vs. week 14 Foxd1Cre::Pdgfrb+/J	0.0134
	week 25 wt vs. week 25 Foxd1Cre::Pdgfrb+/J	0.0003
	week 35 wt vs. week 35 Foxd1Cre::Pdgfrb+/J	0.0010
Figure 7A	aSMA wt UUO vs. Foxd1Cre::Pdgfrb+/J UUO	0.0101
	collagen I wt UUO vs. Foxd1Cre::Pdgfrb+/J UUO	0.0023
	F4/80 wt UUO vs. Foxd1Cre::Pdgfrb+/J UUO	0.0042

		p-values
Figure 7C	wt Ang II d0 vs. wt Ang II d28	< 0,0001
	Foxd1Cre::Pdgfrb+/J Ang II d0 vs. Foxd1Cre::Pdgfrb+/J Ang II d28	< 0,0001
Figure 7D	wt Ang II d0 vs. wt Ang II d28	0,0227
	Foxd1Cre::Pdgfrb+/J Ang II d0 vs. Foxd1Cre::Pdgfrb+/J Ang II d28	0,0119
	wt Ang II d28 vs. Foxd1Cre::Pdgfrb+/J Ang II d28	0,0038
	Foxd1Cre::Pdgfrb+/J Ang II d28 vs. Foxd1Cre::Pdgfrb+/J ctrl	0,0428
Figure 7E	wt Ang II d0 vs. wt Ang II d28	0,9314
	Foxd1Cre::Pdgfrb+/J Ang II d0 vs. Foxd1Cre::Pdgfrb+/J Ang II d28	0,0669
	wt Ang II d28 vs. Foxd1Cre::Pdgfrb+/J Ang II d28	0,2113
	Foxd1Cre::Pdgfrb+/J Ang II d28 vs. Foxd1Cre::Pdgfrb+/J ctrl	0,0227
Figure 7H	wt Ang II vs. Foxd1Cre::Pdgfrb+/J Ang II	0.0353
	Foxd1Cre::Pdgfrb+/J Ang II vs. Foxd1Cre::Pdgfrb+/J ctrl	< 0.0001
Figure 7I	Foxd1Cre::Pdgfrb+/J + water vs. Foxd1Cre::Pdgfrb+/J + imatinib	0.0029
Figure 7J	Foxd1Cre::Pdgfrb+/J + water vs. Foxd1Cre::Pdgfrb+/J + imatinib	0.0109
Figure 7K	Foxd1Cre::Pdgfrb+/J + water vs. Foxd1Cre::Pdgfrb+/J + imatinib	0.3215
Figure 7L	Foxd1Cre::Pdgfrb+/J + water vs. Foxd1Cre::Pdgfrb+/J + imatinib	0.0387
Figure 7P	Foxd1Cre::Pdgfrb+/J + water vs. Foxd1Cre::Pdgfrb+/J + imatinib	0.0139
Figure EV5E	wt vs. Foxd1Cre::Pdgfrb fl/fl	0.0225
Figure EV5F	wt vs. Foxd1Cre::Pdgfrb fl/fl	0.0053
Figure EV5G	wt vs. Foxd1Cre::Pdgfrb fl/fl	< 0.0001
Figure EV1D	p-PDGFRb wt vs. p-PDGFRb Foxd1Cre::Pdgfrb+/J	0.0047
	p-Akt wt vs. p-Akt Foxd1Cre::Pdgfrb+/J	0.0039
	p-p38 wt vs. p-p38 Foxd1Cre::Pdgfrb+/J	0.0013
	p-PLCg wt vs. p-PLCg Foxd1Cre::Pdgfrb+/J	0.1449
	p-ERK1/2 wt vs. p-ERK1/2 Foxd1Cre::Pdgfrb+/J	0.6431
	p-JNK wt vs. p-JNK Foxd1Cre::Pdgfrb+/J	0.1561
Appendix Figure S8	week 6 wt vs. week 6 Foxd1Cre::Pdgfrb+/J vs. week 6 Foxd1Cre::Pdgfrb+/J	0.0269
	week 14 wt vs. week 14 Foxd1Cre::Pdgfrb+/J vs. week 14 Foxd1Cre::Pdgfrb+/J	0.0006
	week 25 wt vs. week 25 Foxd1Cre::Pdgfrb+/J vs. week 25 Foxd1Cre::Pdgfrb+/J	0.0182
	week 35 wt vs. week 35 Foxd1Cre::Pdgfrb+/J	0.0242
Appendix Figure S9	Ifit wt vs. Ifit Foxd1Cre::Pdgfrb+/J	0.0130
	Irf8 wt vs. Irf8 Foxd1Cre::Pdgfrb+/J	0.1262
	Casp1 wt vs. Casp1 Foxd1Cre::Pdgfrb+/J	0.0243
	Trim30d wt vs. Trim30d Foxd1Cre::Pdgfrb+/J	0.0938

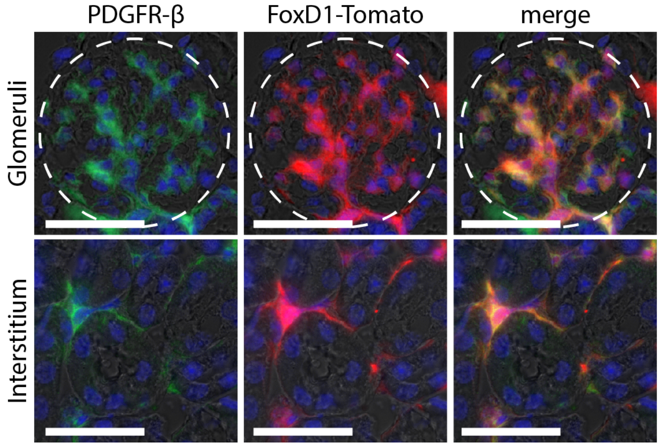

Appendix Figure S1: FoxD1 expression and PDGFR-β staining co-localize in the kidney
Glomerular mesangial cells and interstitial fibroblasts express PDGFR-β, as shown by immunofluorescence staining (green) and originate nearly completely from *Foxd1*-progenitor cells as shown using FoxD1 reporter mice (red; *Foxd1Cre::tdTomato*). Only very few cells were not double positive, suggesting that *Foxd1-Cre* mice are highly suitable to target the PDGFR-β cell population in kidneys. Circle outlines a glomerulus. Scale bars: 50 µm

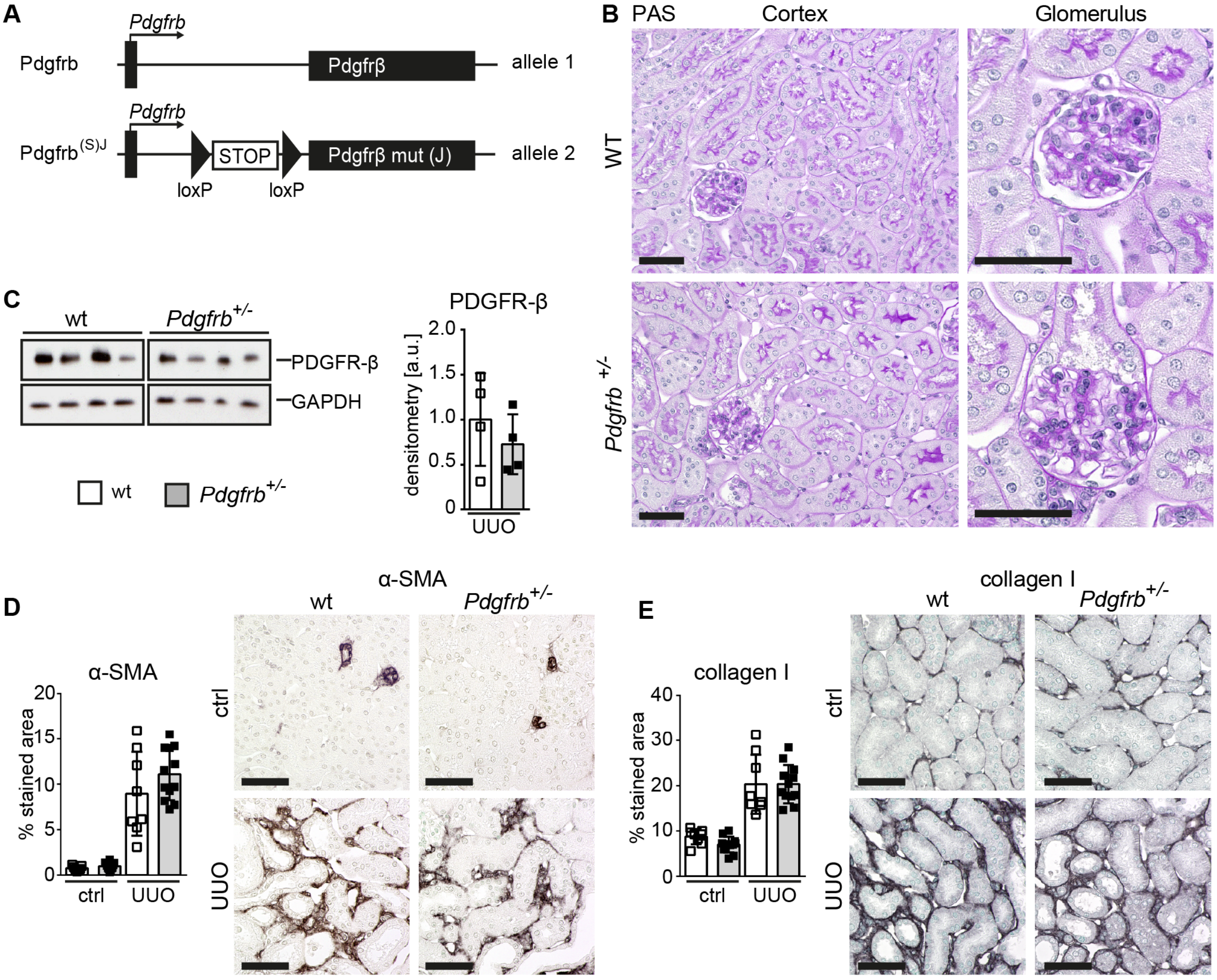

Appendix Figure S2: *Pdgfrb* hemizygous mice do not show a gene-dose effect

(A) Mice carrying one *Pdgfrb*$^{+/J}$ allele but no allele for Cre-expression are, due to the STOP codon, *Pdgfrb* hemizygous. *Pdgfrb* hemizygous mice (*Pdgfrb*$^{+/-}$) develop normal kidneys (B). Shown are representative PAS stained sections of 10 weeks old mice.

(C-E) *Pdgfrb* hemizygous mice (*Pdgfrb*$^{+/-}$) underwent unilateral ureteral obstruction and were analyzed 5 days after induction. *Pdgfrb*$^{+/-}$ mice have same PDGFR-β protein levels as wt mice, as shown by western blot and its densitometric quantification (C) (n = 4 each group). Histomorphometric quantification of the fibrosis parameters α-SMA (D) and collagen I (E) do not show any significant differences in *Pdgfrb*$^{+/-}$ mice (n = 12) compared to wt mice (n = 8). Pictures show representative areas of respective staining.

Scale bars = 50 µm. Bar graphs show means ± SD. UUO= unilateral ureteral obstruction, ctrl = contralateral kidneys

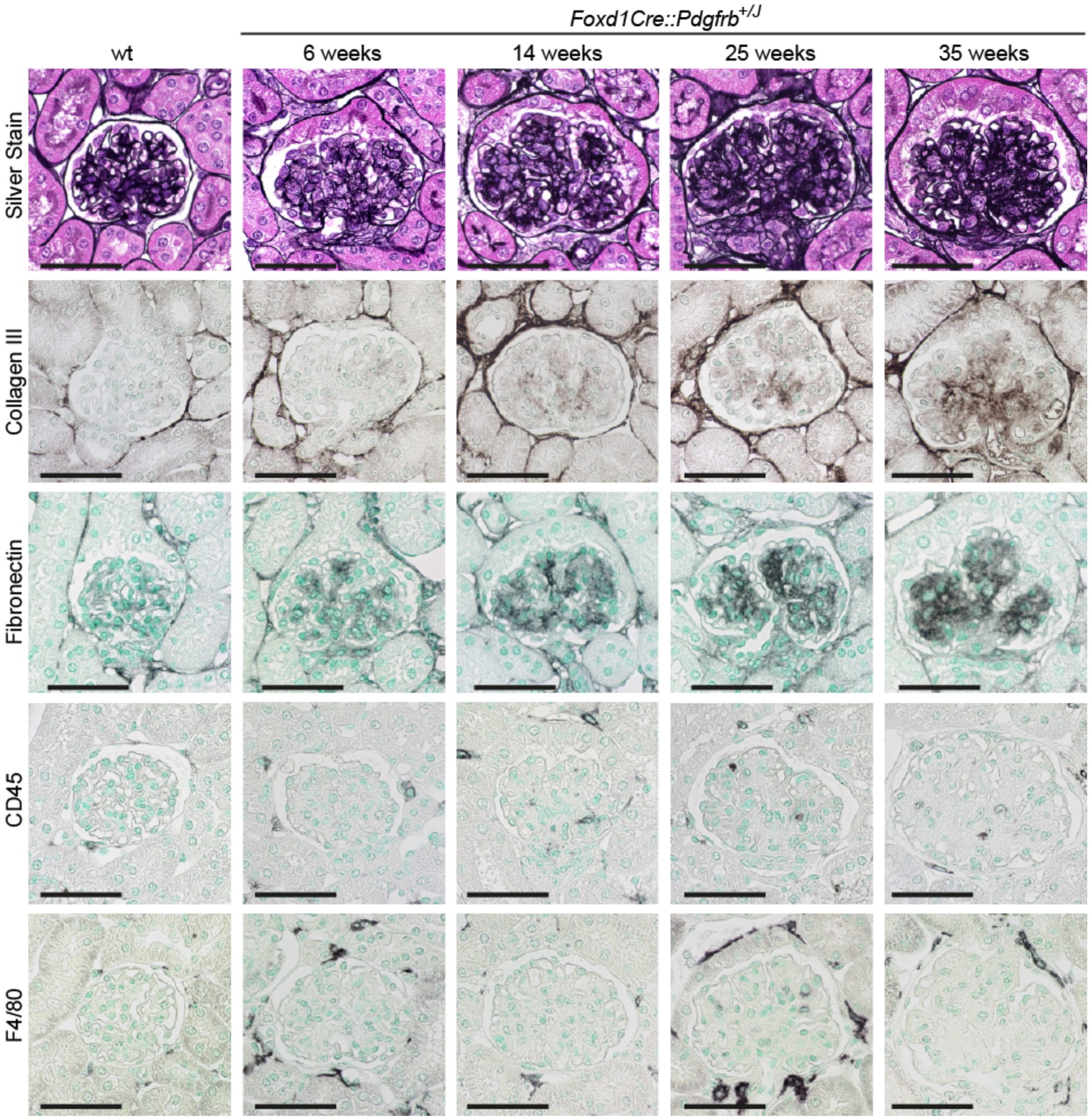

Appendix Figure S3: Activation of PDGFR-β in mesenchymal cells in *Foxd1Cre::Pdgfrb*$^{+/J}$ mice results in progressive proliferative glomerulonephritis and mesangial glomerulosclerosis

Histological stainings of glomeruli of wt kidneys (25 weeks) and *Foxd1Cre::Pdgfrb*$^{+/J}$ kidneys of 6, 14, 25 and 35 weeks old animals. Depicted are immunohistological stainings of fibronectin, collagen III, a silver stain to visualize the glomerular basement membrane, the pan-immune cell marker CD45 and the macrophage marker F4/80. Scale bar = 50 µm.

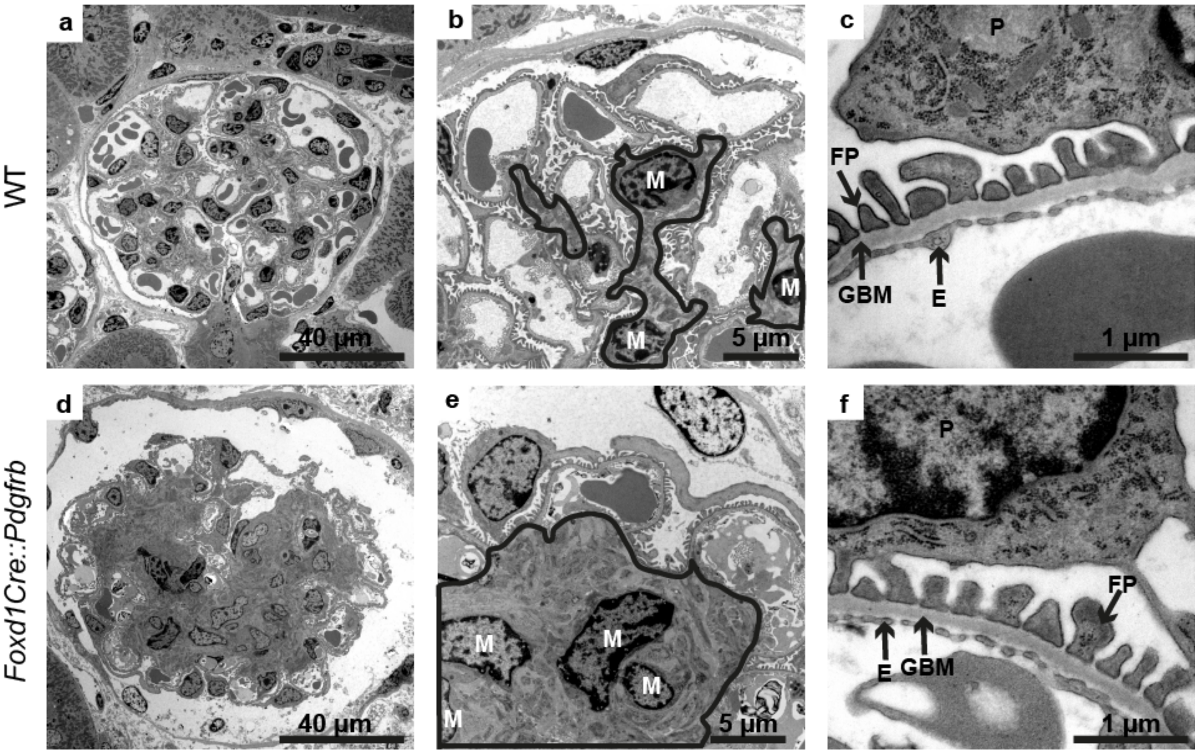

Appendix Figure S4: Transmission electron microscopy of wt and *Foxd1Cre::Pdgfrb*$^{+/J}$ mice

Transmission electron microscopy pictures of glomeruli of wt (a, b, c) and *Foxd1Cre::Pdgfrb*$^{+/J}$ mice (d, e, f) at 25 weeks. M= mesangial cell, P= podocyte, FP= foot processes, GBM= glomerular basement membrane, E= endothelial cell.

Expansion of the mesangium by mesangial cells and matrix was prominent in transgenic mice (b and e; the mesangial area is outlined). Interestingly, the *Foxd1Cre::Pdgfrb*$^{+/J}$ mice still have an intact filtration barrier with normal appearing podocytes forming regular foot processes and thin fenestrated endothelium.

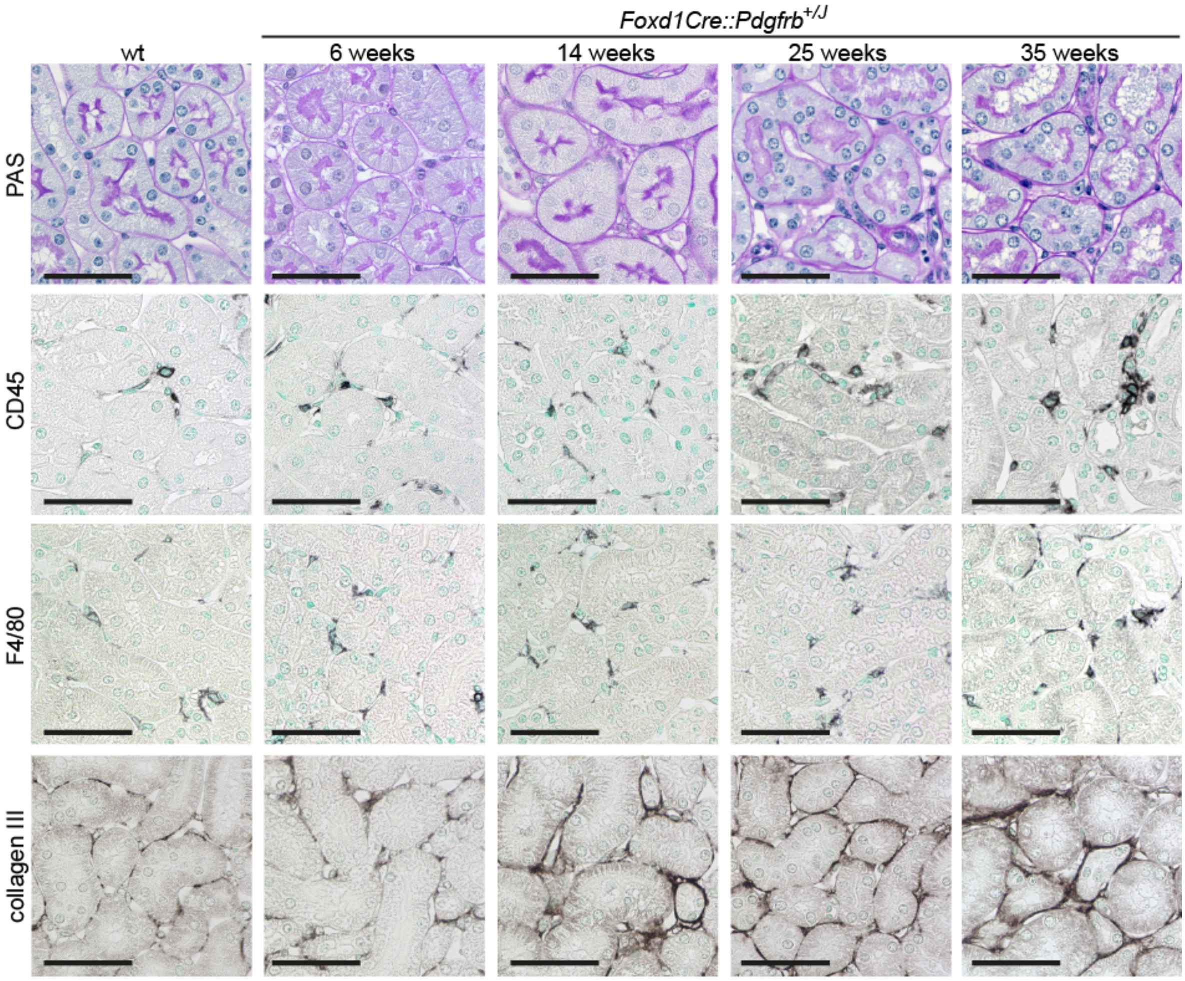

Appendix Figure S5: Interstitial changes in time course of *Foxd1Cre::Pdgfrb$^{+/J}$* mice

Histological stainings of the kidney cortex of wt kidneys (25 weeks) and *Foxd1Cre::Pdgfrb$^{+/J}$* kidneys of 6, 14, 25 and 35 weeks old animals. Depicted are immunohistological stainings of PAS, CD45, F4/80 and collagen III. Scale bar = 50 µm.

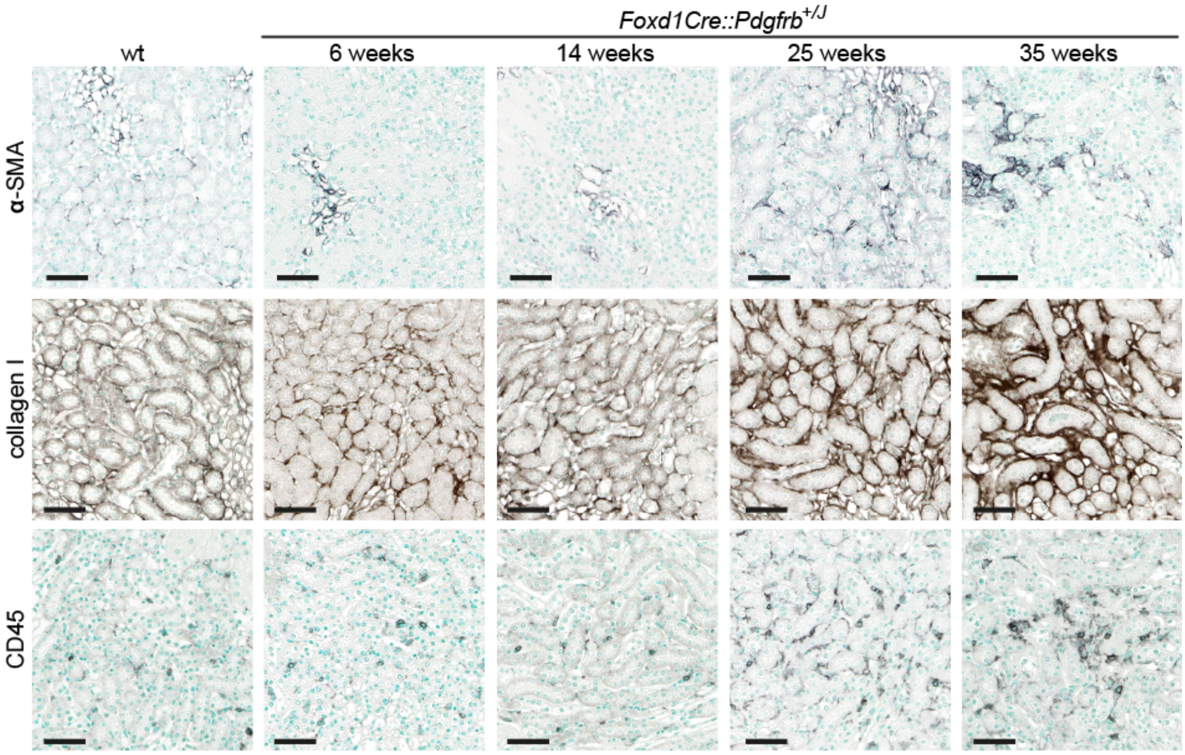

Appendix Figure S6: Interstitial changes in the medulla in time course of *Foxd1Cre::Pdgfrb$^{+/J}$* mice

Histological stainings of the kidney medulla of wt kidneys (25 weeks) and *Foxd1Cre::Pdgfrb$^{+/J}$* kidneys of 6, 14, 25 and 35 weeks old animals. Depicted are immunohistological stainings of α-SMA, collagen I and CD45. Scale bar = 50 µm.

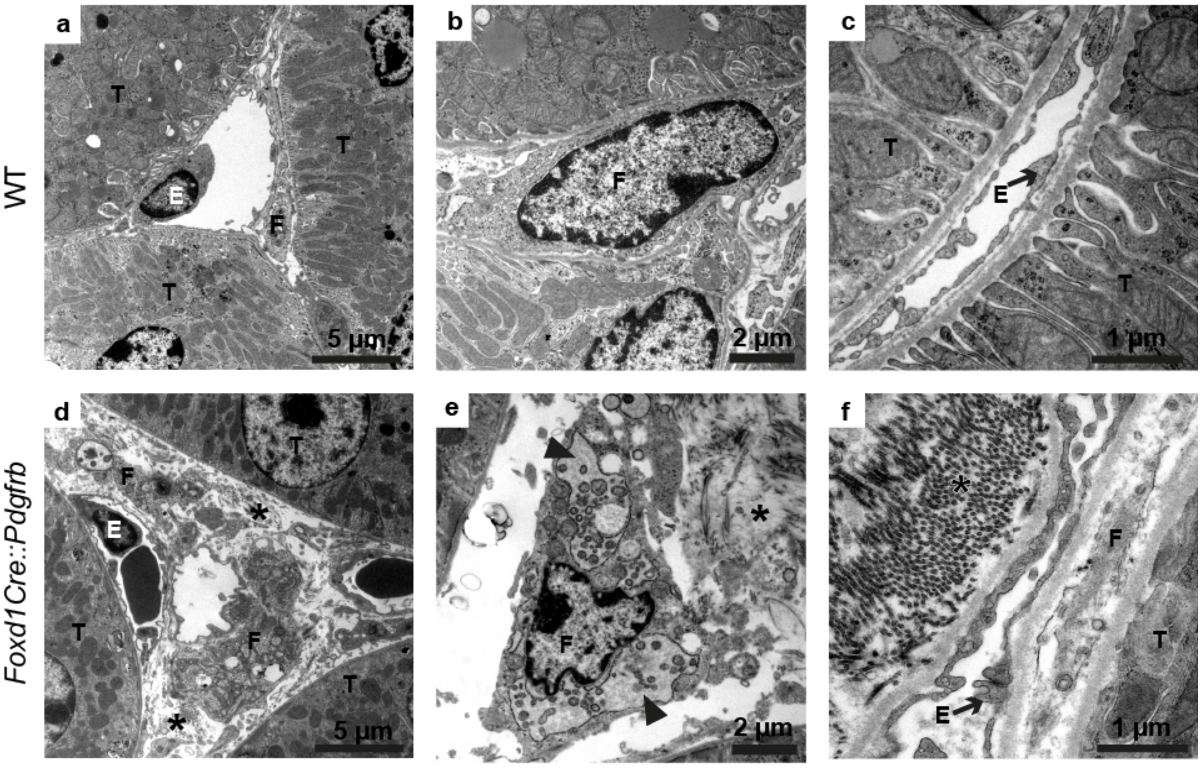

Appendix Figure S7: Transmission electron microscopy of the interstitium of wt and *Foxd1Cre::Pdgfrb*$^{+/J}$ mice

Transmission electron microscopy pictures of renal interstitium of wt (a, b, c) and *Foxd1Cre::Pdgfrb*$^{+/J}$ mice (d, e, f) at 25 weeks. T= tubuli, E= endothelium, F= fibroblast, *= ECM

(a, d) The interstitial space of *Foxd1Cre::Pdgfrb*$^{+/J}$ mice is widened and filled with matrix and collagen deposits. (b, e) The fibroblasts have dilated endoplasmatic reticulum (arrowheads). (c, f) The endothelium of interstitial capillaries showed pathological alterations with thickened cell soma and loss of fenestrations.

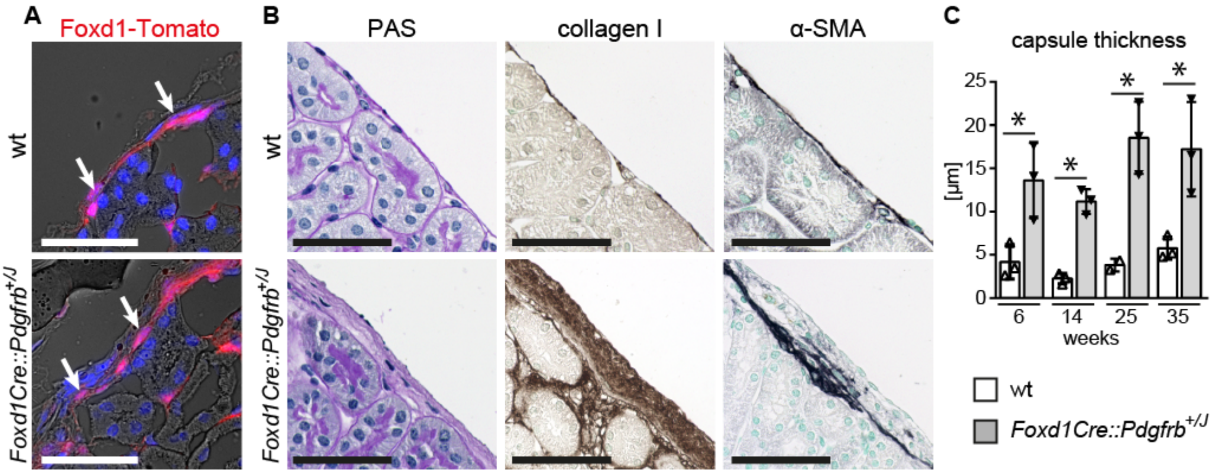

Appendix Figure S8: The renal capsule undergoes fibrous thickening in *Foxd1Cre::Pdgfrb*$^{+/J}$ mice

(A) FoxD1-reporter mice (*Foxd1Cre::tdTomato*) show FoxD1 positive cells directly under the capsule (white arrows).

(B) PAS staining and immunohistological staining of collagen I and α-SMA of the renal capsule show fibrous thickening of the capsule.

(C) The capsule was up to 3.8-fold thicker in the transgenic mice compared to wt. Bar graphs represent means ±SD of n = 3 animals.

*: $p \leq 0.05$ compared to same wt of same time point, scale bar = 50 µm

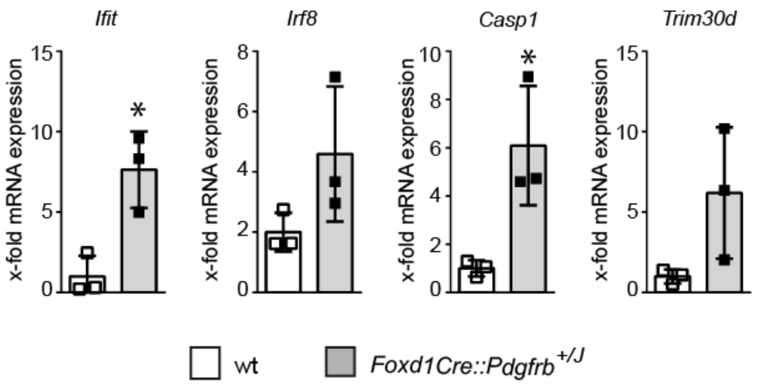

Appendix Figure S9: Quantitative real-time PCR confirm array data

Quantitative real-time PCR of *Ifit*, *Irf8*, *Casp1* and *Trim30d* show upregulated mRNA expression in 6 week old *Foxd1Cre::Pdgfrb*$^{+/J}$ mice compared to wt. Bar graphs represent means ±SD of n = 3 animals, *: p≤0.05

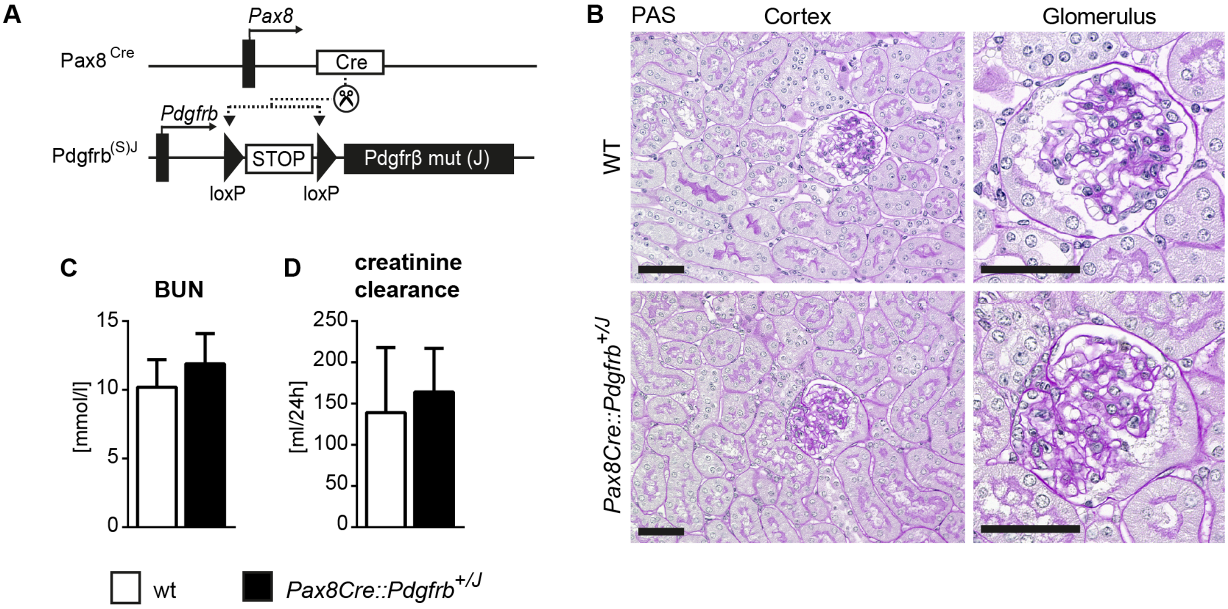

Appendix Figure S10: Tubular cell-specific activation of PDGFR-β signaling had no effects

(A) Activation of PDGFR-β in renal tubular cells, denoted here as *Pax8Cre::Pdgfrb*$^{+/J}$ mice, was achieved by cross-breeding the *Pax8-Cre* mouse line (Pax8^{Cre}) with a mouse line with a heterozygous knock-in with constitutively active *Pdgfrb* mutant (J) allele (Pdgfrb$^{(S)J}$) instead of the wt *Pdgfrb* allele.

(B) PAS sections of 24 weeks old *Pax8Cre::Pdgfrb*$^{+/J}$ and wt mice showed normal morphological findings. Scale bar = 50 µm

(C) *Pax8Cre::Pdgfrb*$^{+/J}$ mice have similar BUN concentrations and creatinine clearance as wt littermates. Data represent means ± SD of *Pax8Cre::Pdgfrb*$^{+/J}$ n = 6 and wt n = 9
