# Supplementary material for: Dysregulated mesenchymal PDGFR‐β drives kidney fibrosis
Source: EMBO Mol Med. 2020 Jan 14;12(3):e11021. doi: 10.15252/emmm.201911021 (PMC7059015; doi:10.15252/emmm.201911021)
Supplement: Supplementary file 2 — Expanded View Figures PDF [file EMMM-12-e11021-s002.pdf]

Expanded View Figures

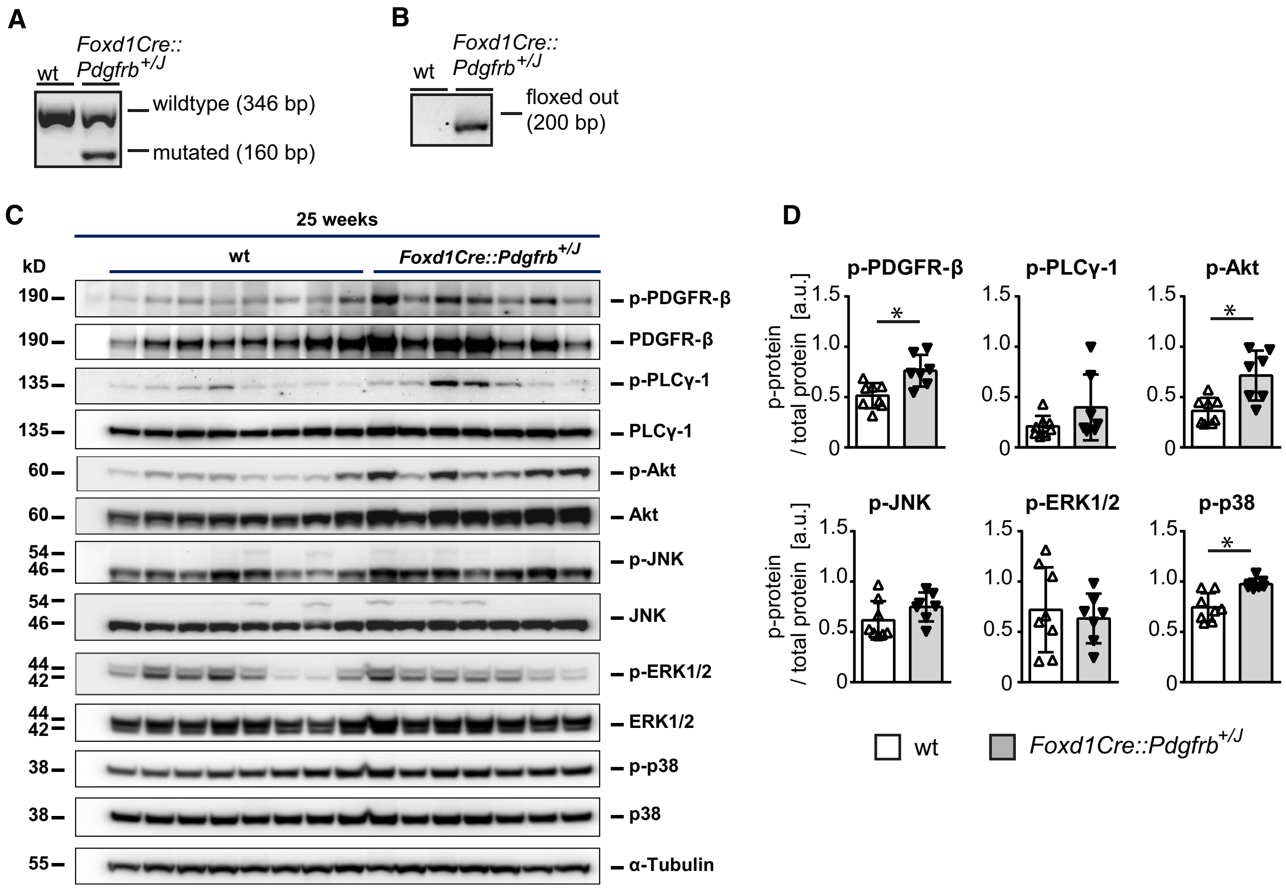

Figure EV1. Constitutive PDGFR-β activation in renal mesenchyme in transgenic *Foxd1Cre::Pdgfrb$^{+/J}$* mice.

A Genotyping PCR in *Foxd1Cre::Pdgfrb$^{+/J}$* mice shows a wild-type band at 346 bp and the mutated *Pdgfrb p.V536A (J)* band with at 160 bp.

B *Foxd1*-driven Cre recombinase activity is proven by PCR detection with primers detecting the *Pdgfrb p.V546A* allele with cutout STOP sequence. The corresponding band has a size of 200 bp.

C, D (C) Western blots of PDGFR-β and its downstream signaling molecules in 25-week-old wt and *Foxd1Cre::Pdgfrb$^{+/J}$* mice and their respective densitometric evaluations (D) show regulations on the level of total protein and on phosphorylation status for PDGFR-β and downstream molecules Akt and p38. Phospho-levels are normalized to the respective unphosphorylated protein forms. kD = kilodalton. Bar graphs represent means ± SD; wt $n = 8$, *Foxd1Cre::Pdgfrb$^{+/J}$* $n = 7$. Statistical analysis was performed by unpaired two-tailed Student's *t*-test. $^{*}P < 0.05$. Exact *P*-values are provided in Appendix Table S4.

© 2020 The Authors

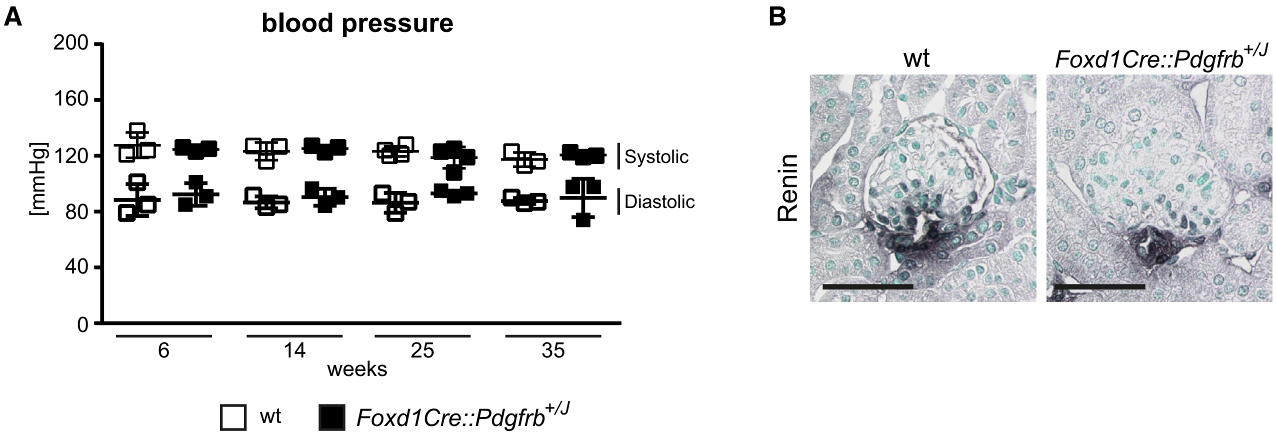

Figure EV2. Blood pressure and renin production are not changed by mesenchymal PDGFR-β activation.

A The blood pressure of *Foxd1Cre::Pdgfrb*$^{+/J}$ and wt mice remains normal during the whole time course. Data represent means ± SD of n = 3 animals.

B The population of renin-expressing cells is also similar between the groups, as shown using the renin staining. Scale bar = 50 μm.

 © 2020 The Authors

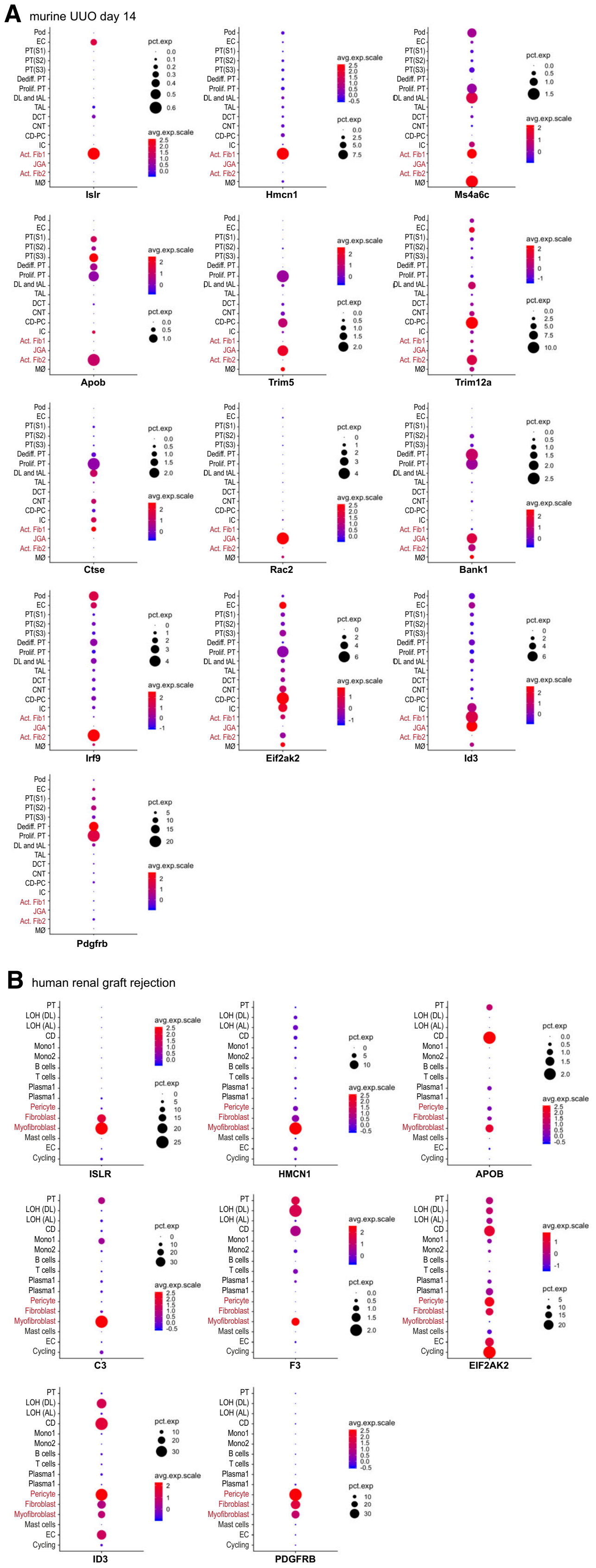

Figure EV3. Single-cell RNA sequome in fibrotic murine and human kidney shows mesenchymal allocation of genes regulated in *Foxd1Cre::Pdgfrb*$^{+/J}$ mice.

A Genes regulated in our array in *Foxd1Cre::Pdgfrb*$^{+/J}$ mice were reanalyzed in a single-cell RNA sequome data set of a fibrotic murine kidney subjected to UUO for 14 days. Shown are the genes that could be allocated to mesenchymal cell populations (activated fibroblasts = Act Fib1 and Act Fib2; or juxtaglomerular cells/renin cells = JGA). Pod = podocytes; EC = endothelial cells; PT(S1-3) = proximal tubules; Dediff. PT = dedifferentiated proximal tubular cells; Prolif. PT = proliferating proximal tubular cells. DL and tAL = descending loop of henle and thin ascending loop; TAL = thick ascending loop; DCT = distal convoluted tubules; CNT = connecting tubules; CD-PC = principal cells of collecting duct; IC = intercalated cells of collecting duct; Mø = macrophages.

B The same expression comparison was done in a data set of a human renal graft rejected kidney. LOH (DL) = descending loop of henle; LOH (AL) = ascending loop of henle; CD = collecting duct; Mono1 and 2 = monocytes.

© 2020 The Authors

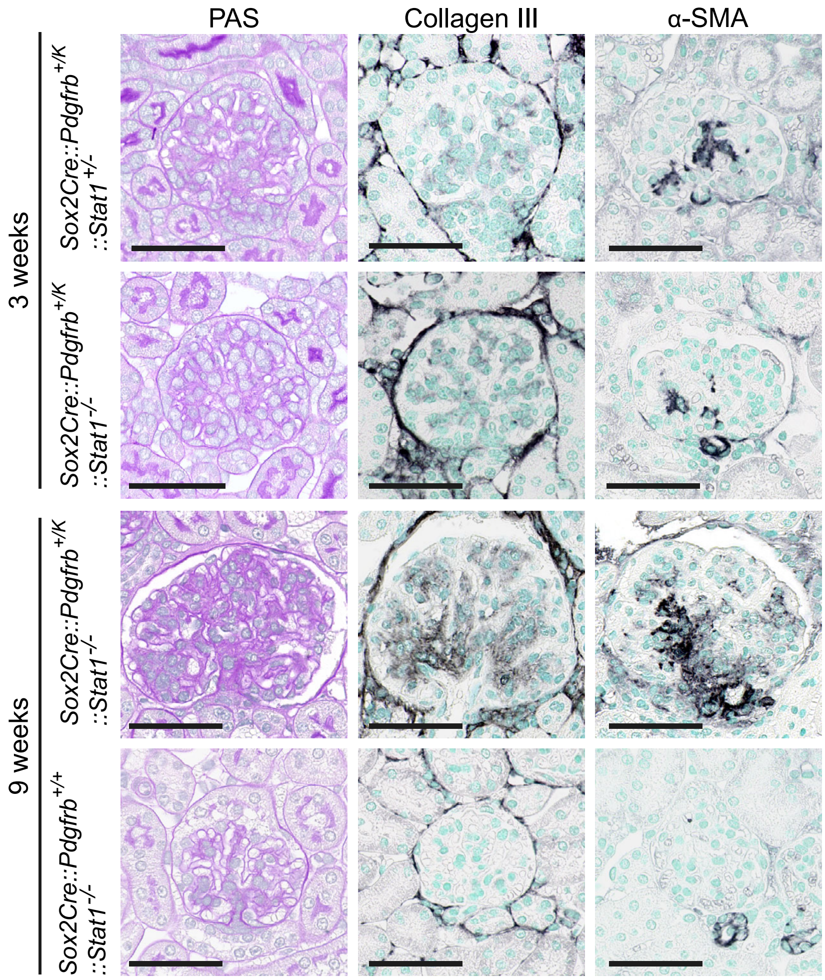

Figure EV4. Glomerular pathology in *Sox2Cre::Pdgfrb*$^{+/K}$ mice resembled the findings in *Foxd1Cre::Pdgfrb*$^{+/J}$ but suggested no role for STAT1.

Glomeruli stained for PAS and immunohistological stainings for collagen III and α-SMA of 3-week-old *Sox2Cre::Pdgfrb*$^{+/K}$ mice with intact STAT1 signaling (*Stat1*$^{+/-}$) and with deletion of STAT1 signaling (Stat1$^{-/-}$). At this very early stage, hardly any sclerosis was found on PAS or collagen III staining; however, the mesangial expression of α-SMA was reduced in mice lacking STAT1. *Sox2Cre::Pdgfrb*$^{+/K}$ mice with intact STAT1 signaling (*Stat1*$^{+/-}$) were not viable at 9 weeks and could not be analyzed. However, the *Sox2Cre::Pdgfrb*$^{+/K}$ mice lacking STAT1 signaling survived up till later age and showed a progression of mesangioproliferative glomerulonephritis and also mesangial sclerosis, resembling closely the findings in *Foxd1Cre::Pdgfrb*$^{+/J}$. Mice without activating *Pdgfrb* mutation and lacking STAT1 (*Sox2Cre::Pdgfrb*$^{+/+}$*::Stat1*$^{-/-}$) do not show any pathological phenotype. Pictures are chosen as representatives from $n = 3$ each group. Scale bar = 50 μm.

Source data are available online for this figure.

© 2020 The Authors

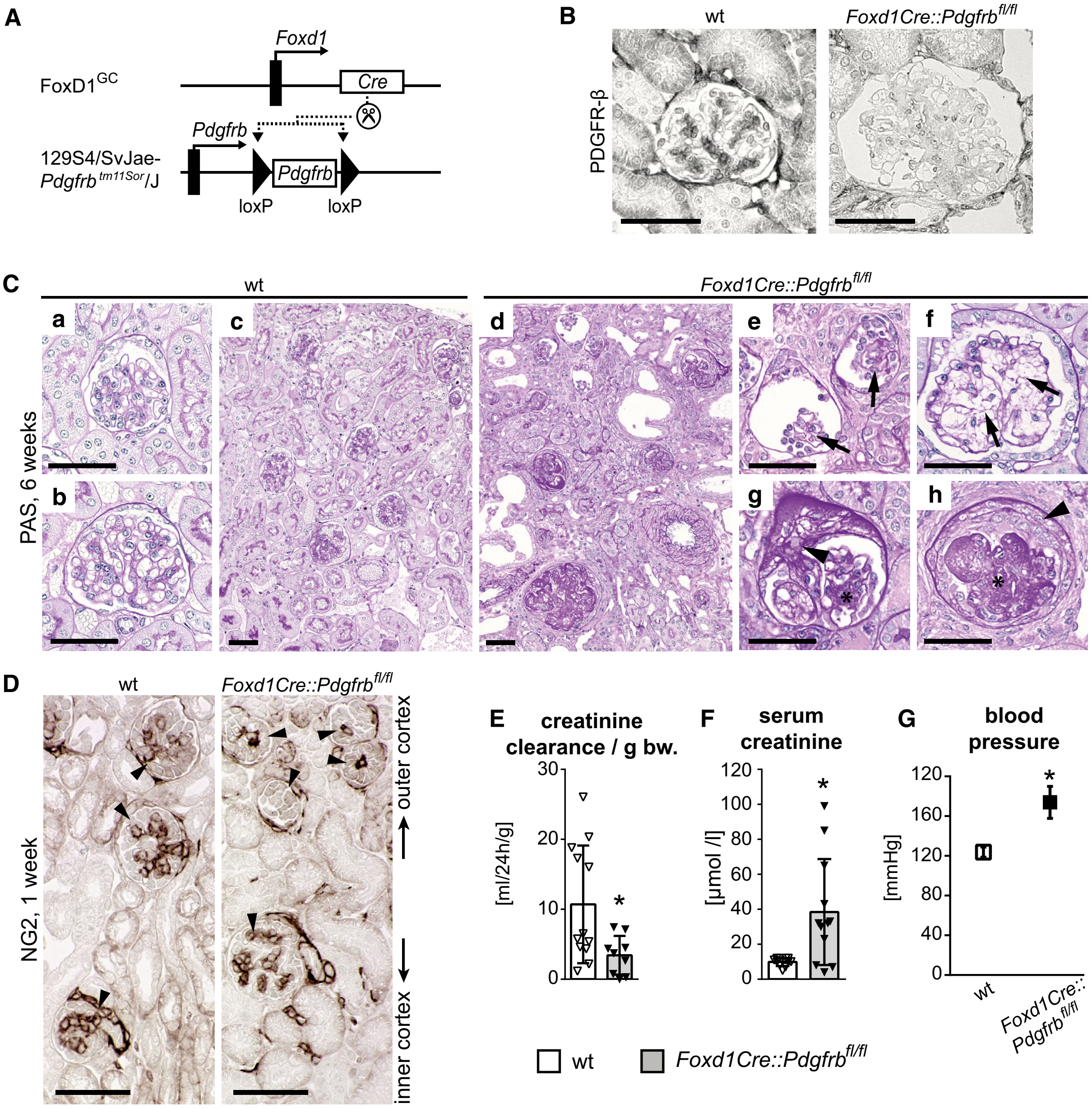

Figure EV5. Developmental knockout of *Pdgfrb* in renal mesenchyme leads to defective glomerulogenesis due to lack of mesangium and resulting pathological frailty of glomeruli.

A Knockout of *Pdgfrb* in renal mesenchyme was achieved by crossbreeding the *Foxd1-Cre* mouse line (FoxD1^{GC}) with a mouse line with a homozygous floxed *Pdgfrb* gene (129S4/SvJae-Pdgfrb^{tm11Sor}/J), resulting in *Foxd1Cre::Pdgfrb*$^{fl/fl}$ mice.

B Immunohistochemical staining for PDGFR-β shows positivity in mesangial cells of wild-type (wt) mice but not of *Foxd1Cre::Pdgfrb*$^{fl/fl}$ mice.

C Six-week-old wt mice (a–c) have normal developed kidney cortex with fully developed glomeruli in outer cortex (a) as well as in the inner cortex (b). *Foxd1Cre::Pdgfrb*$^{fl/fl}$ mice (d–h) exhibit severe pathological changes in the kidney with aberrant glomeruli, dilated tubules, and interstitial fibrosis. The glomeruli of the outer cortex, i.e., the younger glomeruli (e, f), lack mesangium (arrows point to expected mesangial areas), whereas the ones of the inner cortex, i.e., the older glomeruli, show focal (g) or global (h) sclerosis (*) and also segmental rupture of capillaries with extracellular proliferates (crescents) (arrowheads).

D Histological staining of mesangial cell marker NG2 in 1-week-old mice shows that in *Foxd1Cre::Pdgfrb*$^{fl/fl}$ mice, only in the glomeruli of the inner cortex a mesangial tree developed, whereas it is lacking in the glomeruli of the outer cortex, leading to undeveloped glomeruli. Arrowheads point to positive stained mesangial cells.

E, F (E) Decreased creatinine clearance per gram bodyweight and (F) elevated serum creatinine levels in *Foxd1Cre::Pdgfrb*$^{fl/fl}$ mice reflected renal insufficiency in these mice.

G Kidney dysfunction in *Foxd1Cre::Pdgfrb*$^{fl/fl}$ mice results in hypertension.

Data information: Bar graphs show mean ± SD; wt $n = 11$, *Foxd1Cre::Pdgfrb*$^{fl/fl}$ $n = 9$. Statistical analysis was performed by unpaired two-tailed Student's *t*-test. *$P < 0.05$. Exact *P*-values are provided in Appendix Table S4.
Source data are available online for this figure.
