# Supplementary material for: Dysregulated mesenchymal PDGFR‐β drives kidney fibrosis
Source: EMBO Mol Med. 2020 Jan 14;12(3):e11021. doi: 10.15252/emmm.201911021 (PMC7059015; doi:10.15252/emmm.201911021)
Supplement: Supplementary file 4 — Movie EV2 [file EMMM-12-e11021-s004.zip › EMM-2019-11021_Movie_EV2/Legend_Movie_2.docx]

**Appendix Movie 2: GFP-PDGFR-β reporter in fibrotic murine kidney**

Optical tissue clearing and 3D-reconstruction in *Pdgfrb-GFP* reporter mice shows that PDGFR-β (green) expression is up-regulated during fibrosis in the UUO model.
