# Supplementary figures and images for: Dysregulated mesenchymal PDGFR‐β drives kidney fibrosis
Source: EMBO Mol Med. 2020 Jan 14;12(3):e11021. doi: 10.15252/emmm.201911021 (PMC7059015; doi:10.15252/emmm.201911021)

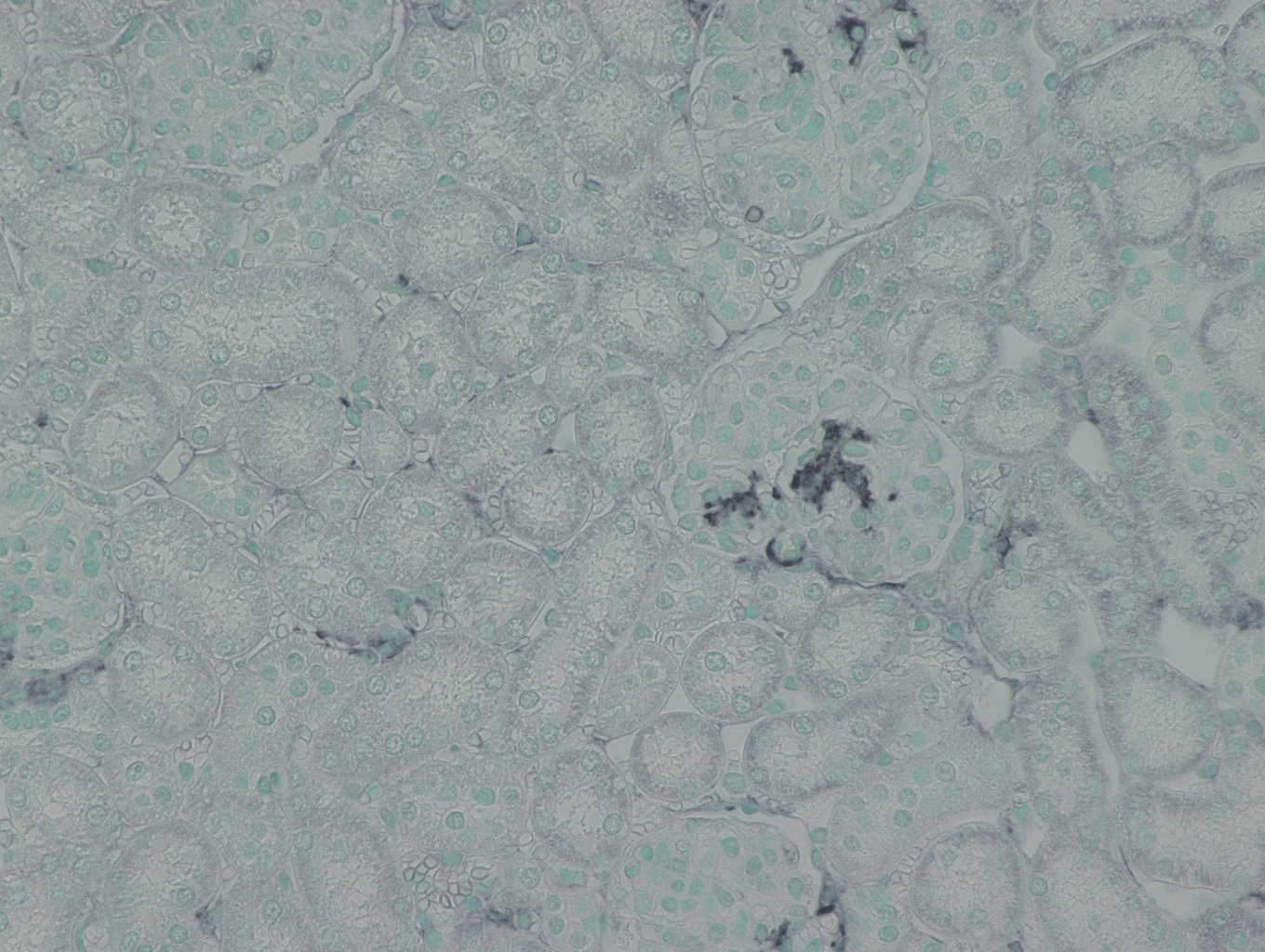

Supplement: Supplementary file 5 — Source Data for Expanded View [file EMMM-12-e11021-s012.zip › EV_source_data/SourceData_FigEV4/FigEV4_3weeks_asma_STAT+-.TIF]

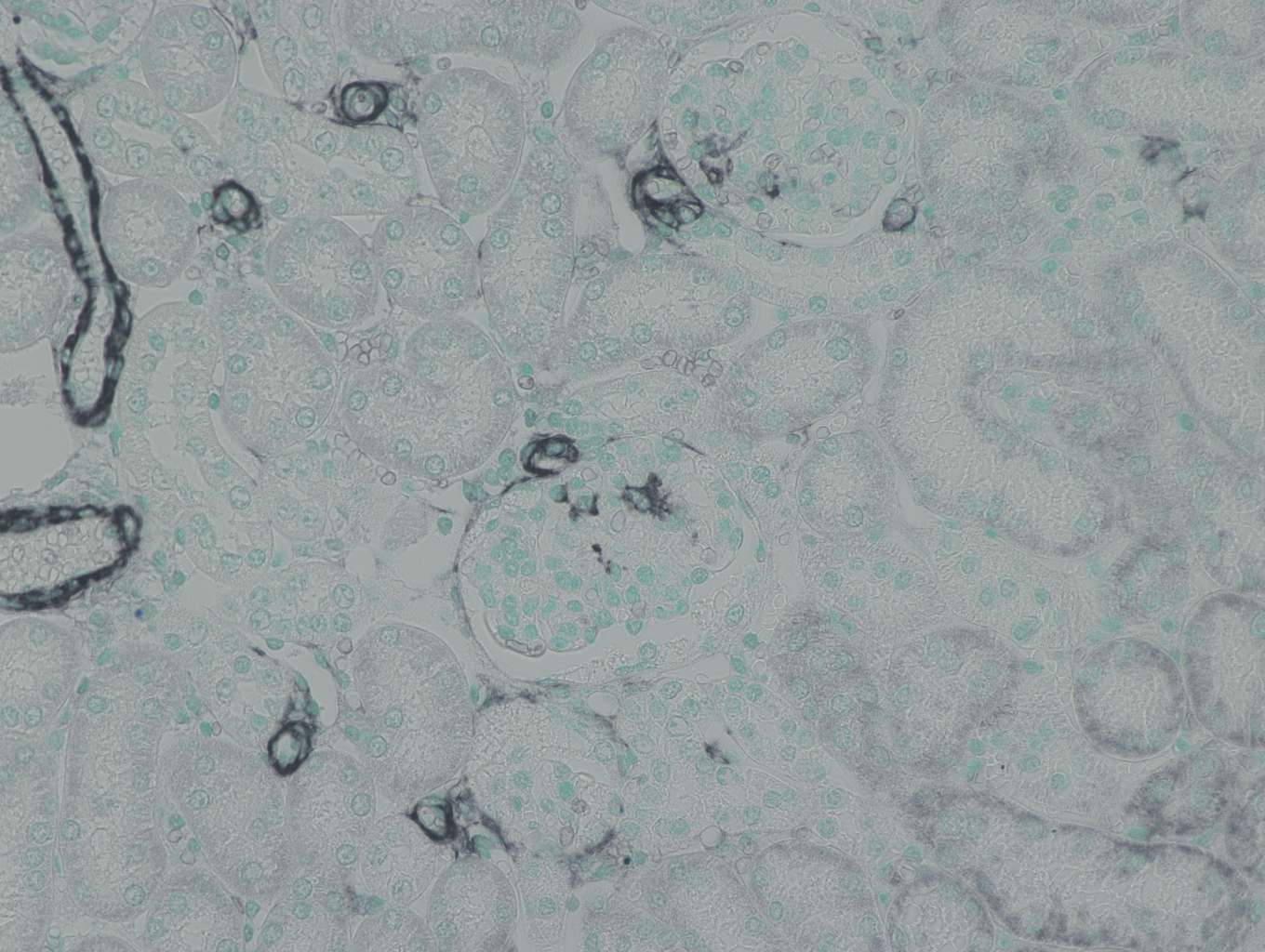

Supplement: Supplementary file 5 — Source Data for Expanded View [file EMMM-12-e11021-s012.zip › EV_source_data/SourceData_FigEV4/FigEV4_3weeks_asma_STAT--.TIF]

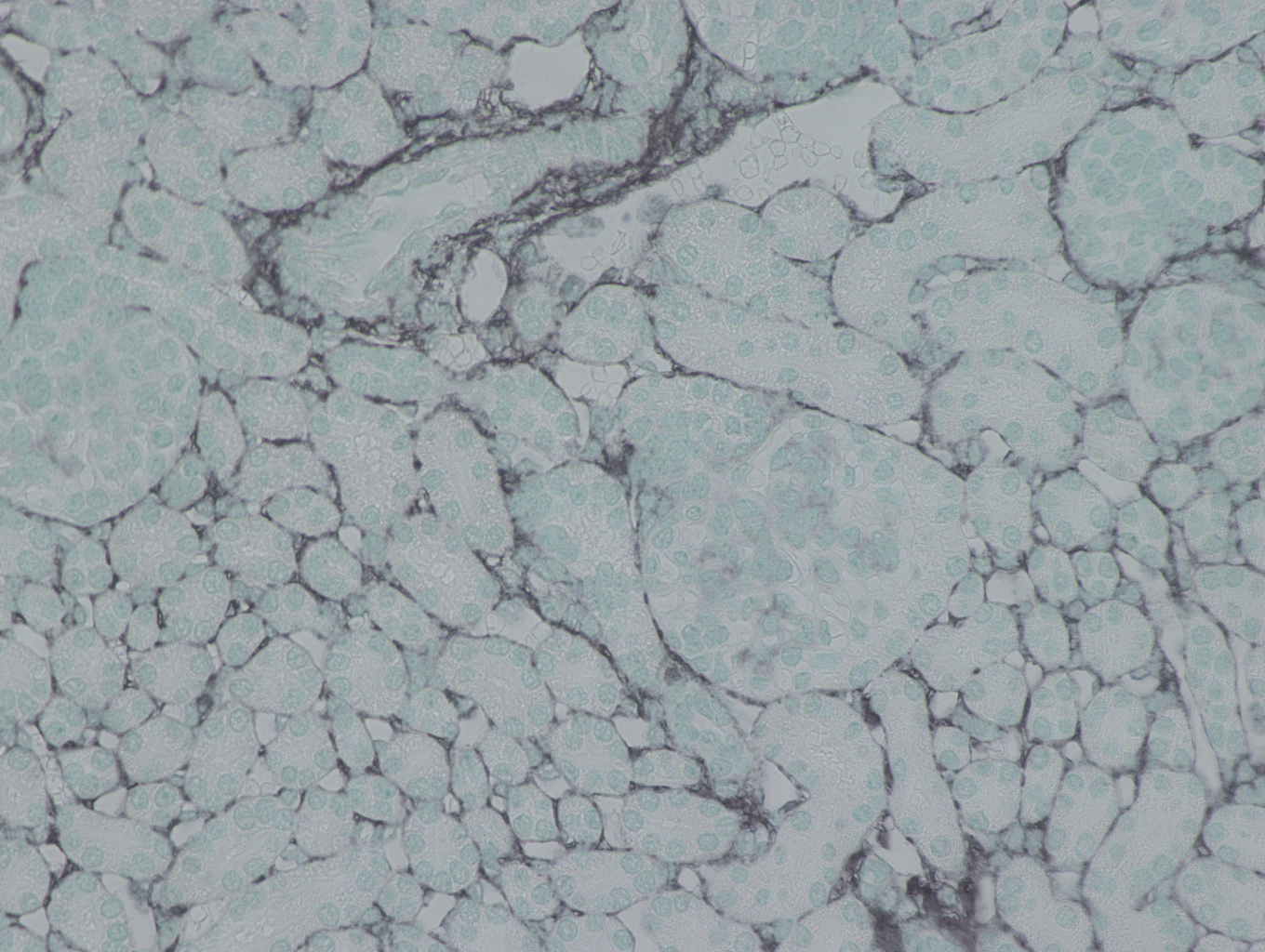

Supplement: Supplementary file 5 — Source Data for Expanded View [file EMMM-12-e11021-s012.zip › EV_source_data/SourceData_FigEV4/FigEV4_3weeks_Col3_STAT+-.TIF]

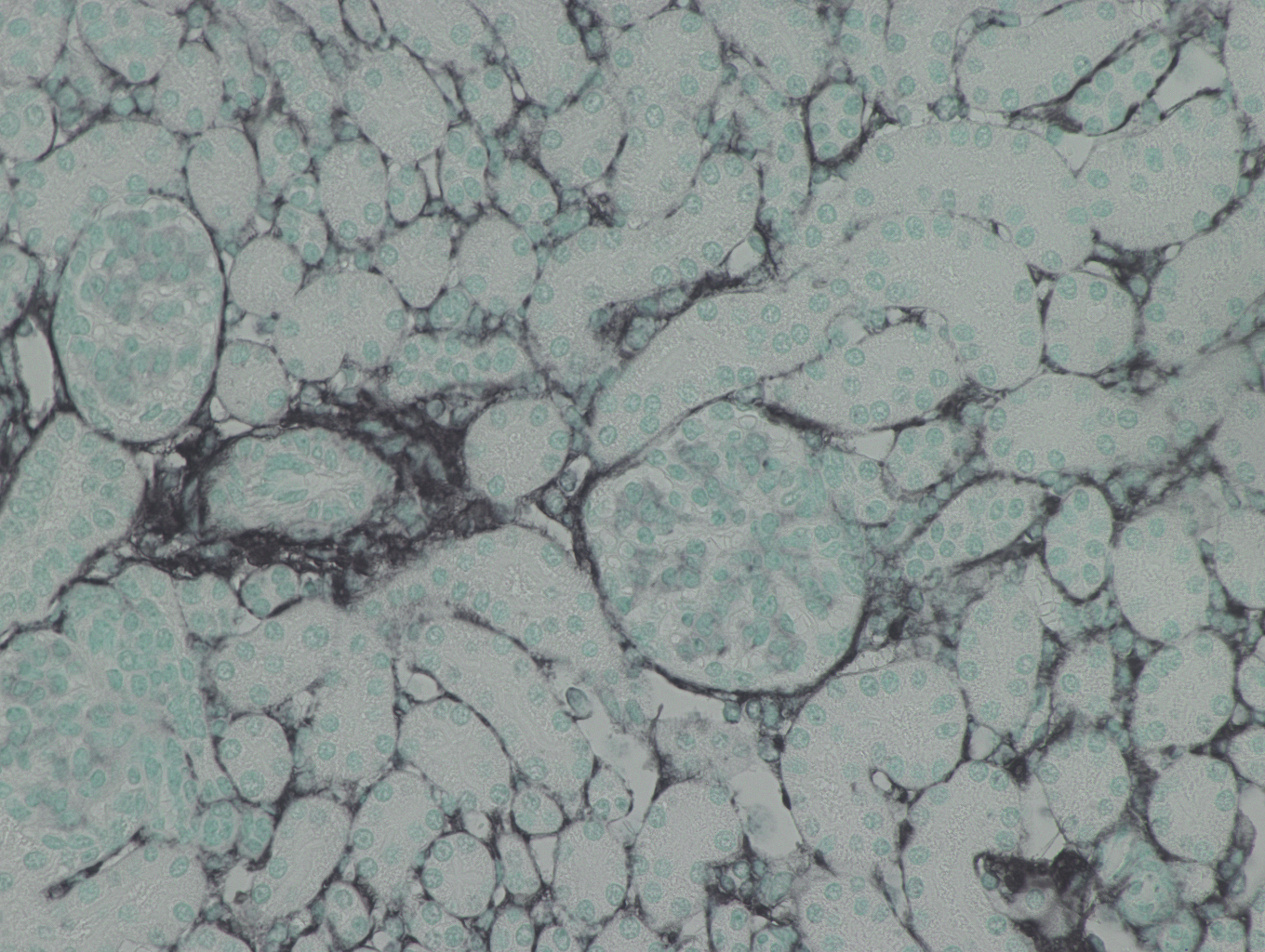

Supplement: Supplementary file 5 — Source Data for Expanded View [file EMMM-12-e11021-s012.zip › EV_source_data/SourceData_FigEV4/FigEV4_3weeks_Col3_STAT--.TIF]

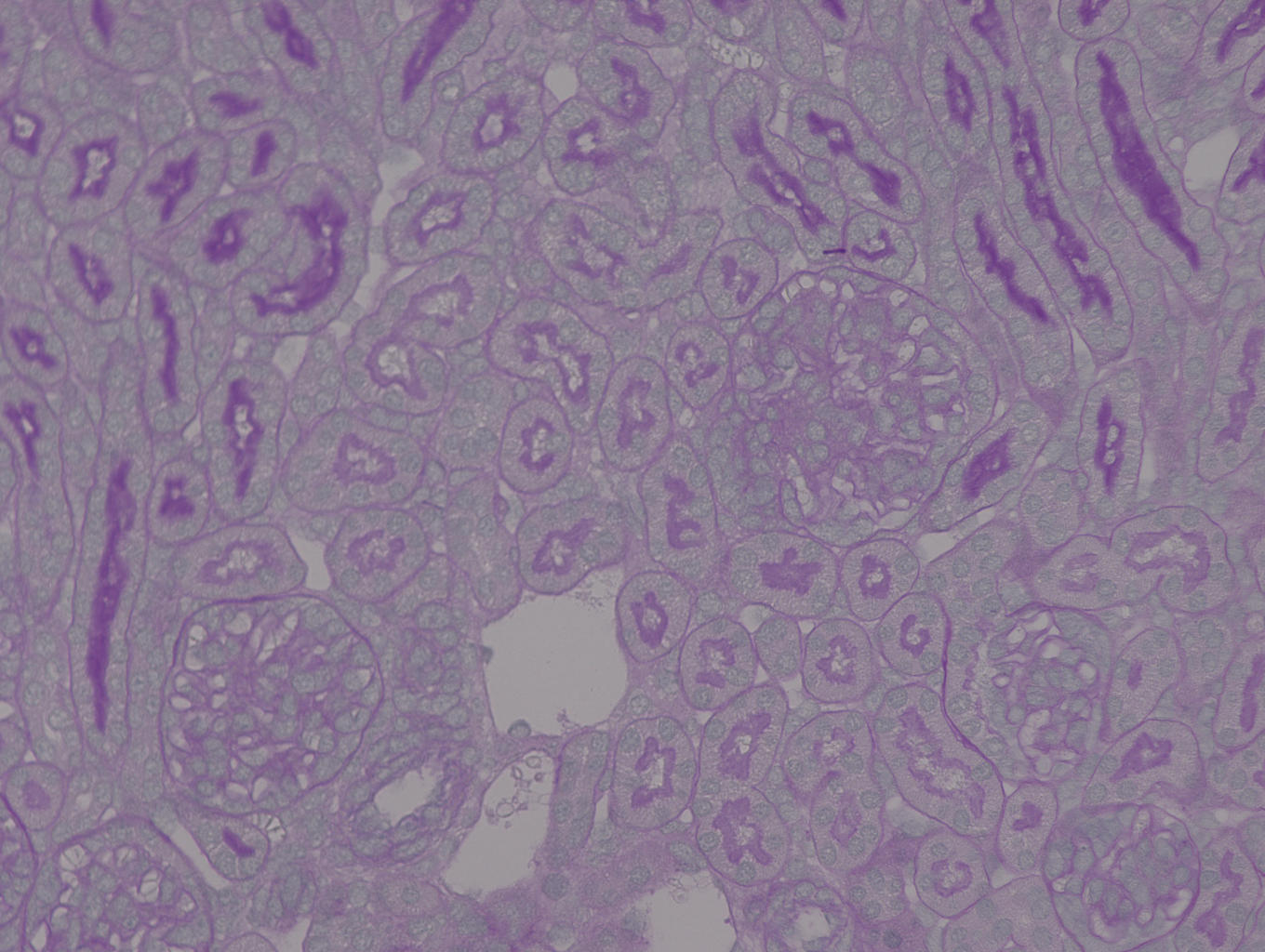

Supplement: Supplementary file 5 — Source Data for Expanded View [file EMMM-12-e11021-s012.zip › EV_source_data/SourceData_FigEV4/FigEV4_3weeks_PAS_STAT+-.TIF]

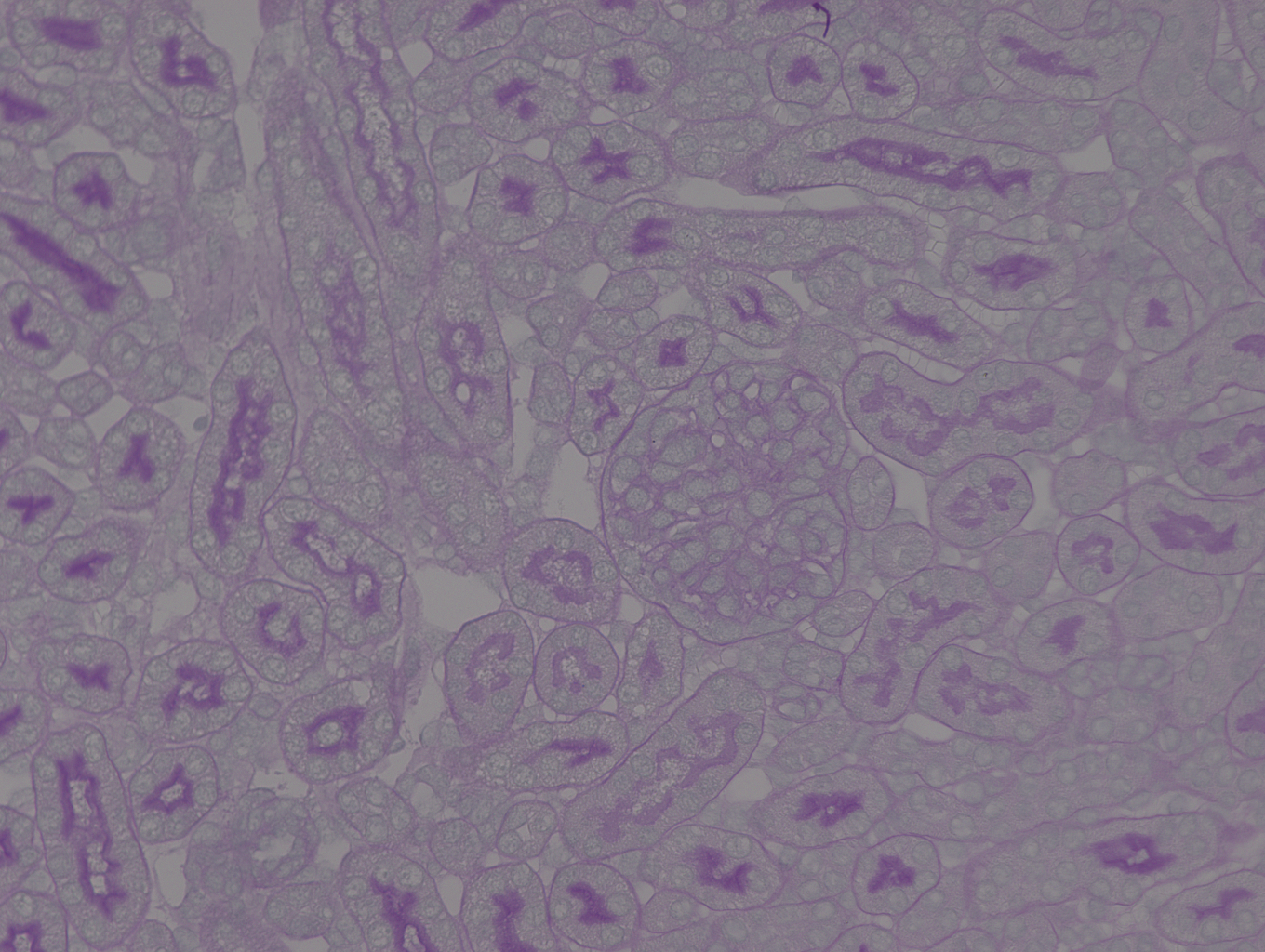

Supplement: Supplementary file 5 — Source Data for Expanded View [file EMMM-12-e11021-s012.zip › EV_source_data/SourceData_FigEV4/FigEV4_3weeks_PAS_STAT--.TIF]

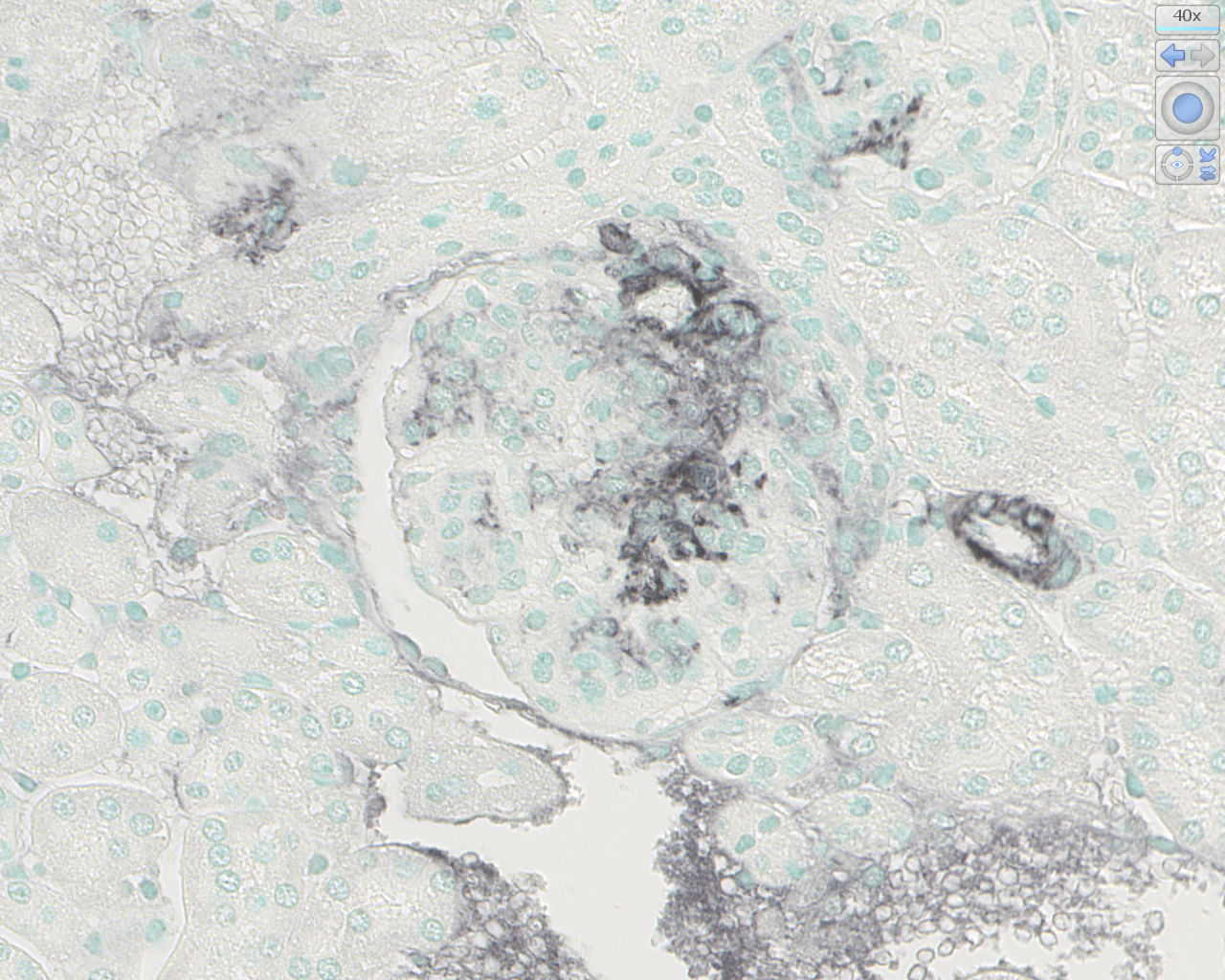

Supplement: Supplementary file 5 — Source Data for Expanded View [file EMMM-12-e11021-s012.zip › EV_source_data/SourceData_FigEV4/FigEV4_9weeks_asma_STAT--.jpg]

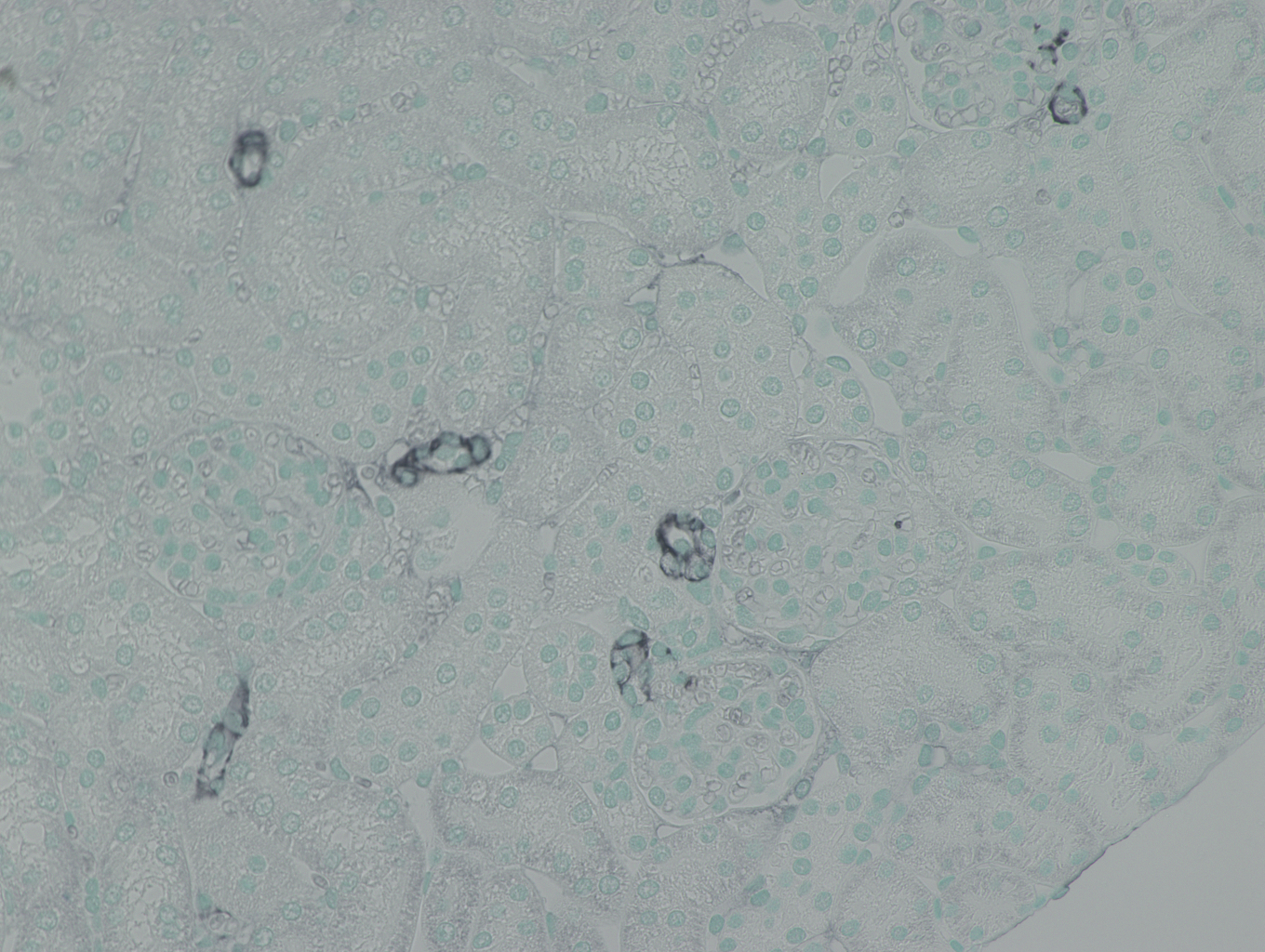

Supplement: Supplementary file 5 — Source Data for Expanded View [file EMMM-12-e11021-s012.zip › EV_source_data/SourceData_FigEV4/FigEV4_9weeks_asma_wt_STAT--.TIF]

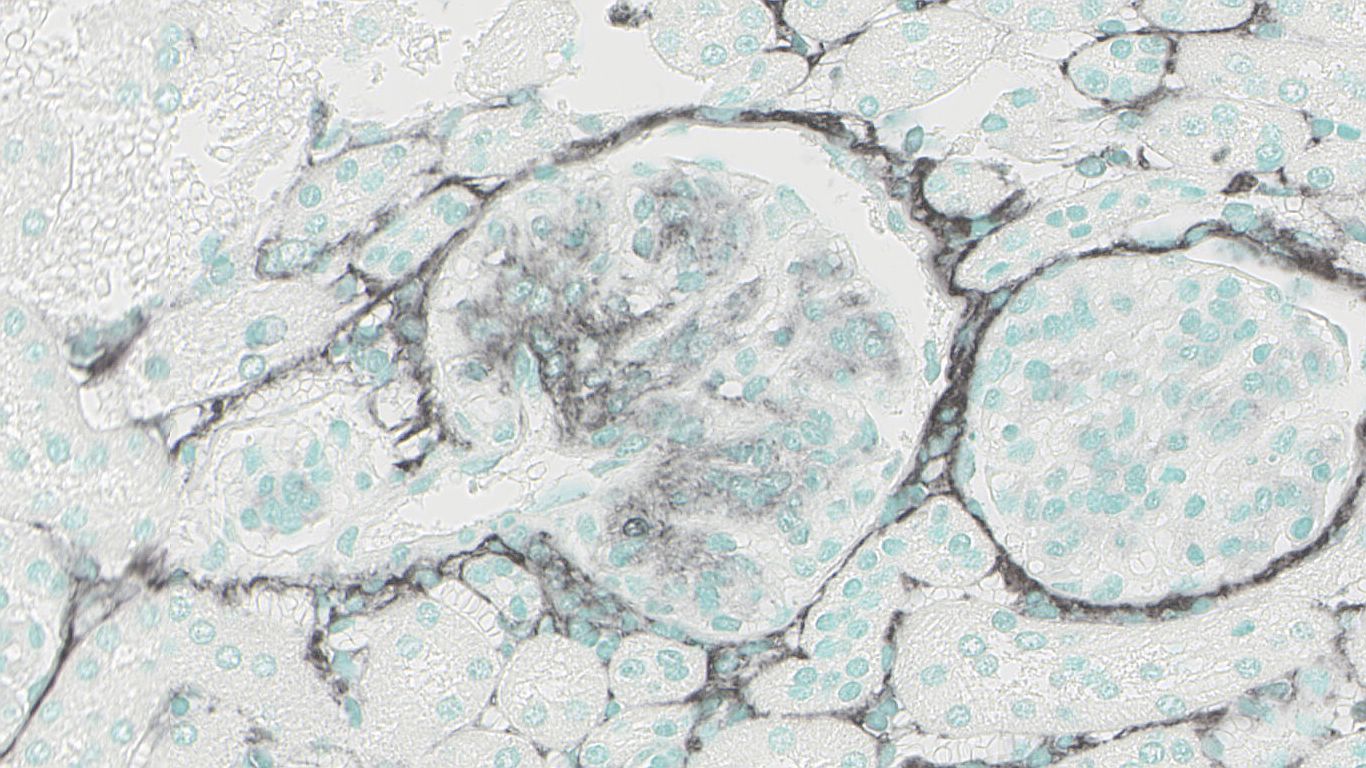

Supplement: Supplementary file 5 — Source Data for Expanded View [file EMMM-12-e11021-s012.zip › EV_source_data/SourceData_FigEV4/FigEV4_9weeks_Col3_STAT--.jpg]

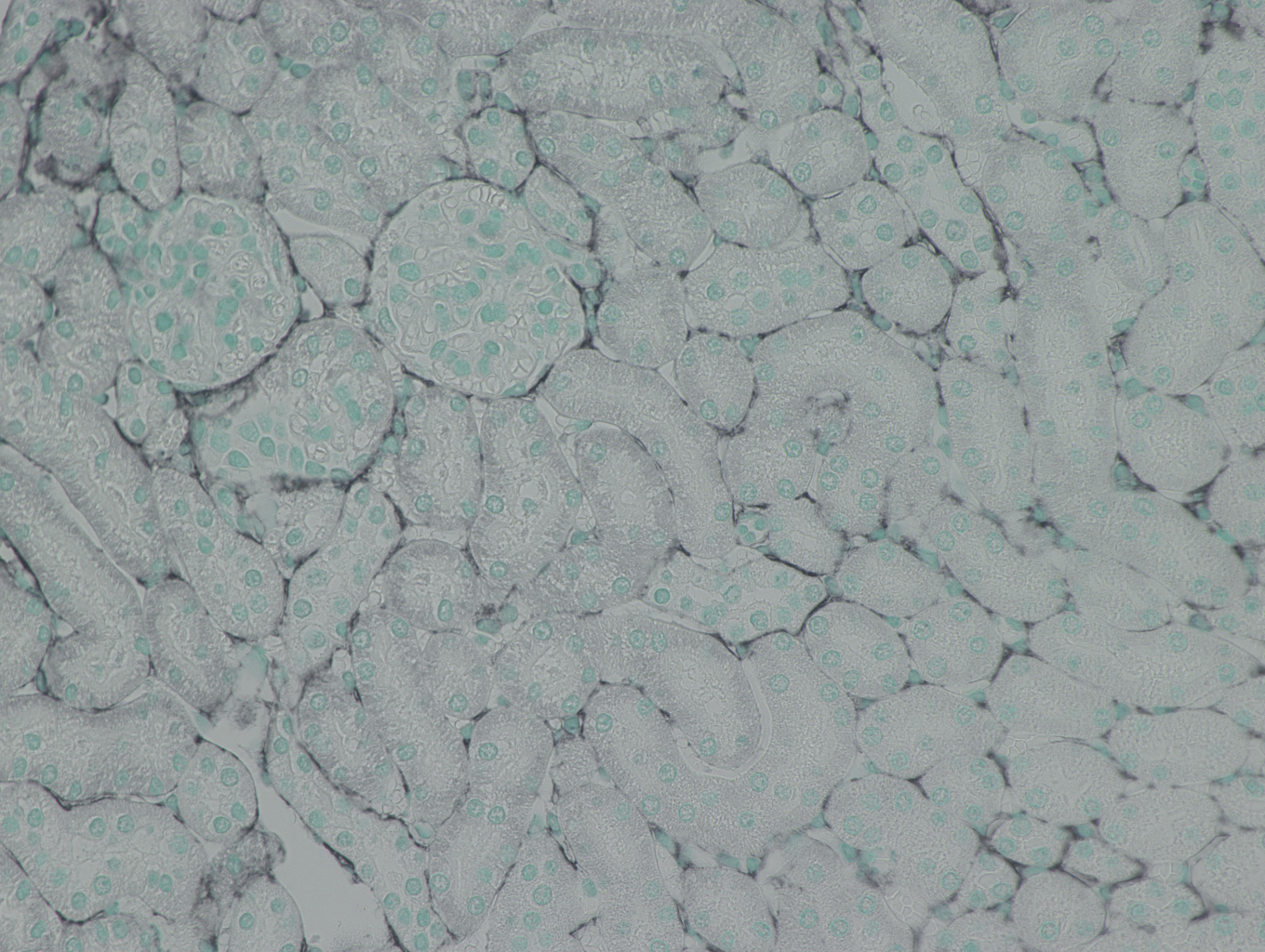

Supplement: Supplementary file 5 — Source Data for Expanded View [file EMMM-12-e11021-s012.zip › EV_source_data/SourceData_FigEV4/FigEV4_9weeks_Col3_wt_STAT--.TIF]

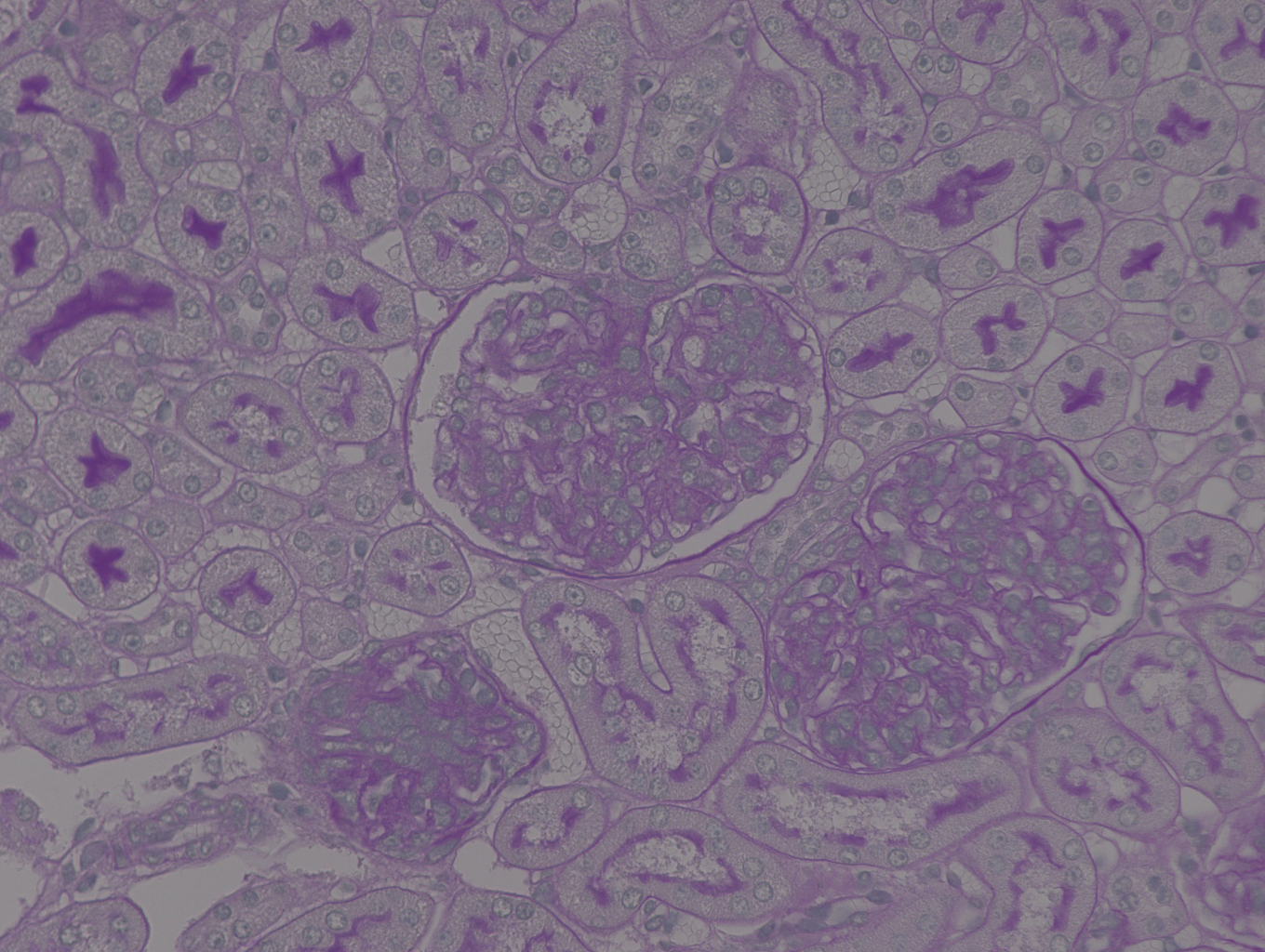

Supplement: Supplementary file 5 — Source Data for Expanded View [file EMMM-12-e11021-s012.zip › EV_source_data/SourceData_FigEV4/FigEV4_9weeks_PAS_mutant_STAT--.TIF]

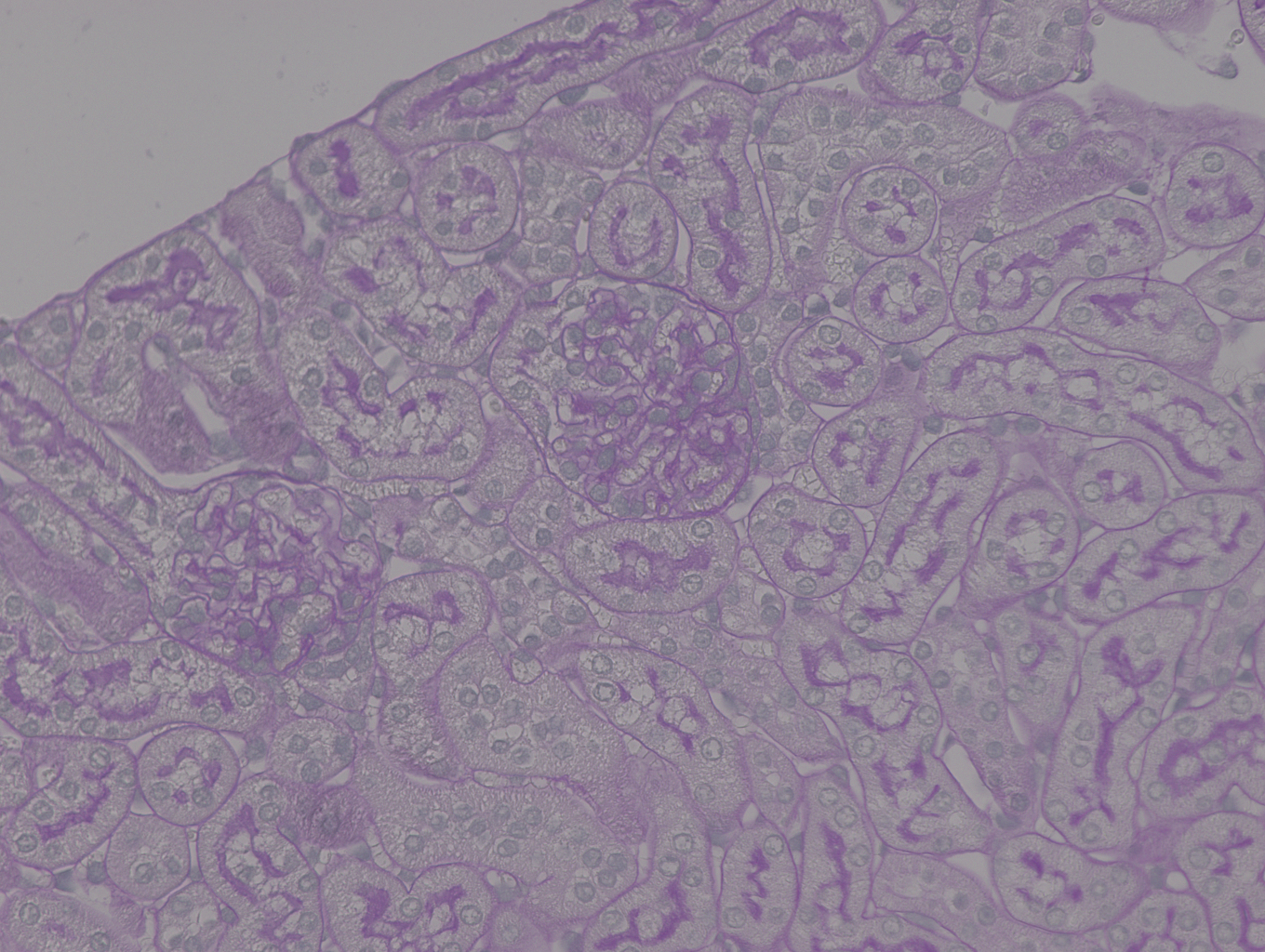

Supplement: Supplementary file 5 — Source Data for Expanded View [file EMMM-12-e11021-s012.zip › EV_source_data/SourceData_FigEV4/FigEV4_9weeks_PAS_wt_STAT--.TIF]

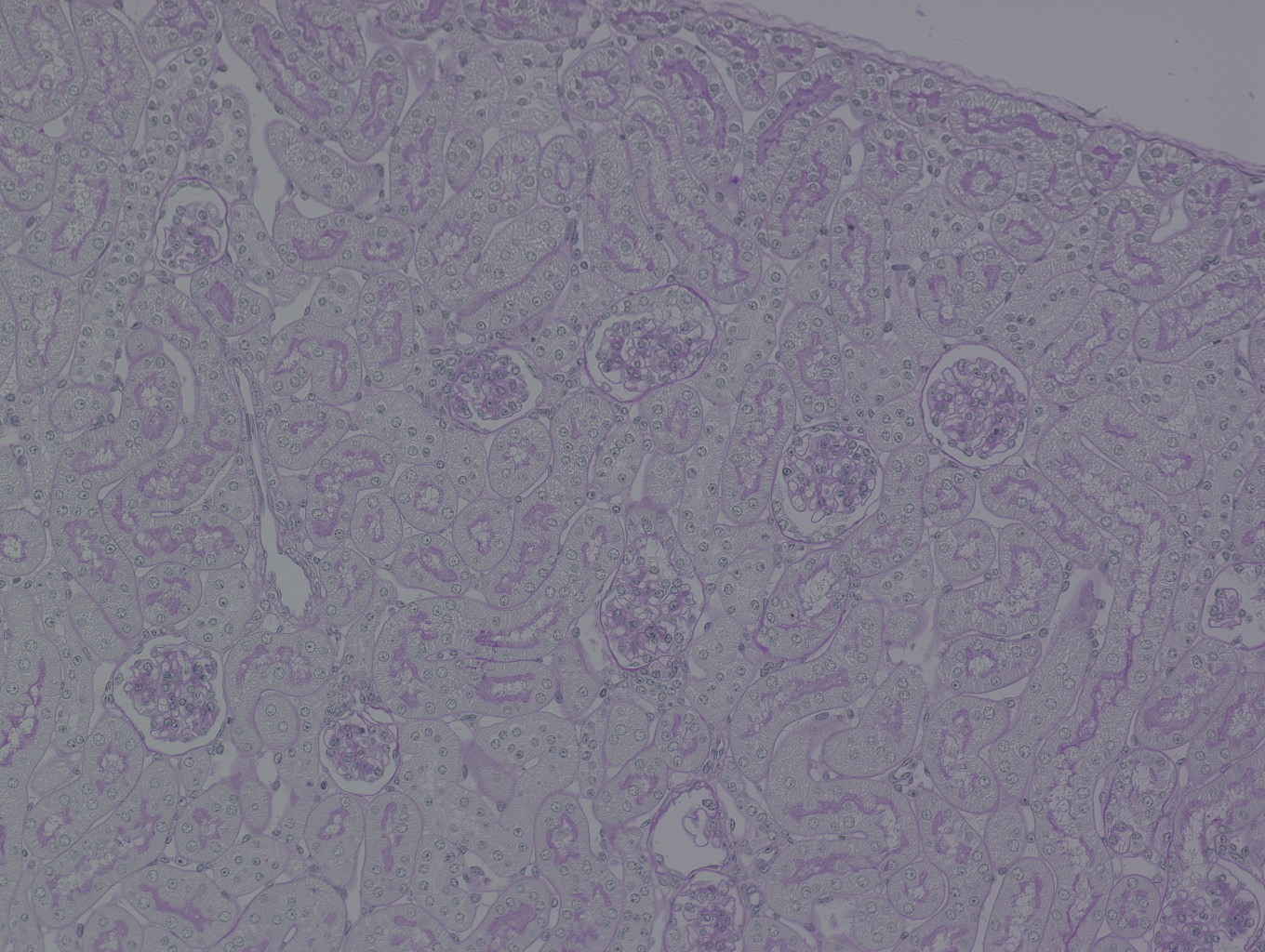

Supplement: Supplementary file 5 — Source Data for Expanded View [file EMMM-12-e11021-s012.zip › EV_source_data/SourceData_FigEV5/FigEV5Ca.TIF]

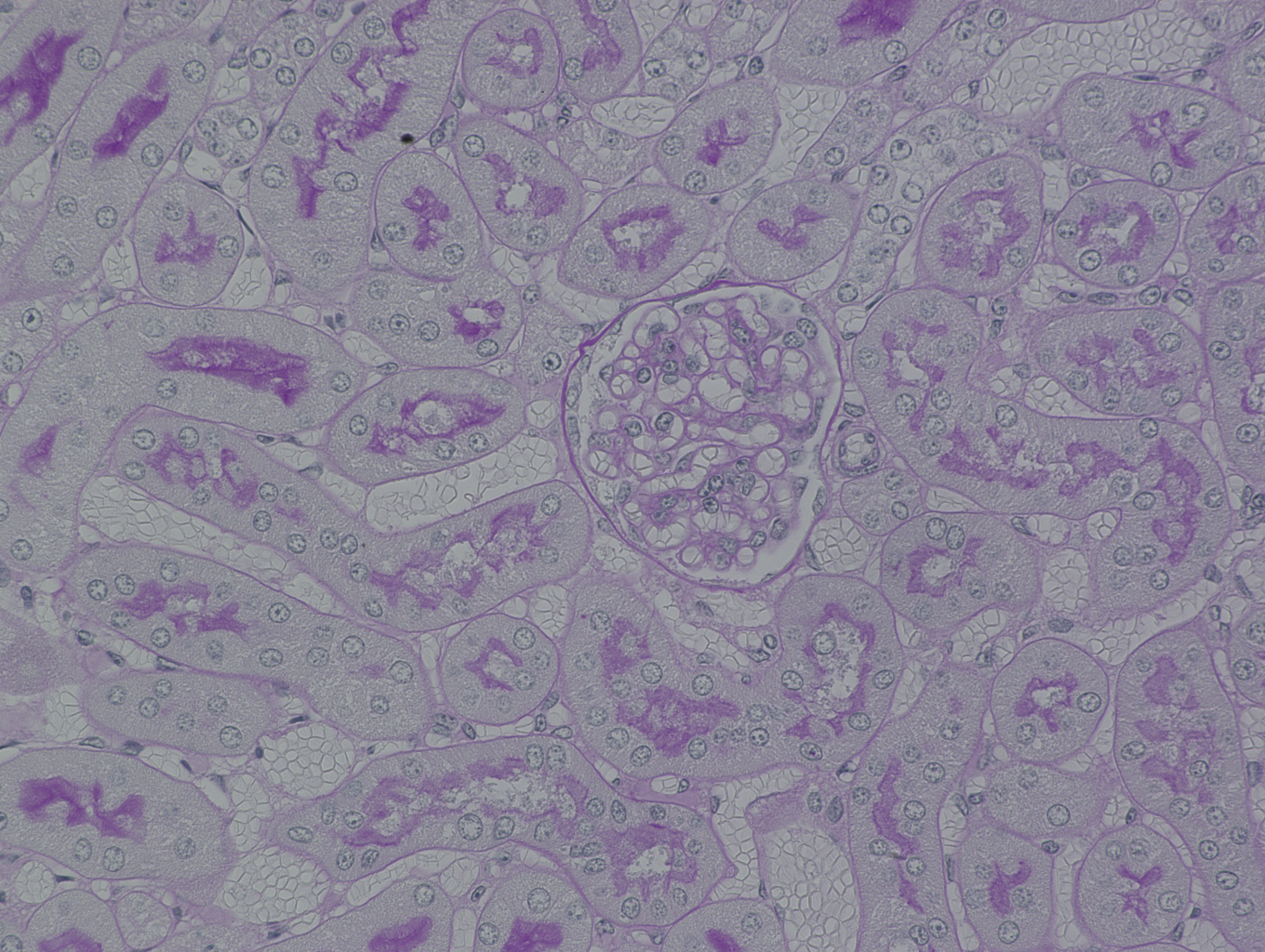

Supplement: Supplementary file 5 — Source Data for Expanded View [file EMMM-12-e11021-s012.zip › EV_source_data/SourceData_FigEV5/FigEV5Cb.tif]

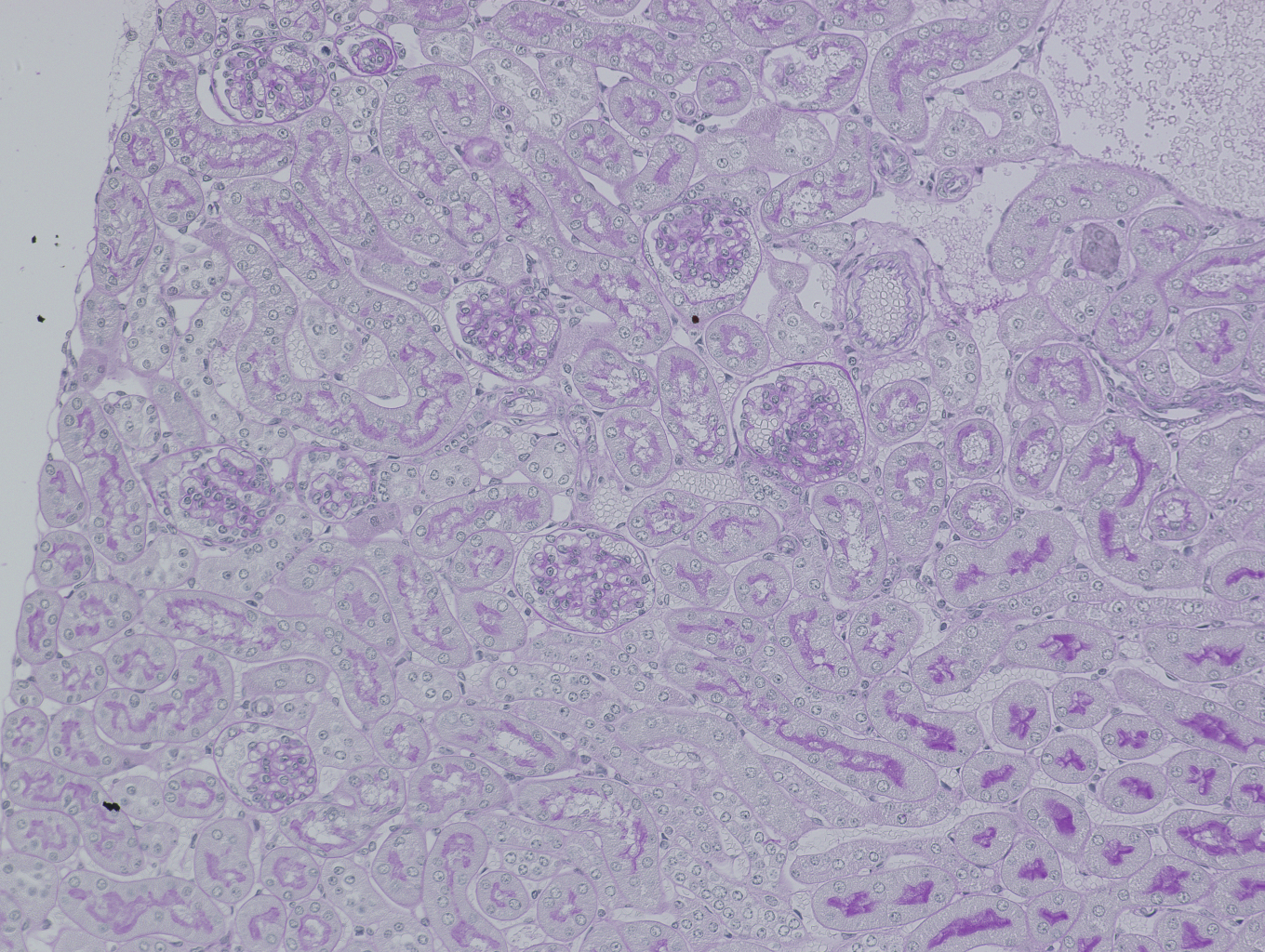

Supplement: Supplementary file 5 — Source Data for Expanded View [file EMMM-12-e11021-s012.zip › EV_source_data/SourceData_FigEV5/FigEV5Cc.tif]

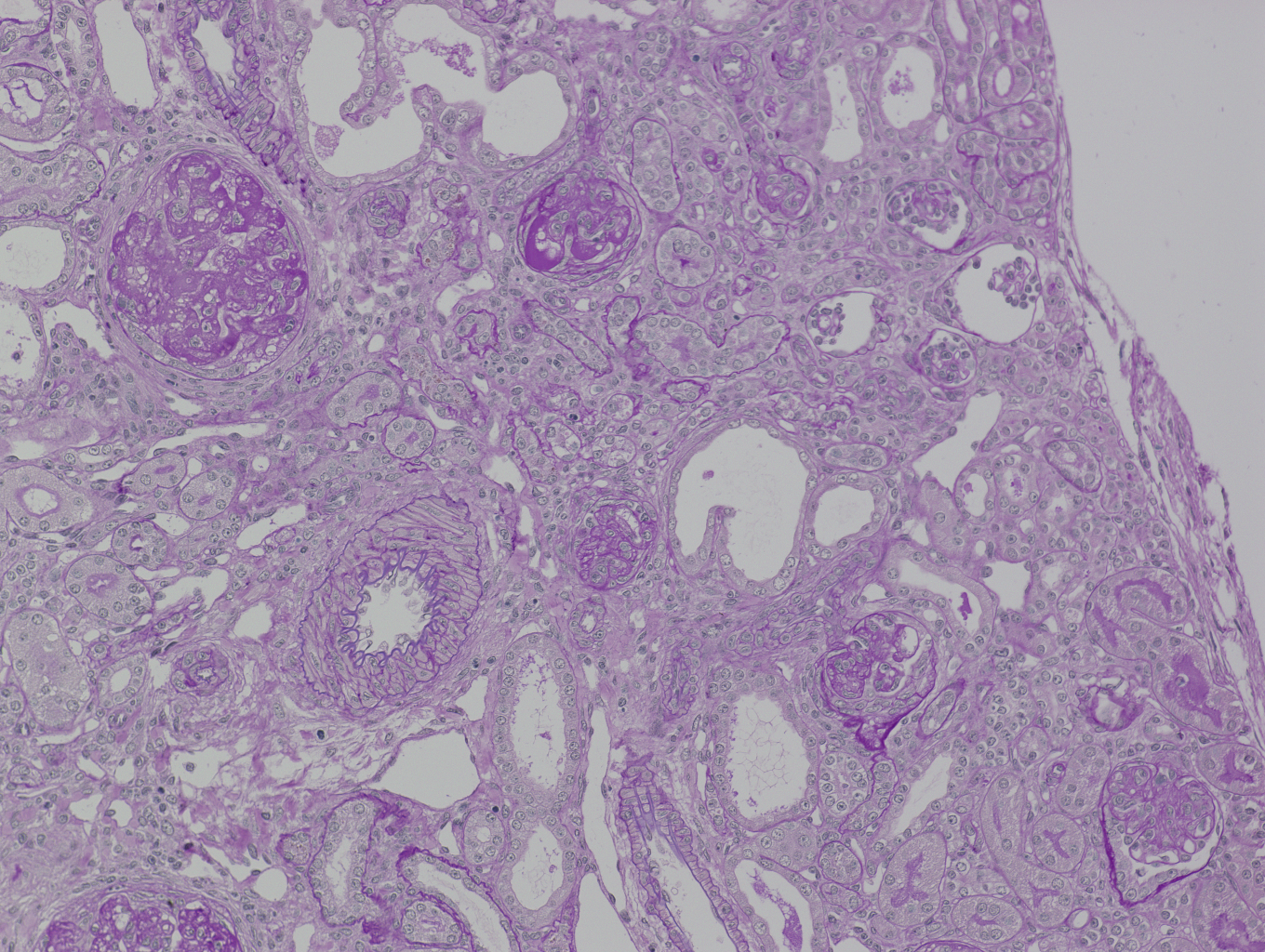

Supplement: Supplementary file 5 — Source Data for Expanded View [file EMMM-12-e11021-s012.zip › EV_source_data/SourceData_FigEV5/FigEV5Cd.tif]

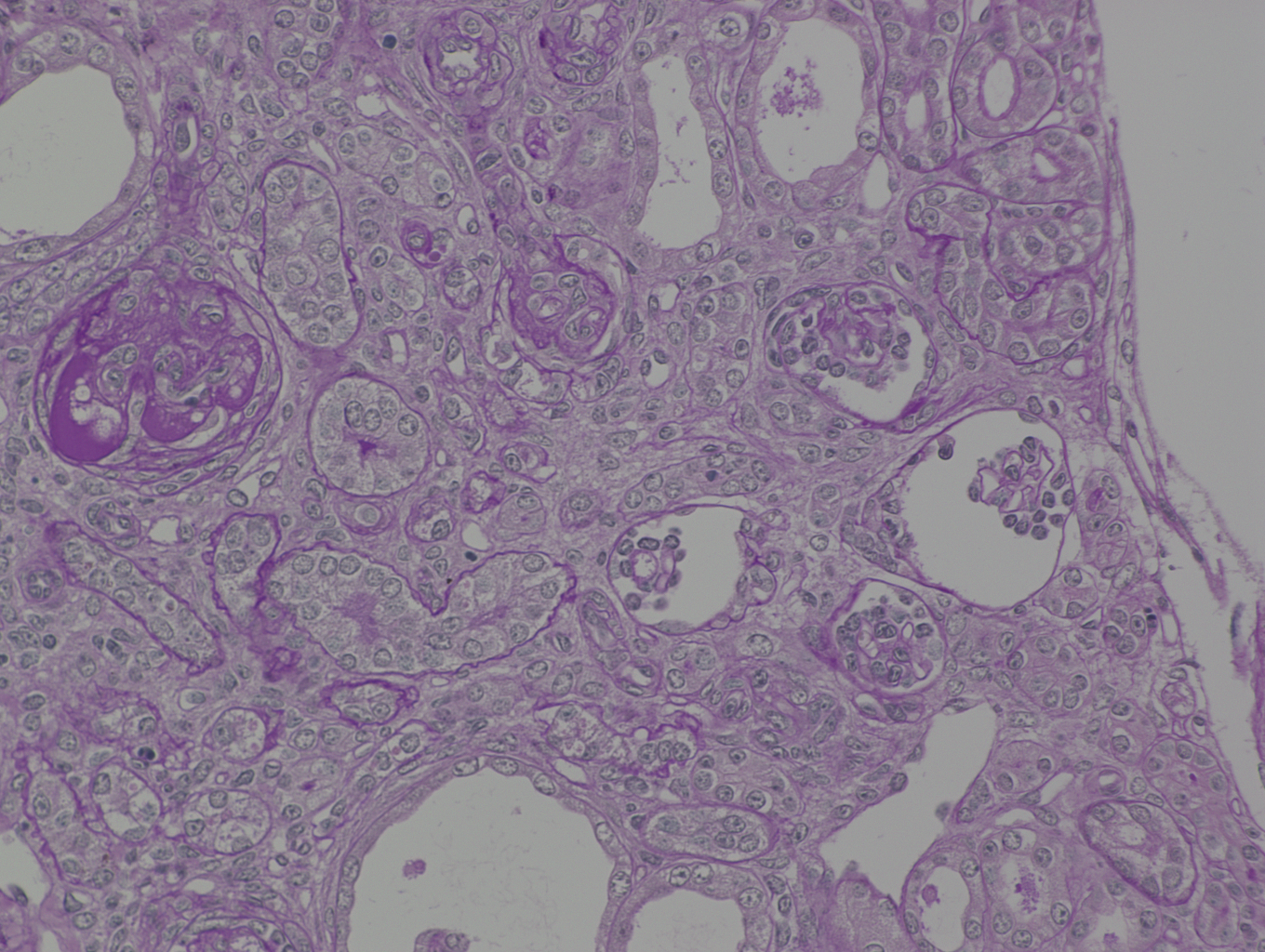

Supplement: Supplementary file 5 — Source Data for Expanded View [file EMMM-12-e11021-s012.zip › EV_source_data/SourceData_FigEV5/FigEV5Ce.tif]

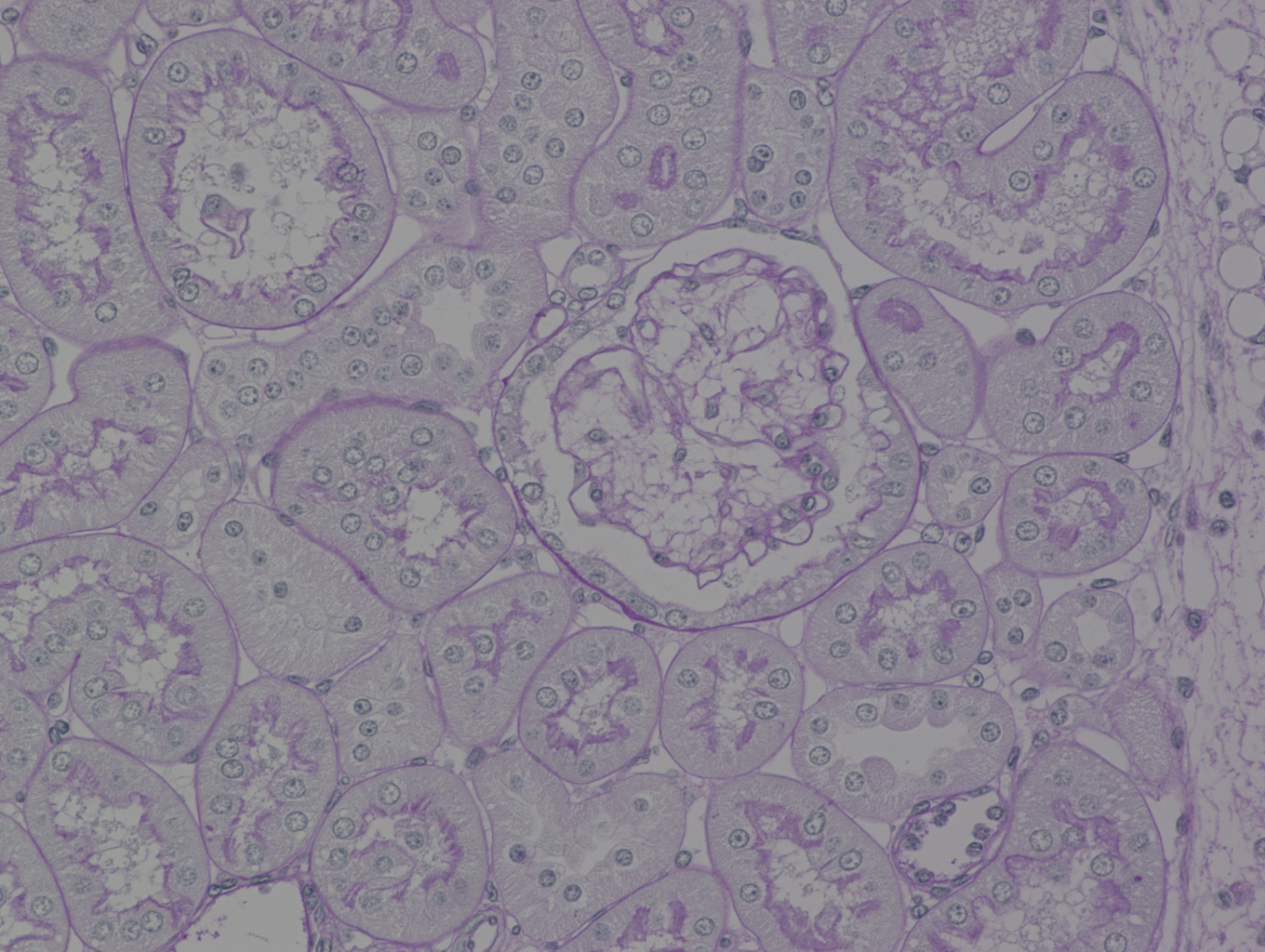

Supplement: Supplementary file 5 — Source Data for Expanded View [file EMMM-12-e11021-s012.zip › EV_source_data/SourceData_FigEV5/FigEV5Cf.tif]

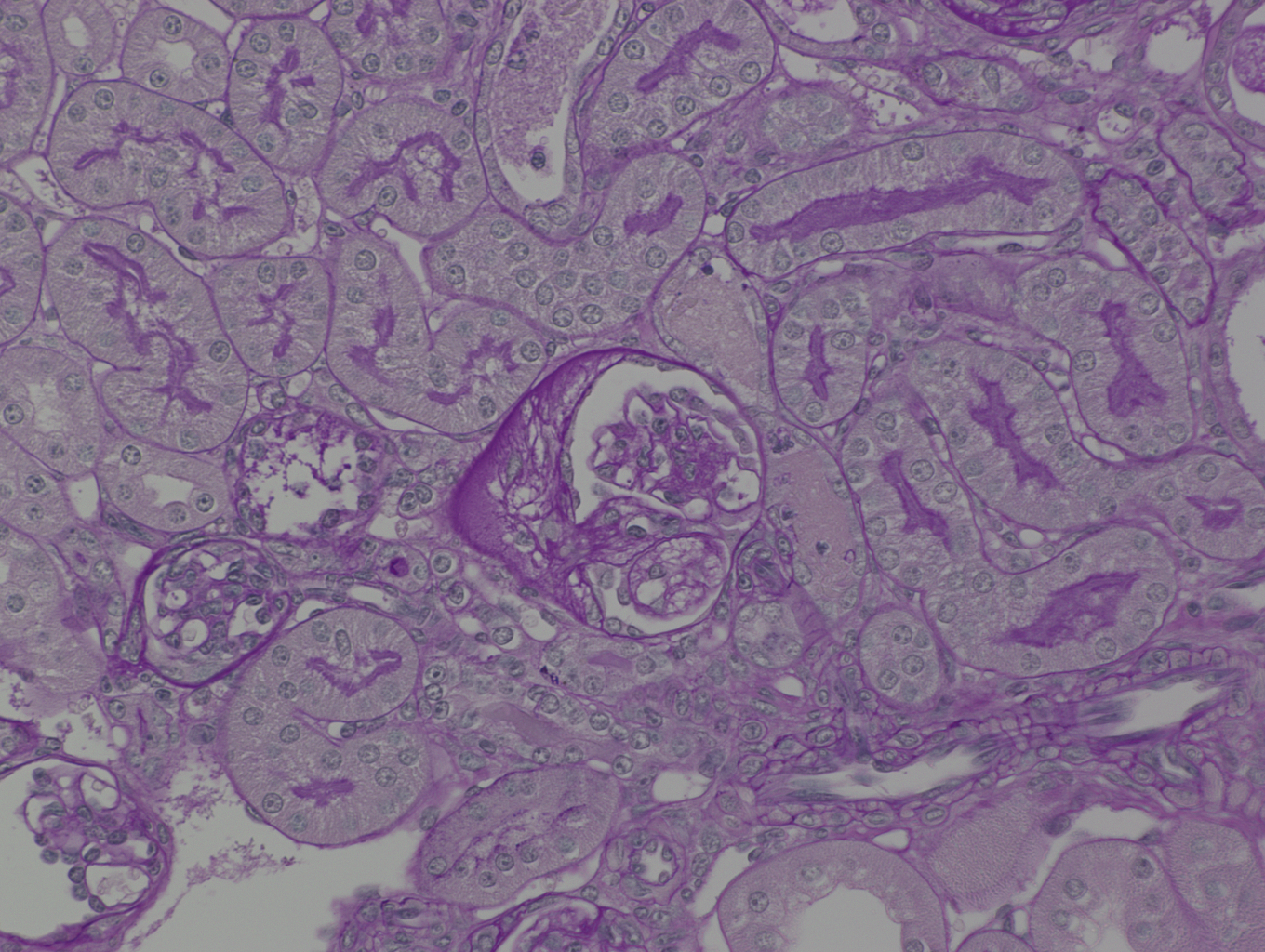

Supplement: Supplementary file 5 — Source Data for Expanded View [file EMMM-12-e11021-s012.zip › EV_source_data/SourceData_FigEV5/FigEV5Cg.tif]

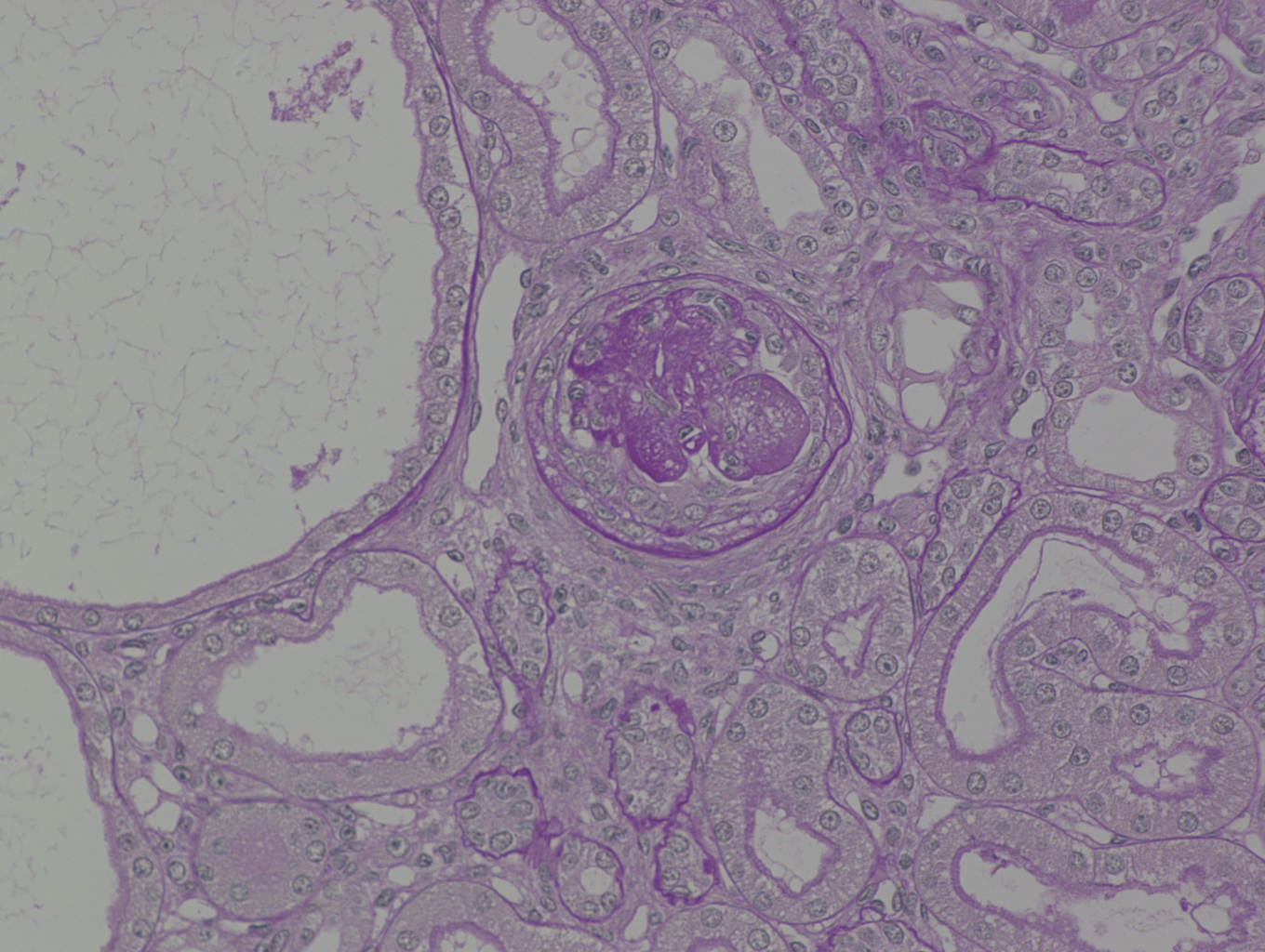

Supplement: Supplementary file 5 — Source Data for Expanded View [file EMMM-12-e11021-s012.zip › EV_source_data/SourceData_FigEV5/FigEV5Ch.tif]

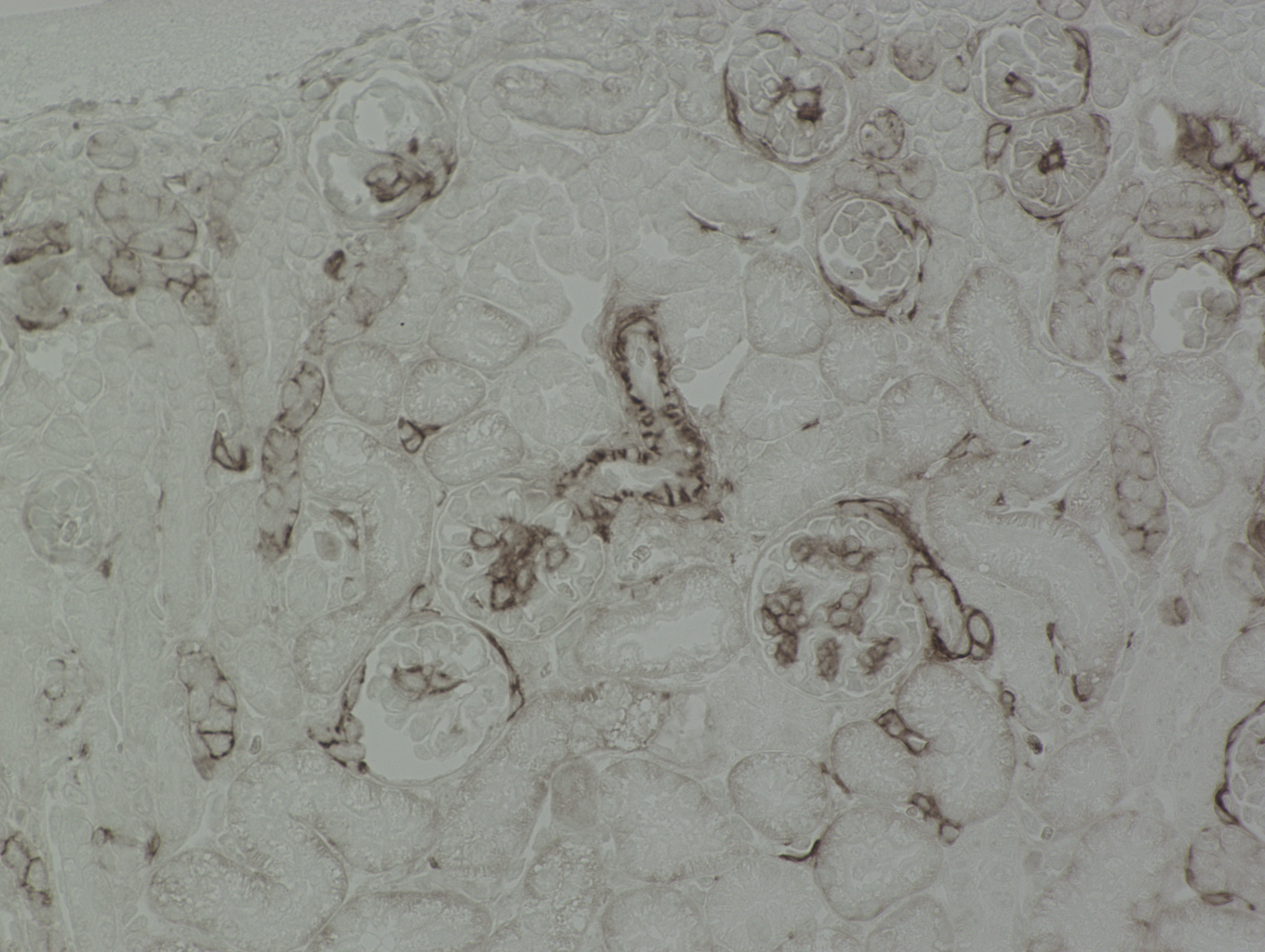

Supplement: Supplementary file 5 — Source Data for Expanded View [file EMMM-12-e11021-s012.zip › EV_source_data/SourceData_FigEV5/FigEV5D_knockout_NG2.tif]

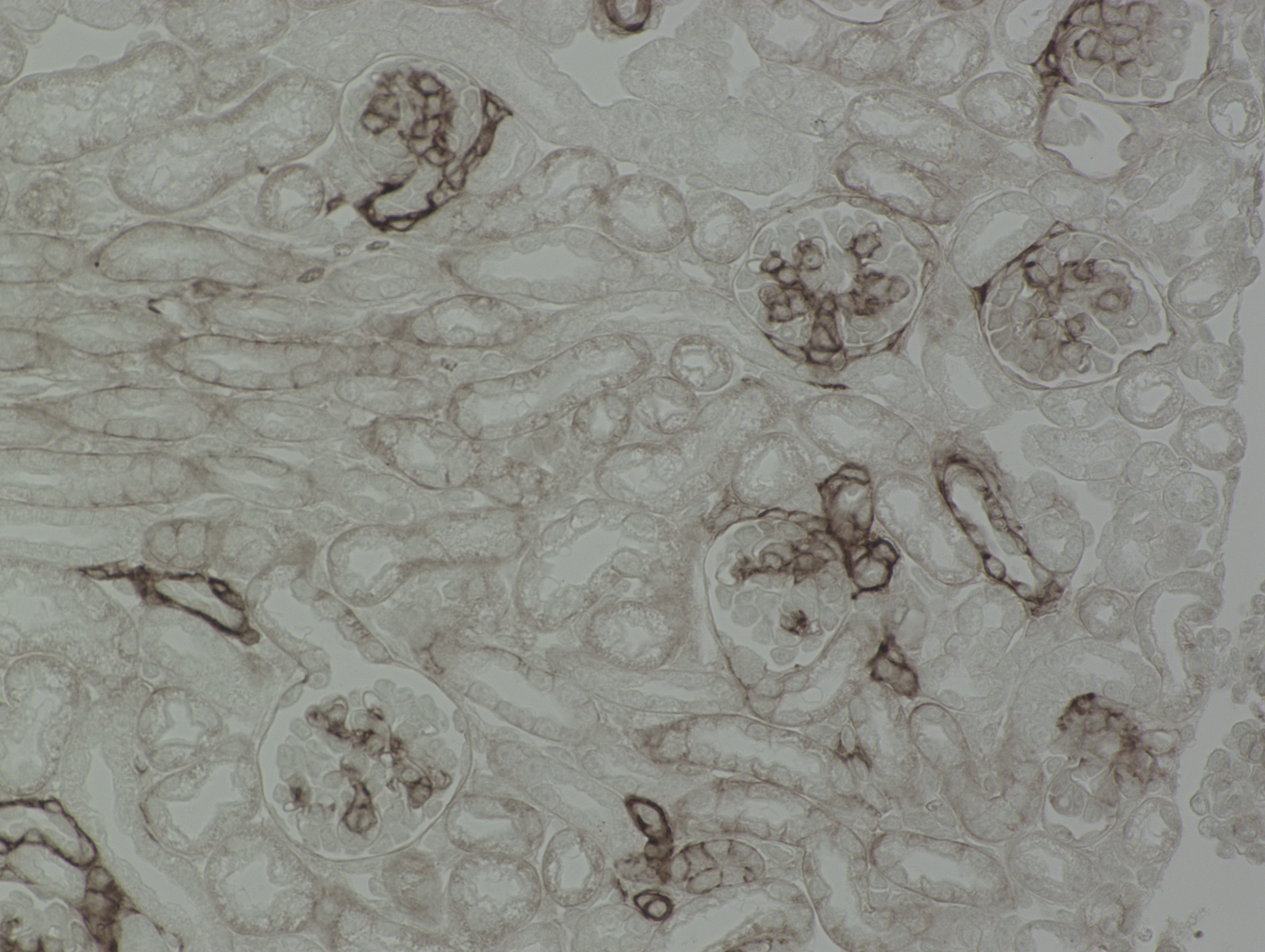

Supplement: Supplementary file 5 — Source Data for Expanded View [file EMMM-12-e11021-s012.zip › EV_source_data/SourceData_FigEV5/FigEV5D_wt_NG2.tif]

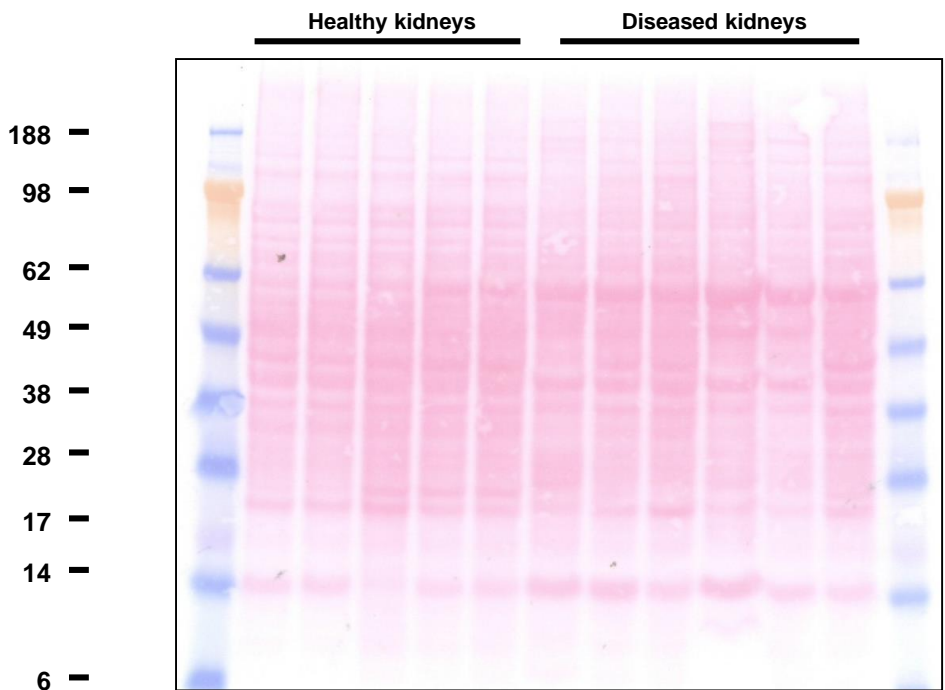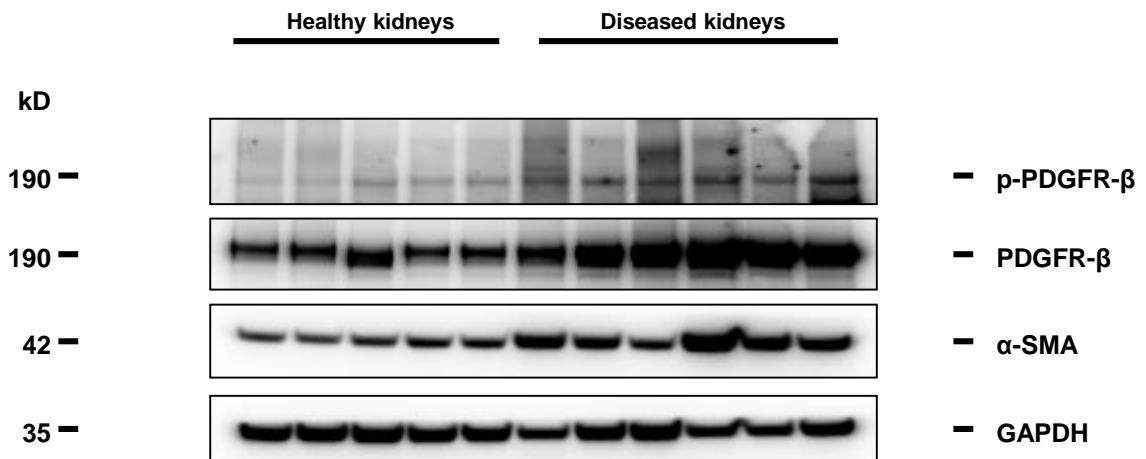

Supplement: Supplementary file 7 — Source Data for Figure 1 [file EMMM-12-e11021-s005.zip › SourceData_Fig1/Fig1A.pdf]

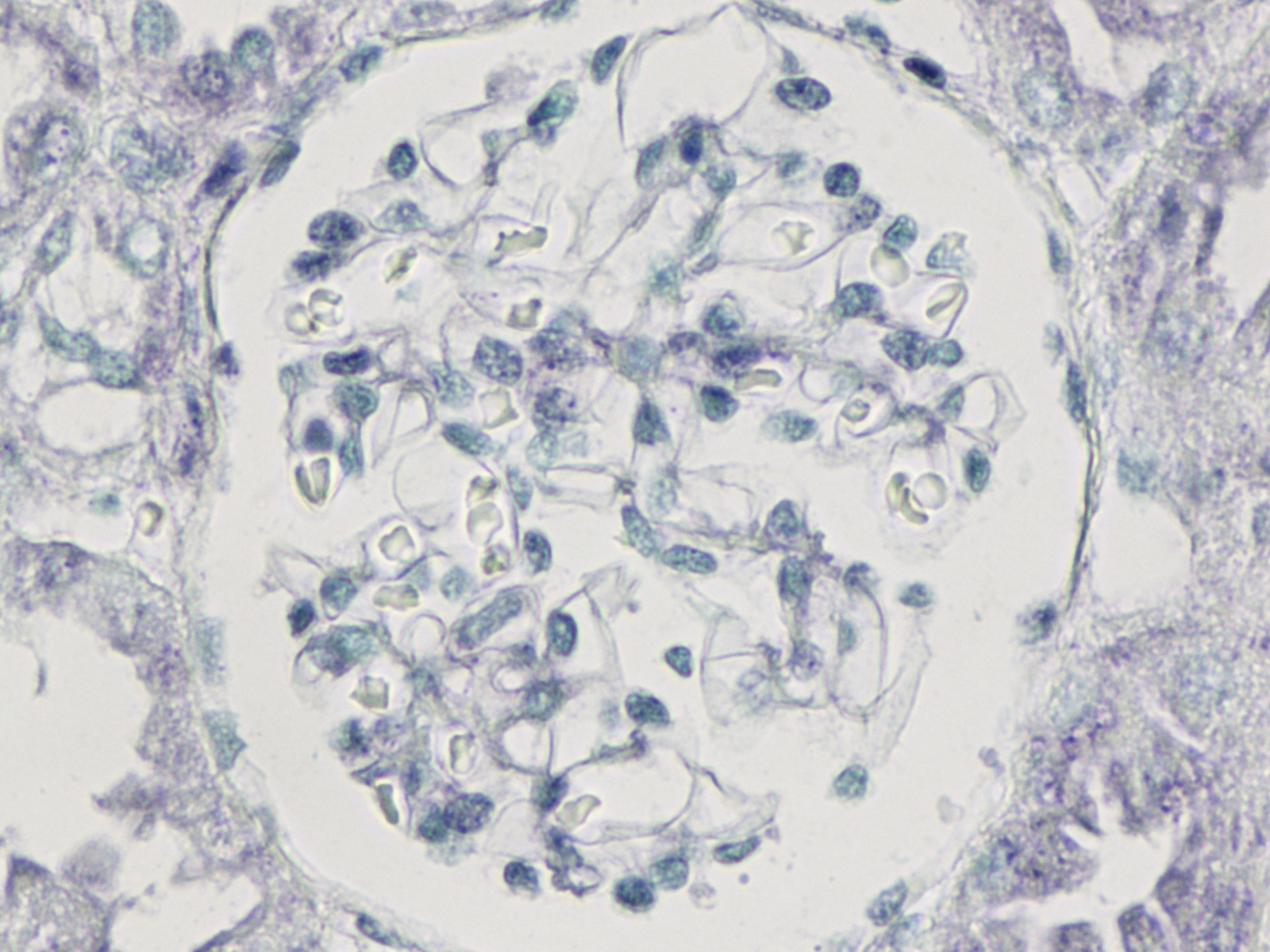

Supplement: Supplementary file 7 — Source Data for Figure 1 [file EMMM-12-e11021-s005.zip › SourceData_Fig1/Fig1Ba.tif]

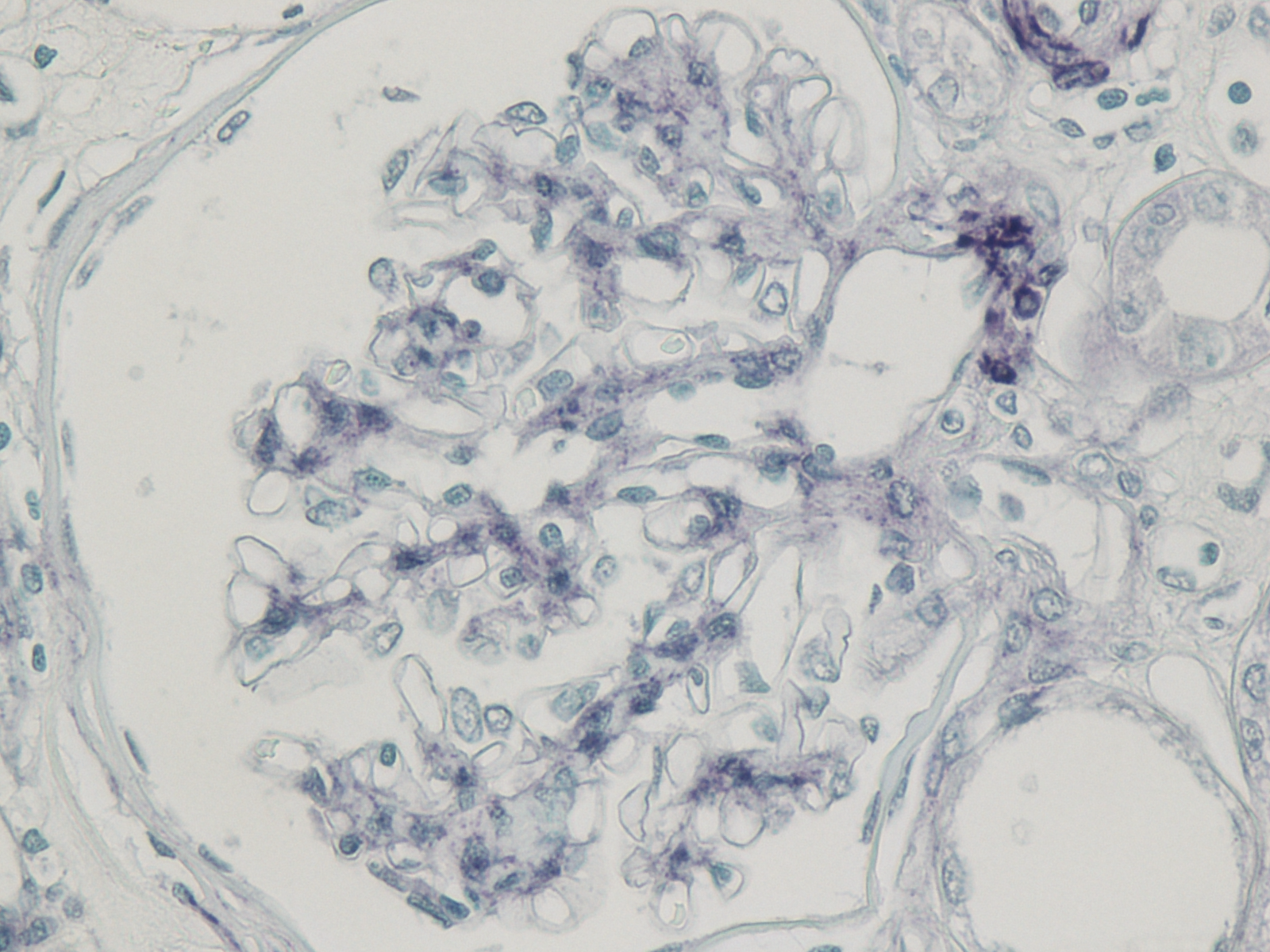

Supplement: Supplementary file 7 — Source Data for Figure 1 [file EMMM-12-e11021-s005.zip › SourceData_Fig1/Fig1Bb.tif]

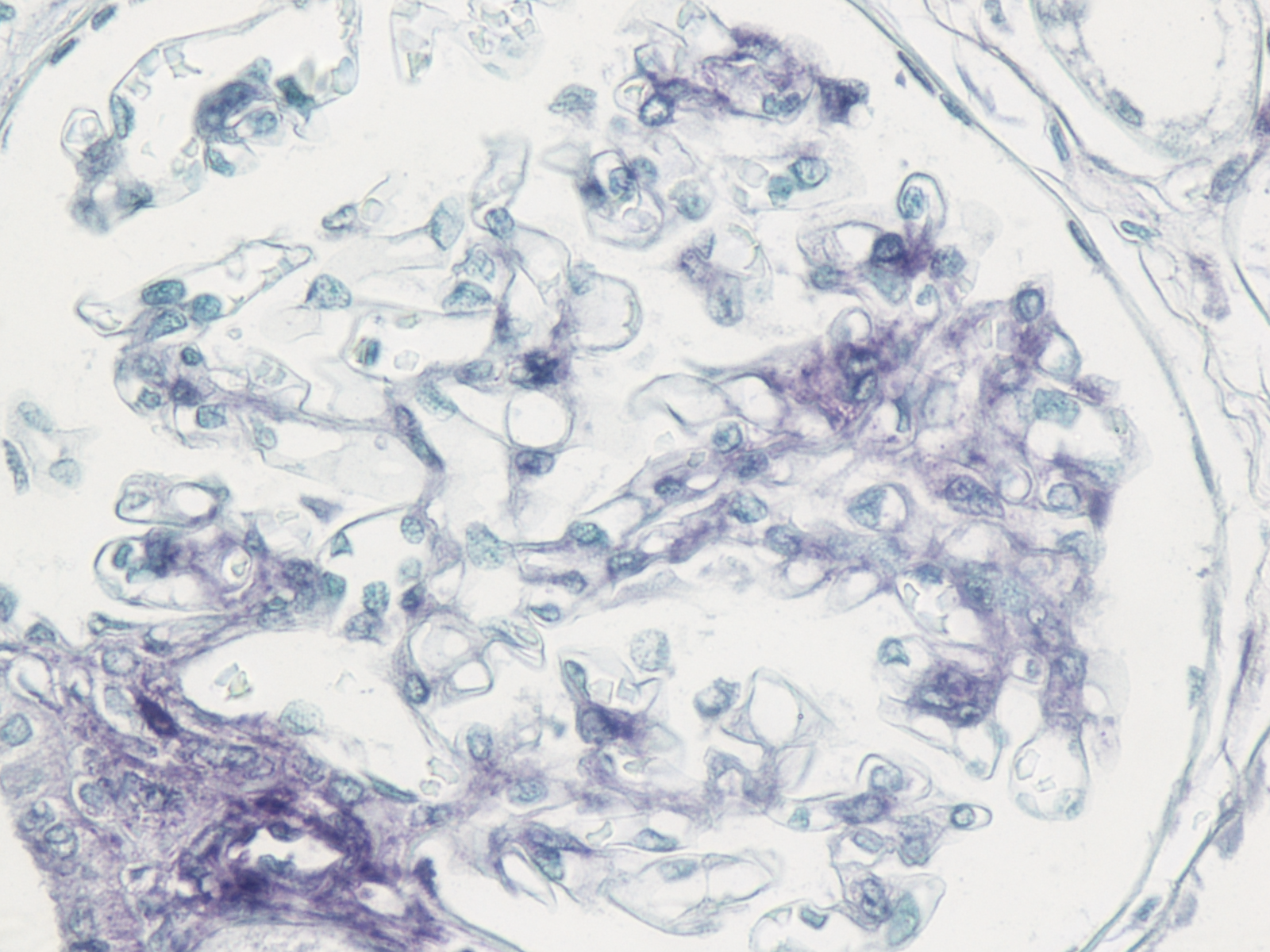

Supplement: Supplementary file 7 — Source Data for Figure 1 [file EMMM-12-e11021-s005.zip › SourceData_Fig1/Fig1Bc.tif]

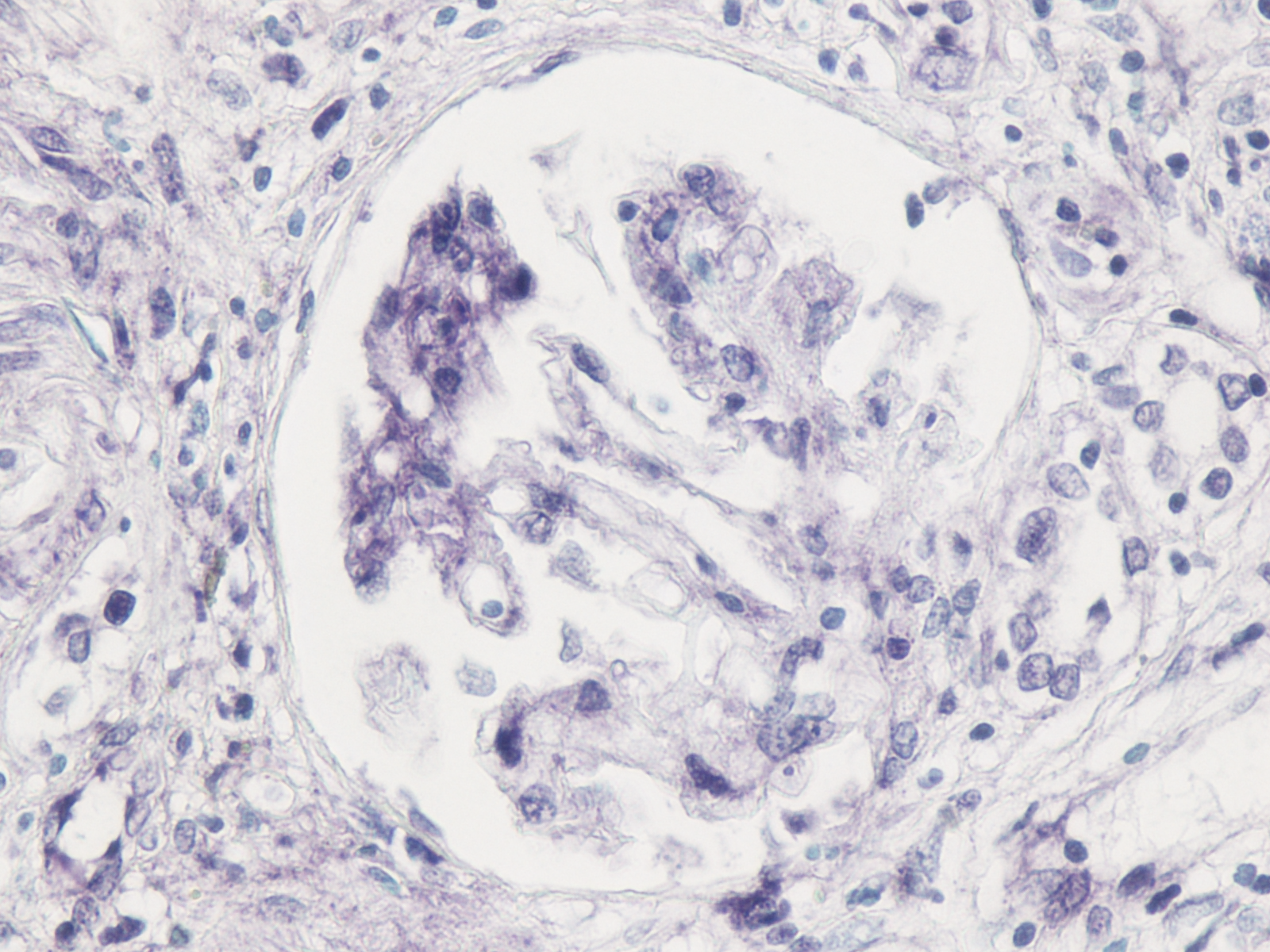

Supplement: Supplementary file 7 — Source Data for Figure 1 [file EMMM-12-e11021-s005.zip › SourceData_Fig1/Fig1Bd.tif]

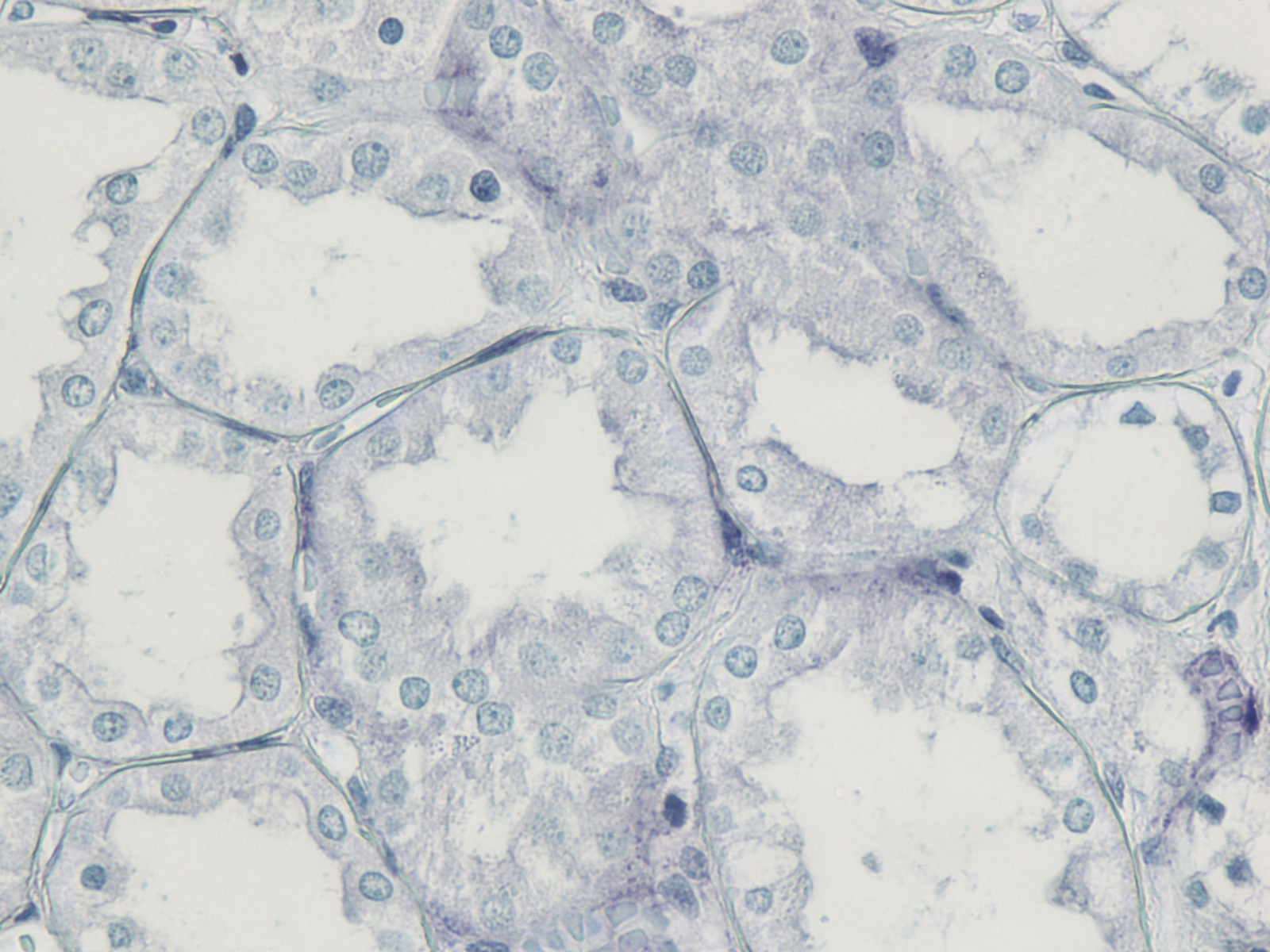

Supplement: Supplementary file 7 — Source Data for Figure 1 [file EMMM-12-e11021-s005.zip › SourceData_Fig1/Fig1Be.tif]

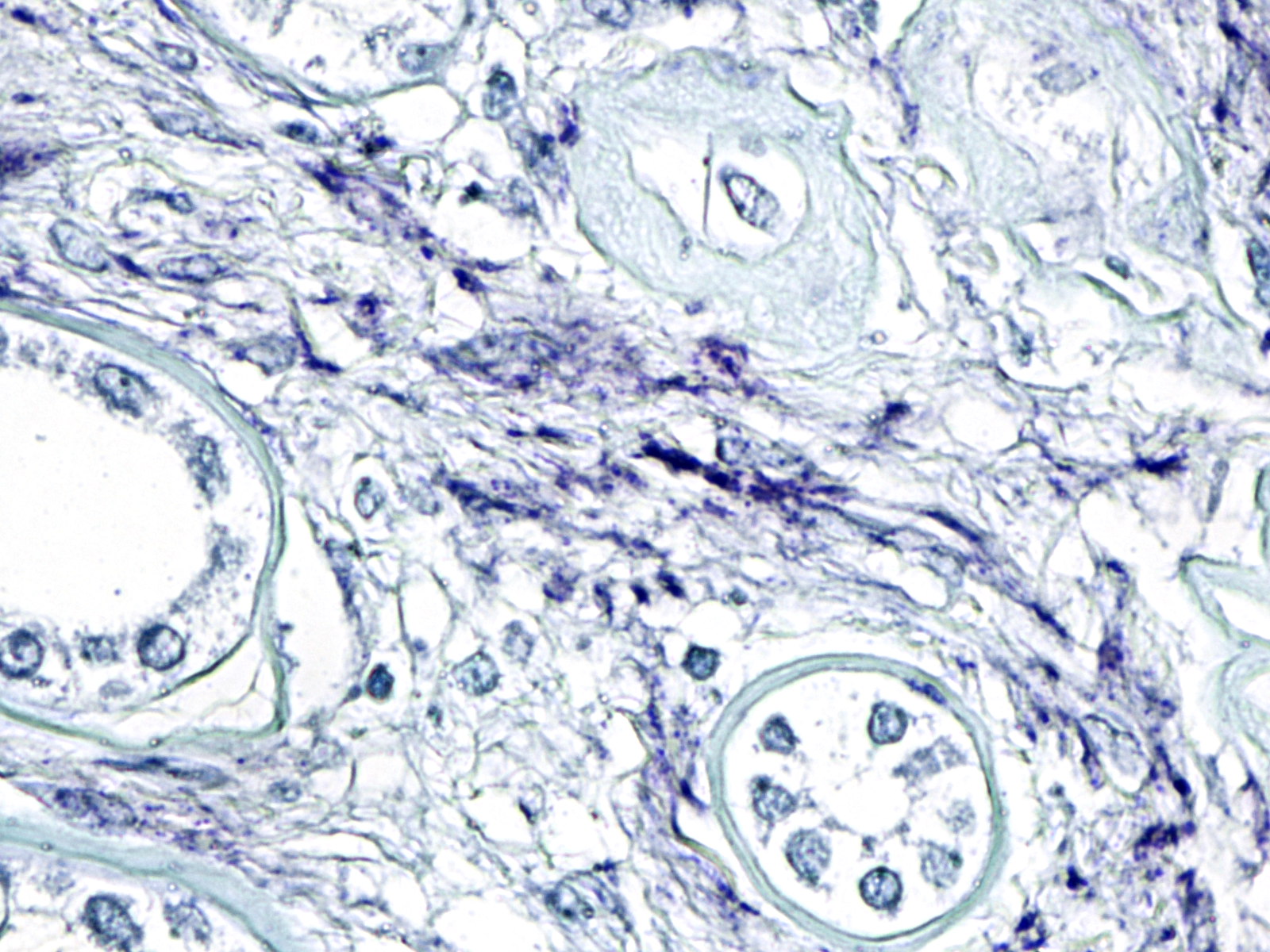

Supplement: Supplementary file 7 — Source Data for Figure 1 [file EMMM-12-e11021-s005.zip › SourceData_Fig1/Fig1Bf.tif]

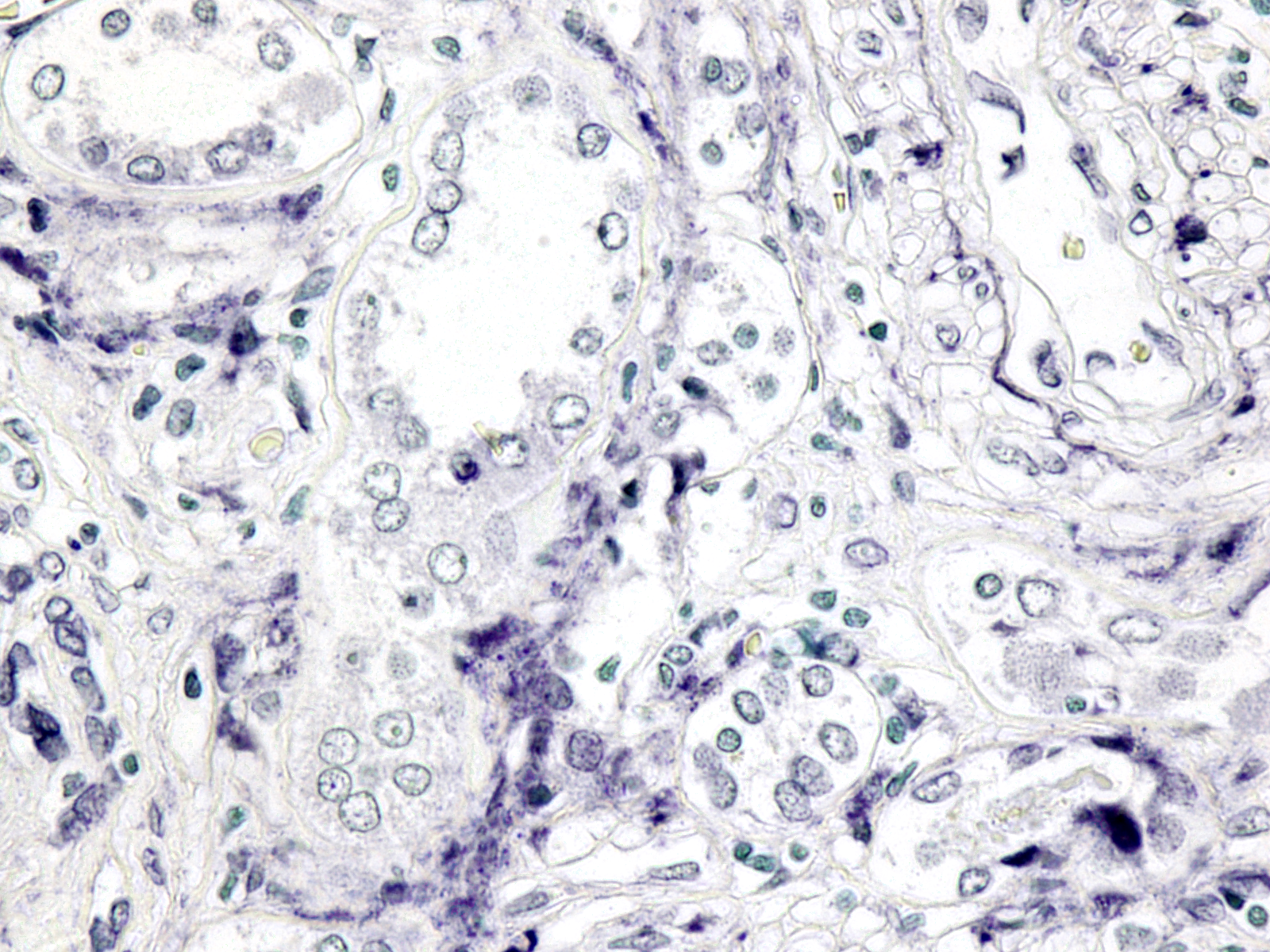

Supplement: Supplementary file 7 — Source Data for Figure 1 [file EMMM-12-e11021-s005.zip › SourceData_Fig1/Fig1Bg.tif]

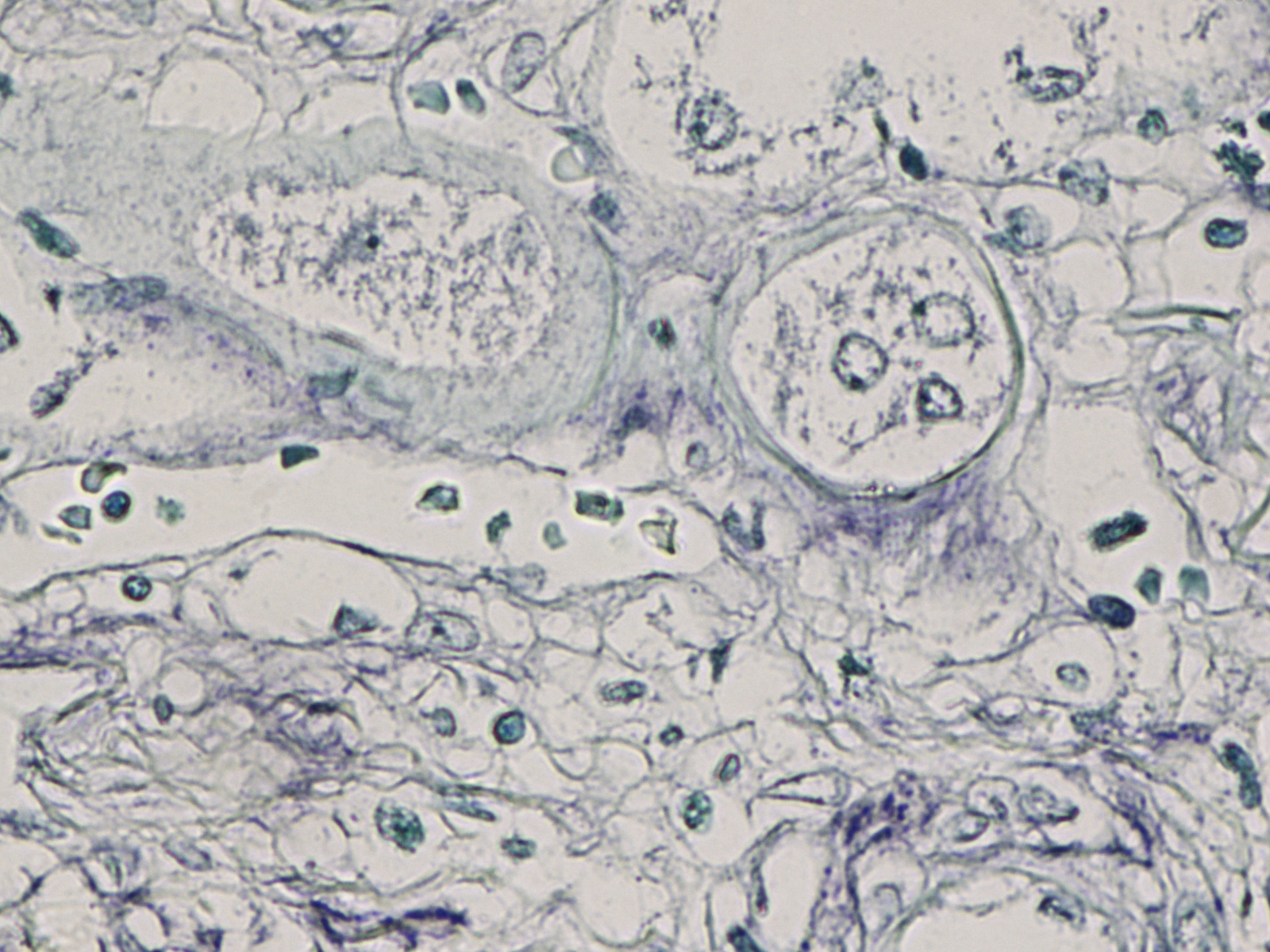

Supplement: Supplementary file 7 — Source Data for Figure 1 [file EMMM-12-e11021-s005.zip › SourceData_Fig1/Fig1Bh.tif]

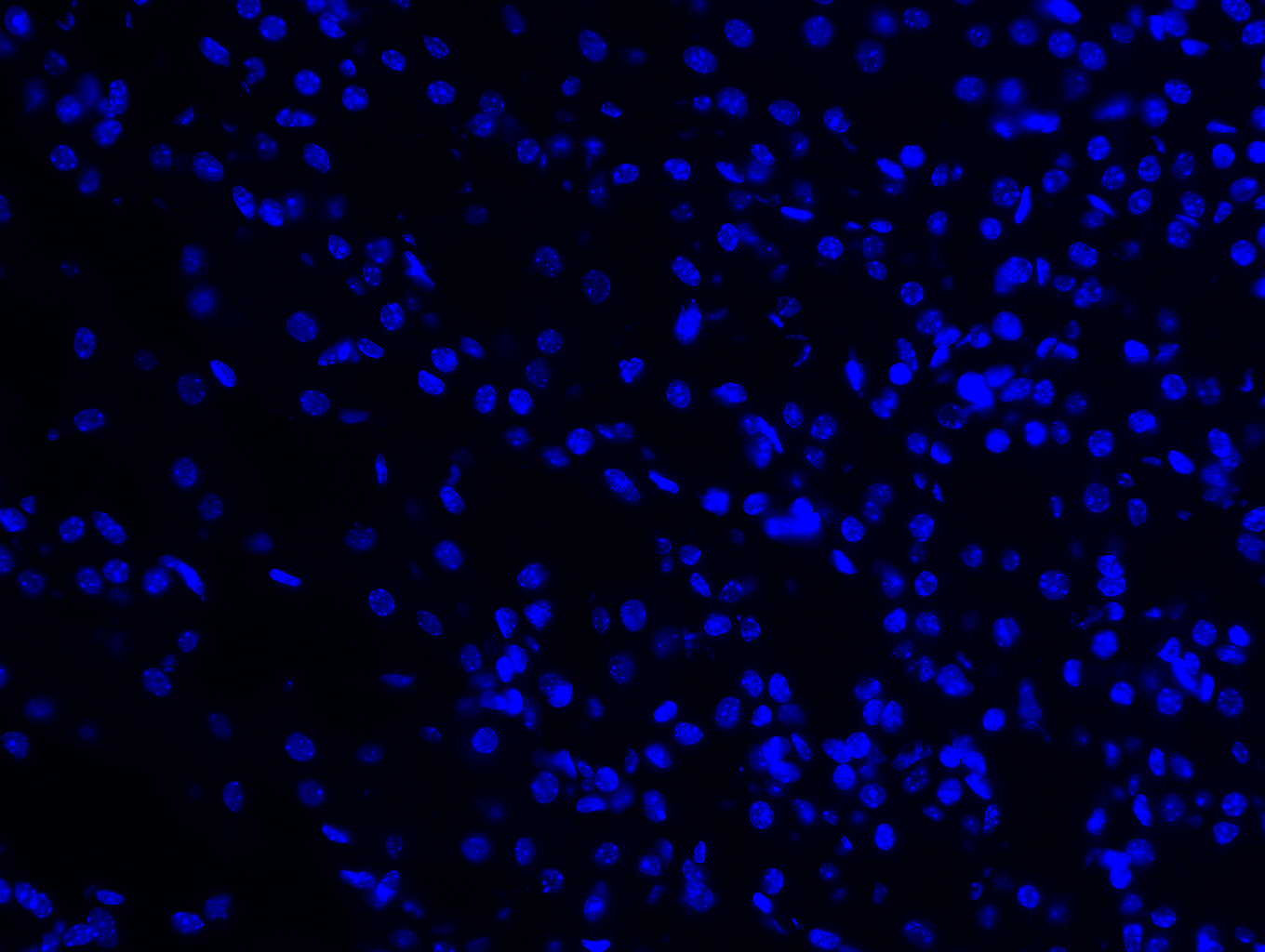

Supplement: Supplementary file 7 — Source Data for Figure 1 [file EMMM-12-e11021-s005.zip › SourceData_Fig1/Fig1C_Cortex_DAPI.TIF]

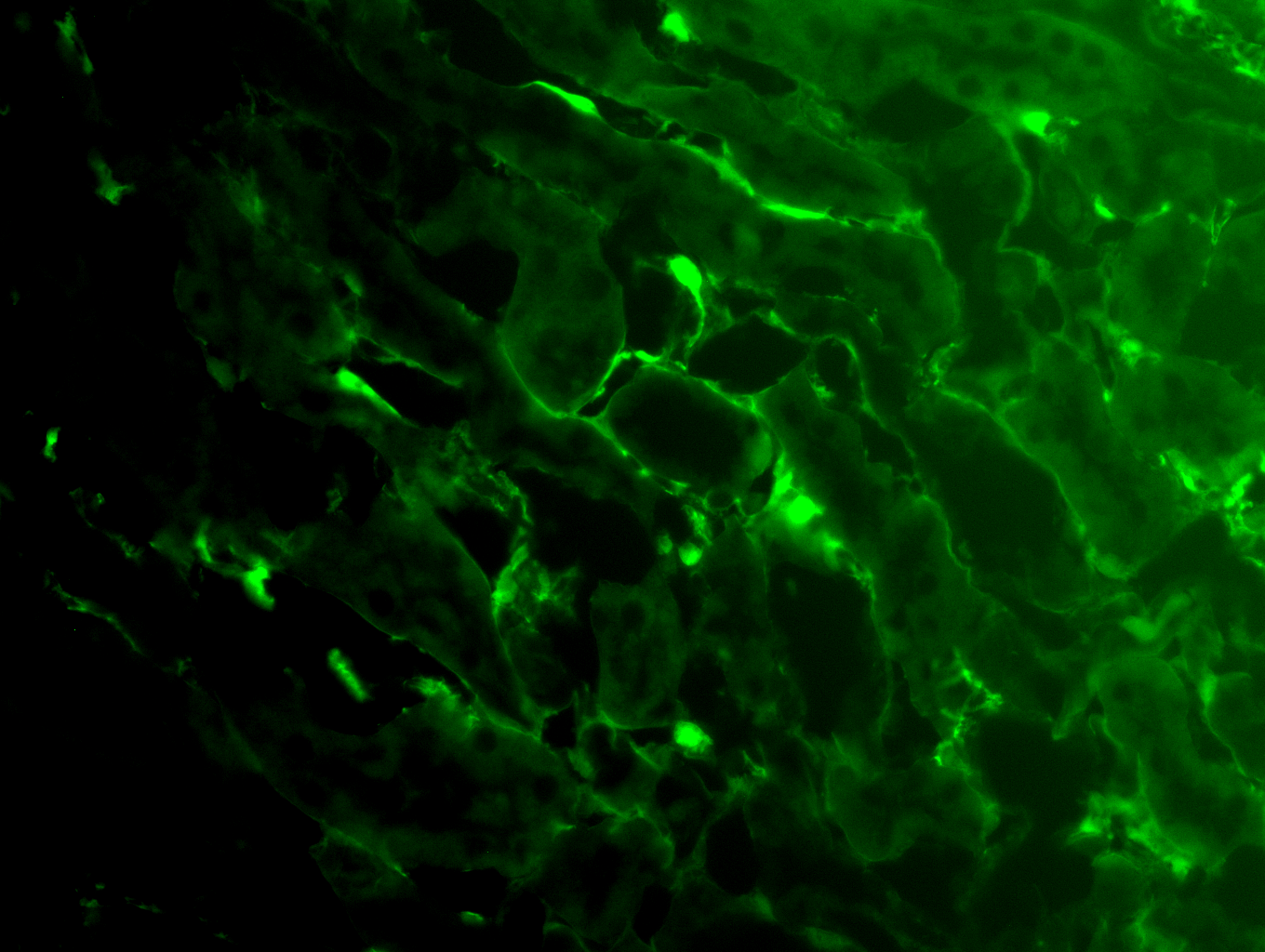

Supplement: Supplementary file 7 — Source Data for Figure 1 [file EMMM-12-e11021-s005.zip › SourceData_Fig1/Fig1C_Cortex_GFP.TIF]

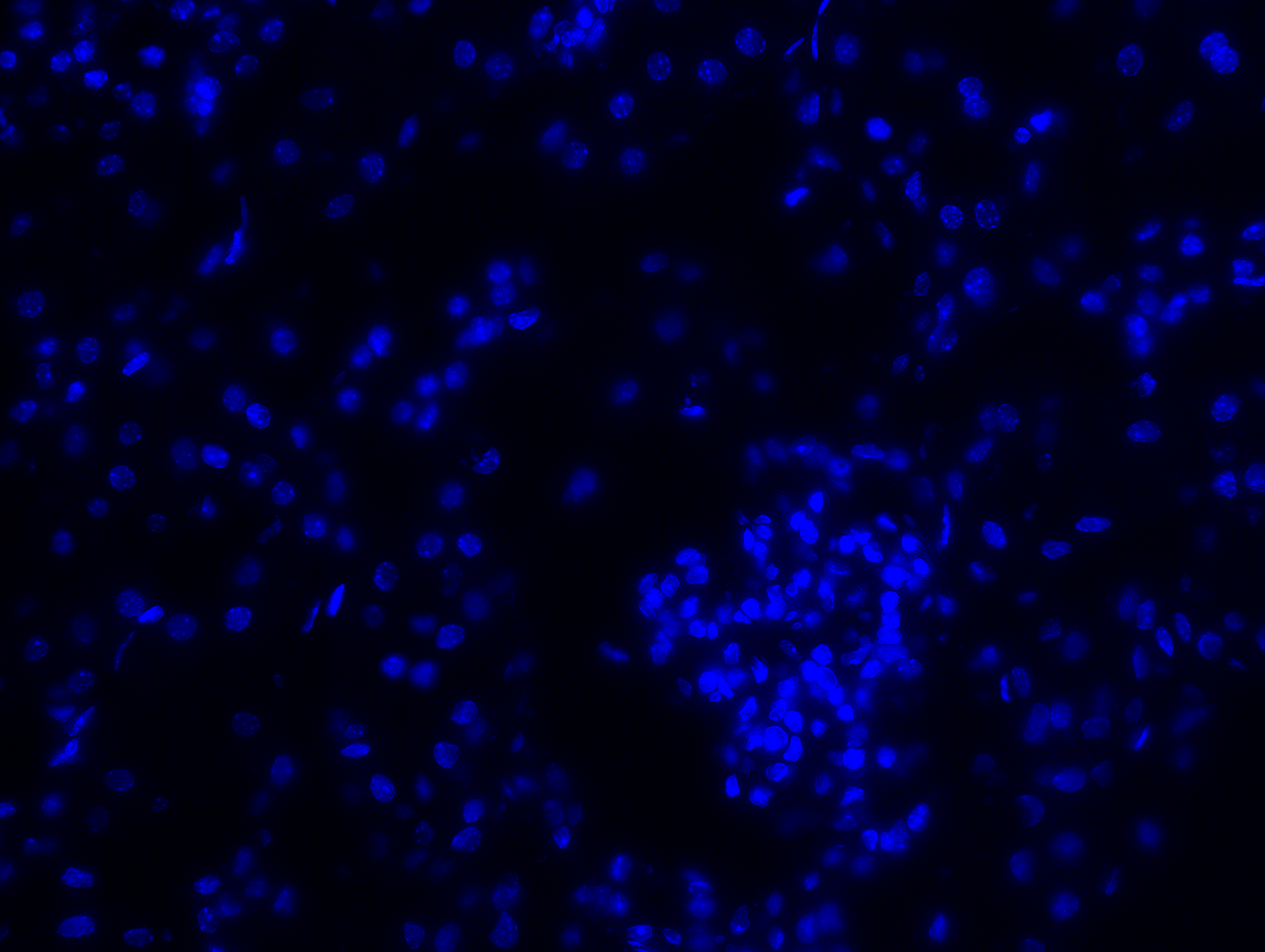

Supplement: Supplementary file 7 — Source Data for Figure 1 [file EMMM-12-e11021-s005.zip › SourceData_Fig1/Fig1C_Glomerulus_DAPI.TIF]

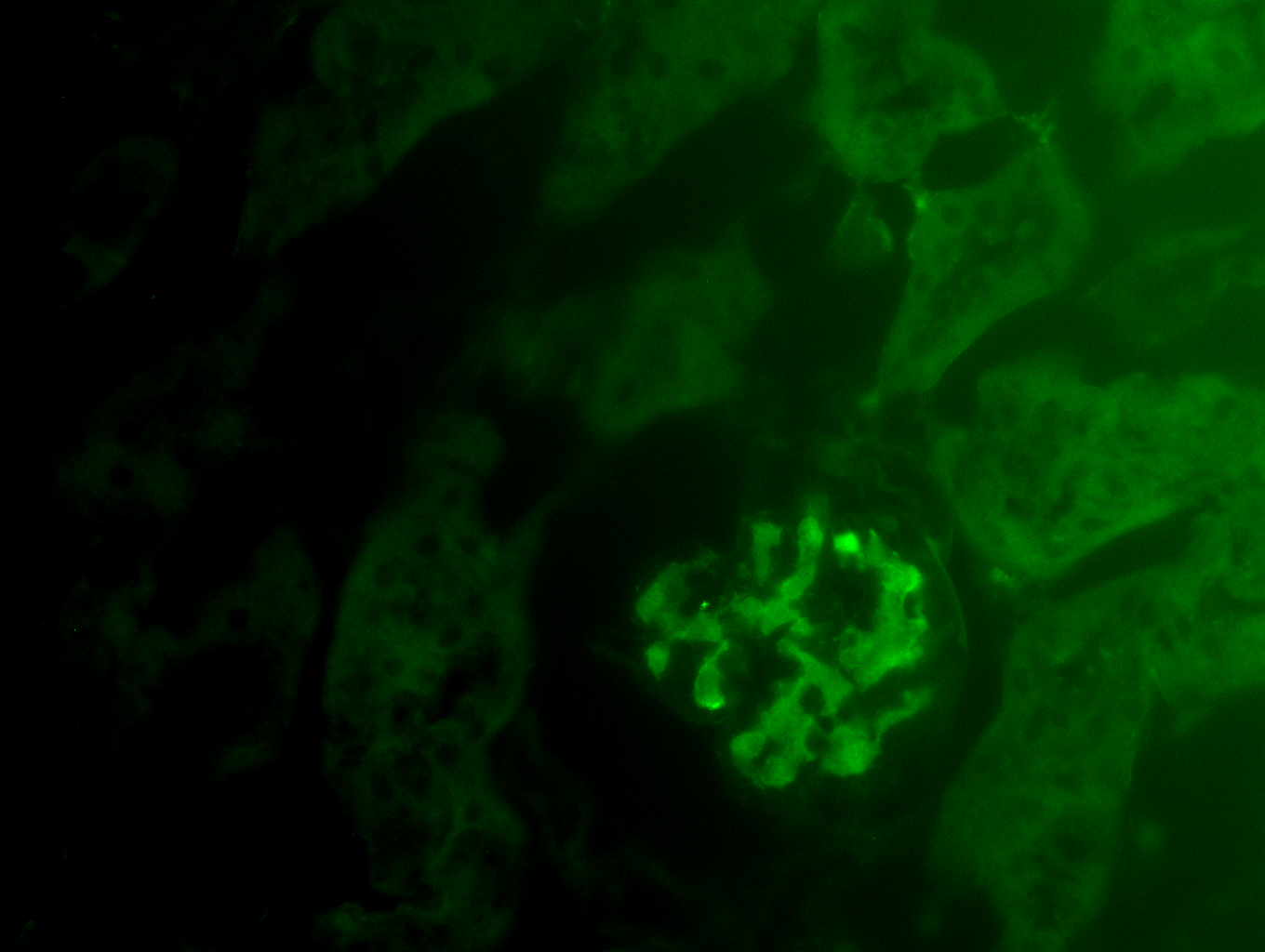

Supplement: Supplementary file 7 — Source Data for Figure 1 [file EMMM-12-e11021-s005.zip › SourceData_Fig1/Fig1C_Glomerulus_GFP.TIF]

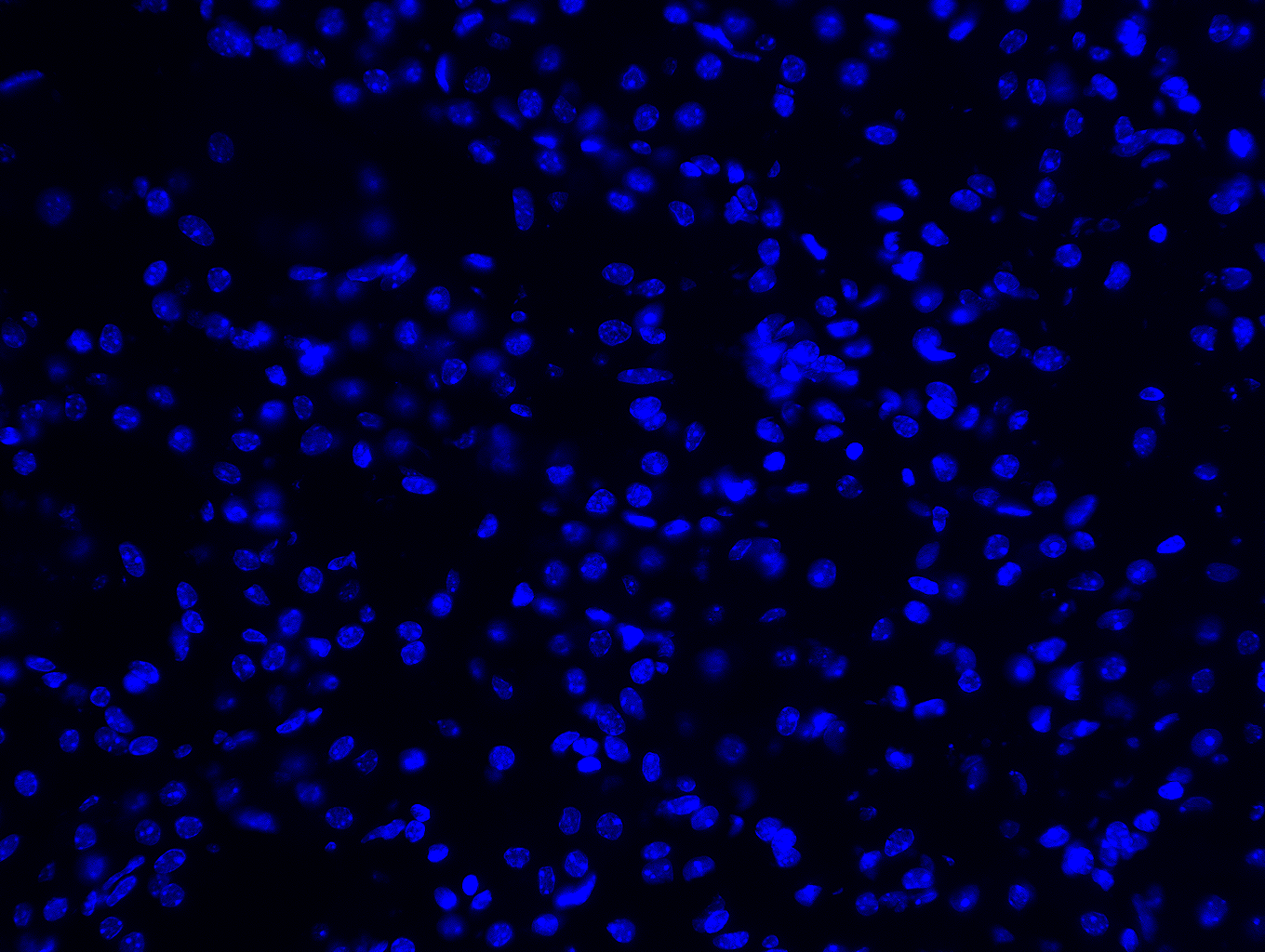

Supplement: Supplementary file 7 — Source Data for Figure 1 [file EMMM-12-e11021-s005.zip › SourceData_Fig1/Fig1C_Medulla_DAPI.TIF]

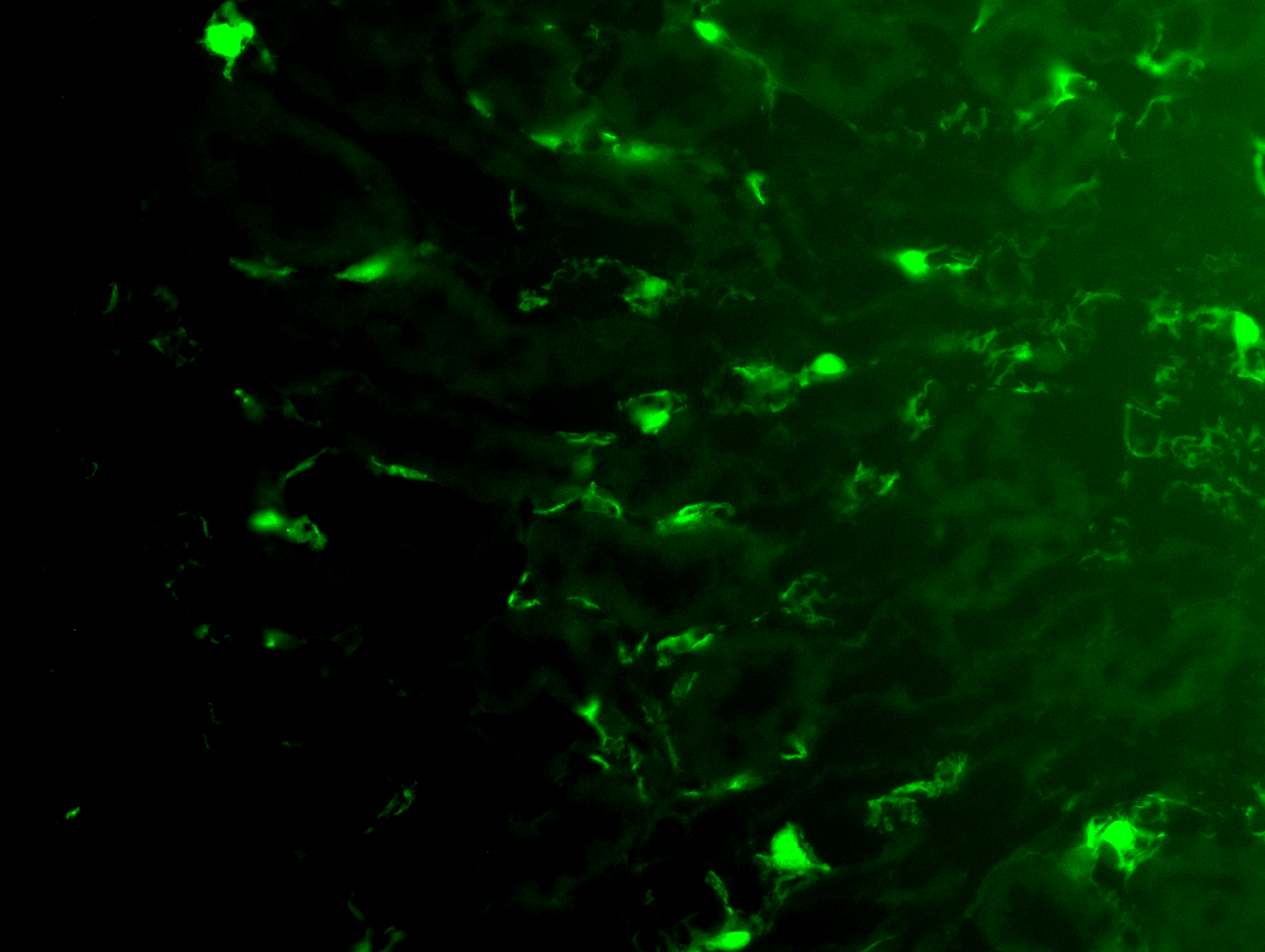

Supplement: Supplementary file 7 — Source Data for Figure 1 [file EMMM-12-e11021-s005.zip › SourceData_Fig1/Fig1C_Medulla_GFP.TIF]

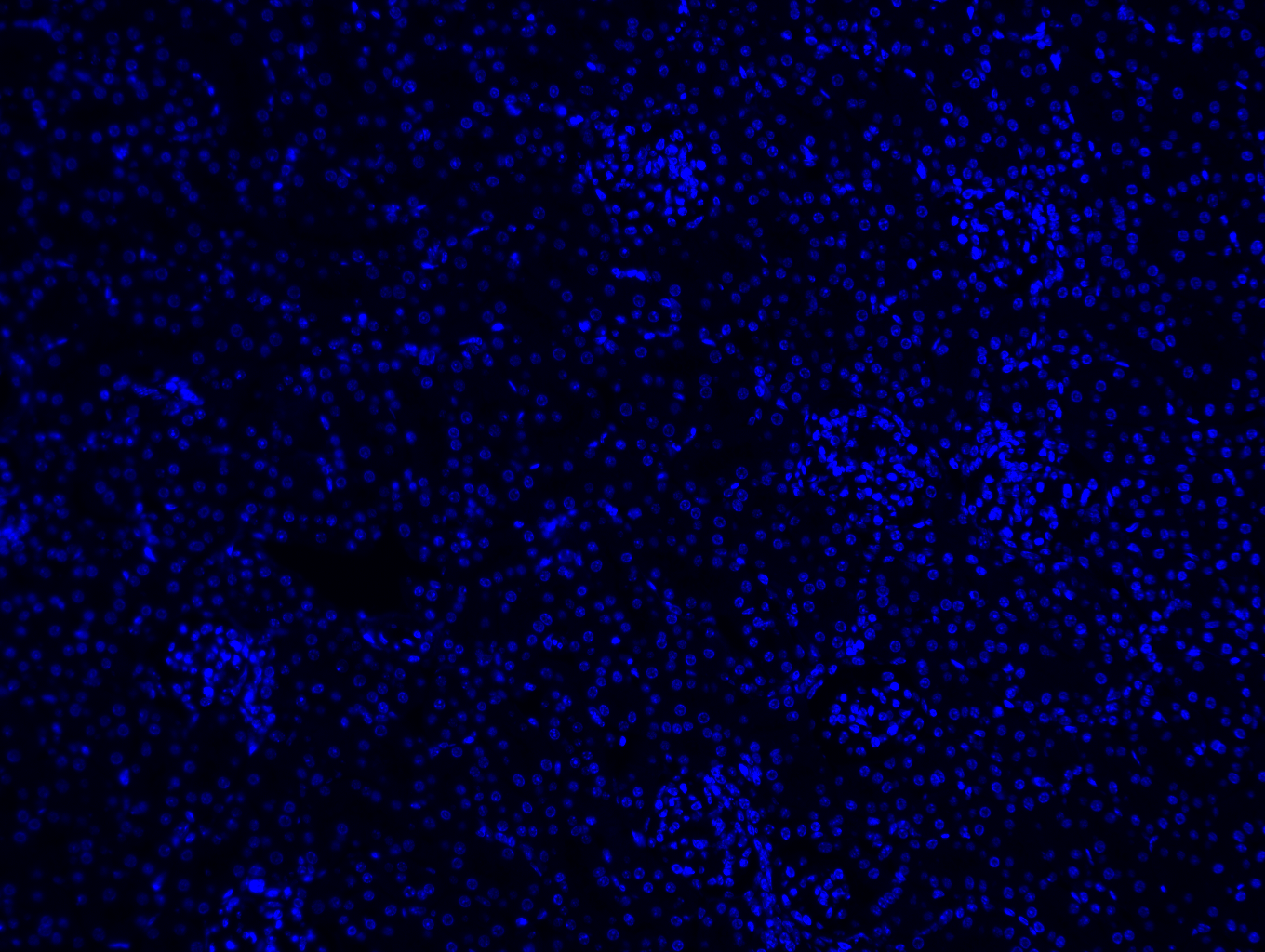

Supplement: Supplementary file 8 — Source Data for Figure 2 [file EMMM-12-e11021-s006.zip › SourceData_Fig2/Fig2B_mutant_DAPI.TIF]

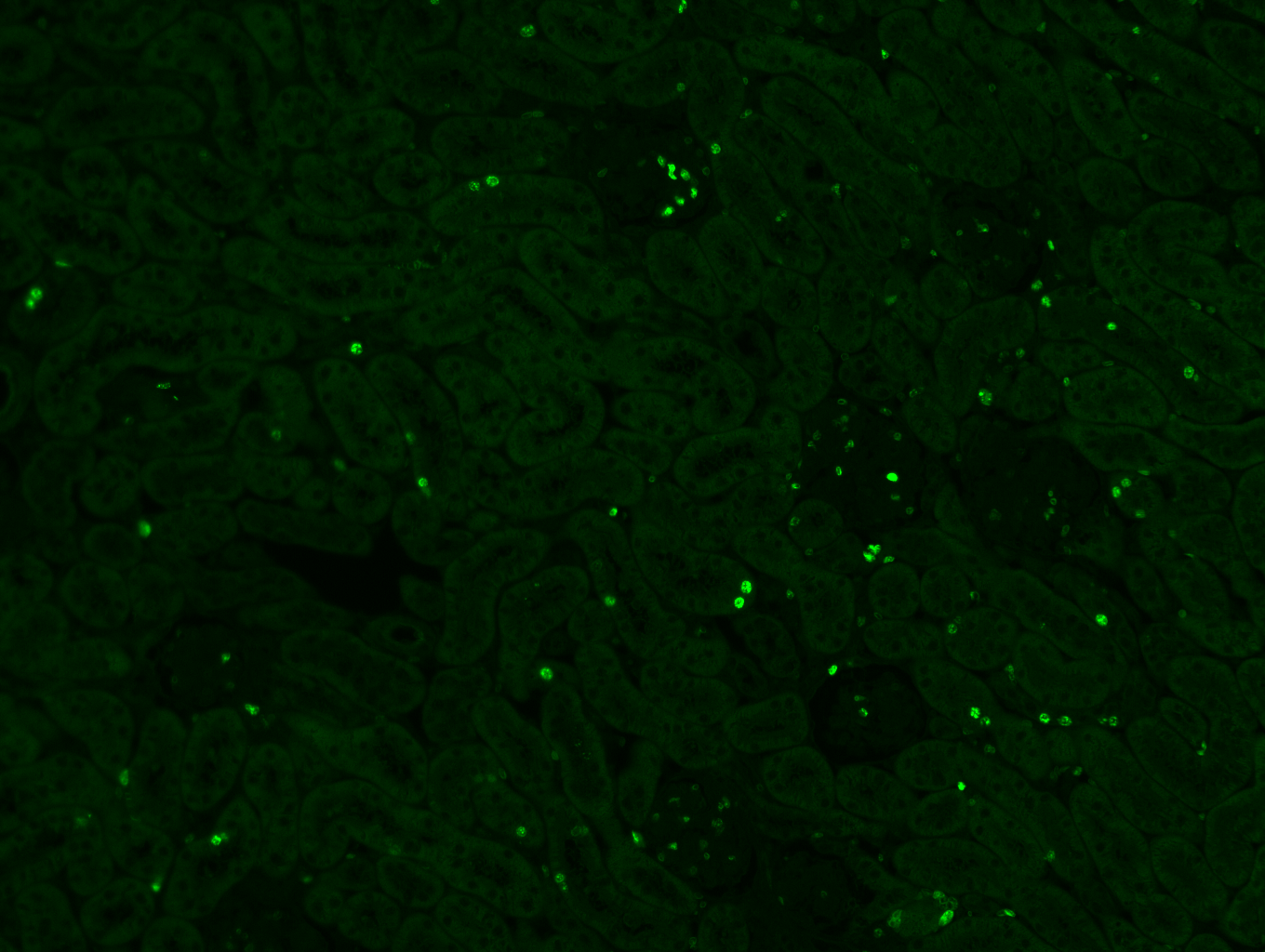

Supplement: Supplementary file 8 — Source Data for Figure 2 [file EMMM-12-e11021-s006.zip › SourceData_Fig2/Fig2B_mutant_Ki67.TIF]

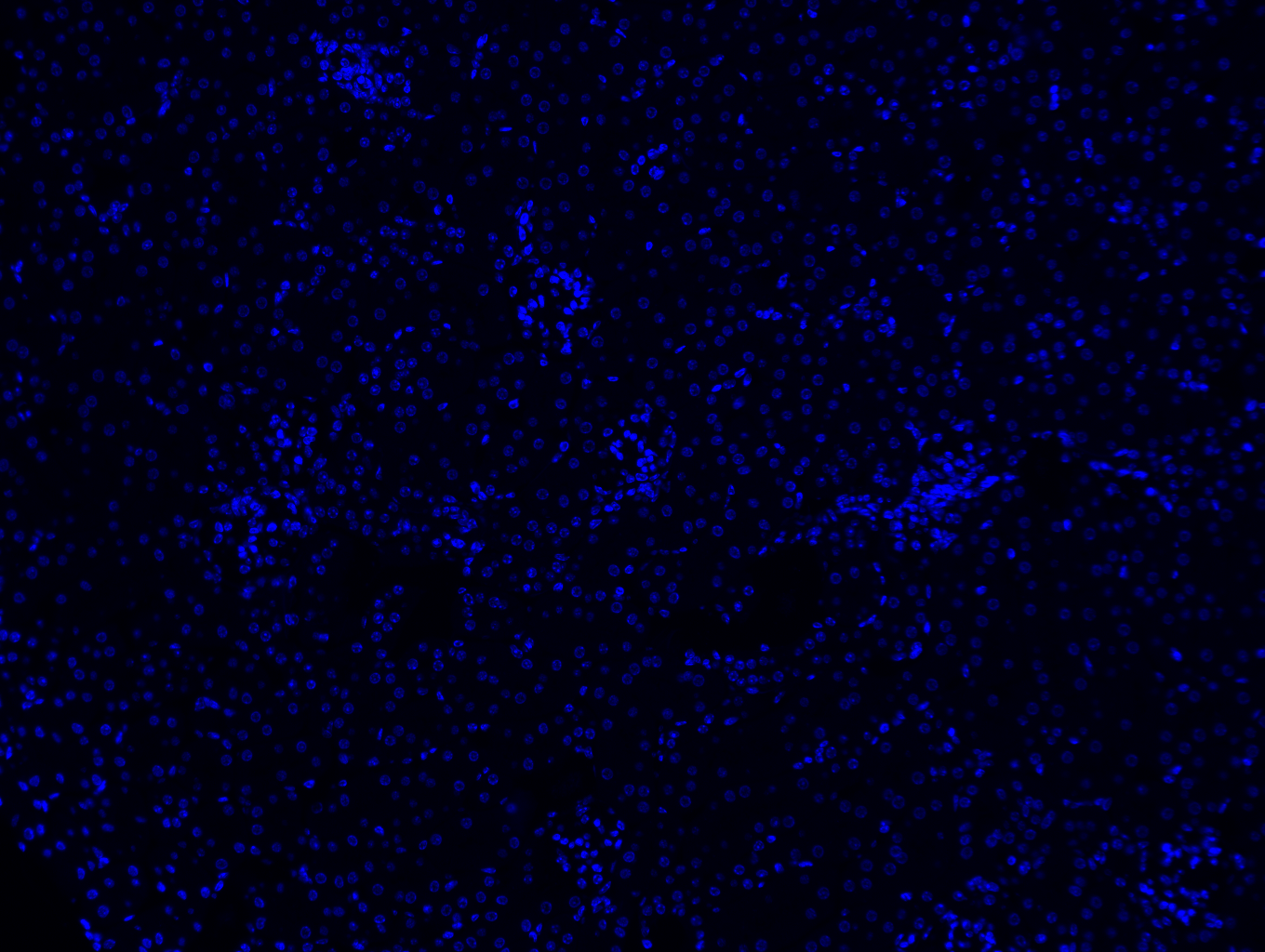

Supplement: Supplementary file 8 — Source Data for Figure 2 [file EMMM-12-e11021-s006.zip › SourceData_Fig2/Fig2B_wt_DAPI.TIF]

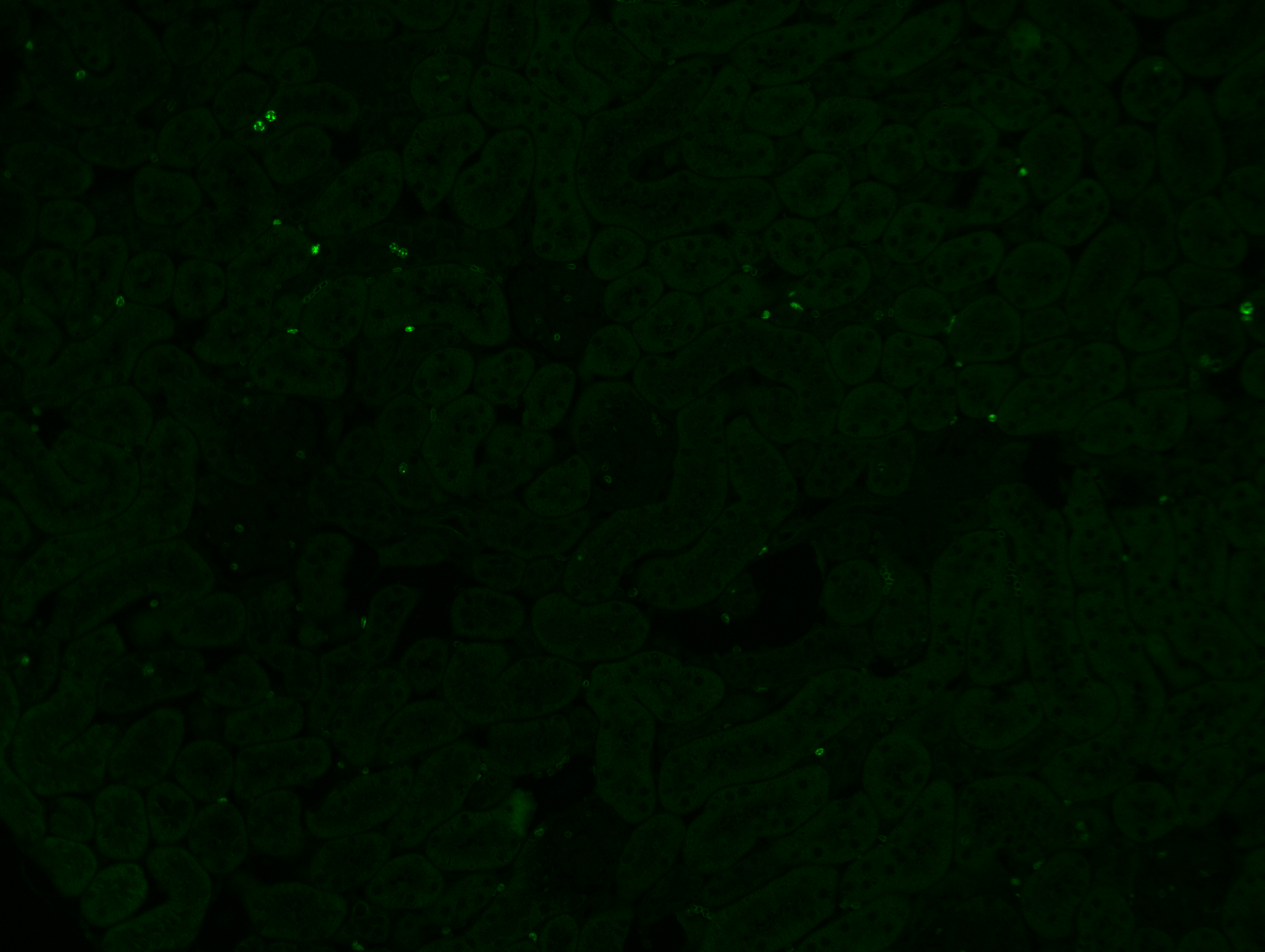

Supplement: Supplementary file 8 — Source Data for Figure 2 [file EMMM-12-e11021-s006.zip › SourceData_Fig2/Fig2B_wt_Ki67.TIF]

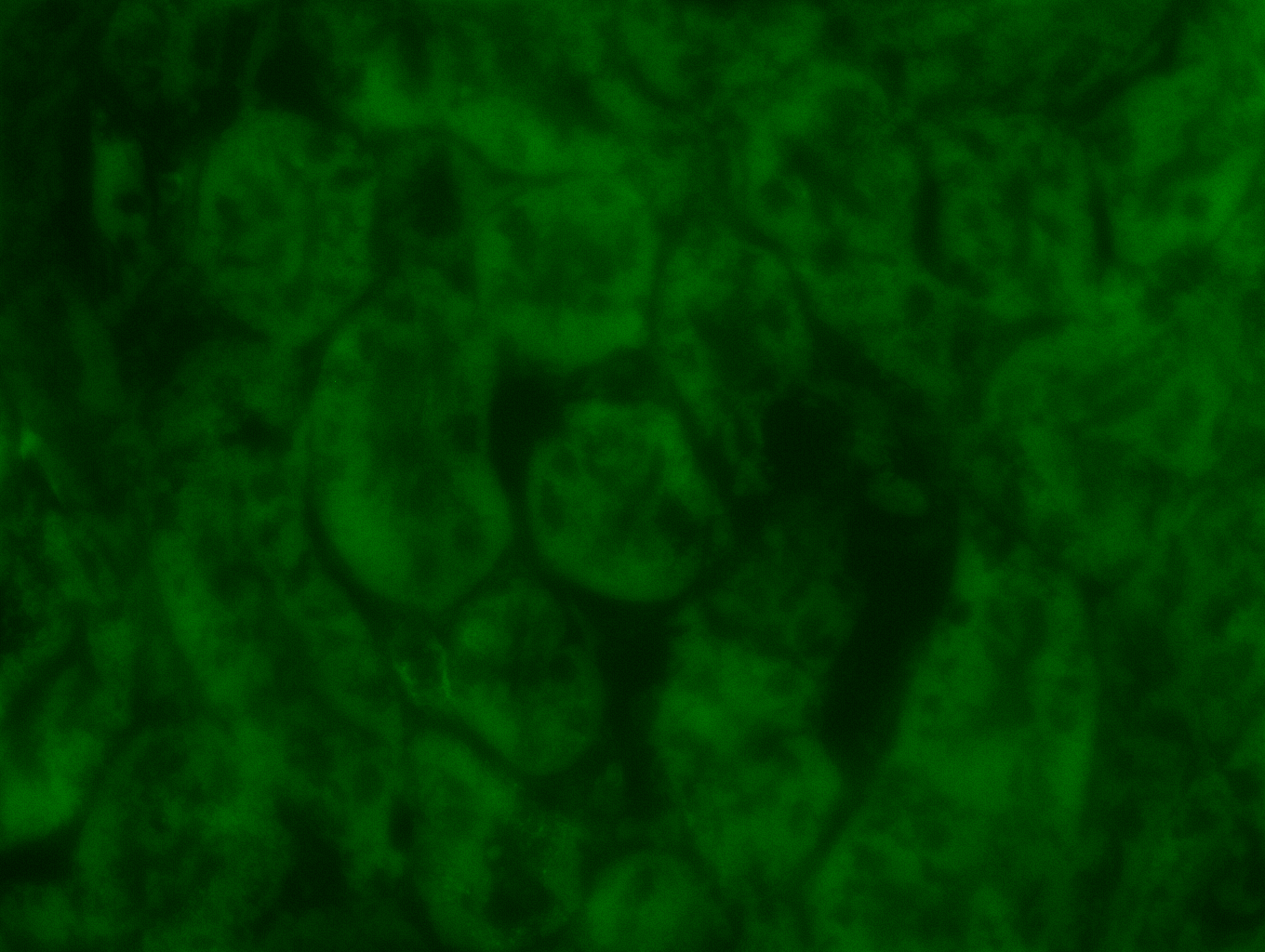

Supplement: Supplementary file 8 — Source Data for Figure 2 [file EMMM-12-e11021-s006.zip › SourceData_Fig2/Fig2F_Cortex_mutant_autofluorescence.tif]

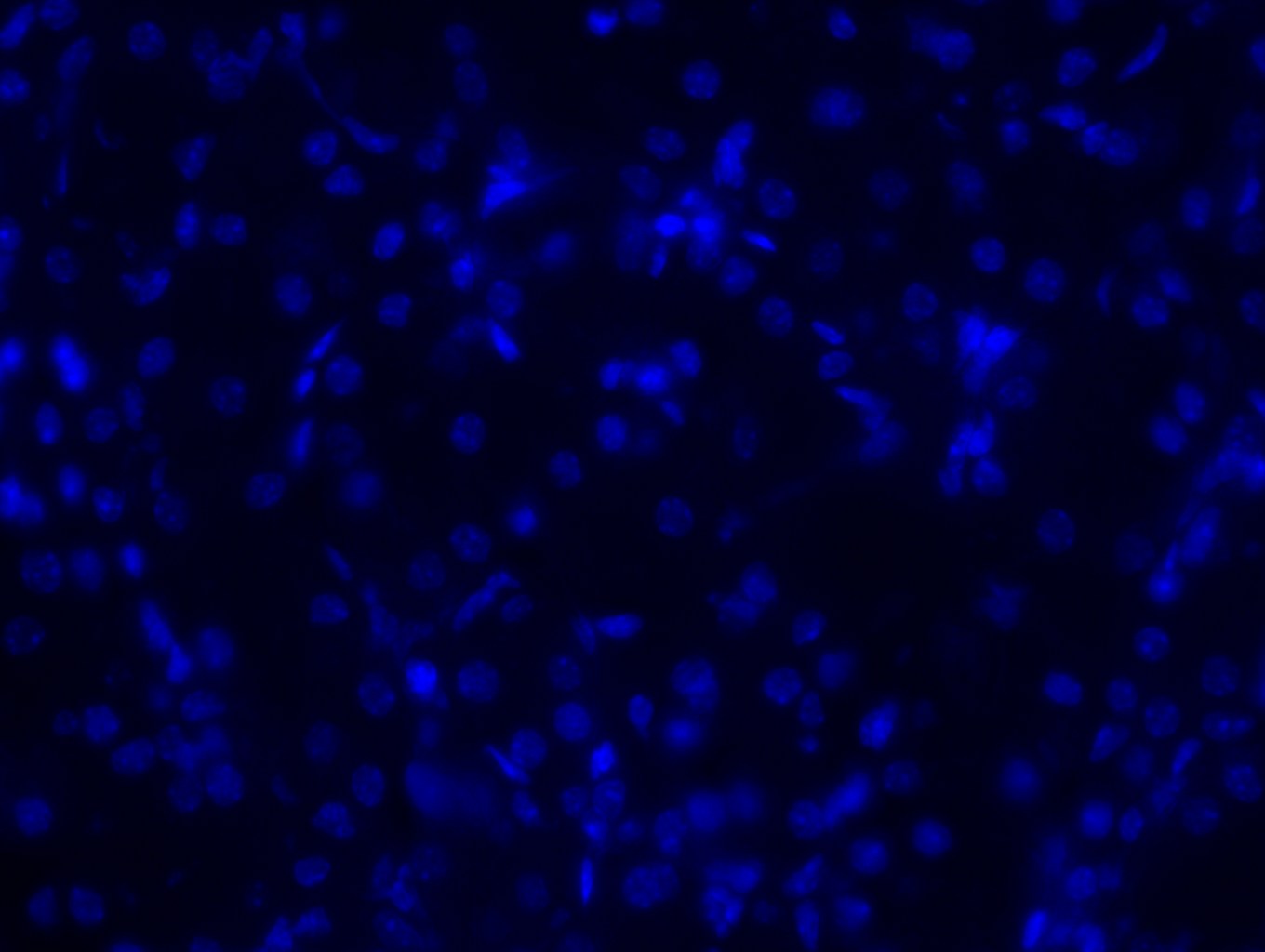

Supplement: Supplementary file 8 — Source Data for Figure 2 [file EMMM-12-e11021-s006.zip › SourceData_Fig2/Fig2F_Cortex_mutant_DAPI.tif]

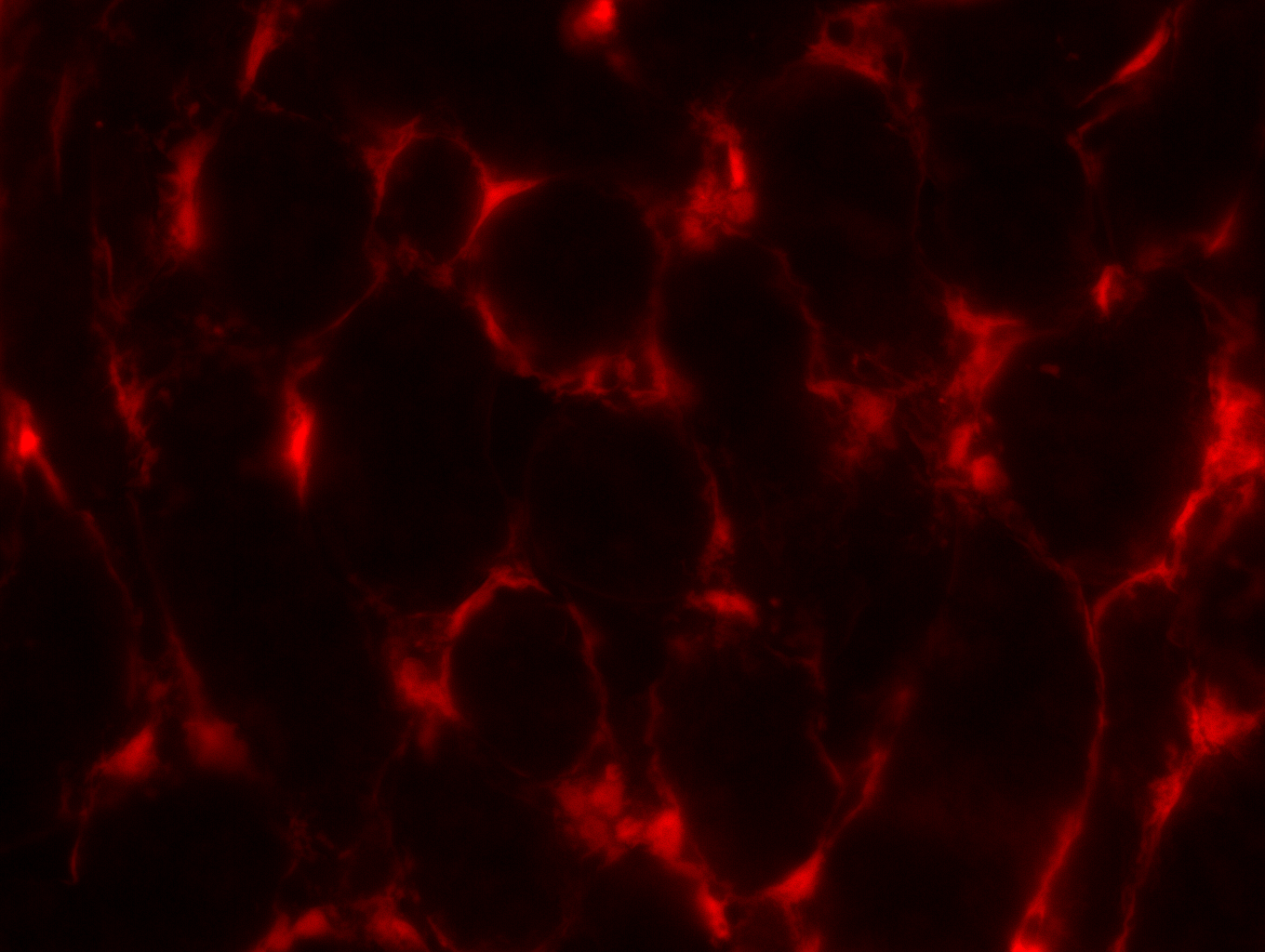

Supplement: Supplementary file 8 — Source Data for Figure 2 [file EMMM-12-e11021-s006.zip › SourceData_Fig2/Fig2F_Cortex_mutant_Tomato.tif]

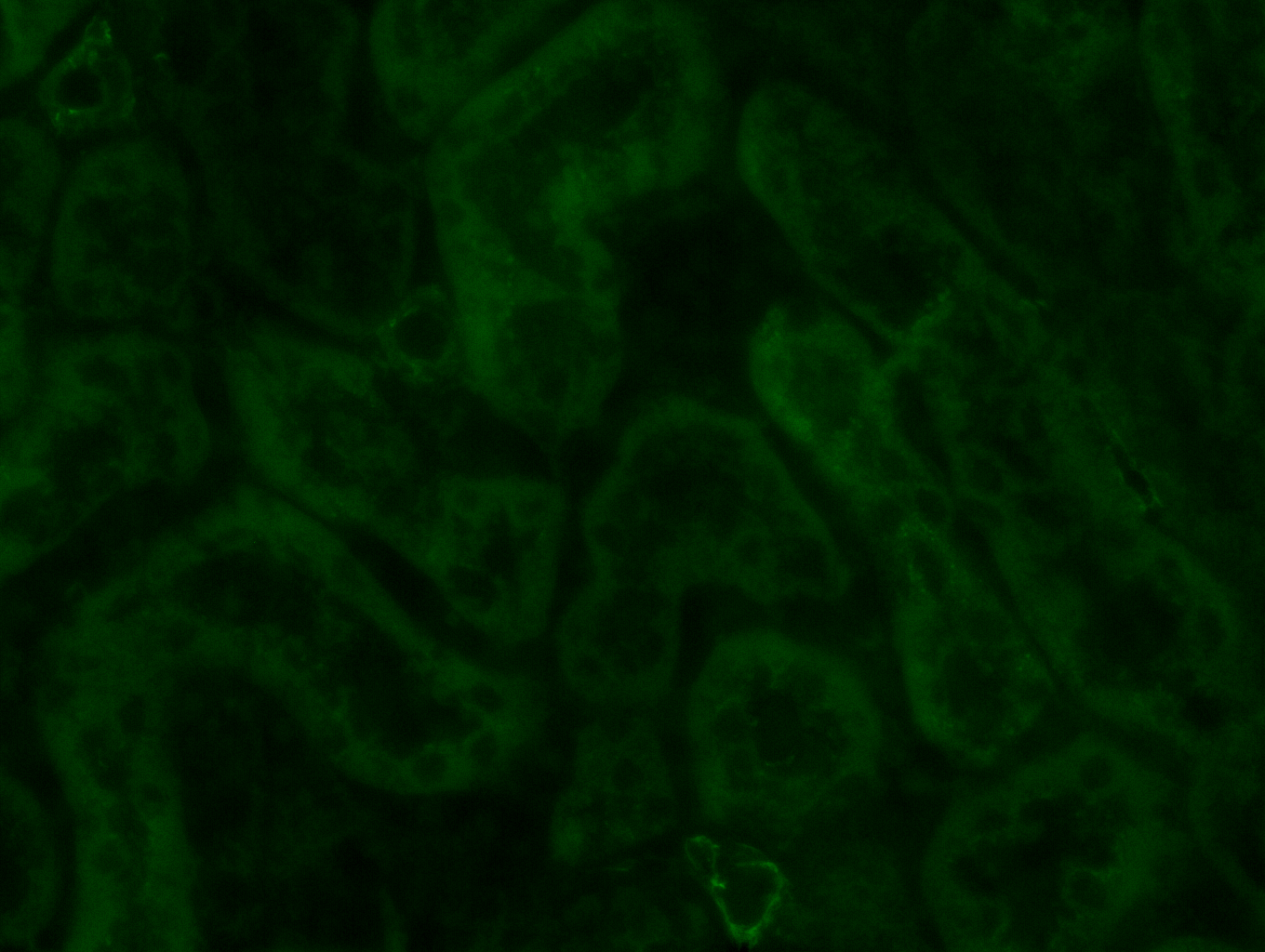

Supplement: Supplementary file 8 — Source Data for Figure 2 [file EMMM-12-e11021-s006.zip › SourceData_Fig2/Fig2F_Cortex_wt_autofluorescence.tif]

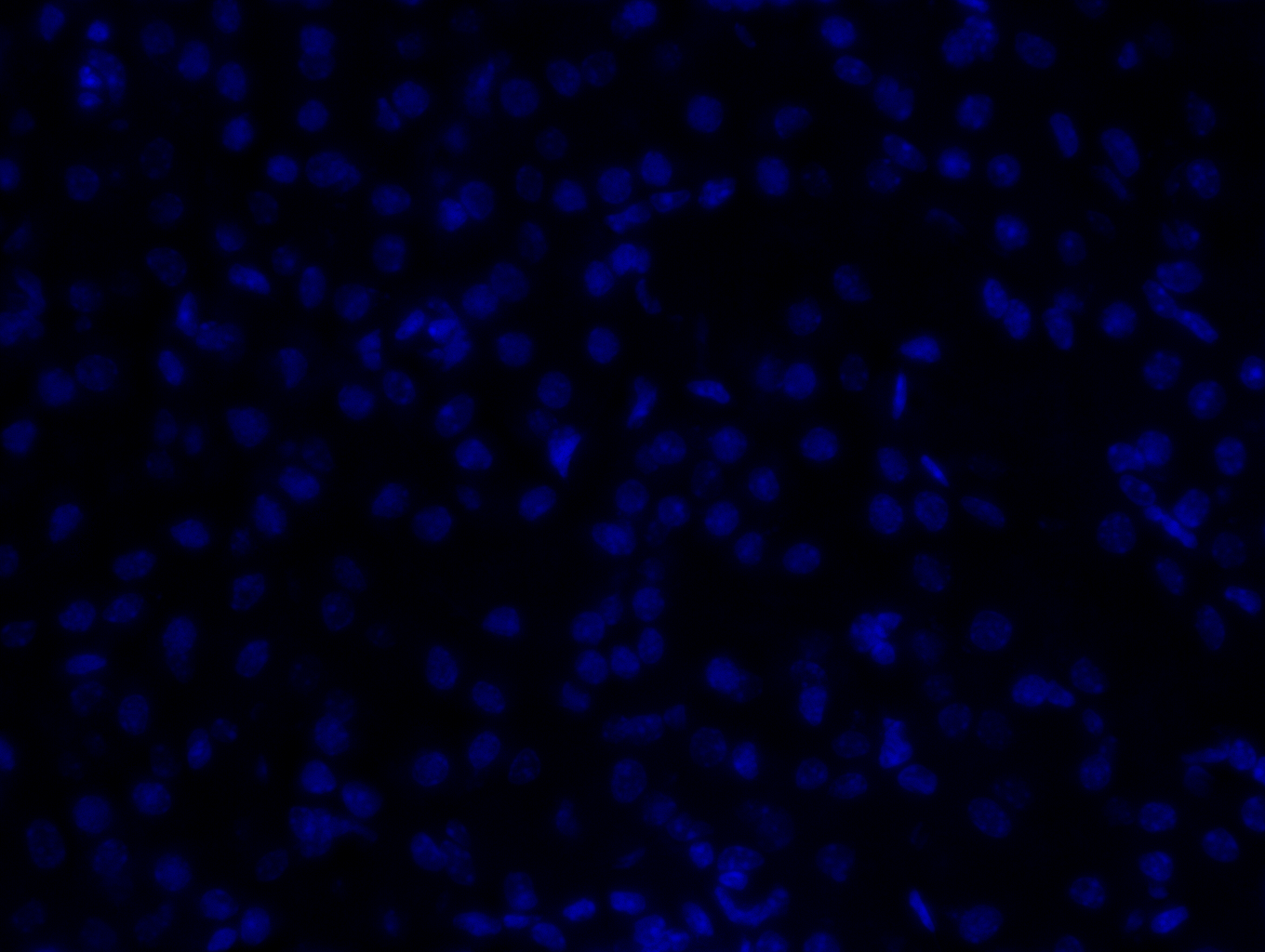

Supplement: Supplementary file 8 — Source Data for Figure 2 [file EMMM-12-e11021-s006.zip › SourceData_Fig2/Fig2F_Cortex_wt_DAPI.tif]

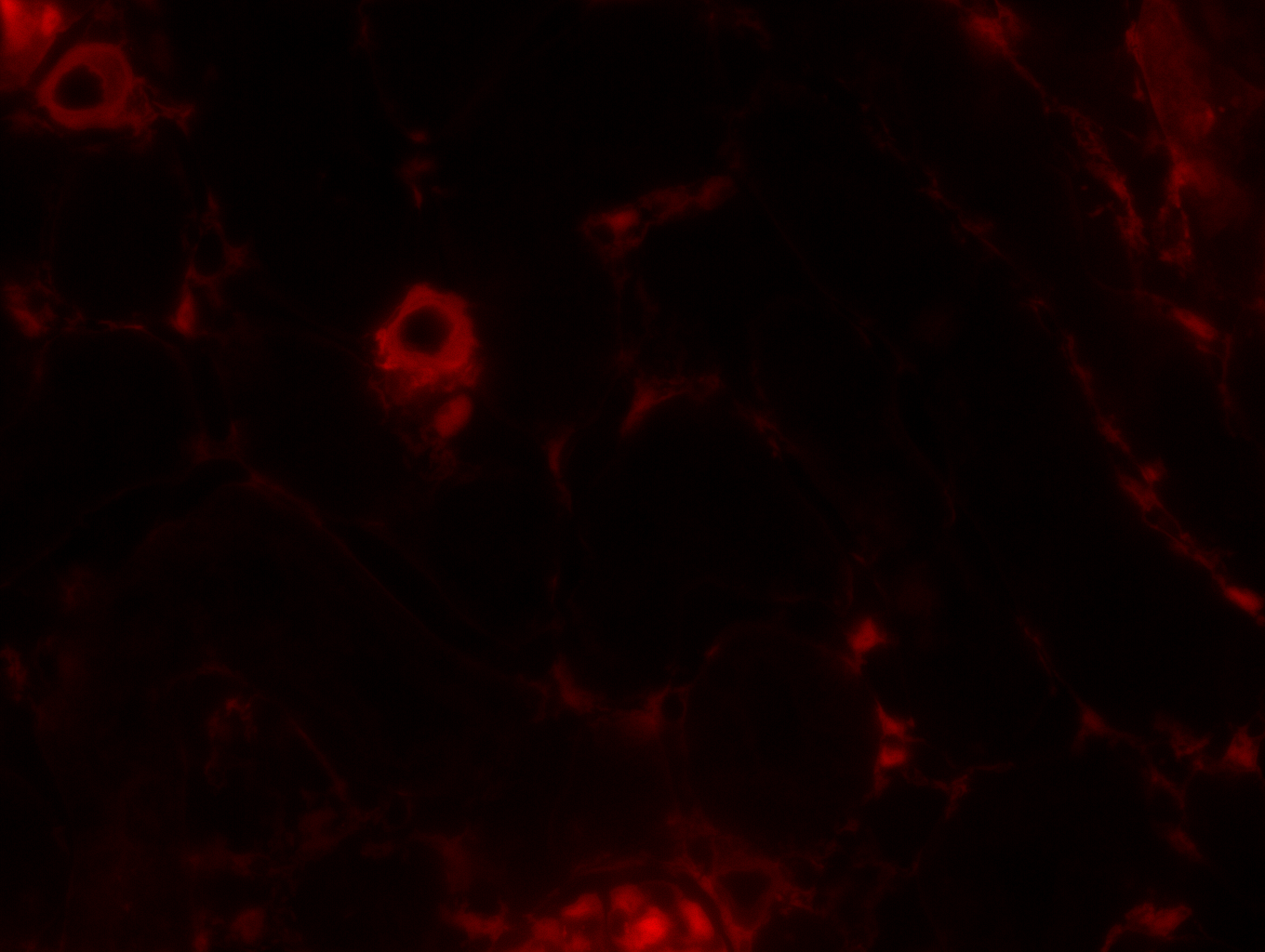

Supplement: Supplementary file 8 — Source Data for Figure 2 [file EMMM-12-e11021-s006.zip › SourceData_Fig2/Fig2F_Cortex_wt_Tomato.tif]

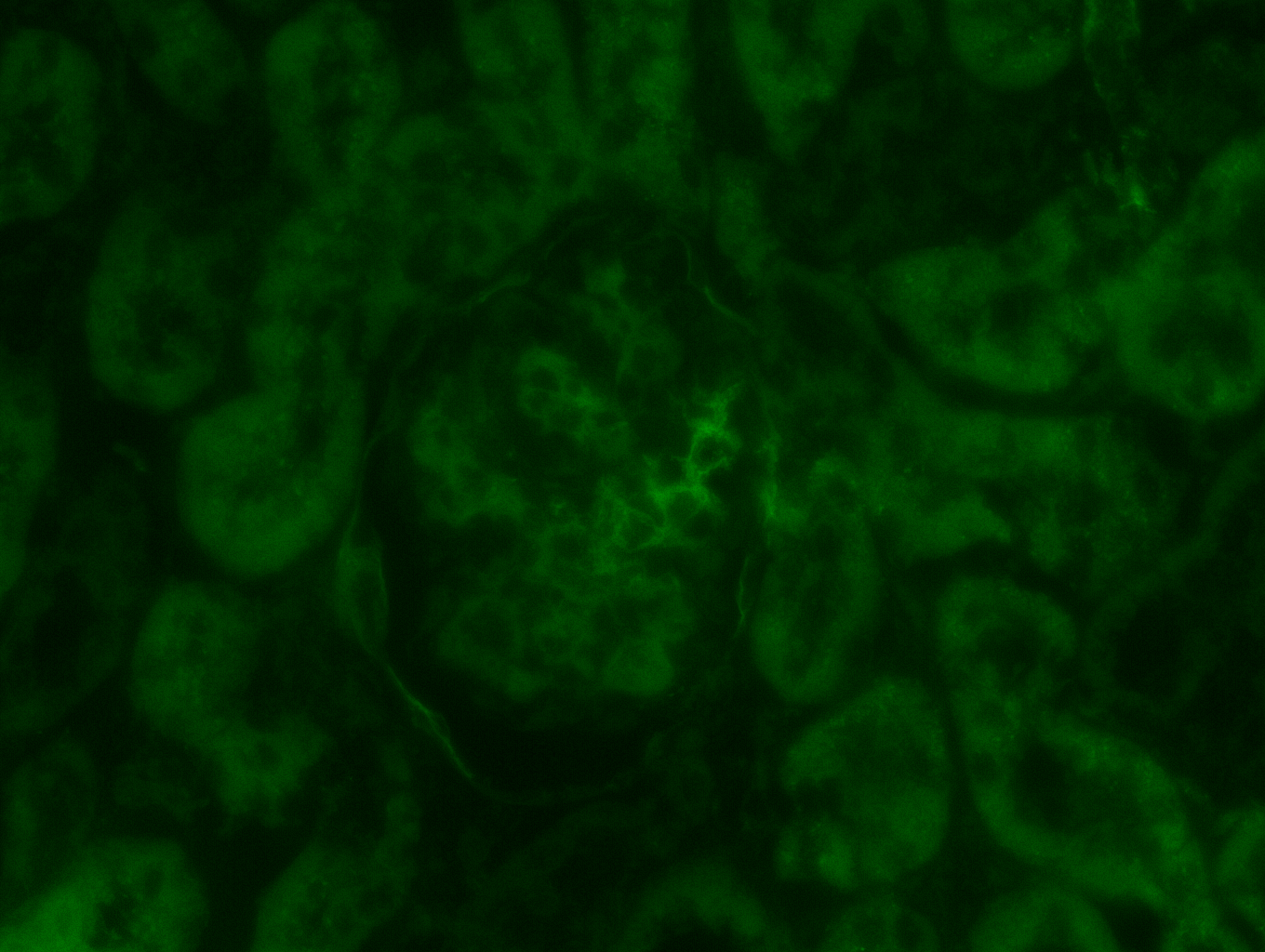

Supplement: Supplementary file 8 — Source Data for Figure 2 [file EMMM-12-e11021-s006.zip › SourceData_Fig2/Fig2F_Glomerulus_mutant_autofluorescence.tif]

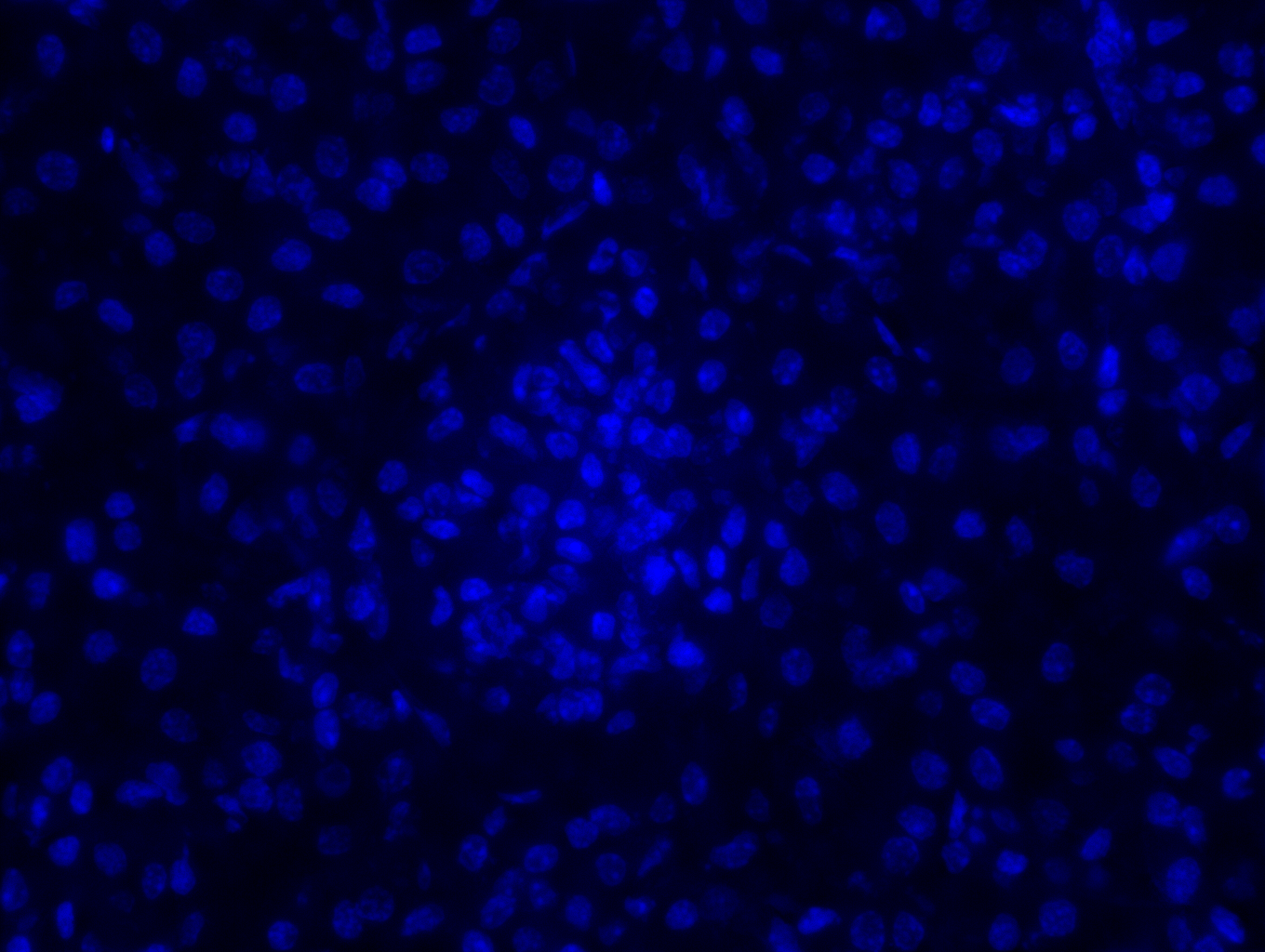

Supplement: Supplementary file 8 — Source Data for Figure 2 [file EMMM-12-e11021-s006.zip › SourceData_Fig2/Fig2F_Glomerulus_mutant_DAPI.tif]

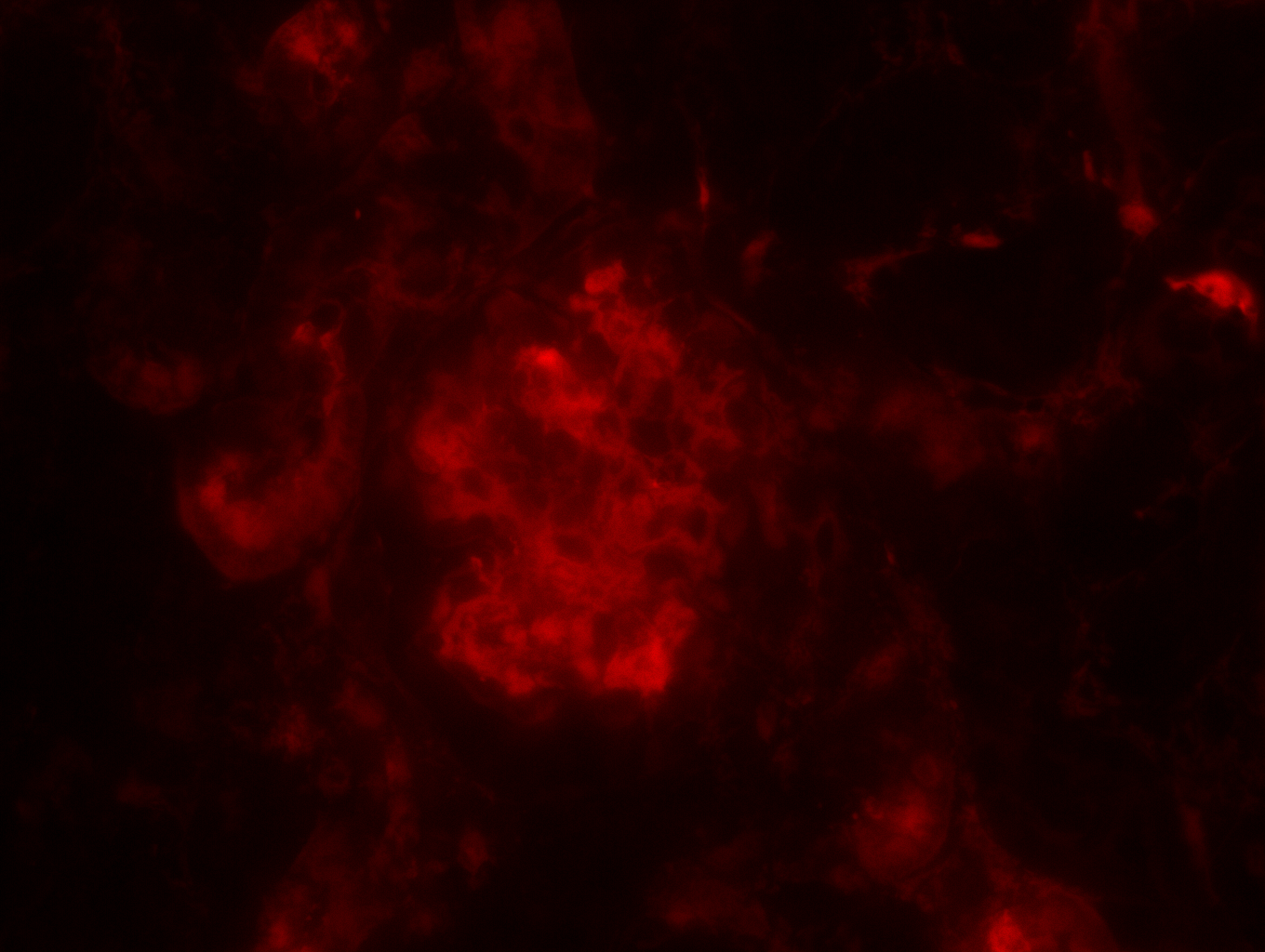

Supplement: Supplementary file 8 — Source Data for Figure 2 [file EMMM-12-e11021-s006.zip › SourceData_Fig2/Fig2F_Glomerulus_mutant_Tomato.tif]

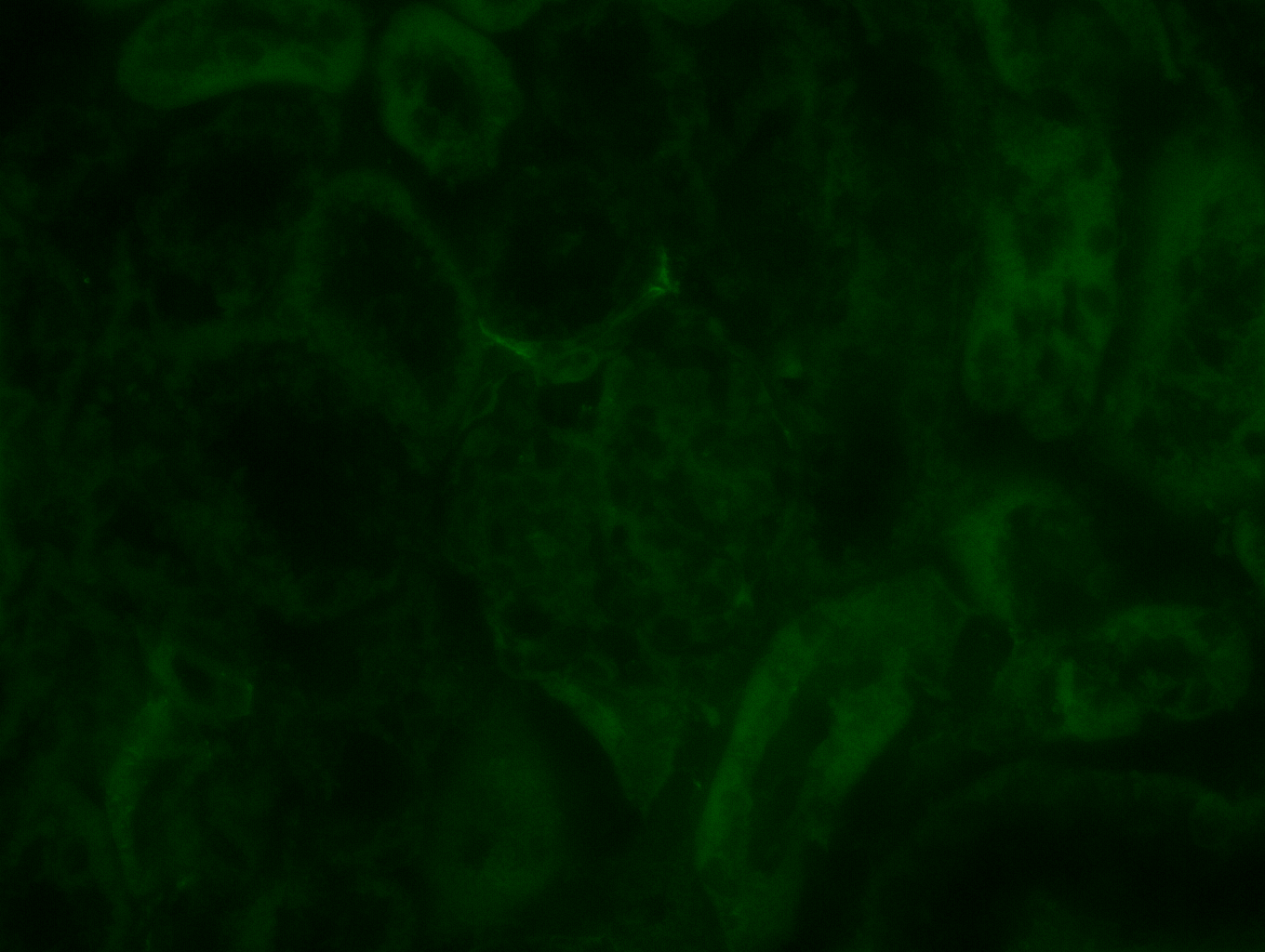

Supplement: Supplementary file 8 — Source Data for Figure 2 [file EMMM-12-e11021-s006.zip › SourceData_Fig2/Fig2F_Glomerulus_wt_autofluorescence.tif]

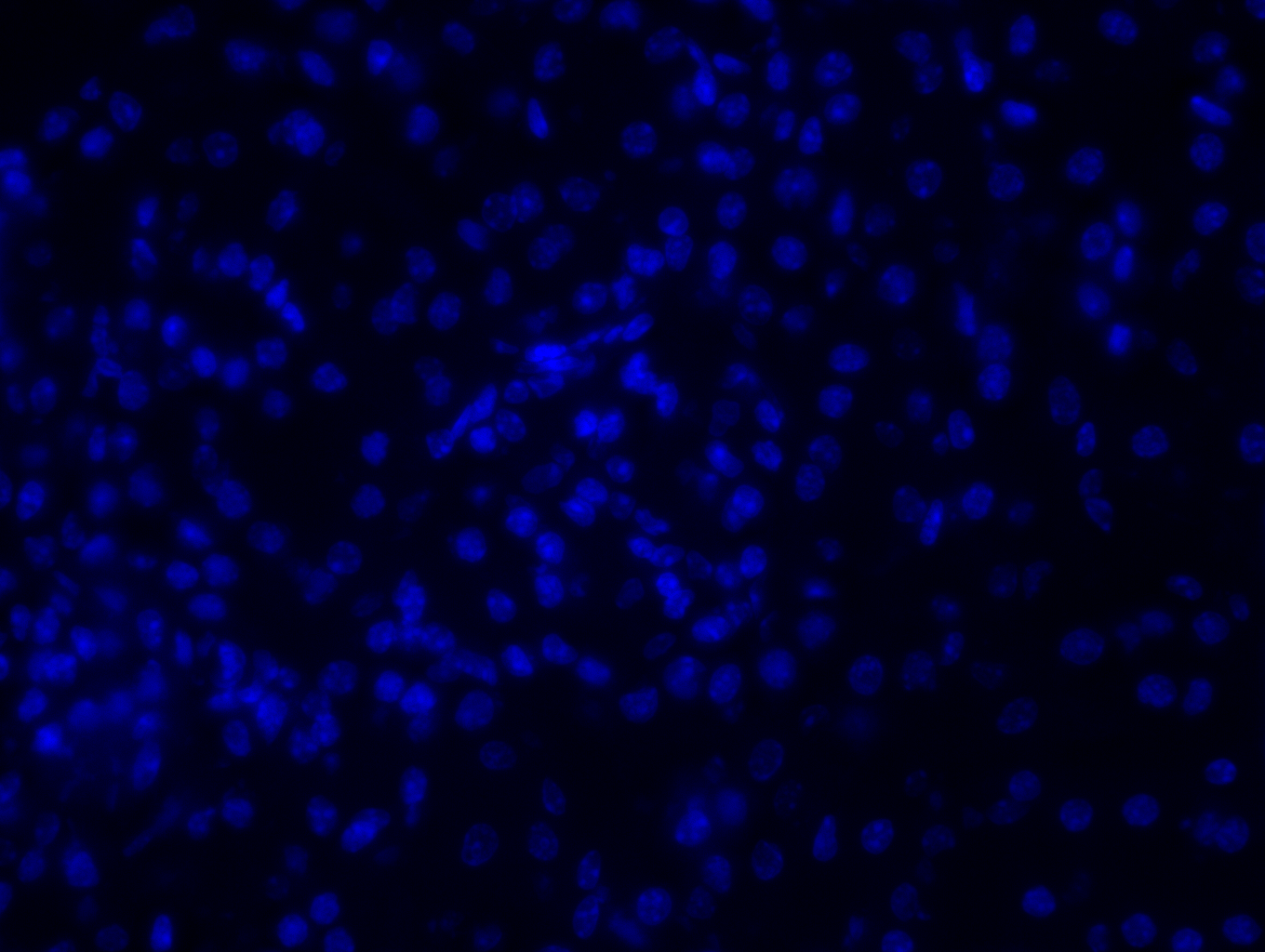

Supplement: Supplementary file 8 — Source Data for Figure 2 [file EMMM-12-e11021-s006.zip › SourceData_Fig2/Fig2F_Glomerulus_wt_DAPI.tif]

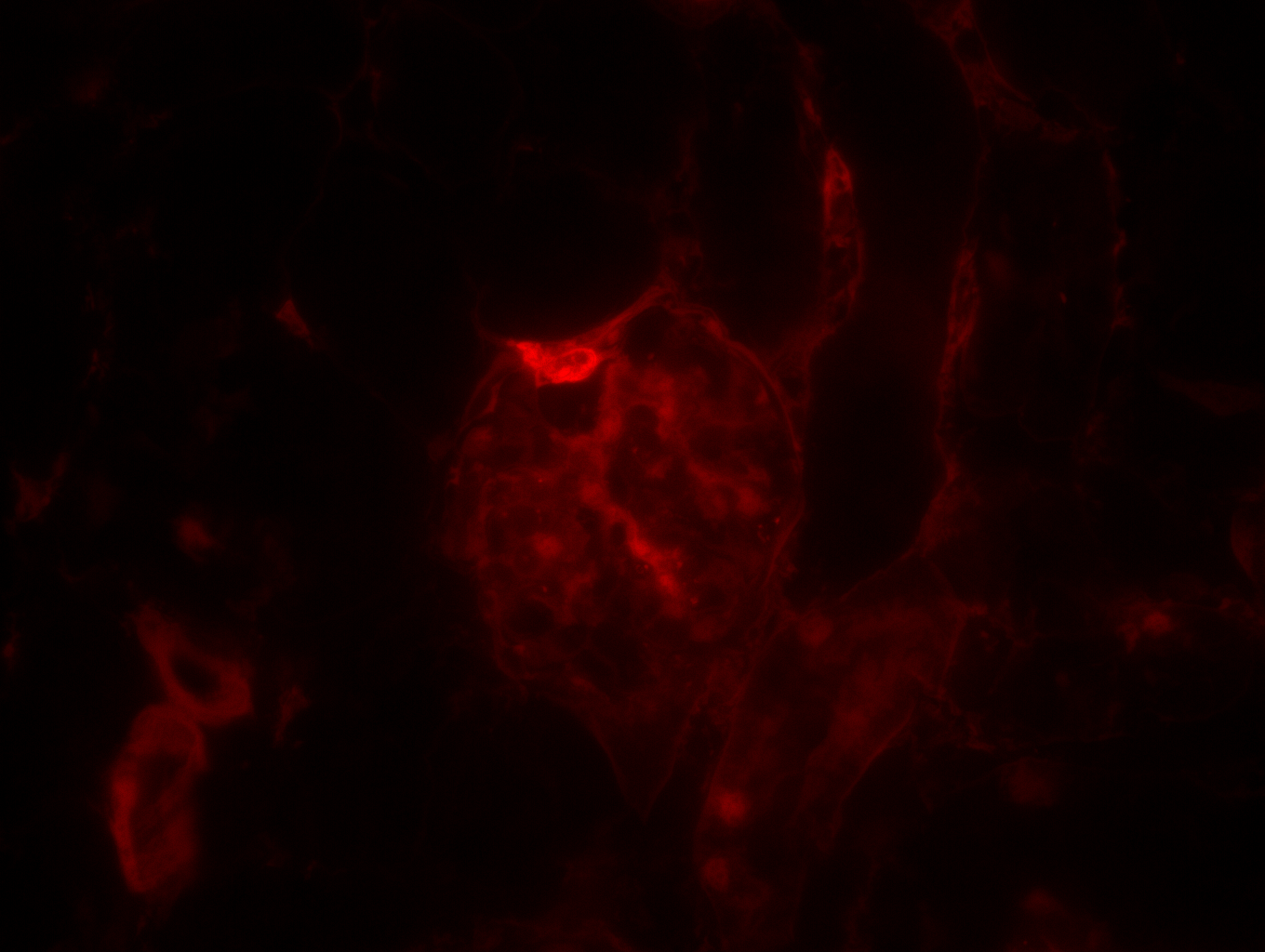

Supplement: Supplementary file 8 — Source Data for Figure 2 [file EMMM-12-e11021-s006.zip › SourceData_Fig2/Fig2F_Glomerulus_wt_Tomato.tif]

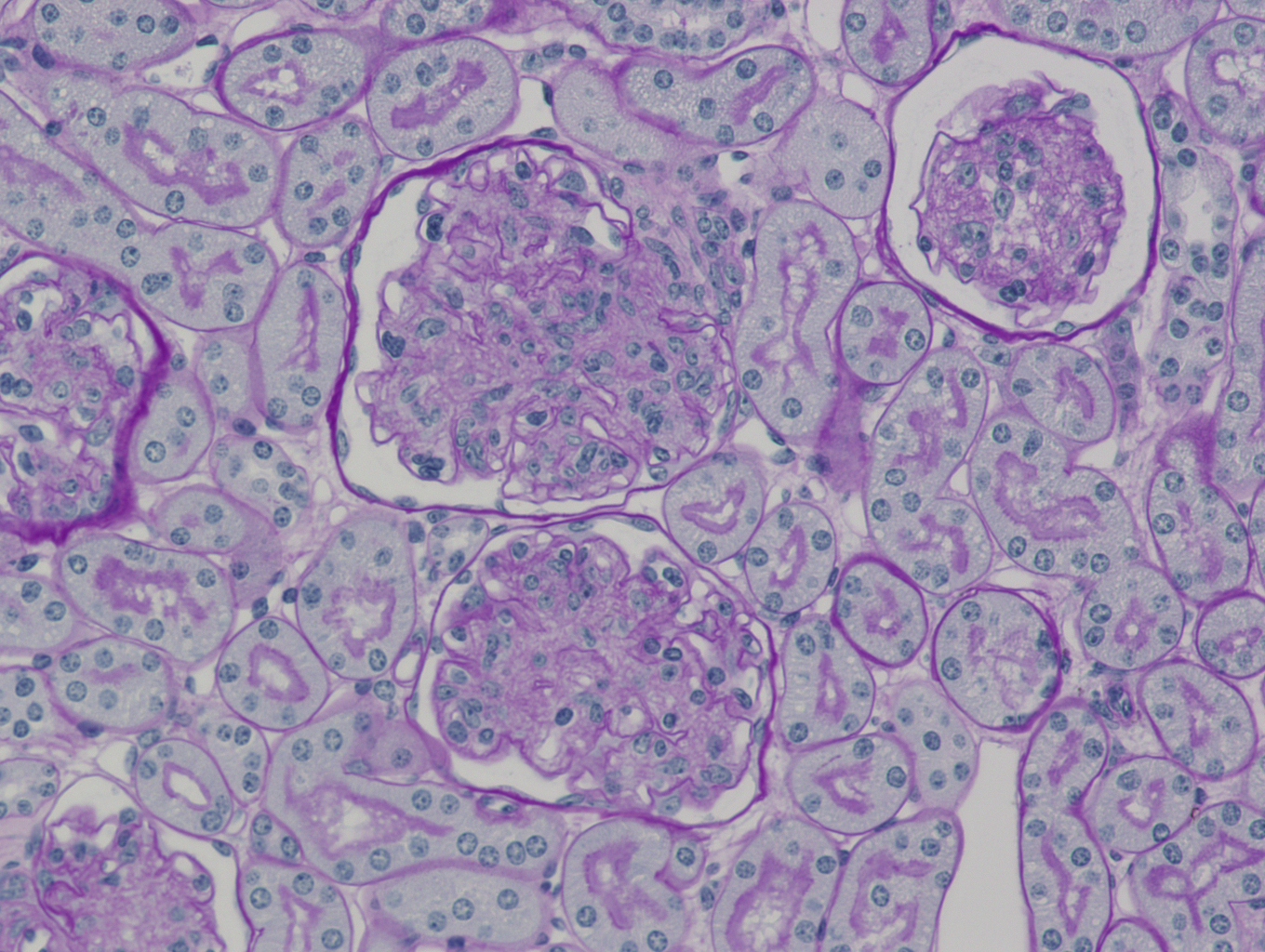

Supplement: Supplementary file 9 — Source Data for Figure 3 [file EMMM-12-e11021-s007.zip › SourceData_Fig3/Fig3A_35weeks.tif]

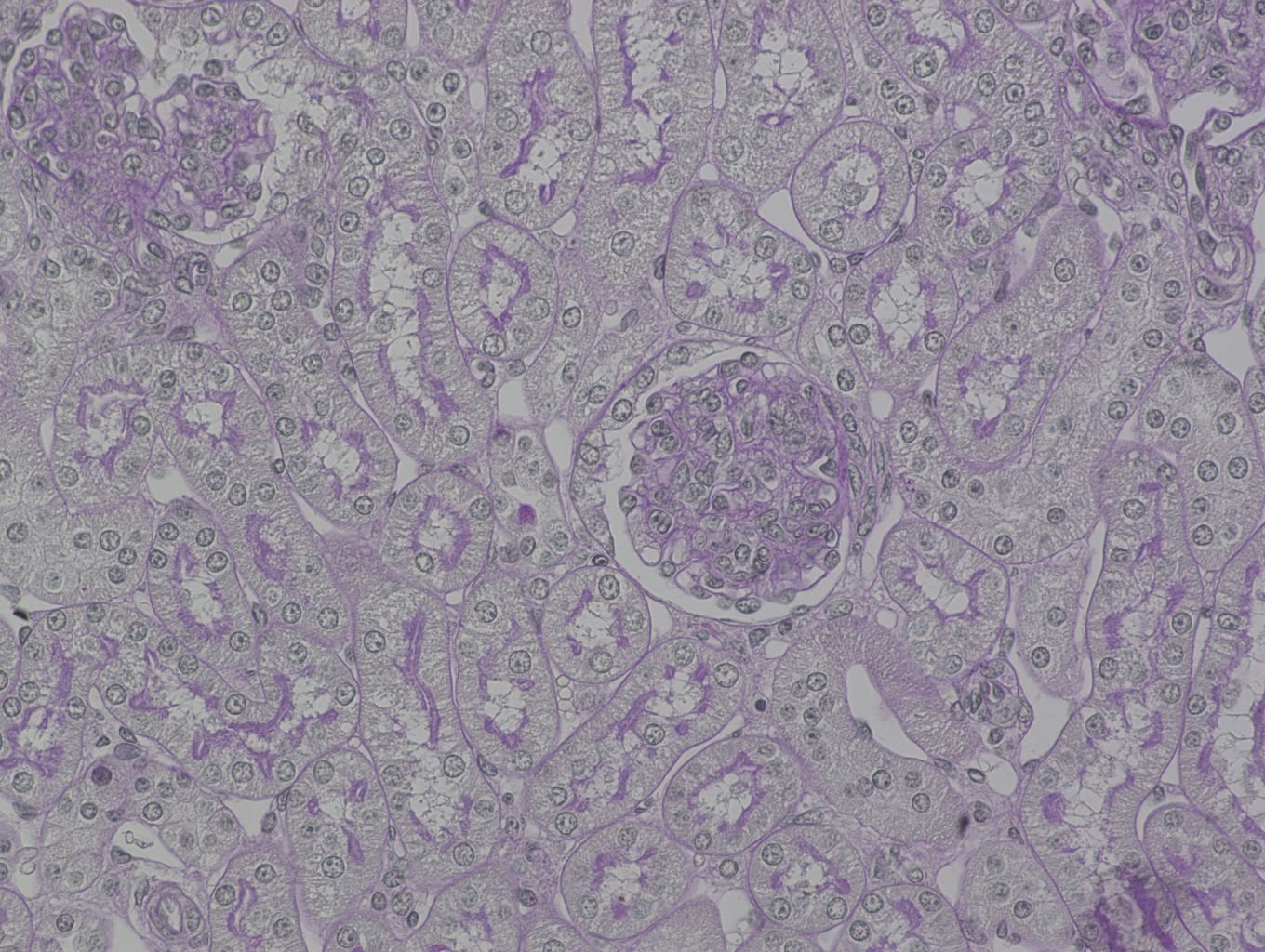

Supplement: Supplementary file 9 — Source Data for Figure 3 [file EMMM-12-e11021-s007.zip › SourceData_Fig3/Fig3A_6weeks.tif]

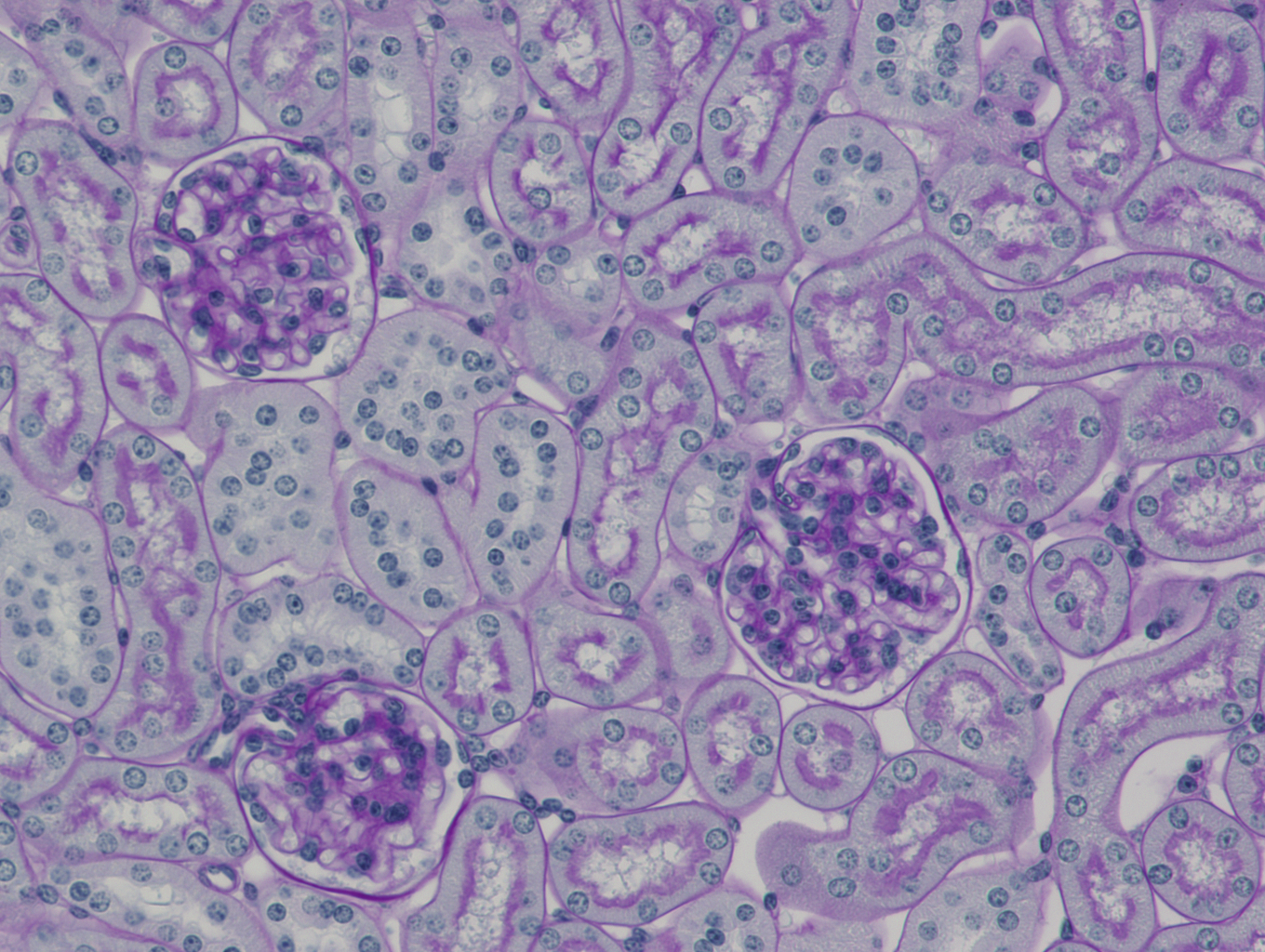

Supplement: Supplementary file 9 — Source Data for Figure 3 [file EMMM-12-e11021-s007.zip › SourceData_Fig3/Fig3A_wt.tif]

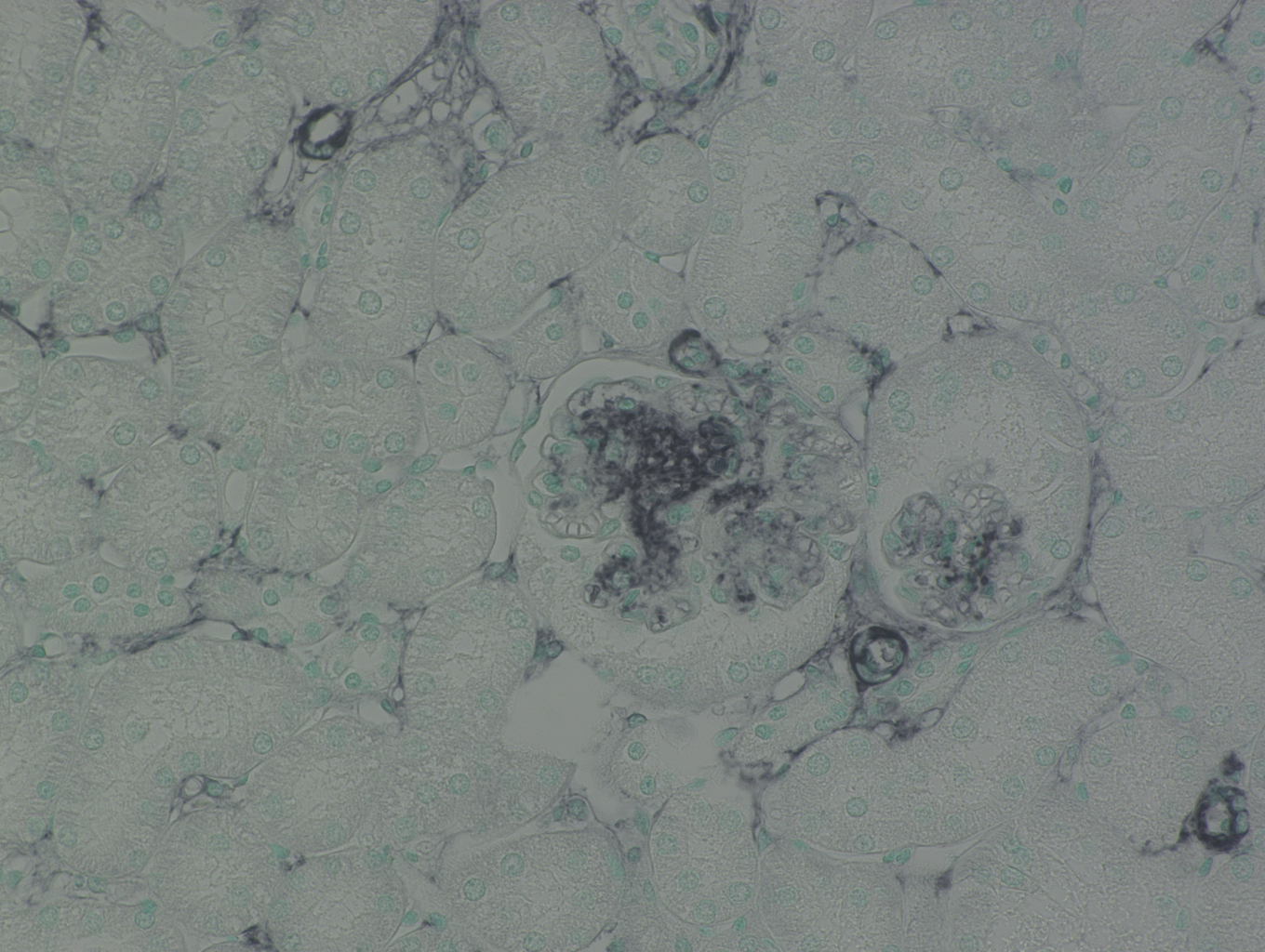

Supplement: Supplementary file 9 — Source Data for Figure 3 [file EMMM-12-e11021-s007.zip › SourceData_Fig3/Fig3C_35weeks.tif]

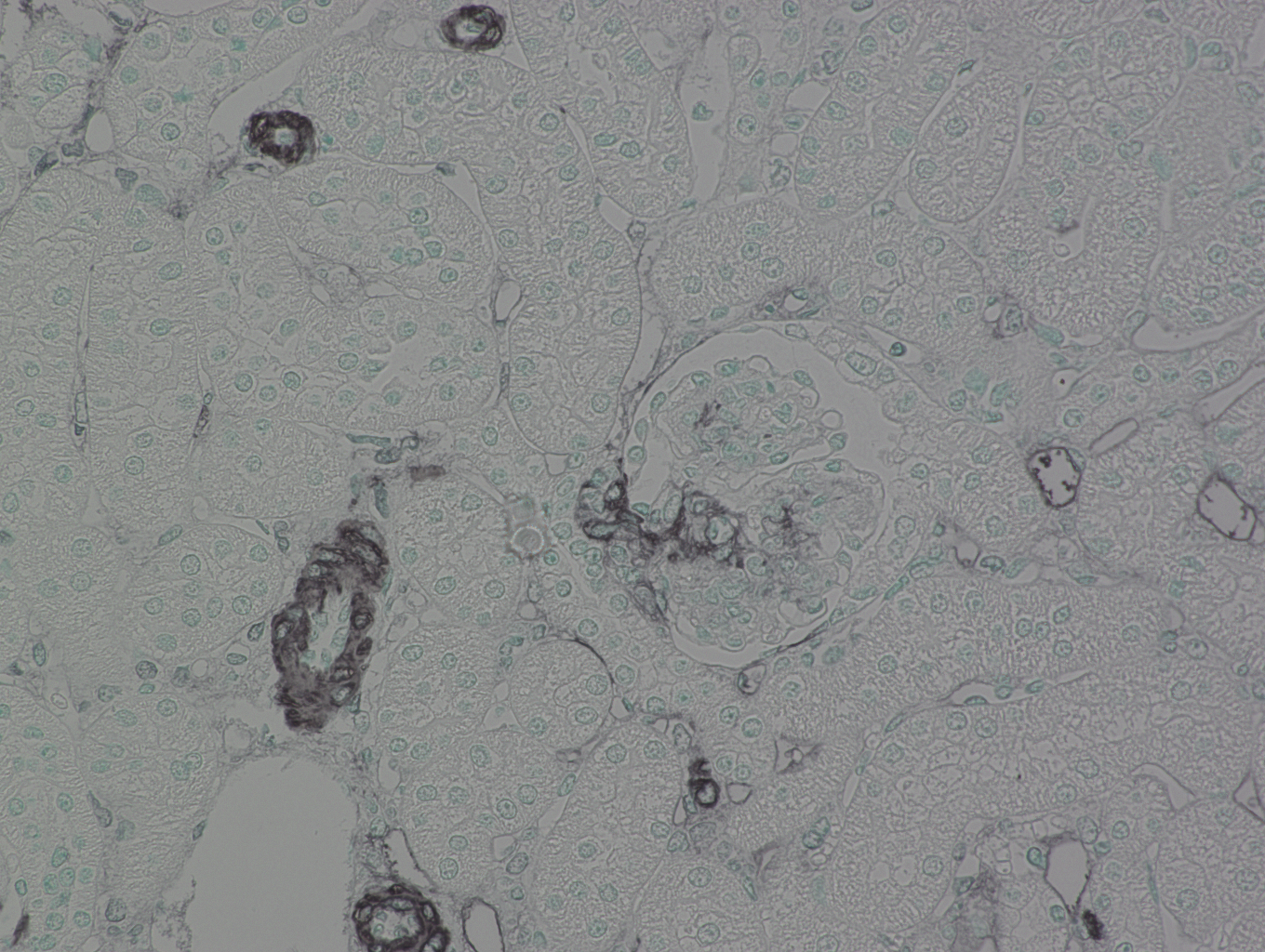

Supplement: Supplementary file 9 — Source Data for Figure 3 [file EMMM-12-e11021-s007.zip › SourceData_Fig3/Fig3C_6weeks.tif]

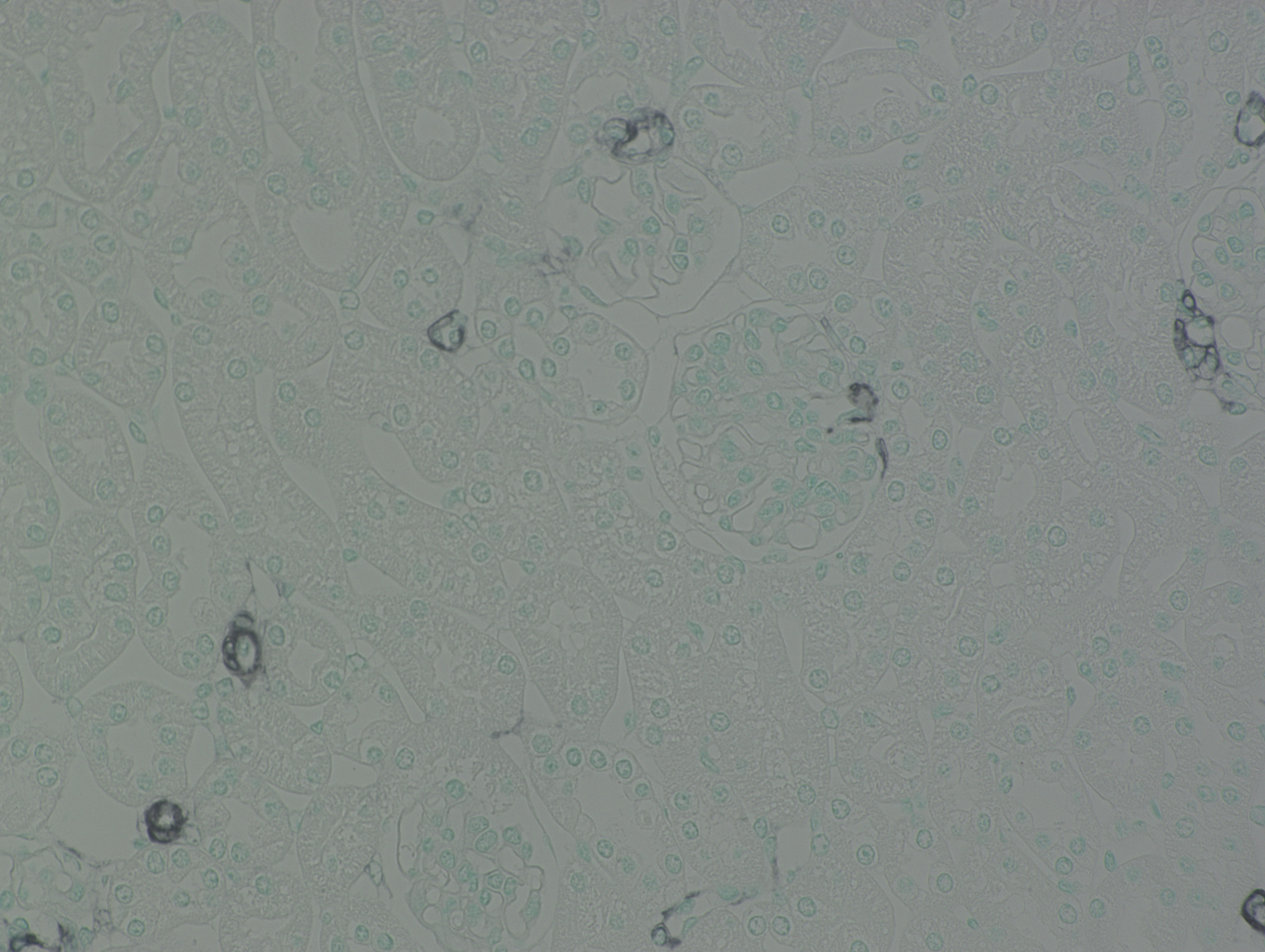

Supplement: Supplementary file 9 — Source Data for Figure 3 [file EMMM-12-e11021-s007.zip › SourceData_Fig3/Fig3C_wt.tif]

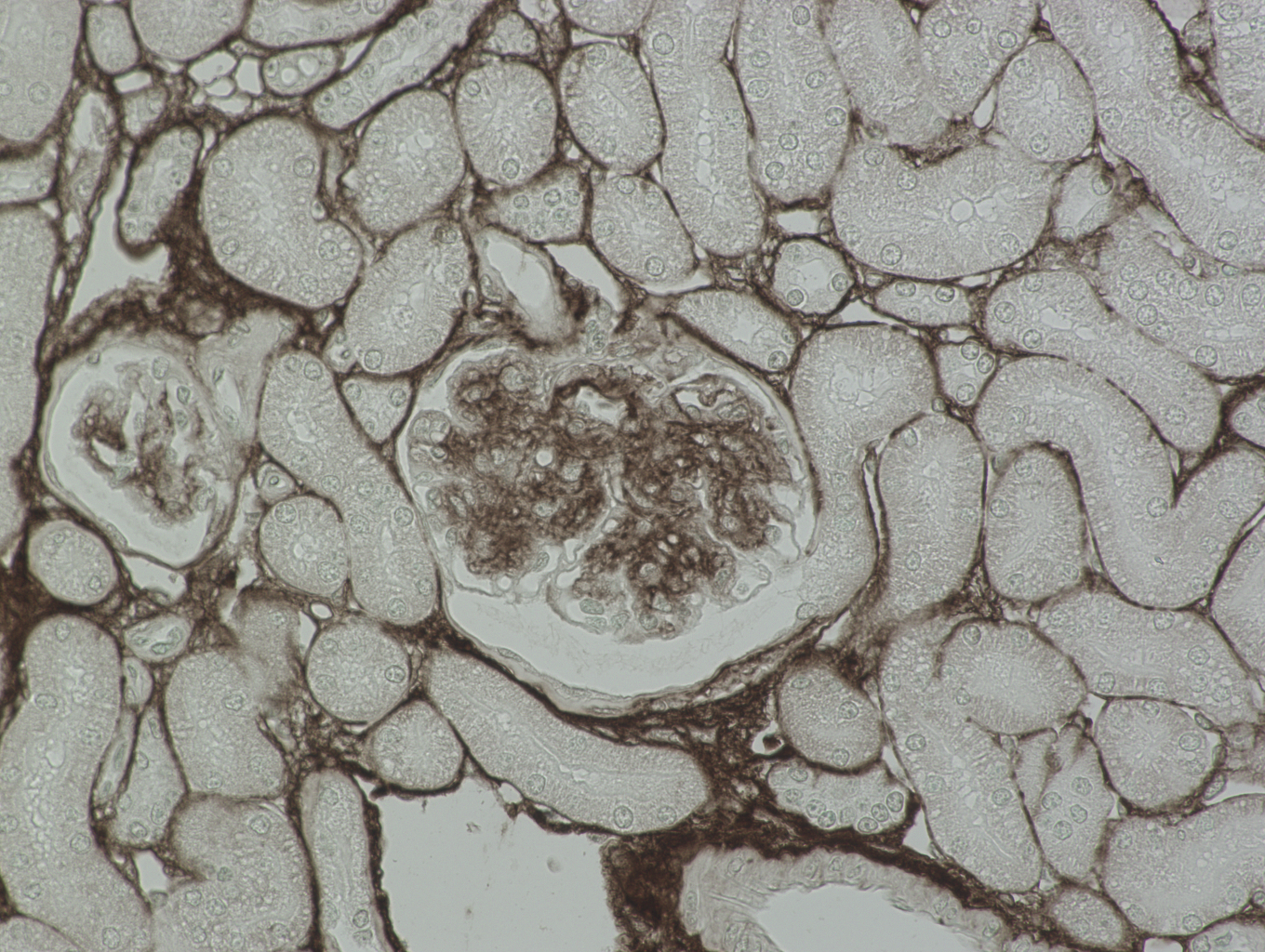

Supplement: Supplementary file 9 — Source Data for Figure 3 [file EMMM-12-e11021-s007.zip › SourceData_Fig3/Fig3E_35weeks.tif]

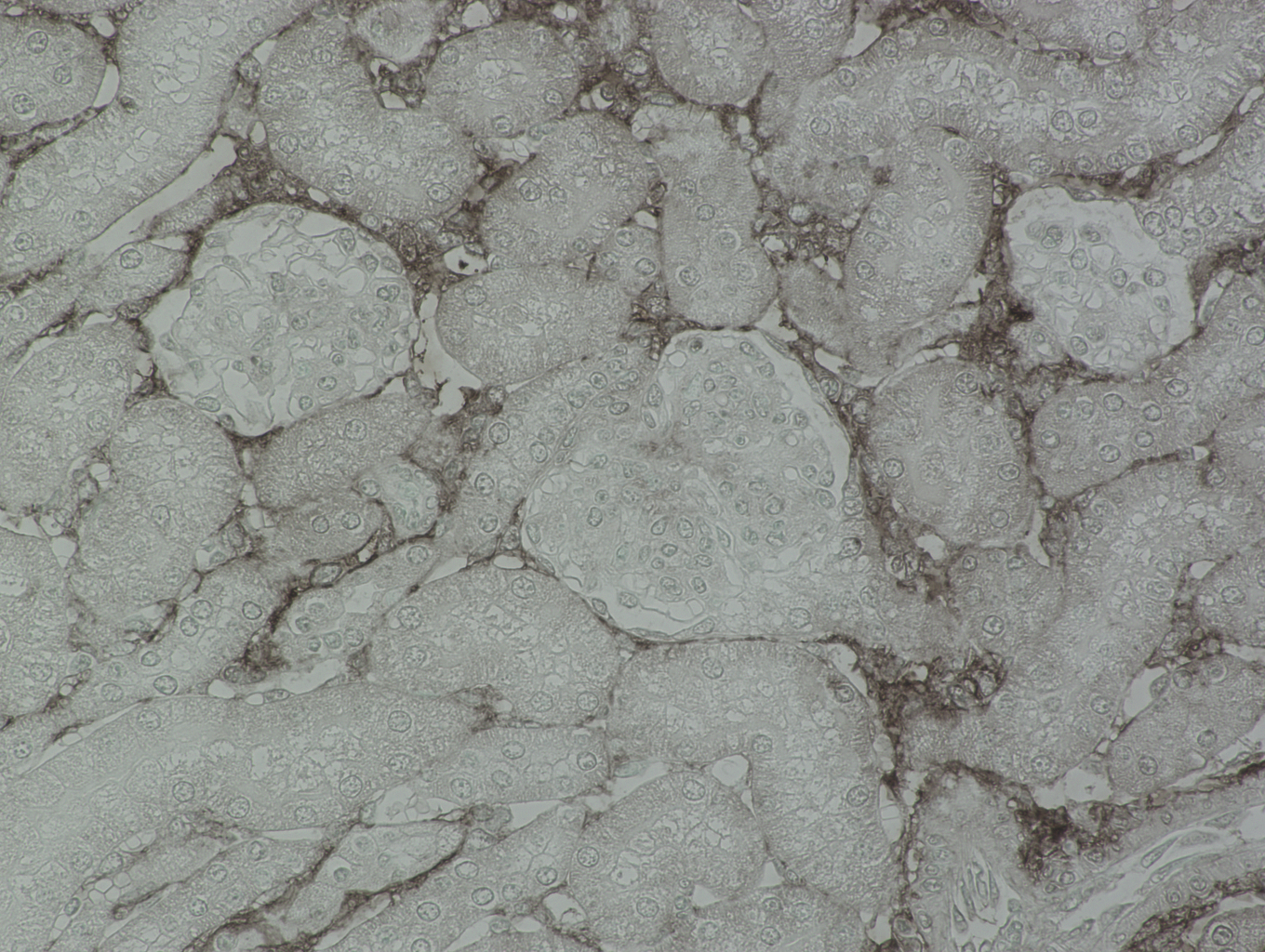

Supplement: Supplementary file 9 — Source Data for Figure 3 [file EMMM-12-e11021-s007.zip › SourceData_Fig3/Fig3E_6weeks.tif]

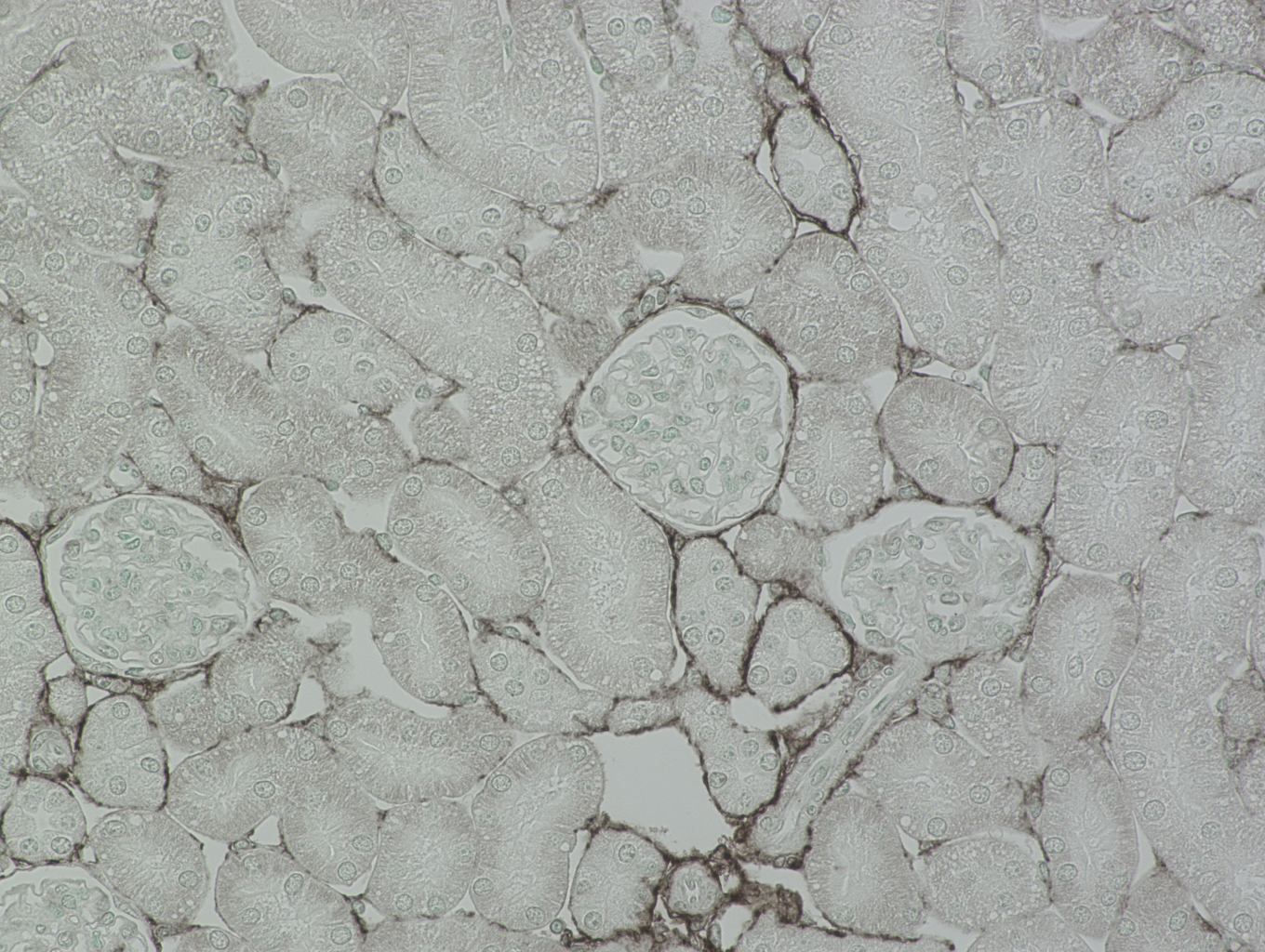

Supplement: Supplementary file 9 — Source Data for Figure 3 [file EMMM-12-e11021-s007.zip › SourceData_Fig3/Fig3E_wt.tif]

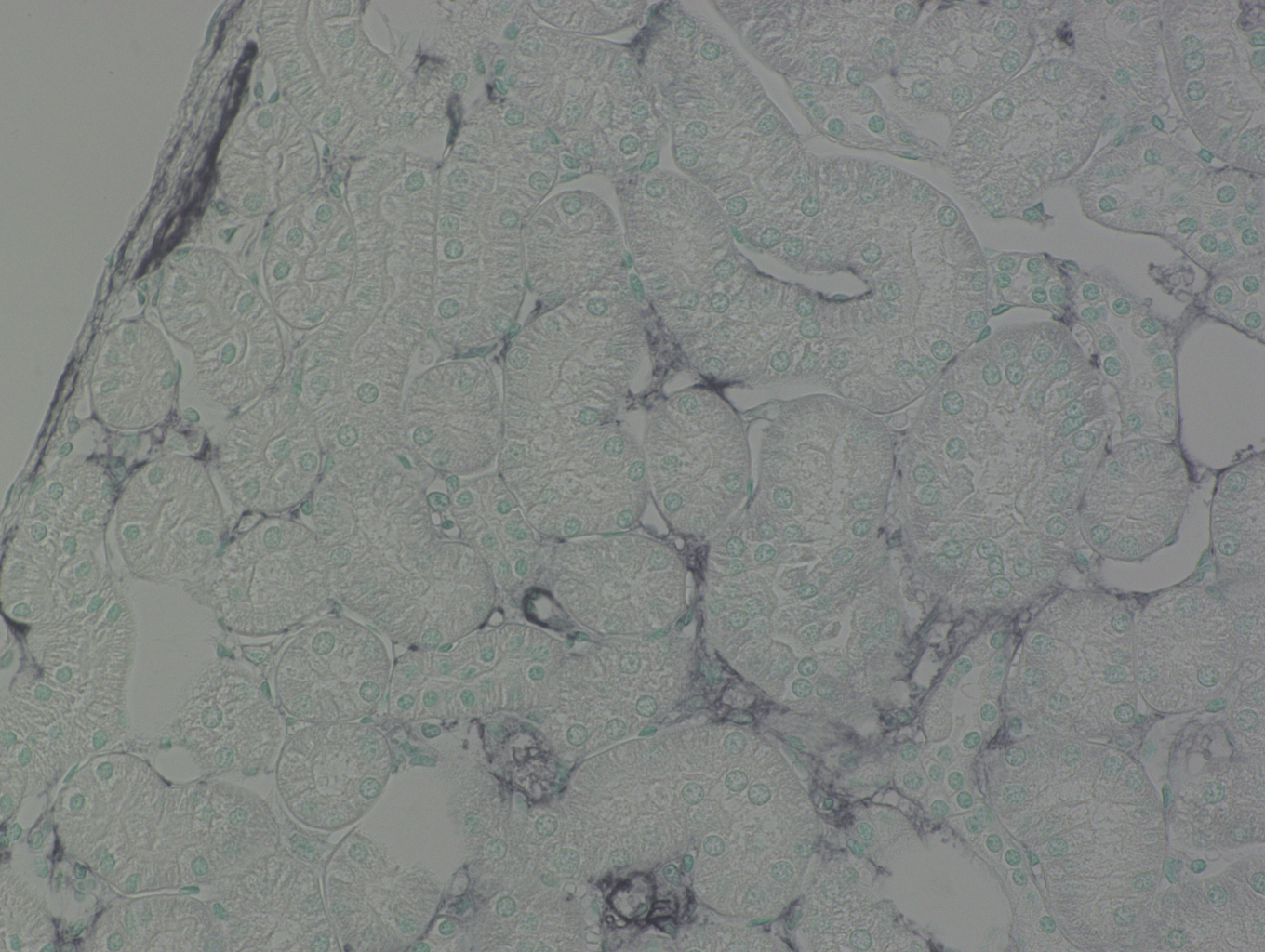

Supplement: Supplementary file 10 — Source Data for Figure 4 [file EMMM-12-e11021-s008.zip › SourceData_Fig4/Fig3A_35weeks.tif]

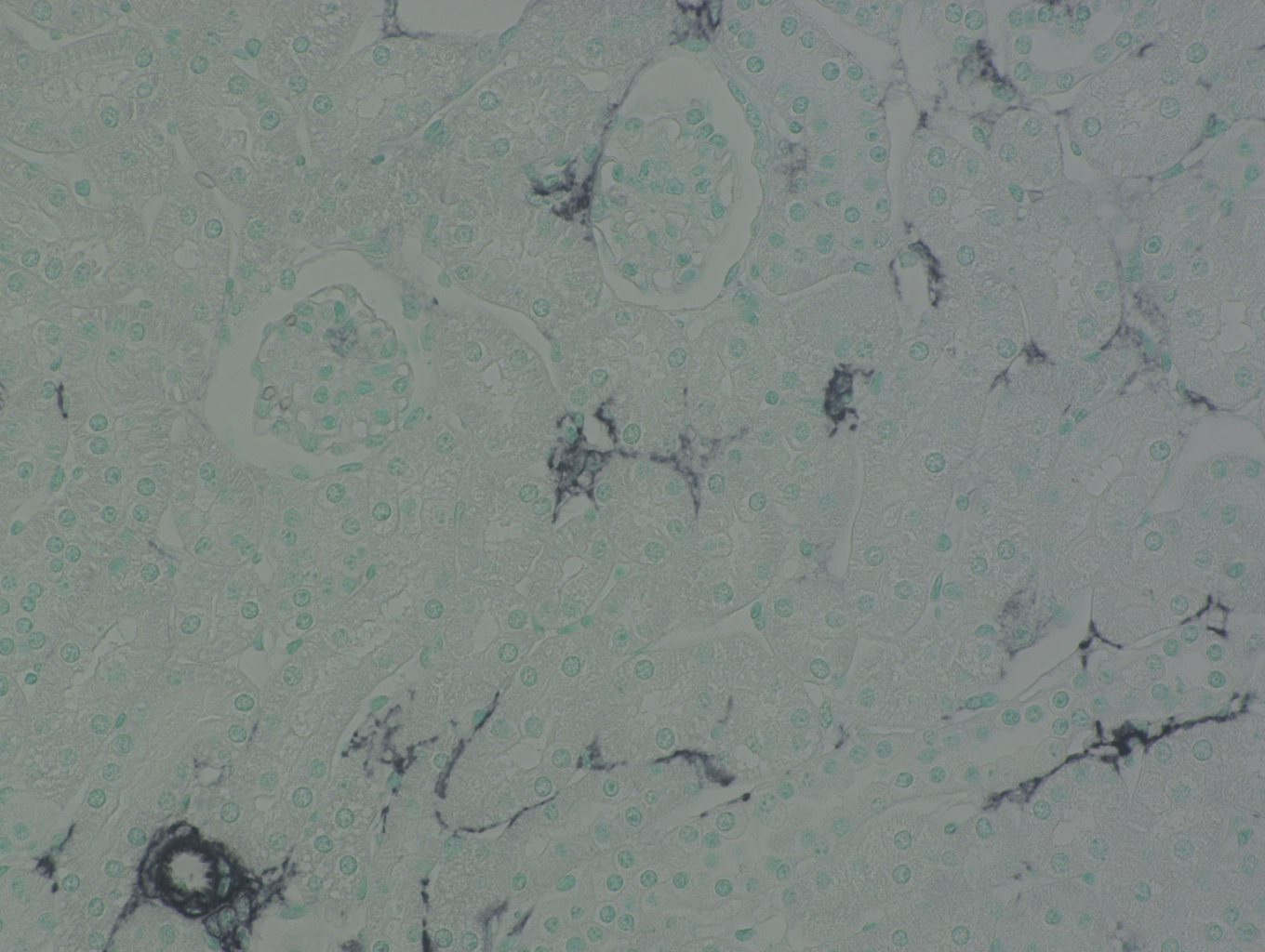

Supplement: Supplementary file 10 — Source Data for Figure 4 [file EMMM-12-e11021-s008.zip › SourceData_Fig4/Fig3A_6weeks.tif]

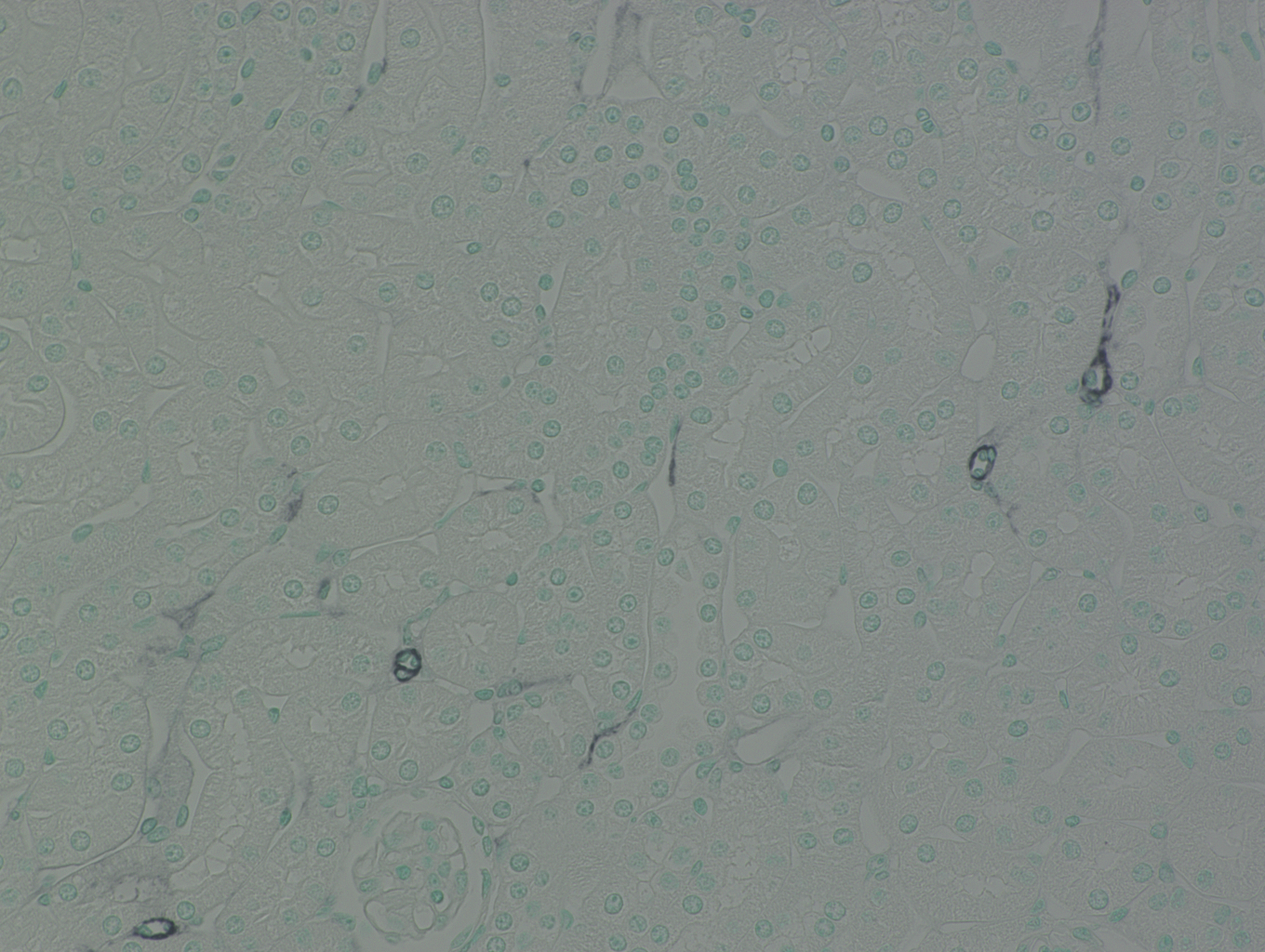

Supplement: Supplementary file 10 — Source Data for Figure 4 [file EMMM-12-e11021-s008.zip › SourceData_Fig4/Fig3A_wt.tif]

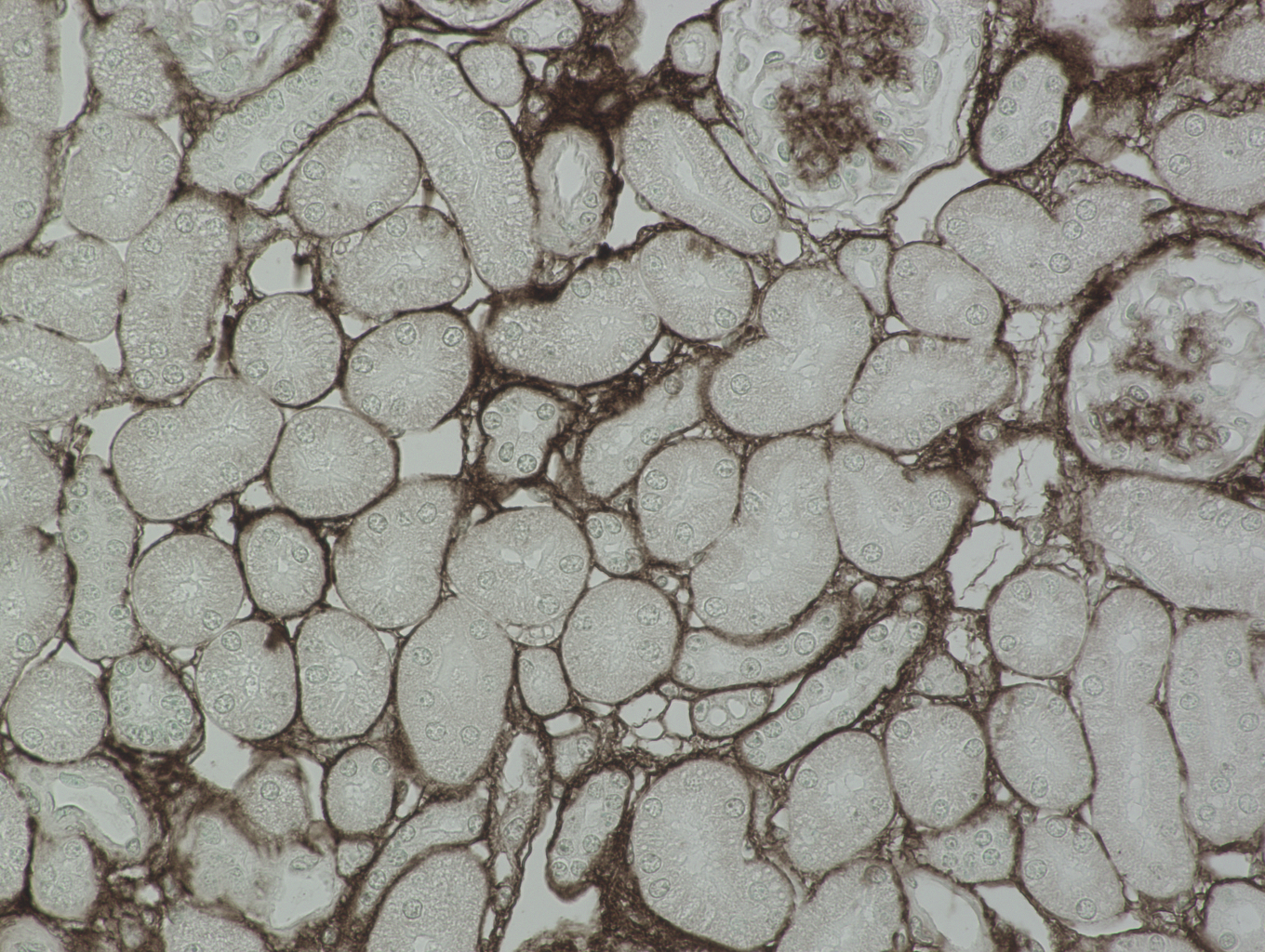

Supplement: Supplementary file 10 — Source Data for Figure 4 [file EMMM-12-e11021-s008.zip › SourceData_Fig4/Fig3C_35weeks.tif]

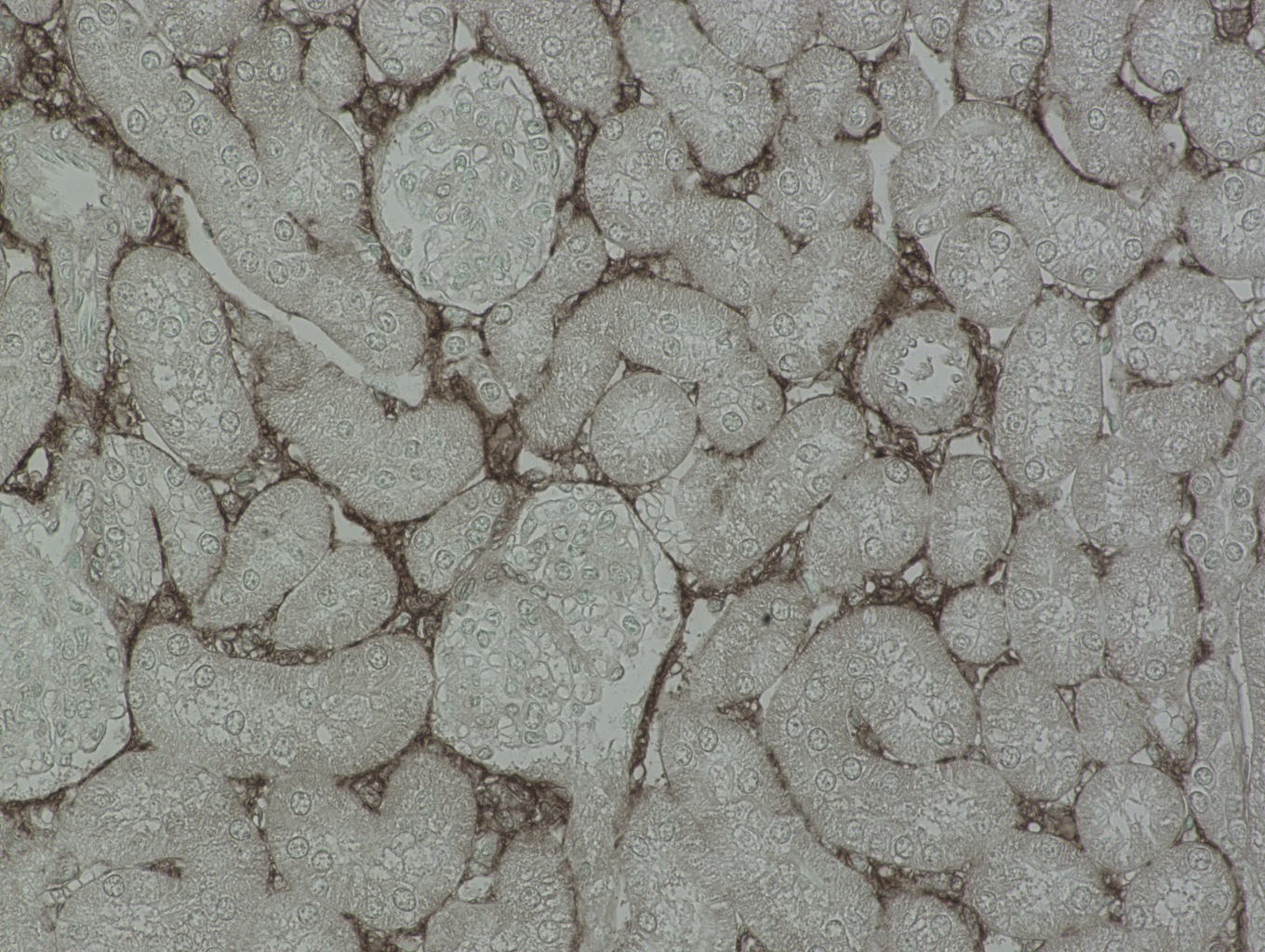

Supplement: Supplementary file 10 — Source Data for Figure 4 [file EMMM-12-e11021-s008.zip › SourceData_Fig4/Fig3C_6weeks.tif]

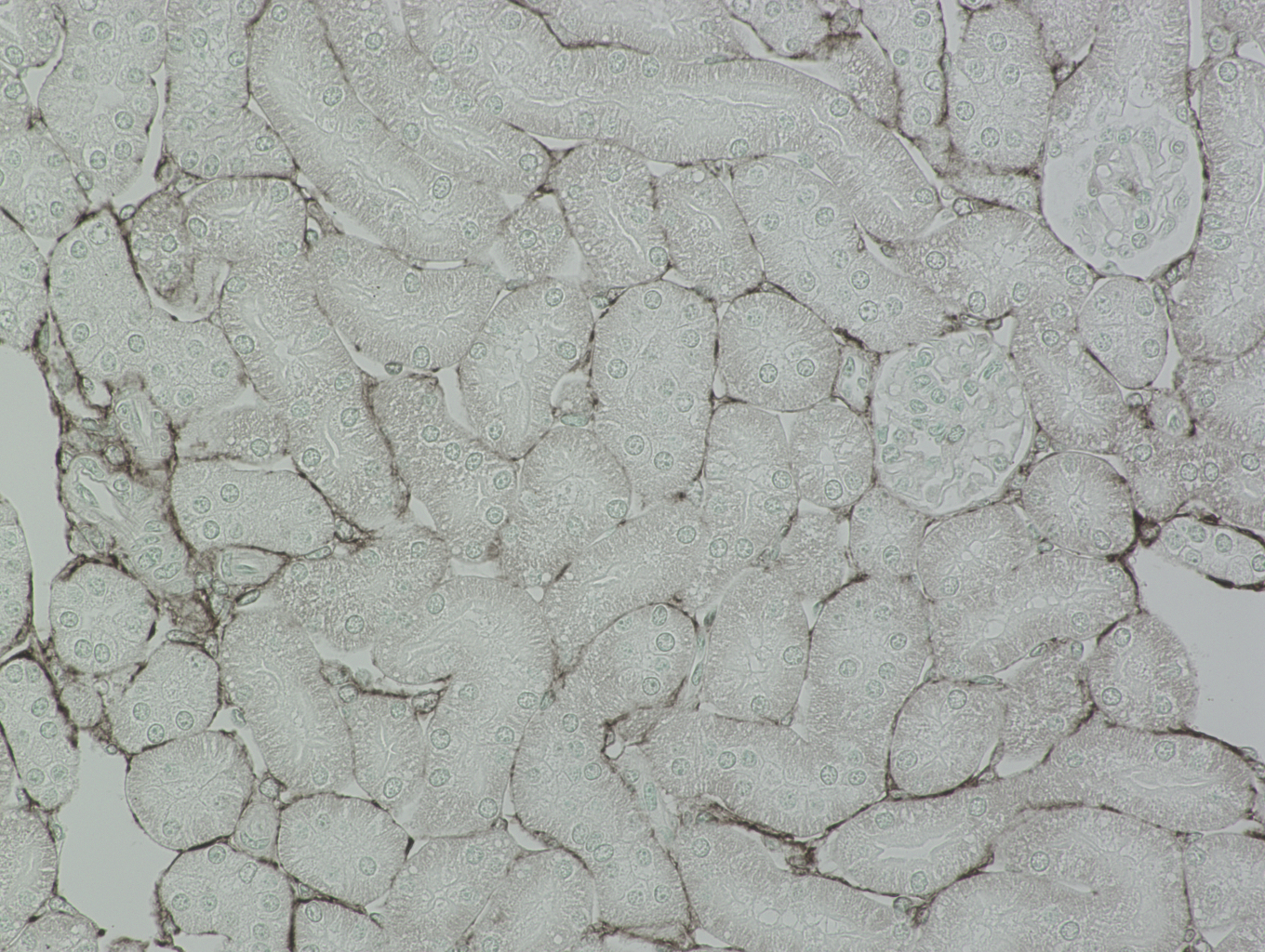

Supplement: Supplementary file 10 — Source Data for Figure 4 [file EMMM-12-e11021-s008.zip › SourceData_Fig4/Fig3C_wt.tif]

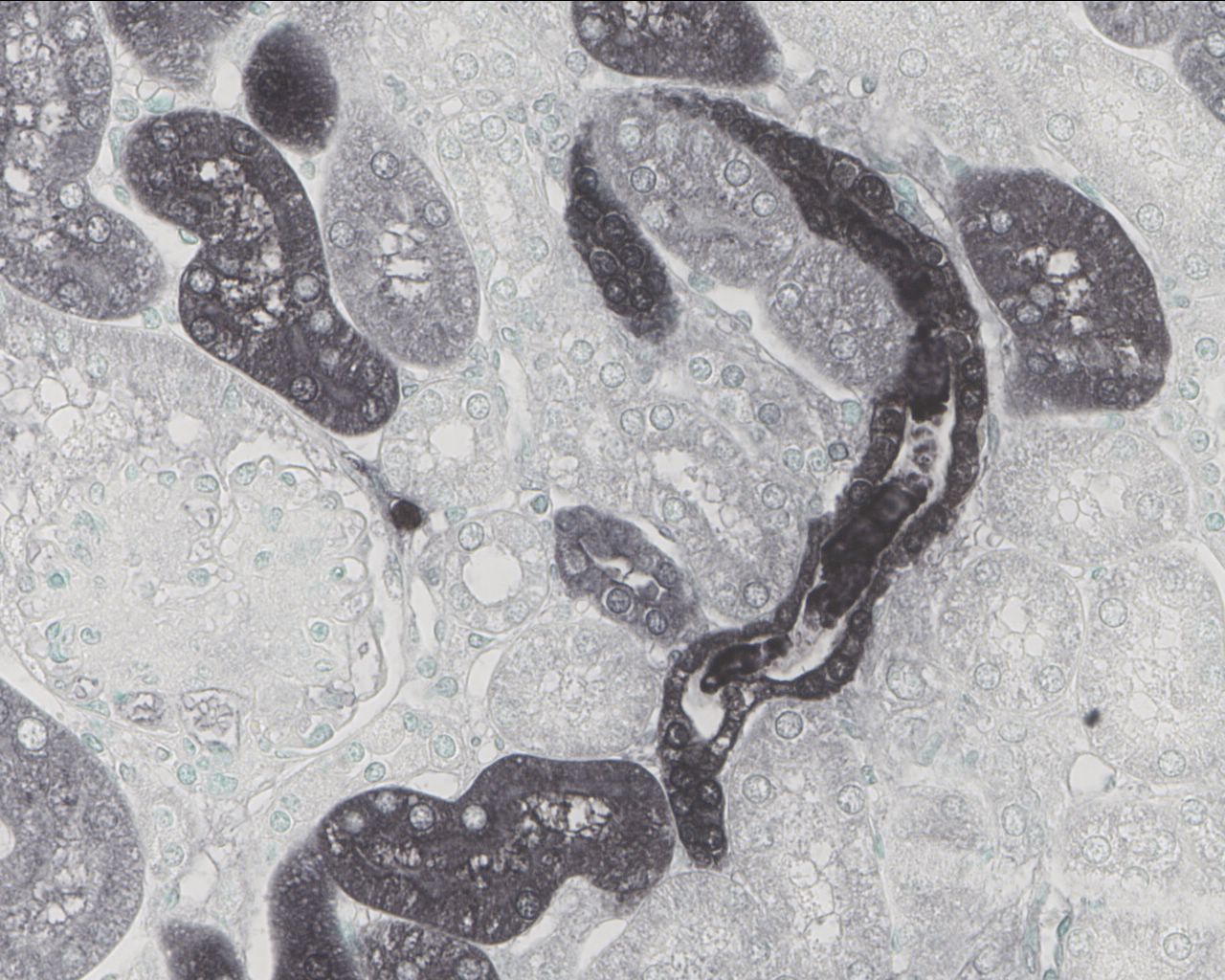

Supplement: Supplementary file 10 — Source Data for Figure 4 [file EMMM-12-e11021-s008.zip › SourceData_Fig4/Fig3D_35weeks.jpg]

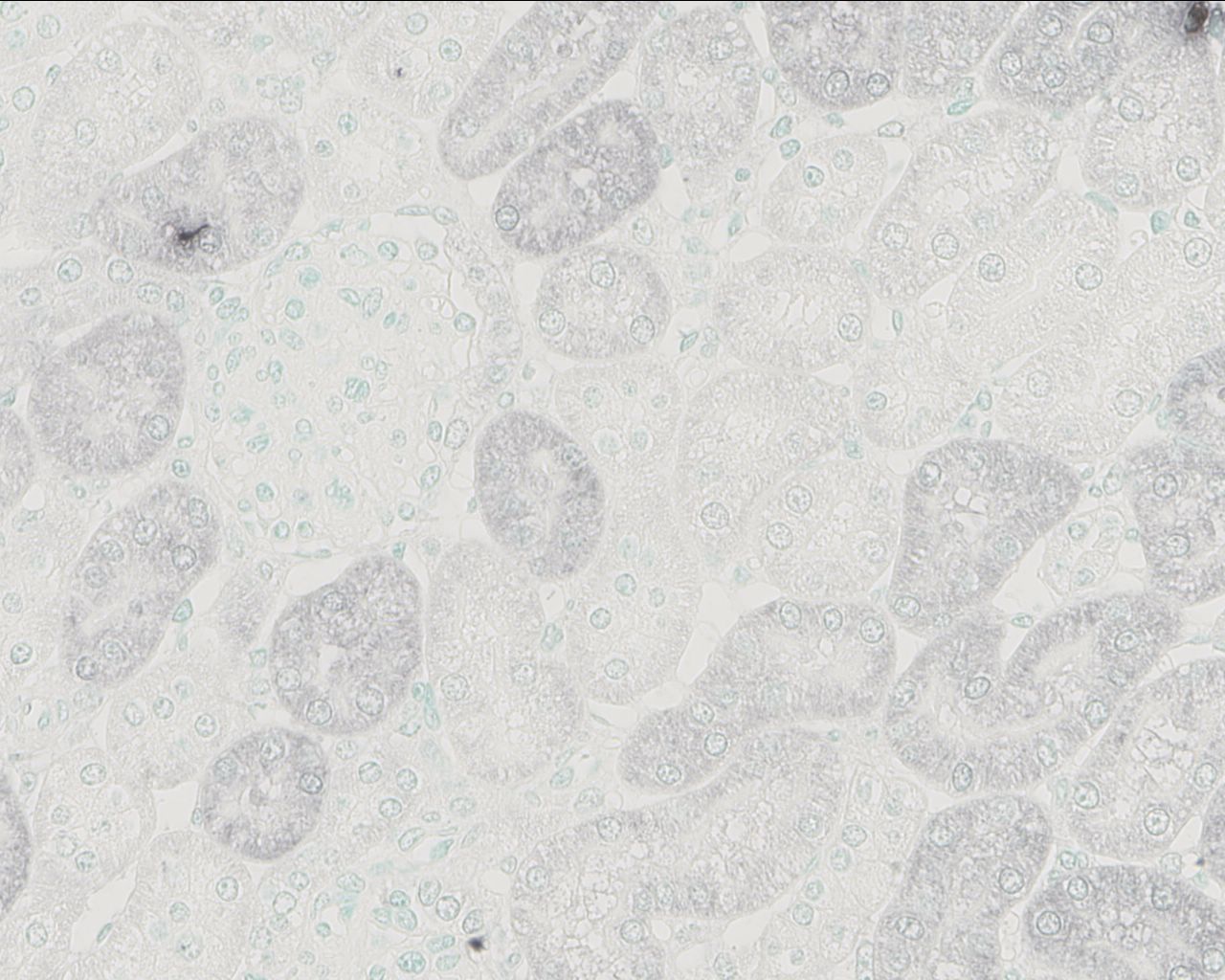

Supplement: Supplementary file 10 — Source Data for Figure 4 [file EMMM-12-e11021-s008.zip › SourceData_Fig4/Fig3D_6weeks.jpg]

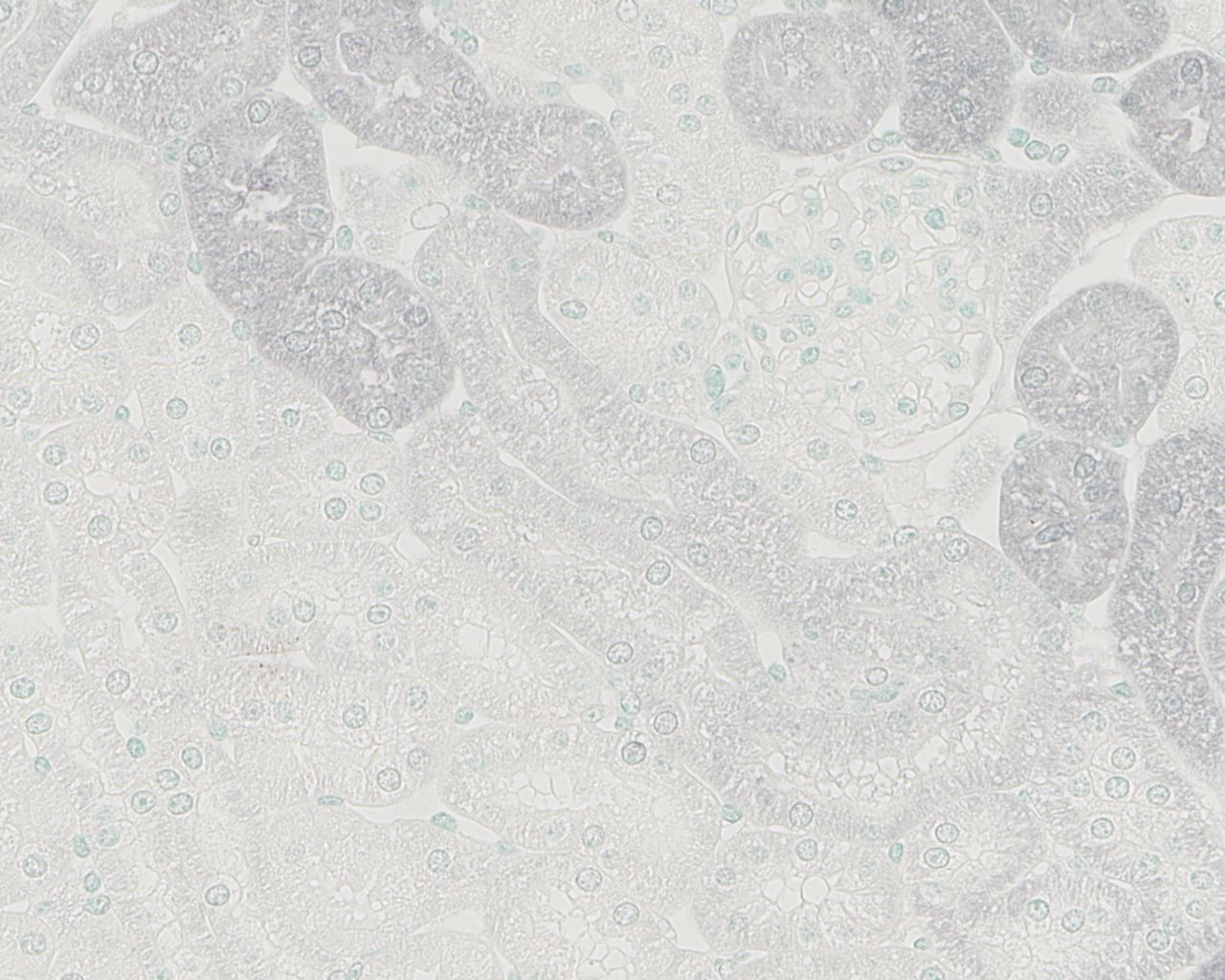

Supplement: Supplementary file 10 — Source Data for Figure 4 [file EMMM-12-e11021-s008.zip › SourceData_Fig4/Fig3D_wt.jpg]

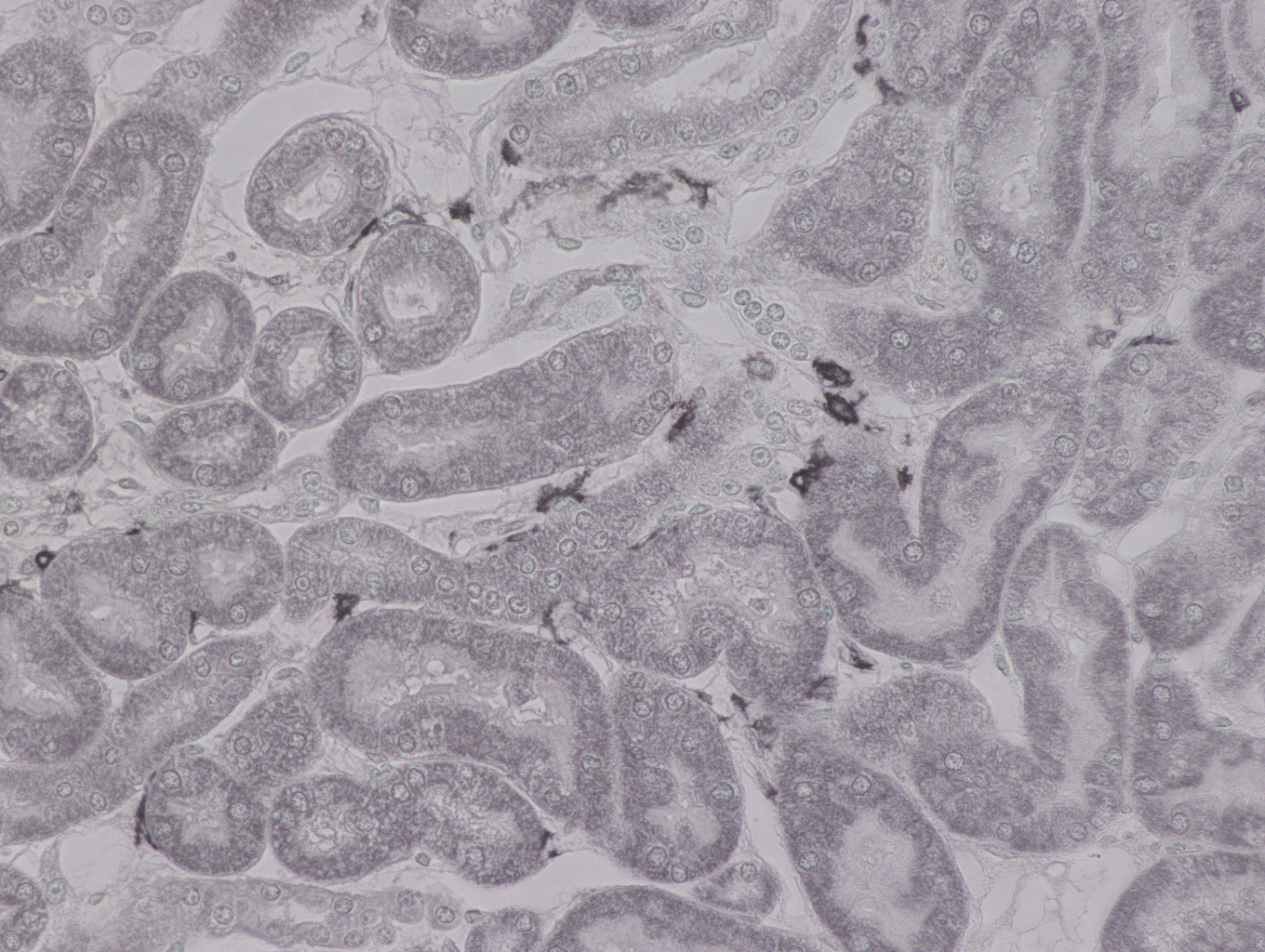

Supplement: Supplementary file 10 — Source Data for Figure 4 [file EMMM-12-e11021-s008.zip › SourceData_Fig4/Fig3G_35weeks.TIF]

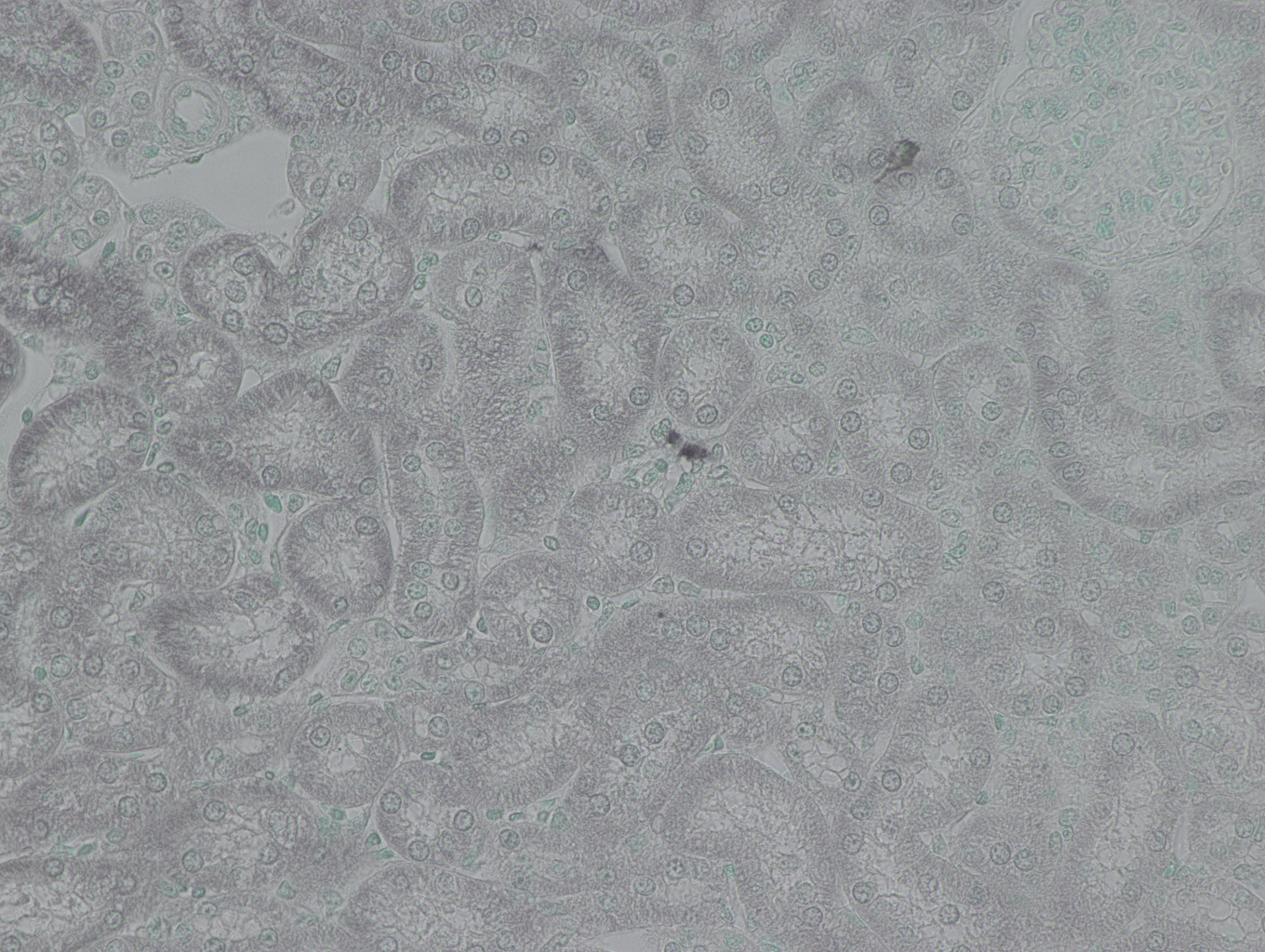

Supplement: Supplementary file 10 — Source Data for Figure 4 [file EMMM-12-e11021-s008.zip › SourceData_Fig4/Fig3G_6weeks.TIF]

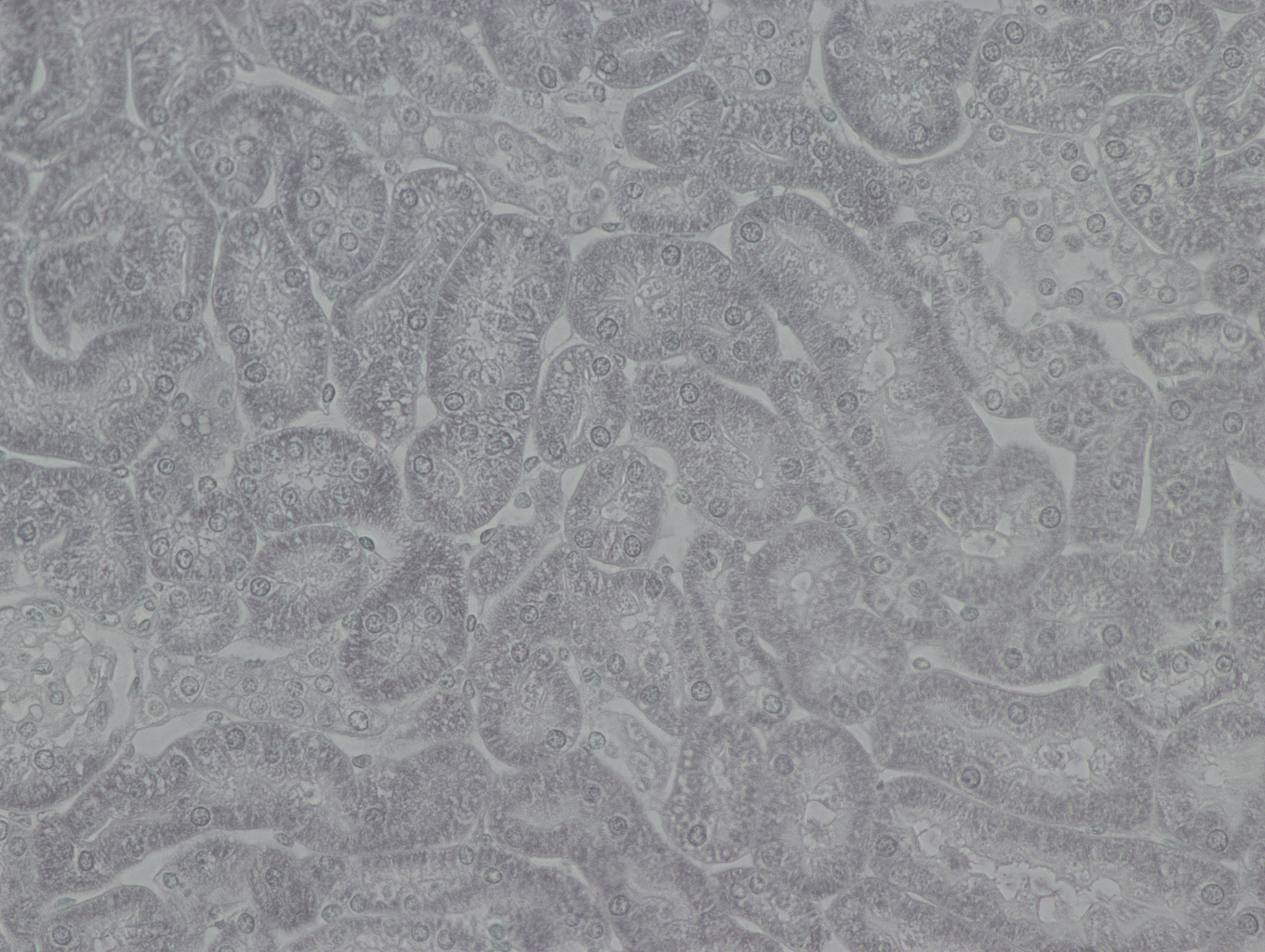

Supplement: Supplementary file 10 — Source Data for Figure 4 [file EMMM-12-e11021-s008.zip › SourceData_Fig4/Fig3G_wt.TIF]

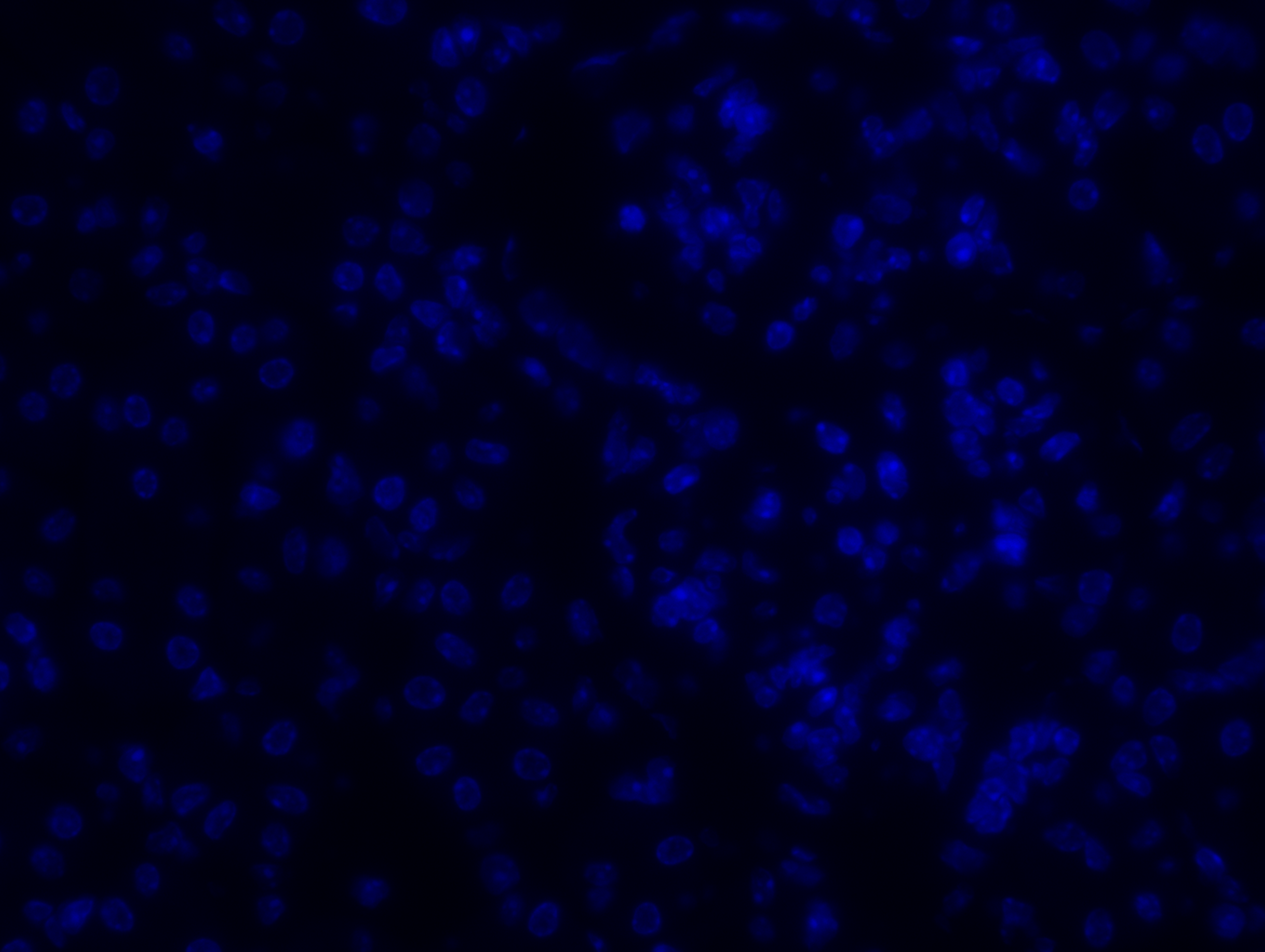

Supplement: Supplementary file 11 — Source Data for Figure 6 [file EMMM-12-e11021-s009.zip › SourceData_Fig6/Fig6E_mutant_DAPI.tif]

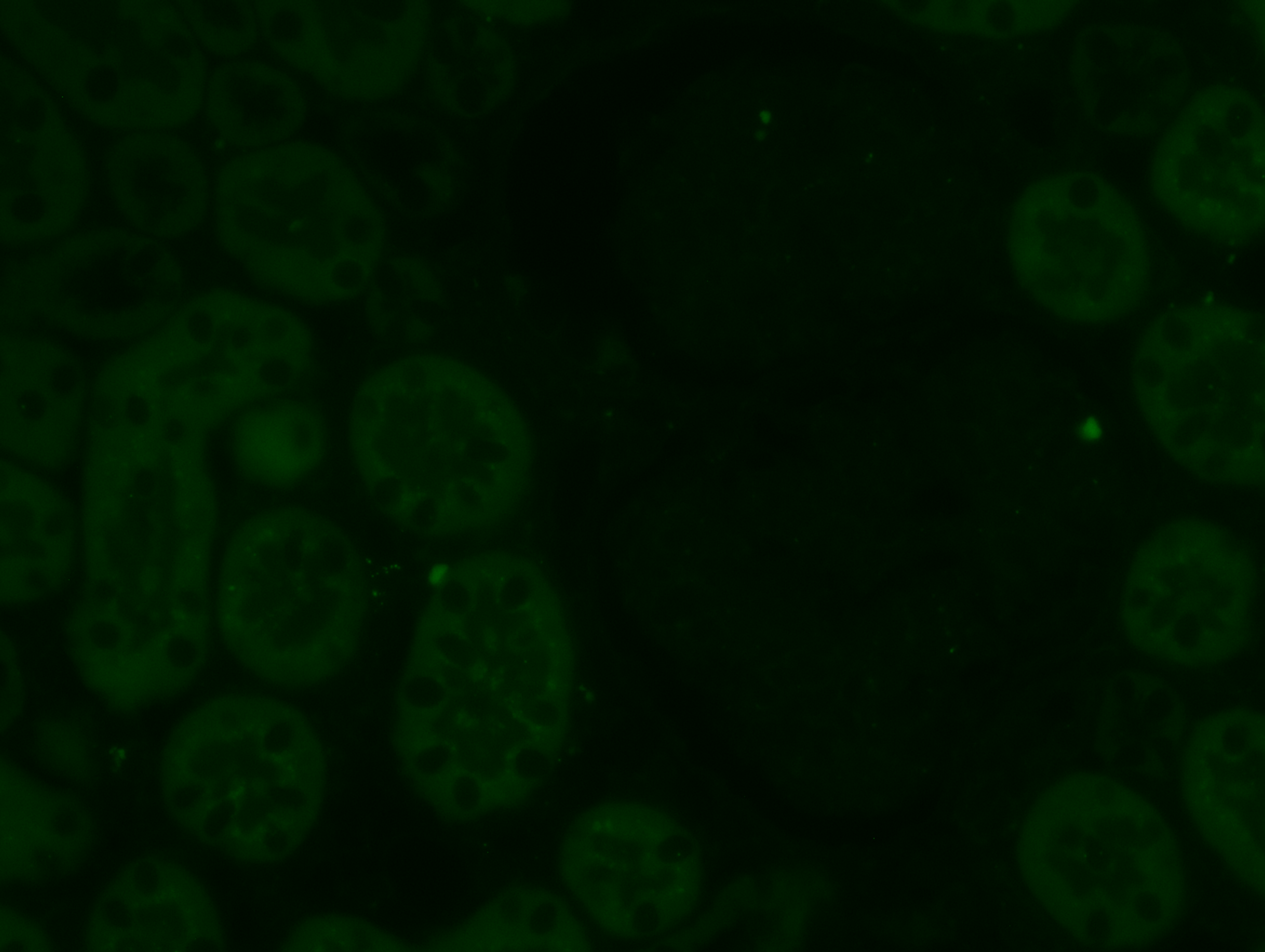

Supplement: Supplementary file 11 — Source Data for Figure 6 [file EMMM-12-e11021-s009.zip › SourceData_Fig6/Fig6E_mutant_EPO.tif]

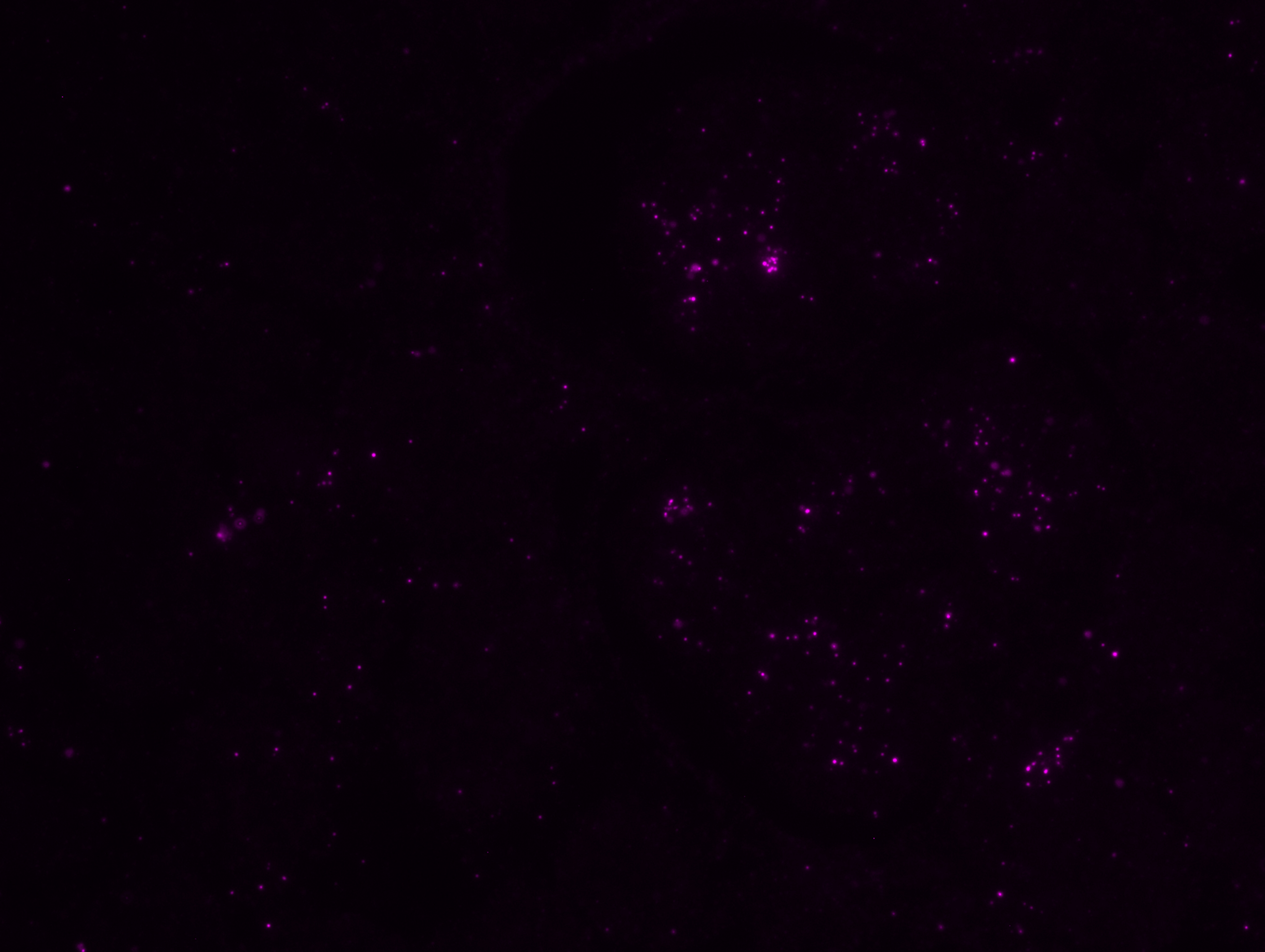

Supplement: Supplementary file 11 — Source Data for Figure 6 [file EMMM-12-e11021-s009.zip › SourceData_Fig6/Fig6E_mutant_PDGFRb.tif]

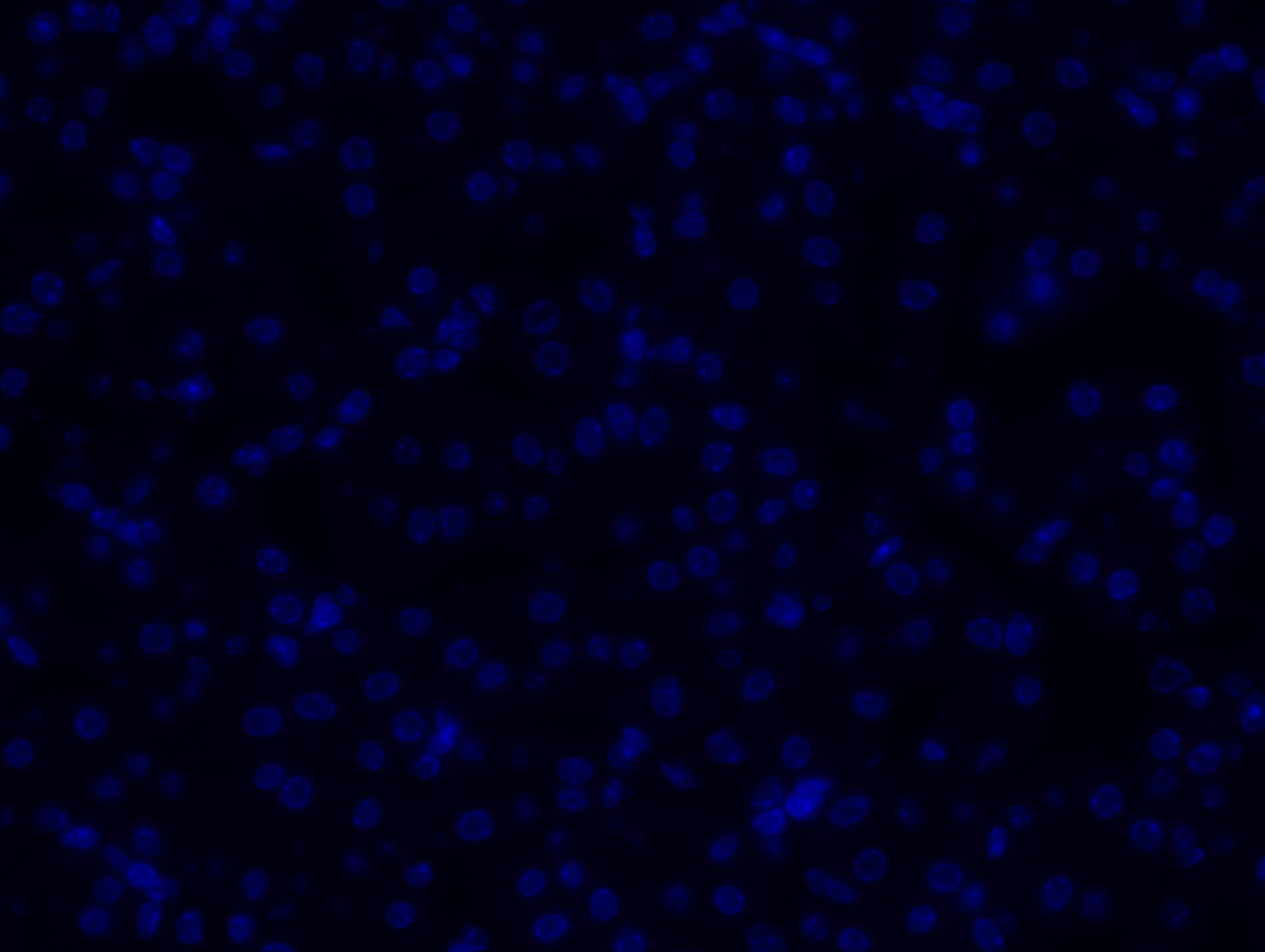

Supplement: Supplementary file 11 — Source Data for Figure 6 [file EMMM-12-e11021-s009.zip › SourceData_Fig6/Fig6E_wt_DAPI.tif]

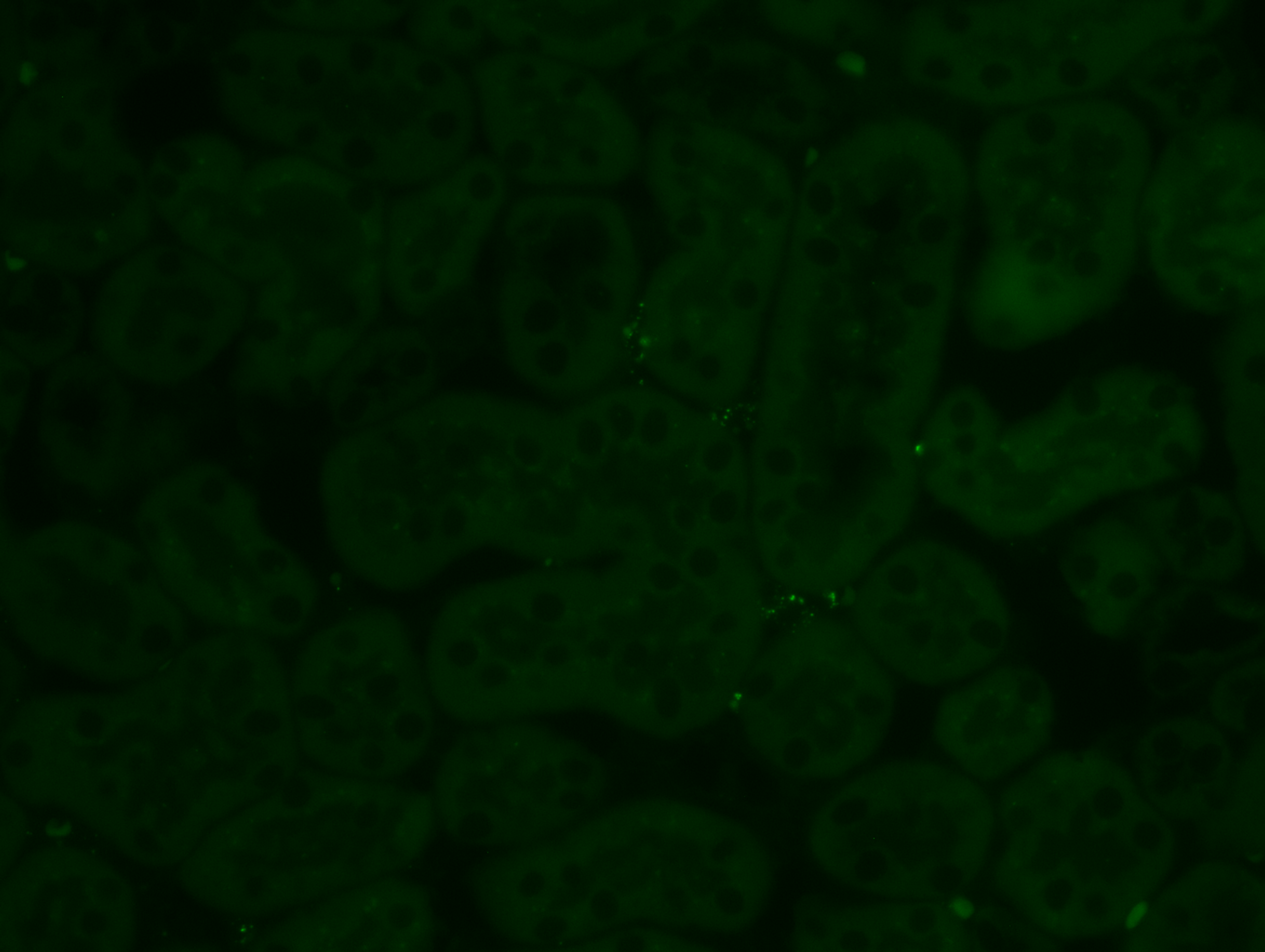

Supplement: Supplementary file 11 — Source Data for Figure 6 [file EMMM-12-e11021-s009.zip › SourceData_Fig6/Fig6E_wt_EPO.tif]

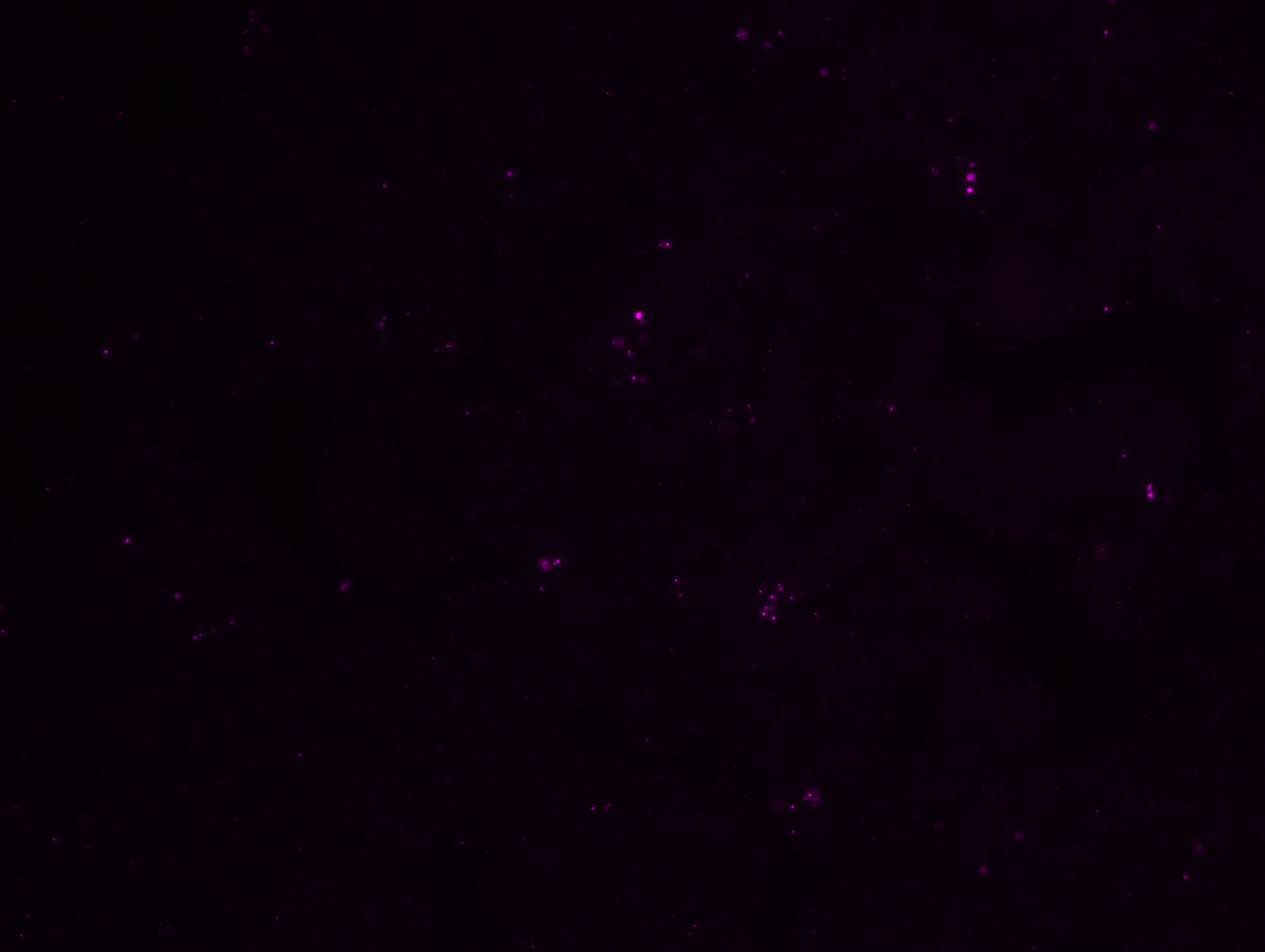

Supplement: Supplementary file 11 — Source Data for Figure 6 [file EMMM-12-e11021-s009.zip › SourceData_Fig6/Fig6E_wt_PDGFRb.tif]

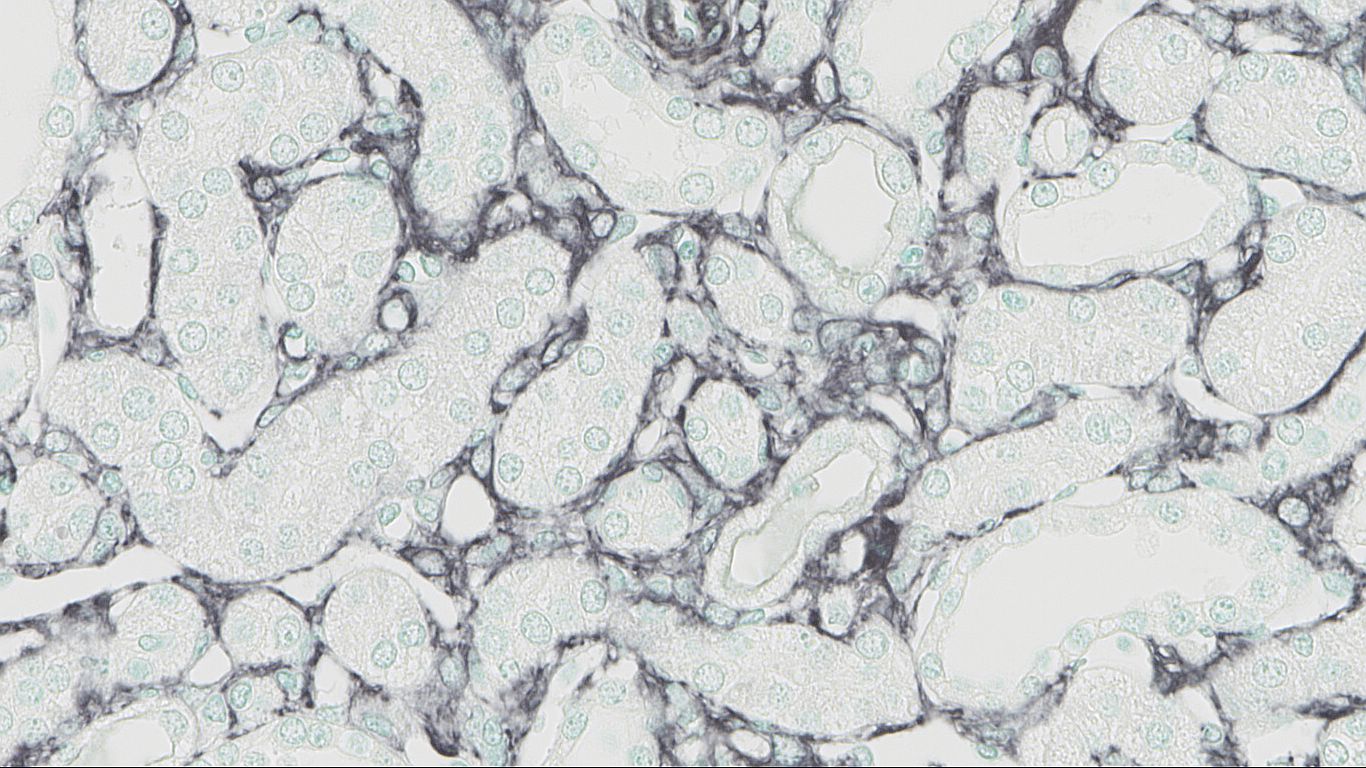

Supplement: Supplementary file 12 — Source Data for Figure 7 [file EMMM-12-e11021-s010.zip › SourceData_Fig7/Fig7B_mutant_asma.jpg]

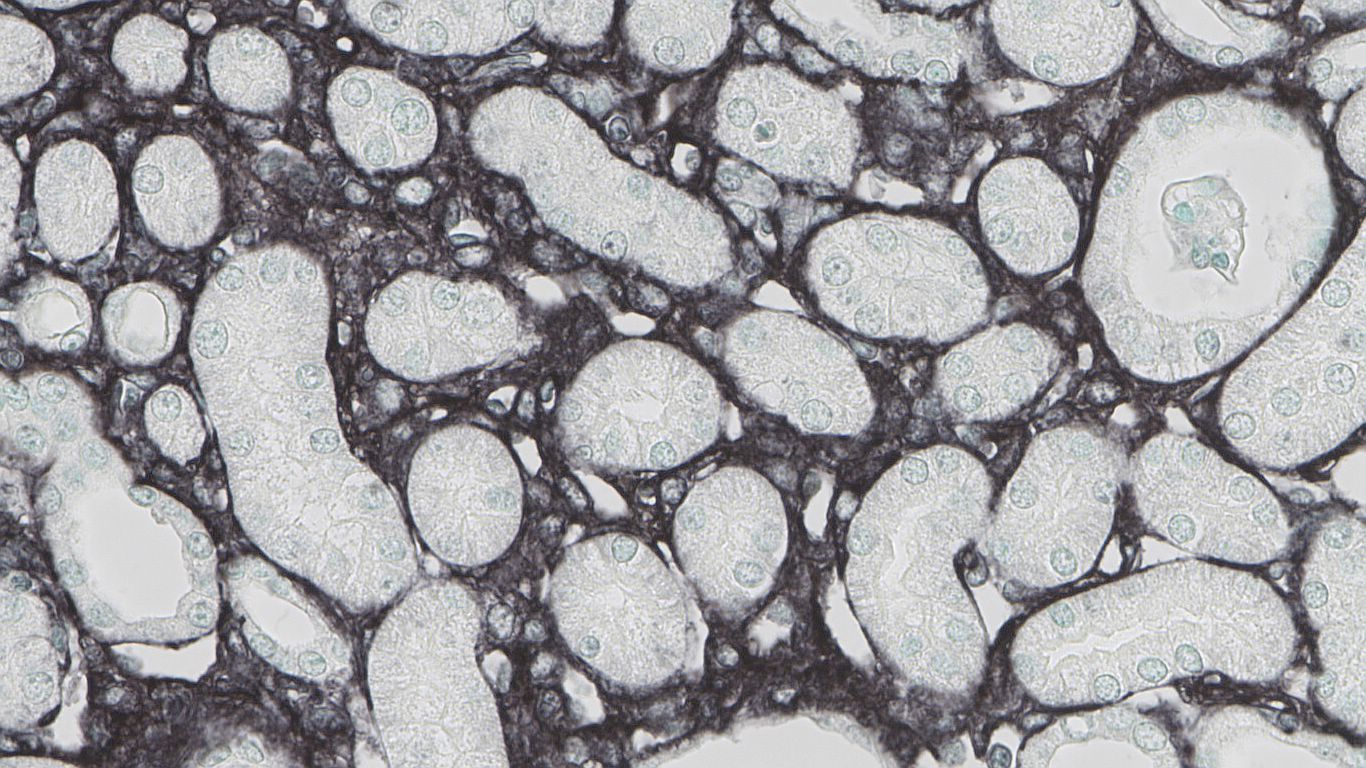

Supplement: Supplementary file 12 — Source Data for Figure 7 [file EMMM-12-e11021-s010.zip › SourceData_Fig7/Fig7B_mutant_col1.jpg]

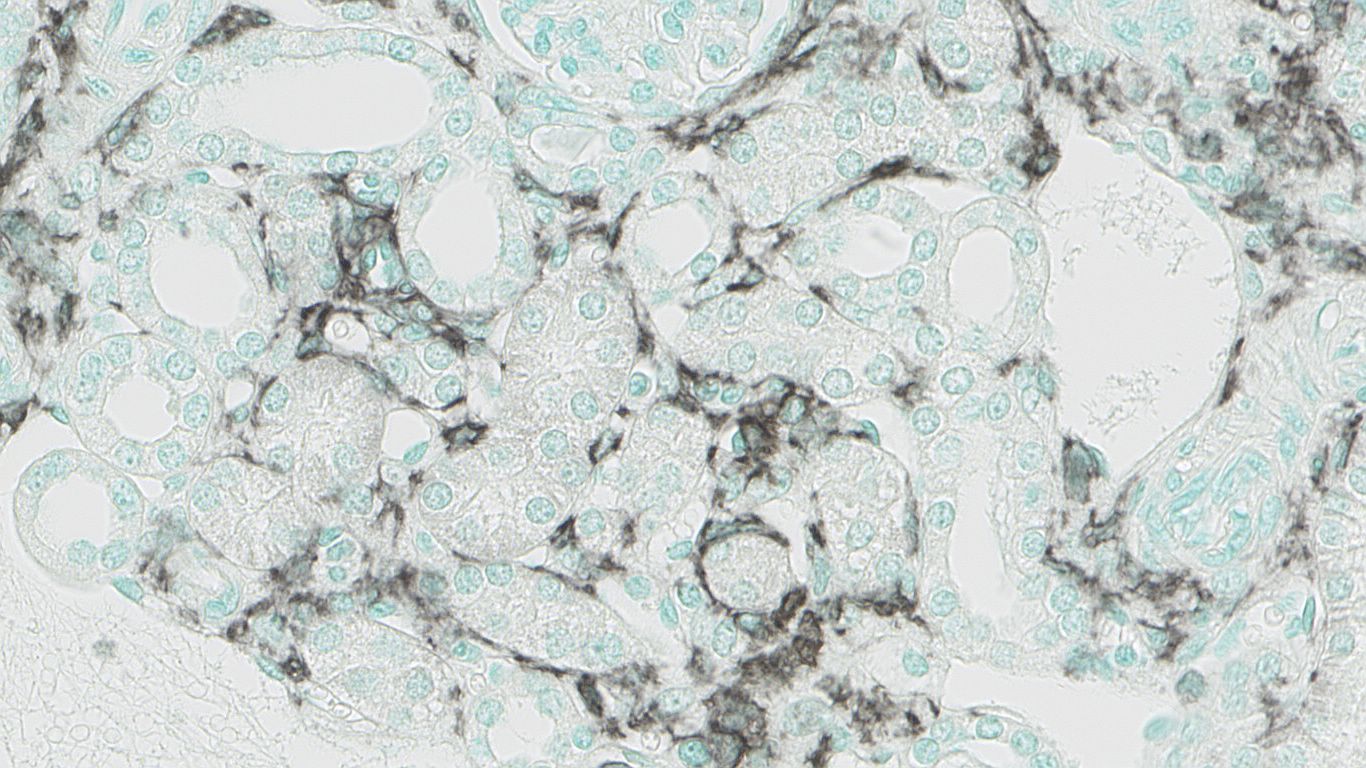

Supplement: Supplementary file 12 — Source Data for Figure 7 [file EMMM-12-e11021-s010.zip › SourceData_Fig7/Fig7B_mutant_F480.jpg]

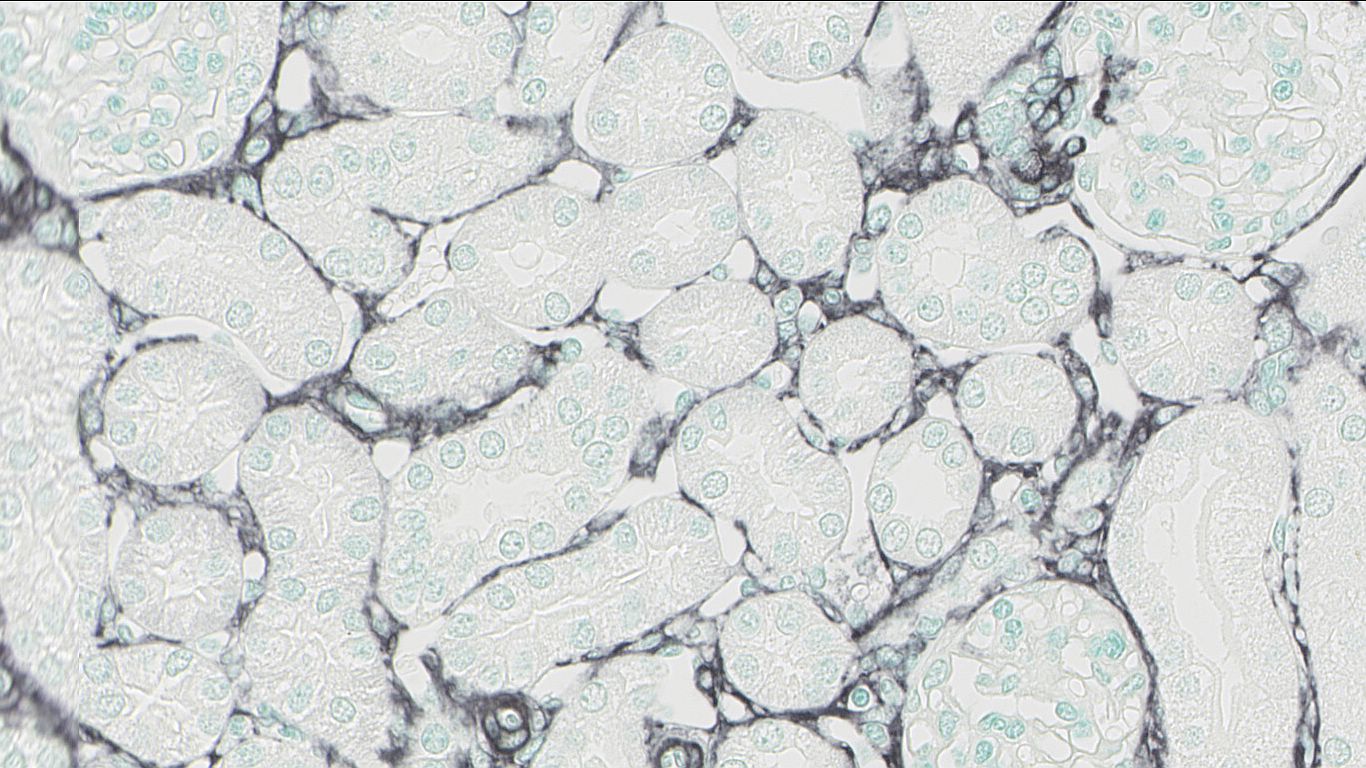

Supplement: Supplementary file 12 — Source Data for Figure 7 [file EMMM-12-e11021-s010.zip › SourceData_Fig7/Fig7B_wt_asma.jpg]

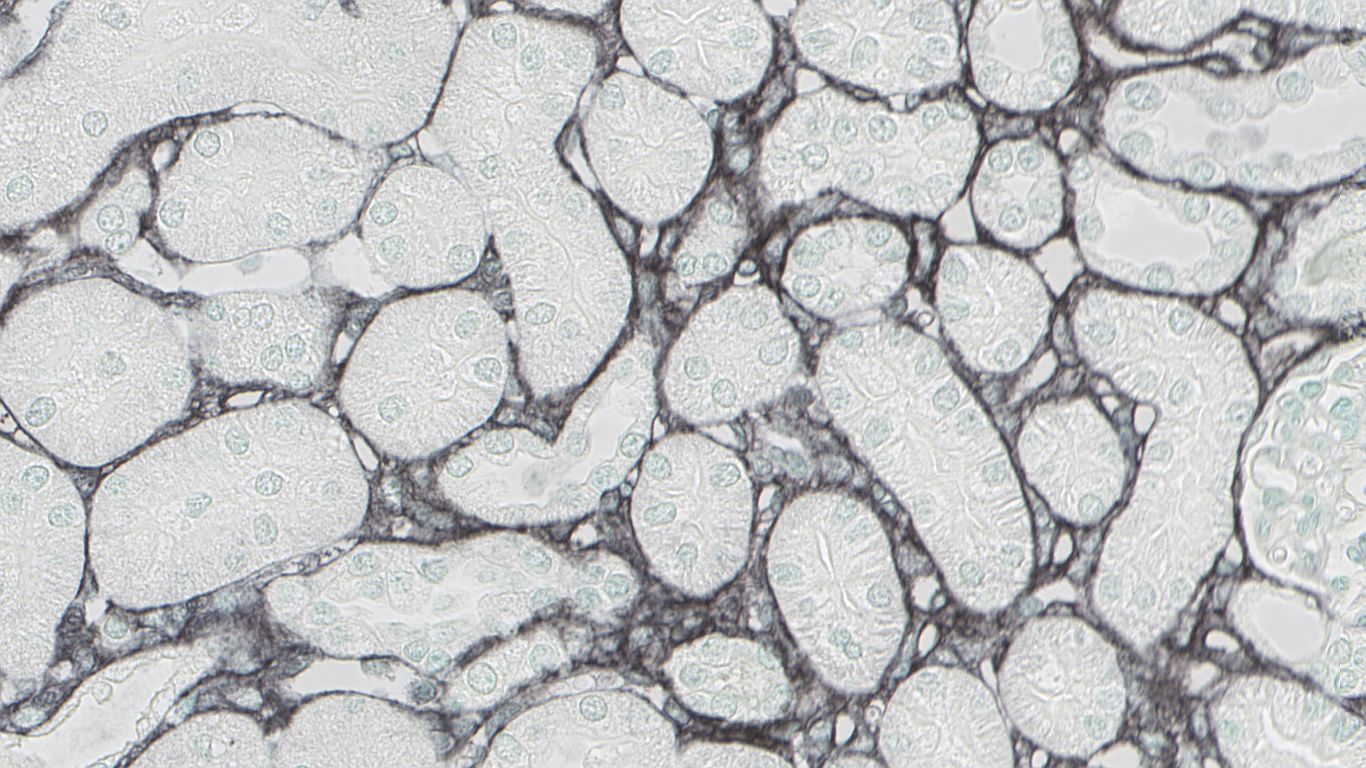

Supplement: Supplementary file 12 — Source Data for Figure 7 [file EMMM-12-e11021-s010.zip › SourceData_Fig7/Fig7B_wt_col1.jpg]

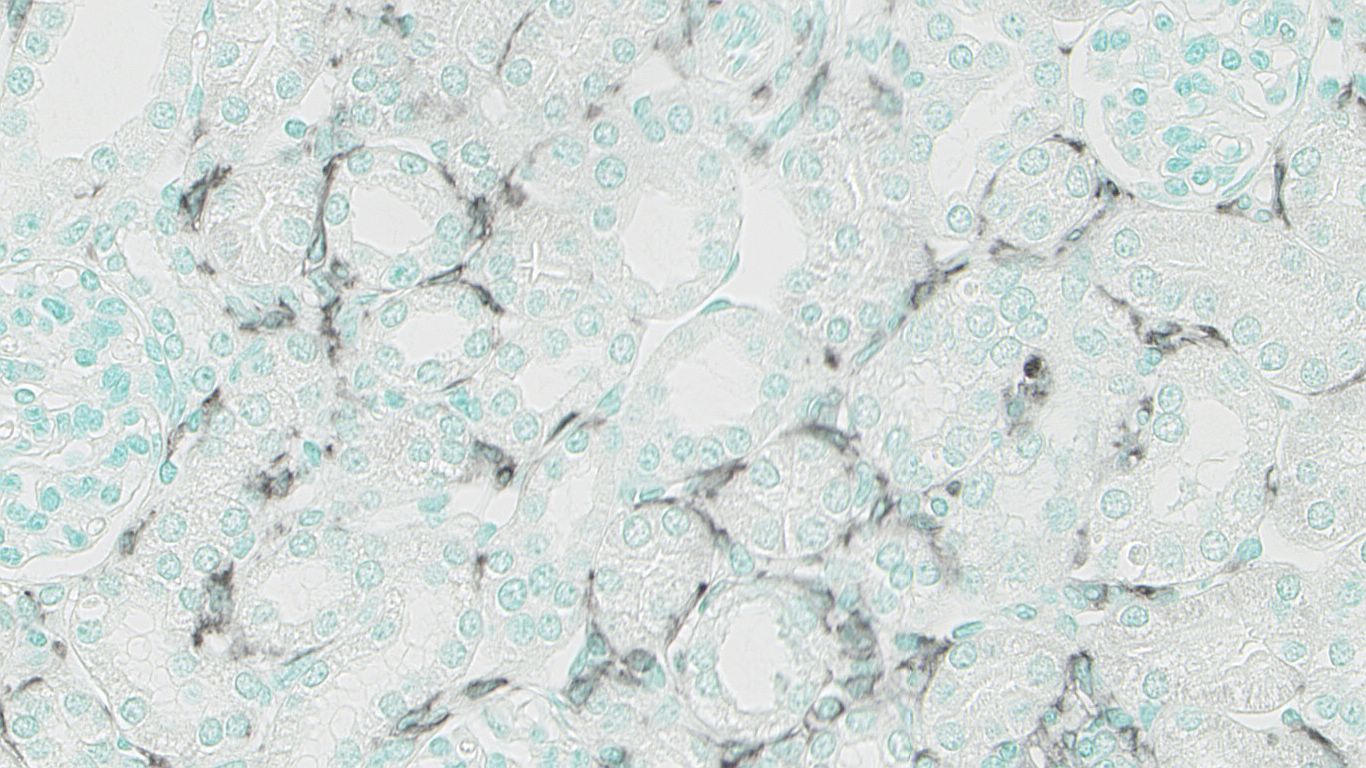

Supplement: Supplementary file 12 — Source Data for Figure 7 [file EMMM-12-e11021-s010.zip › SourceData_Fig7/Fig7B_wt_F480.jpg]

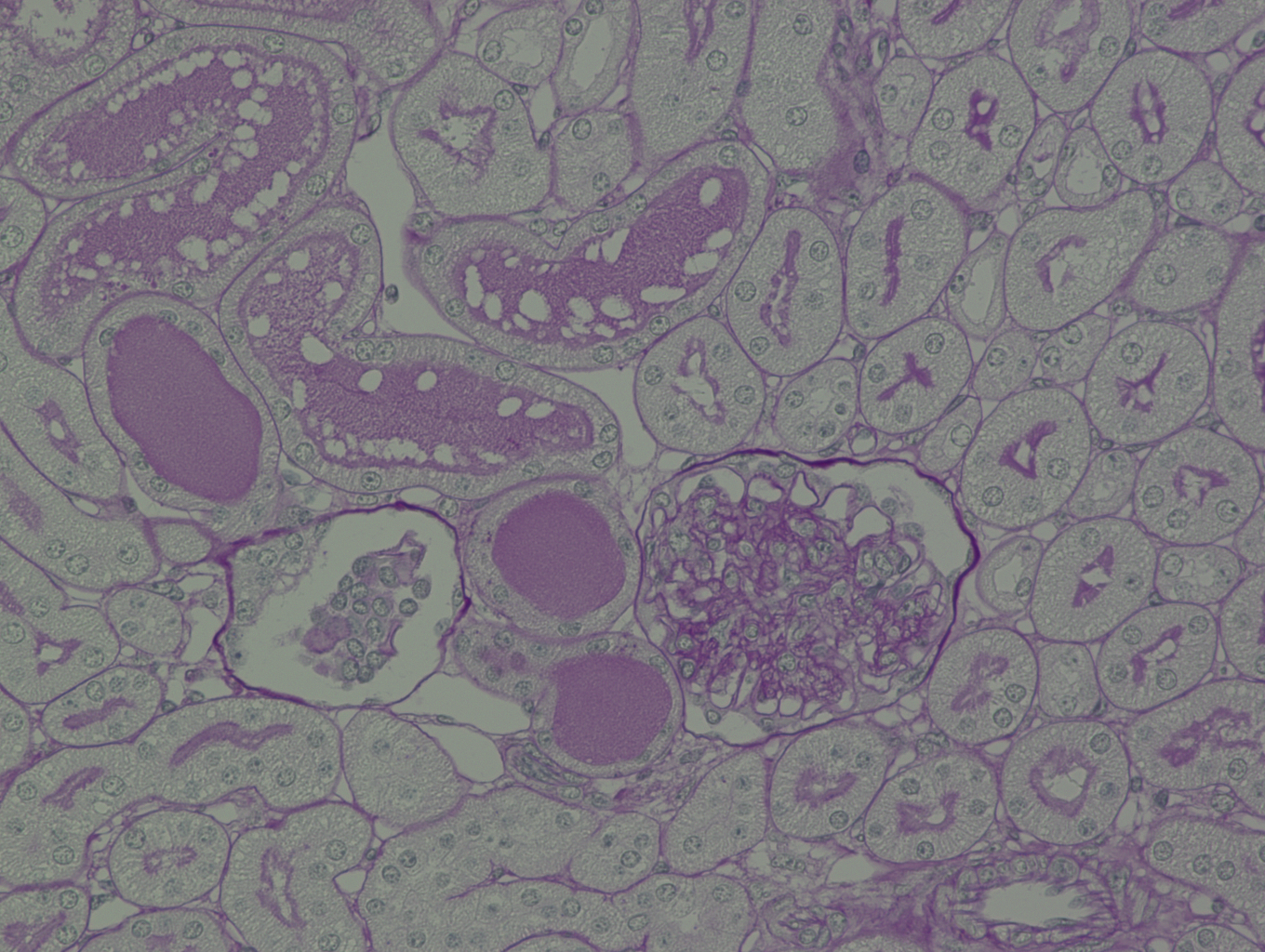

Supplement: Supplementary file 12 — Source Data for Figure 7 [file EMMM-12-e11021-s010.zip › SourceData_Fig7/Fig7F_mutant_AngII_PAS.TIF]

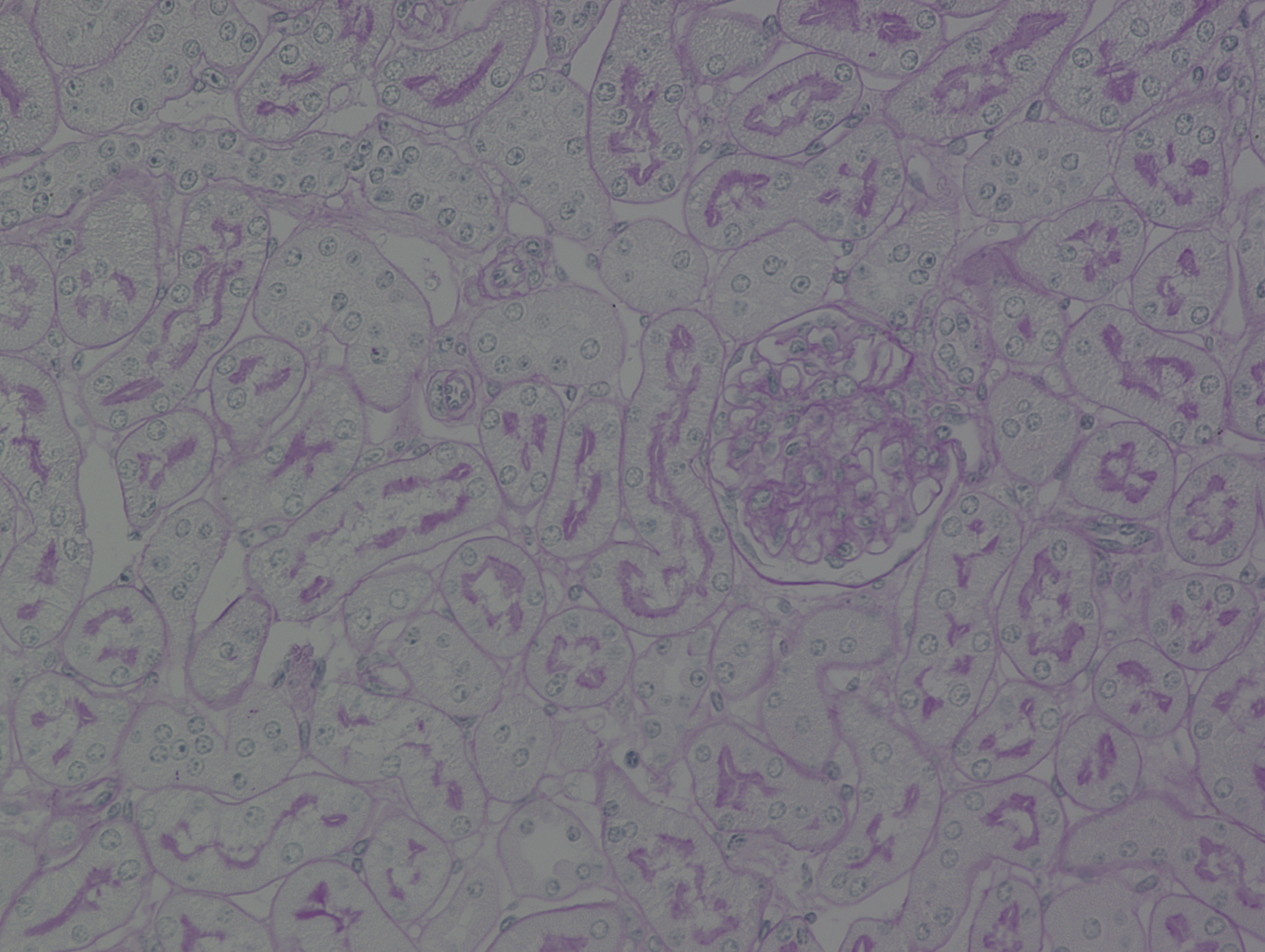

Supplement: Supplementary file 12 — Source Data for Figure 7 [file EMMM-12-e11021-s010.zip › SourceData_Fig7/Fig7F_mutant_ctrl_PAS.TIF]

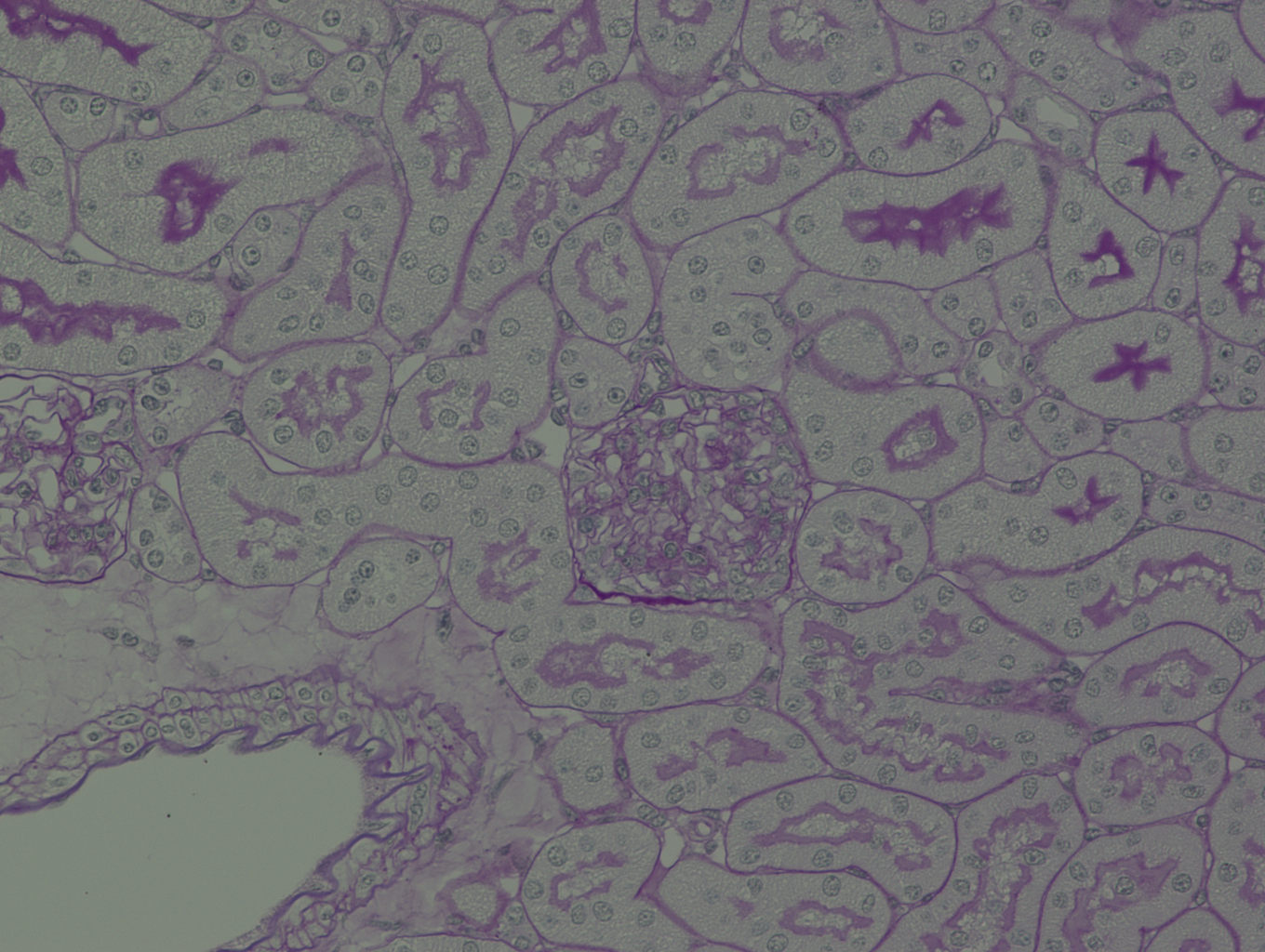

Supplement: Supplementary file 12 — Source Data for Figure 7 [file EMMM-12-e11021-s010.zip › SourceData_Fig7/Fig7F_wt_AngII_PAS.TIF]

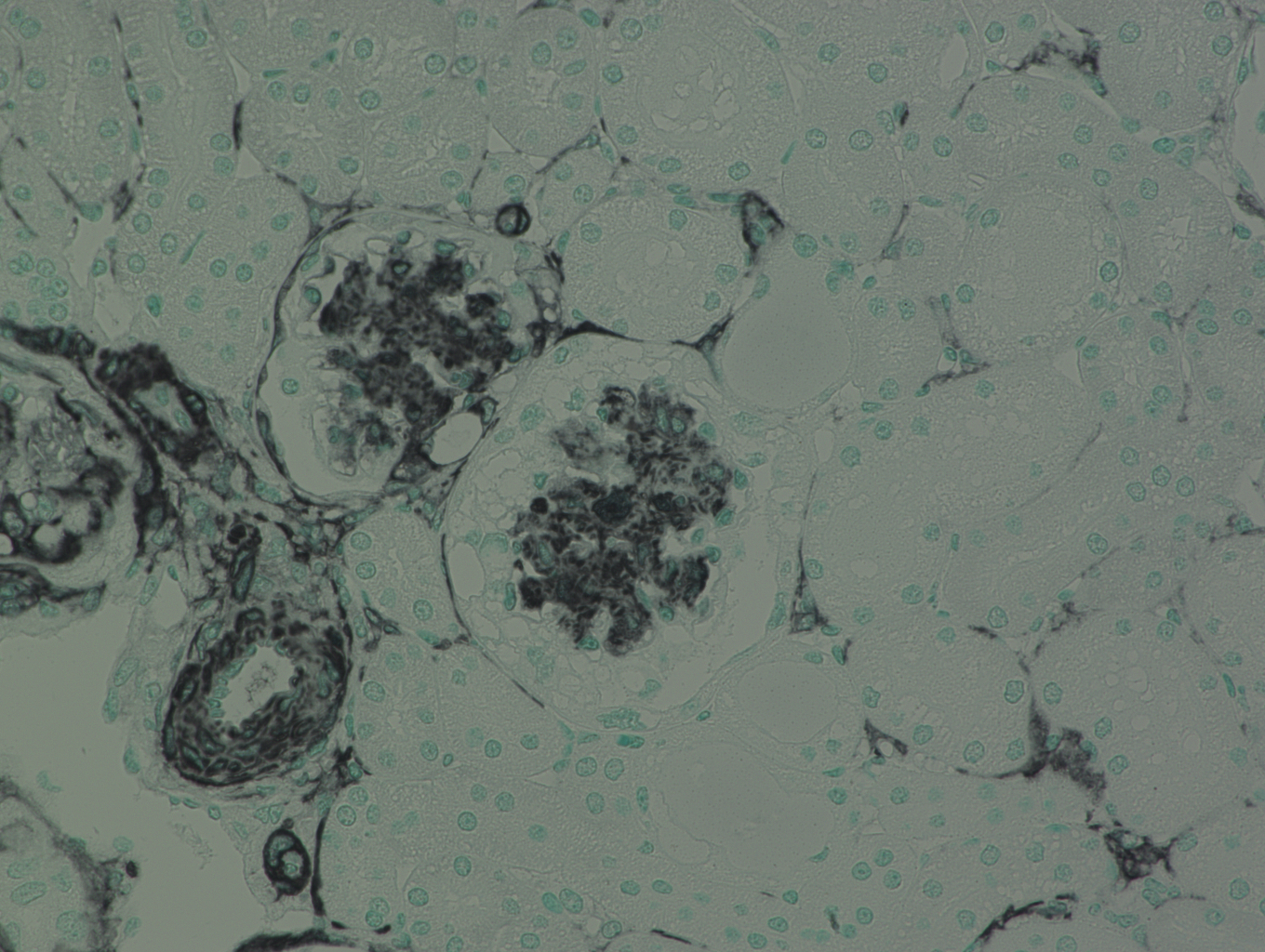

Supplement: Supplementary file 12 — Source Data for Figure 7 [file EMMM-12-e11021-s010.zip › SourceData_Fig7/Fig7G_mutant_AngII_asma.TIF]

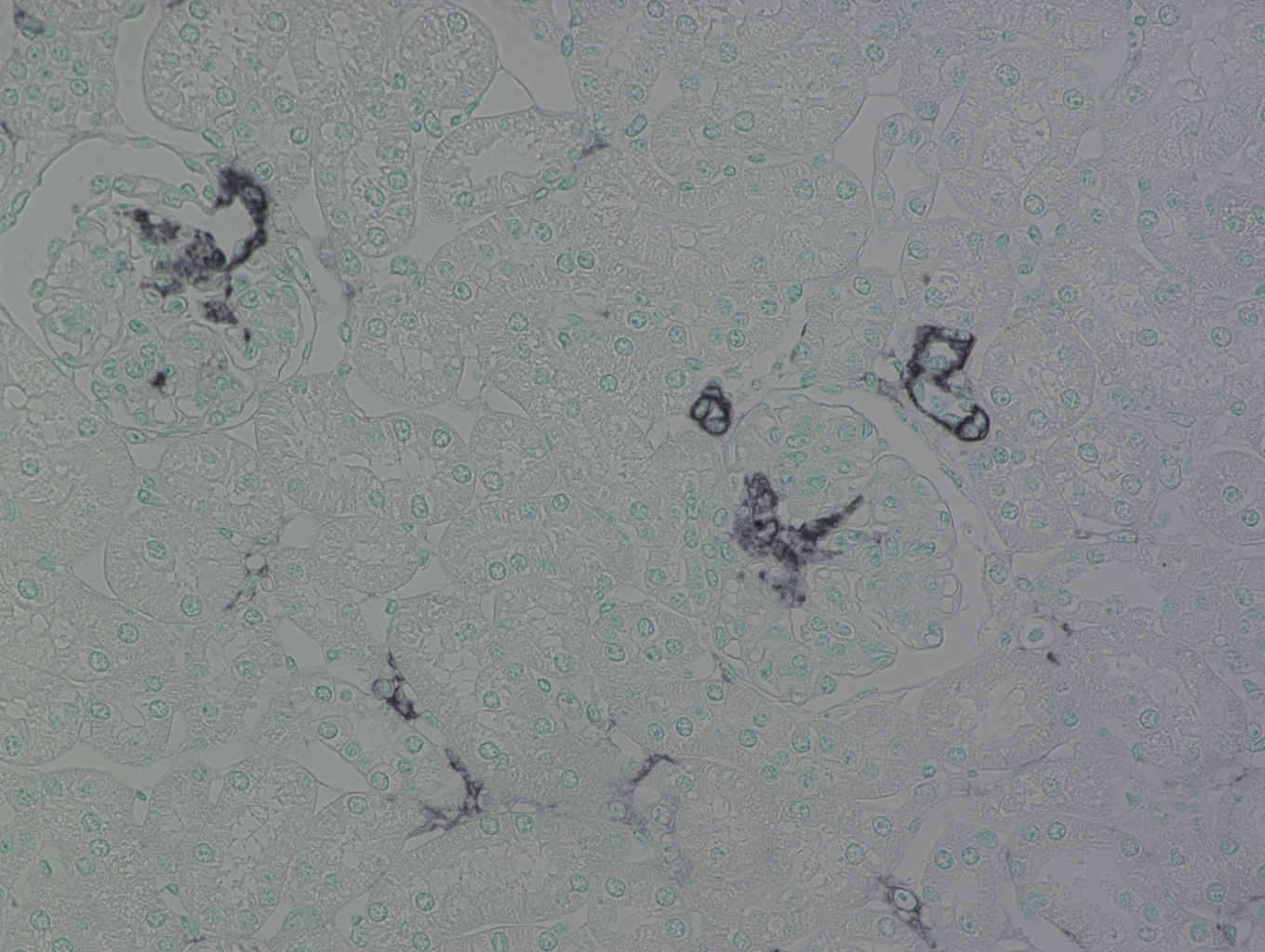

Supplement: Supplementary file 12 — Source Data for Figure 7 [file EMMM-12-e11021-s010.zip › SourceData_Fig7/Fig7G_mutant_ctrl_asma.TIF]

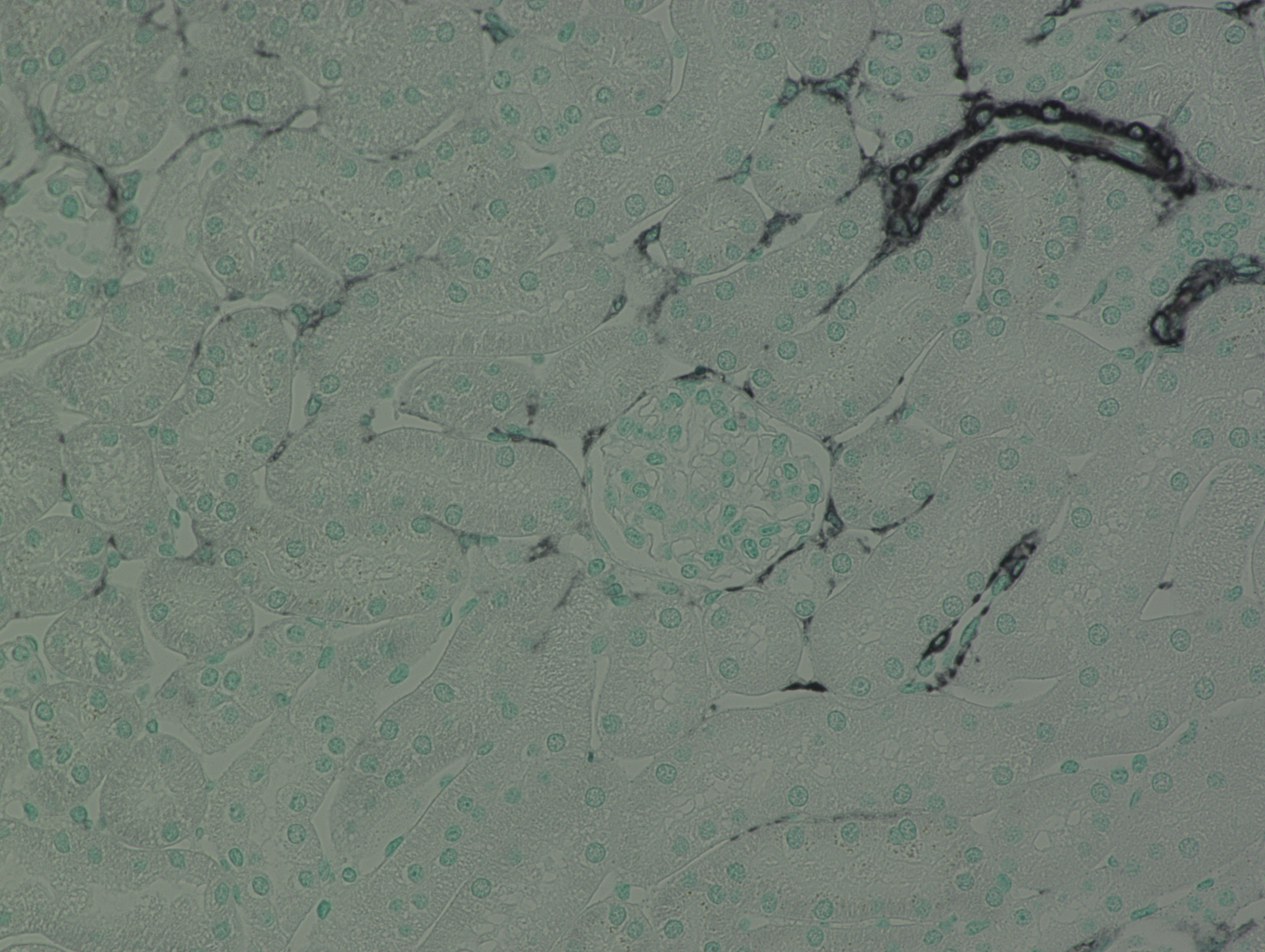

Supplement: Supplementary file 12 — Source Data for Figure 7 [file EMMM-12-e11021-s010.zip › SourceData_Fig7/Fig7G_wt_AngII_asma.TIF]

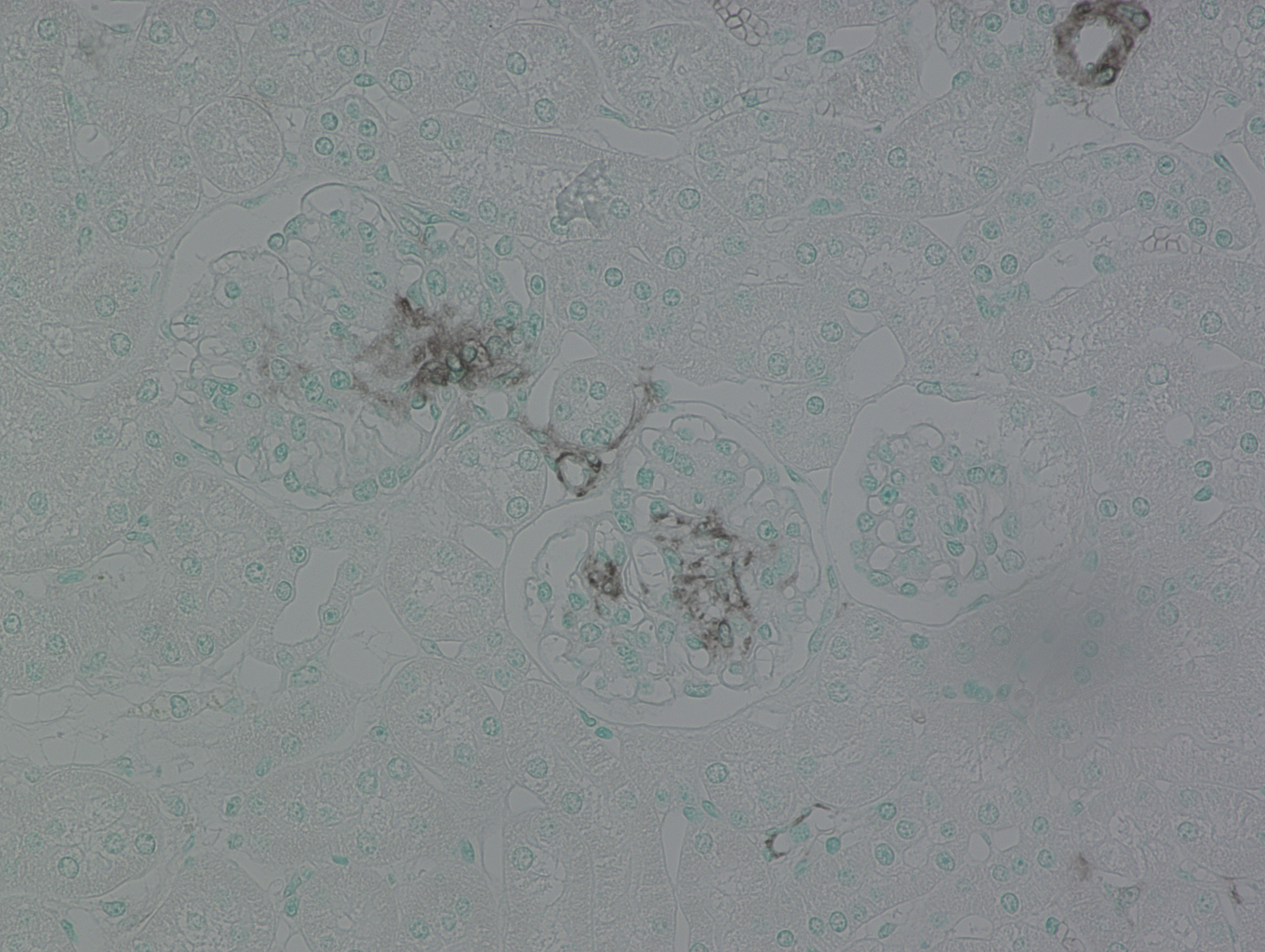

Supplement: Supplementary file 12 — Source Data for Figure 7 [file EMMM-12-e11021-s010.zip › SourceData_Fig7/Fig7N_imatinib_asma.TIF]

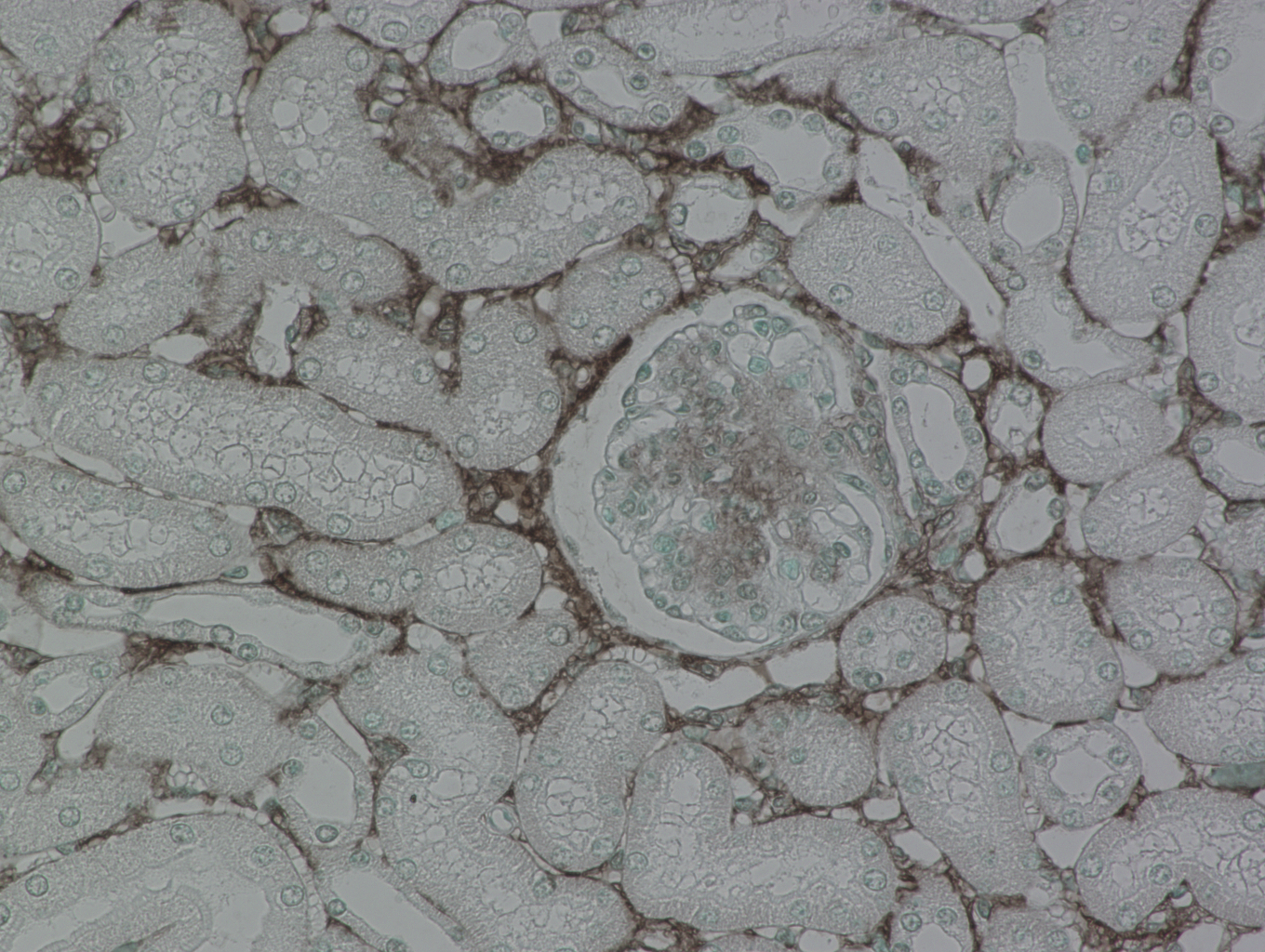

Supplement: Supplementary file 12 — Source Data for Figure 7 [file EMMM-12-e11021-s010.zip › SourceData_Fig7/Fig7N_imatinib_glomerulus_col1.TIF]

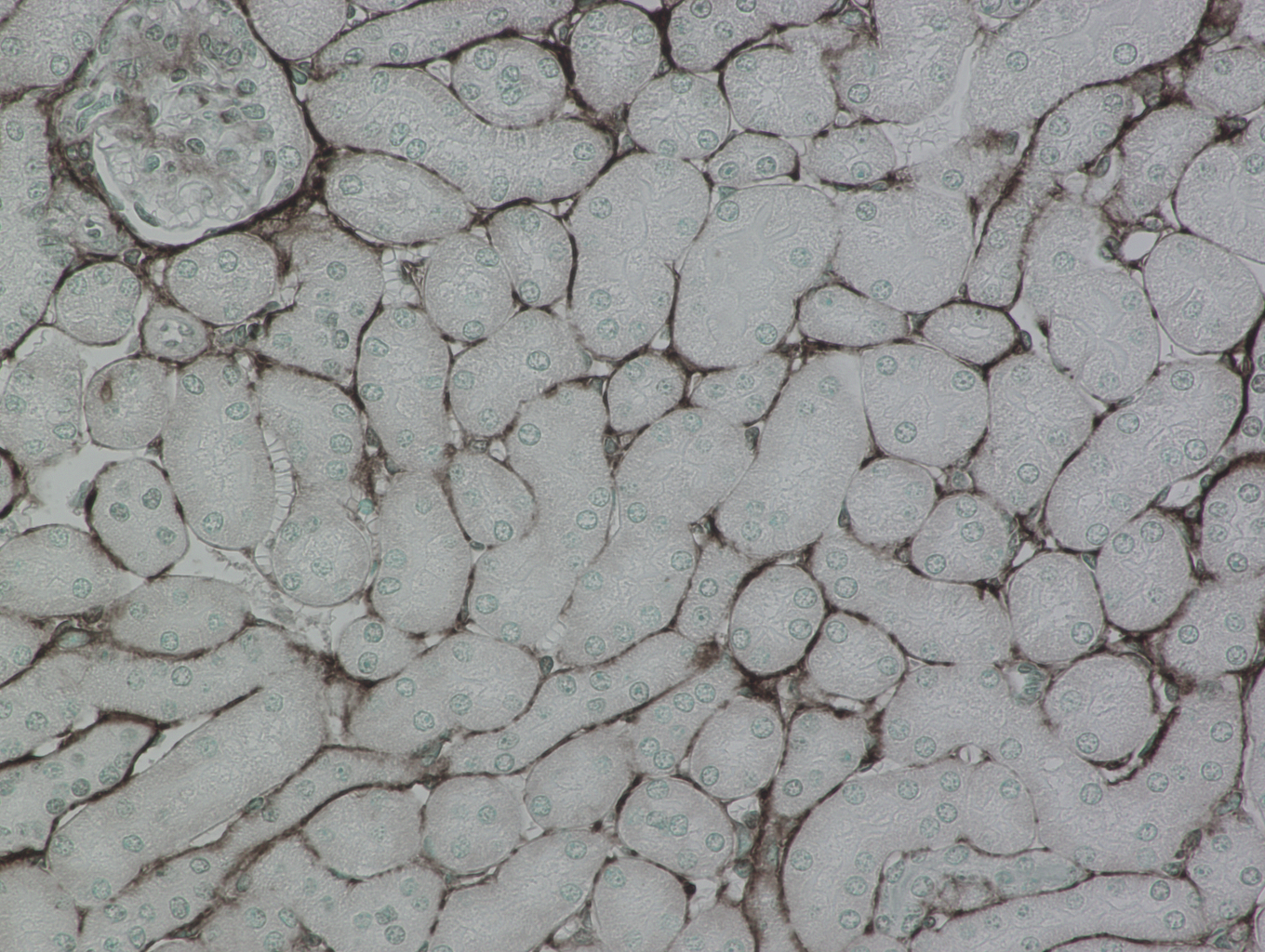

Supplement: Supplementary file 12 — Source Data for Figure 7 [file EMMM-12-e11021-s010.zip › SourceData_Fig7/Fig7N_imatinib_interstitium_col1.TIF]

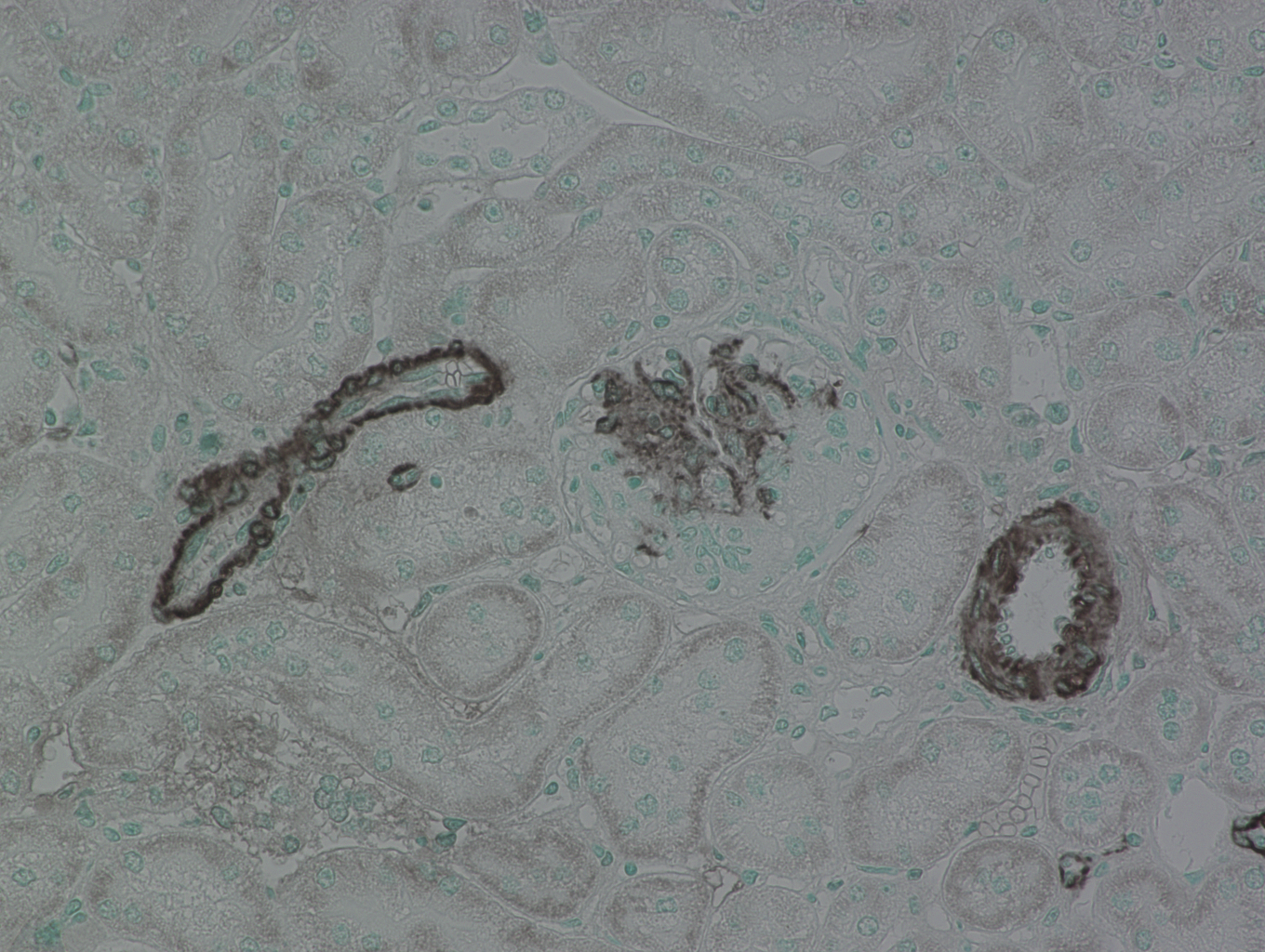

Supplement: Supplementary file 12 — Source Data for Figure 7 [file EMMM-12-e11021-s010.zip › SourceData_Fig7/Fig7N_water_asma.TIF]

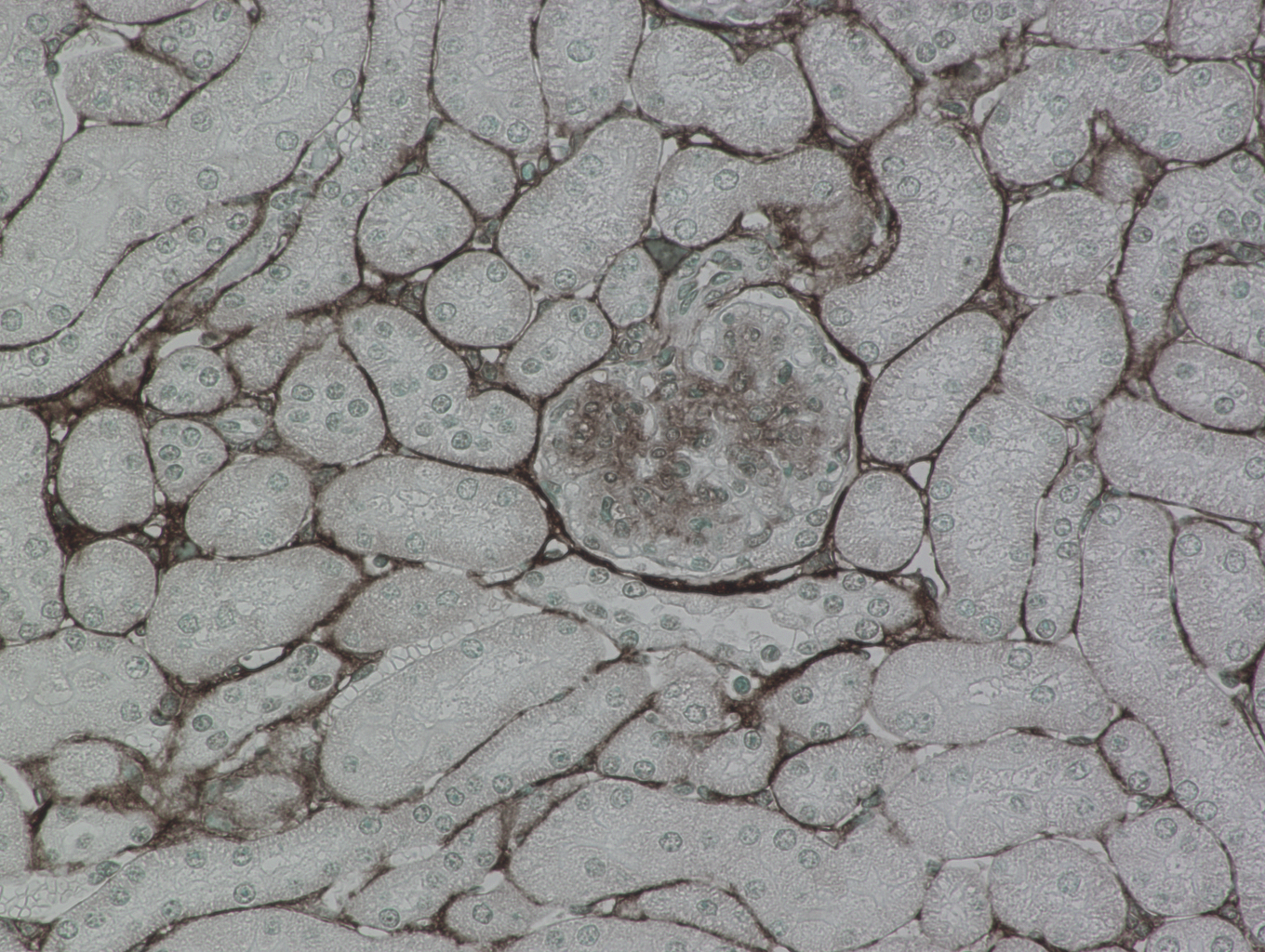

Supplement: Supplementary file 12 — Source Data for Figure 7 [file EMMM-12-e11021-s010.zip › SourceData_Fig7/Fig7N_water_glomerulus_col1.TIF]

**M**

|                                                                                    | <b>wt</b> | <b>Foxd1Cre::Pdgfrb<br/>+ water</b> | <b>Foxd1Cre::Pdgfrb<br/>+ imatinib</b> |
|------------------------------------------------------------------------------------|-----------|-------------------------------------|----------------------------------------|
| 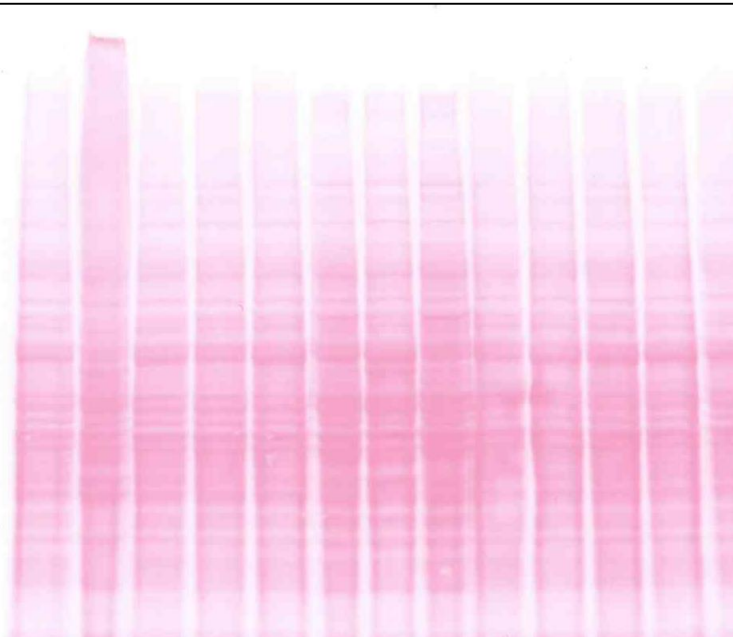 |           |                                     |                                        |

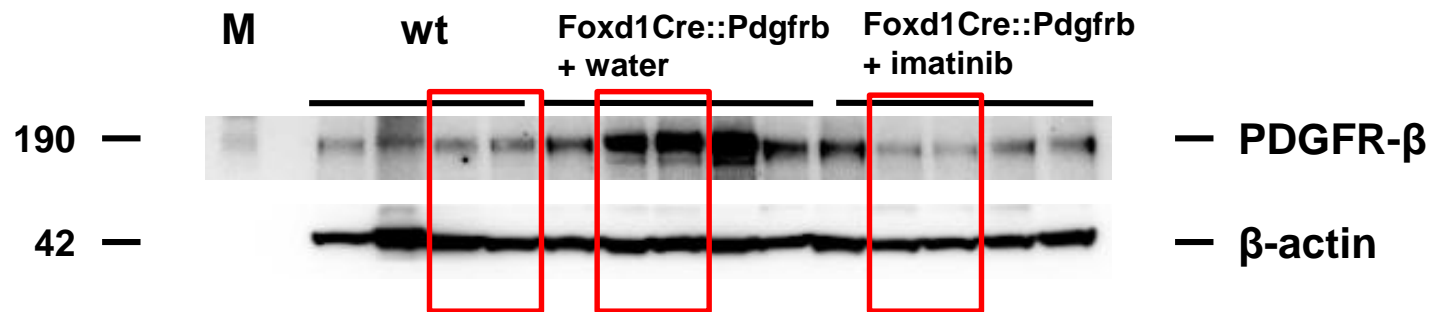

Supplement: Supplementary file 12 — Source Data for Figure 7 [file EMMM-12-e11021-s010.zip › SourceData_Fig7/Fig7O.pdf]
