# Supplementary material for: Dysregulated mesenchymal PDGFR‐β drives kidney fibrosis
Source: EMBO Mol Med. 2020 Jan 14;12(3):e11021. doi: 10.15252/emmm.201911021 (PMC7059015; doi:10.15252/emmm.201911021)
Supplement: Supplementary file 8 — Source Data for Figure 2 [file EMMM-12-e11021-s006.zip › SourceData_Fig2/Fig2_single_values.pdf]

Figure 2C

	value 1	value 2	value 3	p-value
week 6 wt	0.5	1.0	1.096774	0.2169
week 6 Foxd1Cre::Pdgfrb+/J	4.805555	1.979592	0.945946	
week 14 wt	0.1923077	0.3695652	0.1666667	0.0027
week 14 Foxd1Cre::Pdgfrb+/J	1.027778	0.7692308	1.0	
week 25 wt	0.24	0.2765957	n.a.	0.0621
week 25 Foxd1Cre::Pdgfrb+/J	1.432432	1.264151	0.6734694	
week 35 wt	0.5	0.1842105	0.1304348	0.0758
week 35 Foxd1Cre::Pdgfrb+/J	0.44	0.9111111	1.119048	

Figure 2D

	value 1	value 2	value 3	p-value
week 6 wt	11.875	22.875	11.57143	0.1460
week 6 Foxd1Cre::Pdgfrb+/J	35.0	40.375	15.875	
week 14 wt	3.875	3.5	5.625	0.0438
week 14 Foxd1Cre::Pdgfrb+/J	11.625	16.125	27.875	
week 25 wt	1.25	2.125	n.a.	0.0870
week 25 Foxd1Cre::Pdgfrb+/J	22.0	9.625	11.25	
week 35 wt	3.25	0.625	2.285714	0.0664
week 35 Foxd1Cre::Pdgfrb+/J	4.375	11.0	15.25	

Figure 2E

	value 1	value 2	value 3	p-value
week 6 wt	20.875	14.125	11.142	0.3517
week 6 Foxd1Cre::Pdgfrb+/J	24.625	28.875	11,5	
week 14 wt	2.0	7.142857	16.28572	0.3419
week 14 Foxd1Cre::Pdgfrb+/J	4.375	3.25	4.25	
week 25 wt	1.625	0.75	n.a.	0.5634
week 25 Foxd1Cre::Pdgfrb+/J	2.5	2.625	0.375	
week 35 wt	2.25	1.875	3.714286	0.2492
week 35 Foxd1Cre::Pdgfrb+/J	0.75	0.25	2.875	

Figure 2G

	value 1	value 2	value 3	p-value
wt	28.33333	33.5	24.6	0.0446
Foxd1Cre::Pdgfrb+/J	36.0	39.66667	47.33333	

Figure 2F

	value 1	value 2	value 3	value 4	p-value
Fibroblasts wt	0.356	0.494	0.407	0.485	< 0.0001
Fibroblasts Foxd1Cre::Pdgfrb+/J	0.8495	0.9385	1.0765	0.9705	
Mesangial cells wt	0.27025	0.25825	0.47425	0.44525	< 0.0001
Mesangial cells Foxd1Cre::Pdgfrl	0.976	0.926	1.025	1.004	
